# Estimation of the Global Disease Burden of Depression and Anxiety between 1990 and 2044: An Analysis of the Global Burden of Disease Study 2019

**DOI:** 10.3390/healthcare12171721

**Published:** 2024-08-29

**Authors:** Jinnan Liu, Wei Ning, Ning Zhang, Bin Zhu, Ying Mao

**Affiliations:** 1 School of Public Policy and Administration, Xi’an Jiaotong University, Xi’an 710049, China; 2 International Centre for Reproductive Health, Ghent University, 9000 Ghent, Belgium; 3 Vanke School of Public Health, Tsinghua University, Beijing 100084, China; 4 School of Public Health and Emergency Management, Southern University of Science and Technology, Shenzhen 518055, China

**Keywords:** depression, anxiety, disease burden, risk factor, prediction

## Abstract

(1) Background: Depression and anxiety are the most common and severe mental disorders. This research estimated the prevalence and disease burden of depression and anxiety from 1990 to 2044. (2) Methods: Data on disease burden, population, and risk factors were identified and gathered from the Global Health Data Exchange database. The time trends, sex and age differences, key factors, and regional variations in and predictions of depression and anxiety were analyzed based on the age-standardized incidence rate, prevalence rate, and DALY rate. (3) Results: Our findings revealed that the burden of depression and anxiety was heavy. Specifically, the age-standardized DALY rate of depression started to decrease compared with trends related to anxiety disorders. Meanwhile, females bear a heavier burden for both depression and anxiety. Seniors and the middle-aged population carry the highest burden regarding mental disorders. Both high- and low-socio-demographic-index countries were found to be high-risk regions for depressive disorders. The disease burden attributed to childhood sexual abuse, bullying victimization, and intimate partner violence has increased since 1990. Finally, projections regarding depression and anxiety revealed geographic and age variations. (4) Conclusions: Public health researchers, officers, and organizations should take effective age-, sex-, and location-oriented measures.

## 1. Introduction

Mental disorders have been identified as a leading contributor to disease burden by various global and national health institutes, emphasizing the critical need for comprehensive data and analytics to better understand and combat these issues. The Global Health Data Exchange (GHDx) provides an invaluable opportunity in this regard, offering extensive datasets that enhance our understanding of the trends and drivers behind mental health challenges. As the most frequent and serious psychological disorders, depression and anxiety are key areas where the GHDx’s data prove indispensable. In 2019, approximately 301 million people were affected by anxiety disorders, and about 280 million suffered from depression, and it is estimated that mental health disorders will account for the largest proportion of the global disease burden by 2030 [1,2]. The lifetime risk of depression and anxiety is approximately 15–18%, which means that almost one in five people experience one episode at some point in their lifetime [3]. Even worse, suicide can be linked to depression and anxiety [4]. It is estimated that the prevalence of depression and anxiety will continue to increase [5]. Utilizing the GHDx’s rich datasets, our study analyzes the prevalence and impact of these disorders, guiding targeted interventions and policymaking.

Risk factors such as individual (sex [6], age [7], education [8], income [9], and physical health [10]), family (family structure [11], family history [12], and family interactions [13]), social (social norms [14], socioeconomic class [15], and social support [16]), cultural (religion [17], moral values [18], and traditions [19]), and genetic and biological factors [20] are the main contributors to depression and anxiety. Furthermore, new underlying factors and events have gradually been identified and analyzed by researchers worldwide. A large amount of research has increasingly documented the potential effects of childhood sexual abuse [21,22], bullying victimization [23,24], and intimate partner violence [25,26] on depression and anxiety. Meanwhile, emergent events such as the coronavirus disease outbreak have dramatically increased the disease burden of depression and anxiety globally and regionally. Recent studies have documented significant impacts on mental health due to the pandemic, highlighting the escalation in depression and anxiety rates across various demographics [26,27].

The focus of treating depression and anxiety is the remission of symptoms through psychological therapy, pharmacotherapy, or both [28,29]. However, on the one hand, the treatment course is an extremely long and repetitive process that poses threats to patients’ recovery [30]. On the other hand, treatment methods may vary according to sex, age, country, and other factors [31]. Therefore, projecting and estimating disease burden are requirements for the enactment and implementation of population-specific measures.

While the prevalence and burden of depression and anxiety have been explored by previous research, systematic analyses that integrate predictions of burden alongside risk factors remain limited [1,2]. Our study addresses this gap by examining the global burden of these mental disorders through the lenses of age, sex differences, temporal trends, and geographic location and by predicting their future trajectory using a Bayesian age–period–cohort (APC) model. The findings of this study are crucial for informing targeted interventions and public health policies aimed at mitigating the impact of depression and anxiety. Moreover, the insights gained here lay the groundwork for future research that will explore the evolving nature of these disorders, particularly in the wake of the COVID-19 pandemic. The implications of our study extend beyond academic research, offering practical guidance for public health initiatives and policymaking.

## 2. Methods

### 2.1. Data Source

The Global Burden of Disease (GBD) 2019 Diseases and Injuries Collaborators have expanded on full-scale primary data sources and estimation methods [32]. Data on depressive and anxiety disorders were identified and extracted based on sex and age groups in 204 countries and regions between 1990 and 2019 at the Institute for Health Metrics and Evaluation (http://ghdx.healthdata.org/gbd-results-tool (accessed on 15 May 2023)). Specifically, the database sorted national data into five groups (countries and territories with low, low–middle, middle, high–middle, or high SDI) according to the socio-demographic index (SDI). This index is a synthesized measurement of income per person, years of education, and fertility. Furthermore, the continents were categorized into seven GBD areas, such as North Africa and the Middle East, Latin America and the Caribbean, and sub-Saharan Africa, which could be divided into 21 GBD subregions, such as East Asia, South Asia, and Western Europe. Finally, there is well-established research and analysis on the risk factors for depressive and anxiety disorders [33,34]. Childhood sexual abuse, bullying victimization, and intimate partner violence have been proven to have a causal association with depression, while bullying victimization could be a risk factor for anxiety, according to an estimation [35,36,37].

### 2.2. Statistical Analysis

In our analysis, raw data on the incidence, prevalence, and DALYs of depressive and anxiety disorders were initially extracted and categorized by region, age, and sex. To facilitate comprehensive comparisons across different demographics and ensure robustness in our results, we subsequently processed these data into age-standardized rates (ASIR, ASPR, and DALY rates). The 95% uncertainty intervals (UI) for these metrics were calculated using the 25th- and 75th-order values from 1000 random draws of a posterior distribution. We extracted the available data for three risk factors: childhood sexual abuse, bullying victimization, and intimate partner violence. To assess the impact of specific risk factors on DALYs, we utilized a comparative risk assessment framework. This involved attributing changes in DALYs to these factors based on their relative risk ratios derived from the literature, with regression analysis adjusting for key confounders such as socioeconomic status and previous health conditions. The age-standardized rates (ASIR, ASPR, and DALYs) facilitated robust comparisons across demographics.

In addition, this research performed correlation analysis to qualify the relationship between standardized DALY rates and the SDI of different countries and territories. We used standardized DALY rates of different gender and age groups in 2019 to identify the impact of different attributes.

The prediction of depression and anxiety was divided into two parts: global estimation and country forecasting. At the global level, we conducted Bayesian APC analyses and adjusted the original data according to the model set. The whole population was classified into 18 age groups (0–10, 10–14, 15–19, 20–24, 25–29, 30–34, 35–39, 40–44, 45–49, 50–54, 55–59, 60–64, 65–69, 70–74, 75–79, 80–84, 85–89, and 90 years old and above). The research period was divided into six groups in 5-year intervals (1990–1994, 1995–1999, 2000–2004, 2005–2009, 2010–2014, and 2015–2019). The incidence and rate of depression and anxiety in the 18 age groups were predicted for 2020 to 2044. For the country forecast, the population and observation period were altered to specify and model requirements such as global estimation. We only present the estimation results for each country and GBD region.

### 2.3. Software

This study constructed Bayesian APC through R (version 4.1.12) to project depression and anxiety from 2020 to 2044. Furthermore, the map figures were drawn using ArcMap 10.8, and the other figures were created using OriginPro (version 2020b).

## 3. Results

### 3.1. Burden and Time Trends for Depressive and Anxiety Disorders

**Figure 1:**
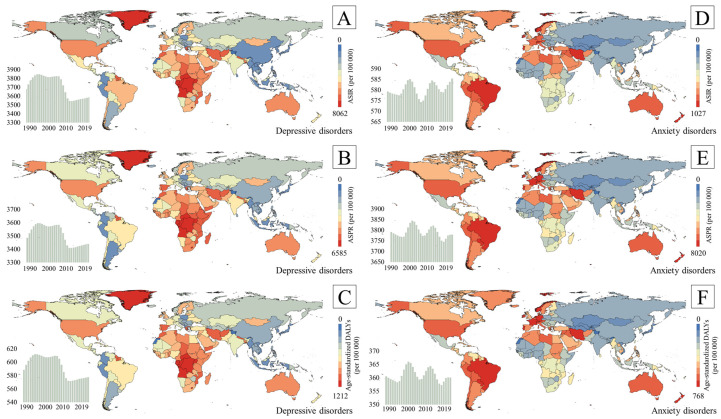
Temporal and spatial distribution of ASIR, ASPR, and age-standardized DALY rates of depressive and anxiety disorders (per 100,000). ((**A**). The temporal and spatial distribution of ASIR of depressive disorders; (**B**). The temporal and spatial distribution of ASPR of depressive disorders; (**C**). The temporal and spatial distribution of age-standardized DALY rates of depressive disorders; (**D**). The temporal and spatial distribution of ASIR of anxiety disorders; (**E**). The temporal and spatial distribution of ASPR of anxiety disorders; (**F**). The temporal and spatial distribution of age-standardized DALY rates of anxiety disorders).

In 2019, depressive disorders accounted for 46.86 million (95% UI 32.93–63.80) DALYs globally, equating to an age-standardized DALY rate of 577.75 (405.79–788.88) per 100,000. This rate has decreased by 4.97% since 1990. Similarly, ASIR and ASPR dropped significantly. There were large geographic differences in the depression DALYs in 2019 (see Table 1 and Figure 1). The highest age-standardized DALY rate was 786.51 (549.28–1083.35) per 100,000 in sub-Saharan Africa, followed by North Africa and the Middle East (781.06 [535.18–1075.62] per 100,000) and high-income countries (647.16 [451.97–882.46] per 100,000). The lowest age-standardized depression DALY rate was recorded in Southeast Asia, East Asia, and Oceania (415.06 [290.21–569.35] per 100,000). In addition, apart from the high-SDI regions, the age-standardized depression DALY rates of the other SDI groups have decreased dramatically since 1990.

In 2019, the global DALY rate for anxiety disorders was 28.68 million (95% UI 19.86–39.32), corresponding to an age-standardized DALY rate of 360.12 (248.60–494.44) per 100,000. In contrast to the findings for depressive disorders, the rate fluctuated and finally stabilized at a similar rate compared to that in 1990, only rising by 2.13% (Table 2 and Figure 1). The majority of the anxiety disease burden, unlike that of depression, was observed in Latin America and the Caribbean (524.13 [361.15–721.08] per 100,000), North Africa, and the Middle East (492.15 [333.99–685.31] per 100,000), and high-income countries (480.87 [330.35–657.48] per 100,000). The lowest age-standardized anxiety DALY rate was found in Central Europe, Eastern Europe, and Central Asia (285.83 [197.74–392.62] per 100,000). In addition, the age-standardized anxiety DALY rates of the high-SDI and high–middle-SDI regions showed an upward trend, whereas other SDI groups displayed a downward trend.

The crude and age-standardized prevalence, incidence, and DALY rates of depression and anxiety in different countries and regions in 2019 are presented in Supplementary Materials S1 in the Supplementary Materials.

### 3.2. Variation in the Burden of Depressive and Anxiety Disorders in Sex and Age Groups

From a sex perspective, females were at a higher risk of suffering from depression or anxiety than their male counterparts. In 2019, the age-standardized DALY rate for females (702.08 [492.30–963.58] per 100,000) was over 1.5 times larger than that of males (452.17 [316.79–618.13] per 100,000) for depression. Globally, the group aged 25–45 years had the greatest absolute burden of depression, accounting for 36.82% of total DALYs. The age-specific depression DALY rate was highest in the population aged 60–64 years, at 957.68 (634.59–1344.88) per 100,000, followed by those aged 55–59 years, at 948.15 (641.79–1349.08), and those aged 65–69 years, at 932.88 (637.67–1277.20). The lowest depression DALY rate was observed among children aged 0–14, at 66.03 (37.75–111.36), followed by adolescents aged 15–19, at 498.68 (311.69–759.02). More specifically, females aged 60–64 (1158.58 [768.13–1623.72] per 100,000), 55–59 (1156.08 [783.51–1638.74] per 100,000), 50–54 (1118.17 [780.07–1580.16] per 100,000), and 65–69 years (1112.19 [758.67–1516.92] per 100,000) experienced significantly higher disease burden than all other age groups in men and women.

Similarly, for anxiety, the age-standardized DALY rate for women (444.89 [307.26–609.32] per 100,000) is around 1.6 times that of men (275.20 [191.39–377.89] per 100,000). In terms of age, the largest absolute burden of anxiety was detected in children aged 0–14, constituting over 12% of total DALYs. Conversely, this age group showed a low anxiety DALY rate, 156.79 (94.59–244.41) per 100,000. The largest anxiety DALY rate was discovered in the middle-aged group, namely, 40–44 (469.04 [300.73–685.62] per 100,000), 35–39 (468.29 [302.38–669.00] per 100,000), and 45–49 years old (459.23 [299.90–669.54] per 100,000). Likewise, a detailed analysis of sex and age group differences showed that the highest anxiety DALY rate was found in females aged 35–39, 40–44, and 45–49 years.

**Figure 2:**
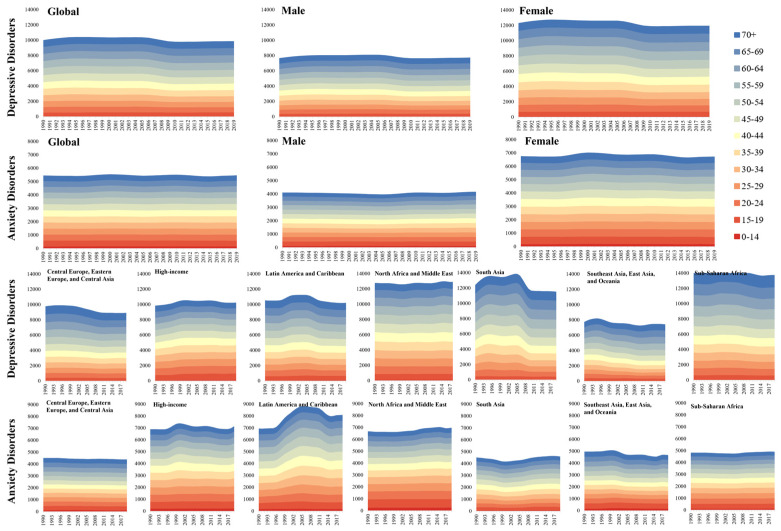
Age-standardized DALY rate for depression and anxiety from 1990 to 2019 based on sex, age group, and region (per 100,000).

Figure 2 illustrates the sex and age differences in the DALY rates globally and regionally from 1990 to 2019. The crude and age-standardized prevalence, incidence, and DALY rates of depression and anxiety of different sexes from 1990 to 2019 are shown in Supplementary Materials S2 in the Supplementary Materials.

### 3.3. Burden of Depressive and Anxiety Disorders in Different SDI Countries

**Figure 3:**
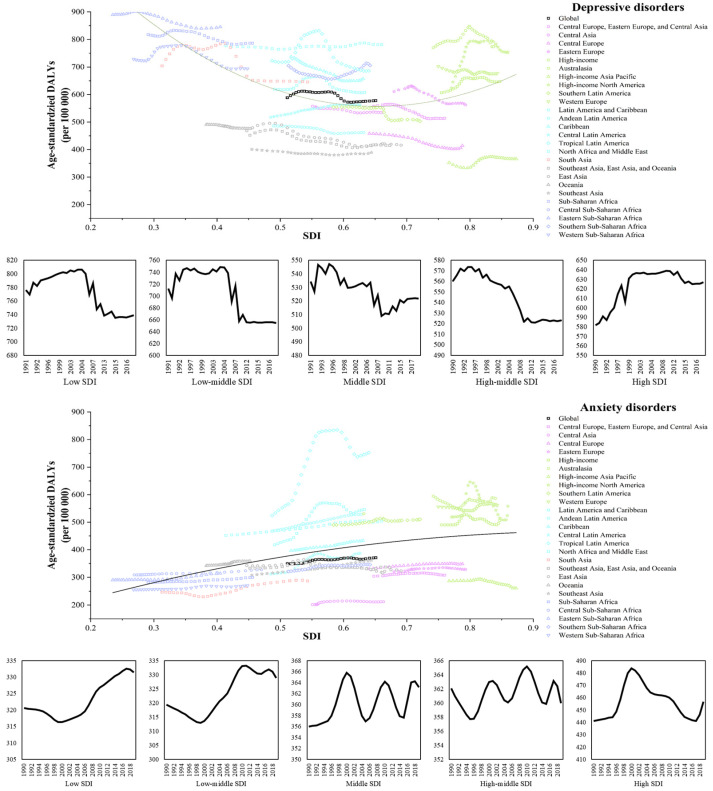
Age-standardized DALY rates for depression and anxiety regionally according to SDI, 1990–2019 (per 100,000).

The correlation between the age-standardized rate of disease and SDI is shown in Figure 3. Regarding depression, different continents displayed various relationships. Central Europe, Eastern Europe, and Central Asia were found to have middle SDIs and low disease burden with only a few outliers, while the worst of all, sub-Saharan Africa, showed high-risk and underdeveloped characteristics. It is notable that apart from high-income Asia, other high-income regions had a notable disease burden of depression. The Latin American and Caribbean subgroups showed completely different disease burdens.

Regarding anxiety, high-income countries and Latin American and Caribbean countries were found to have both a significantly higher disease burden and higher SDI, while sub-Saharan Africa showed low disease burden and low SDI. Simultaneously, Central Europe, Eastern Europe, Central Asia, East Asia, and Southeast Asia had relatively high SDI and low disease burden.

The age-standardized DALY rates for depression and anxiety in different countries according to SDI from 2019 are shown in Supplementary Materials S3 in the Supplementary Materials.

### 3.4. Attributable Risk Factors of Depressive and Anxiety Disorders

Of the global DALYs caused by depressive disorders in 2019, it is estimated that 2.05 (0.96–3.56) million, or 25.11 (11.72–43.38) per 100,000, were attributable to childhood sexual abuse, accounting for 4.36% (2.37–6.79%) of total DALYs. The proportion of DALYs caused by childhood sexual abuse increased globally between 1990 and 2019. Notably, the burden of depression caused by this factor was higher in women than in men. Furthermore, this risk factor predominantly affected middle-aged and older adults, especially in the age groups 40–44 (47.12 [18.15–90.32] per 100,000), 45–49 (45.89 [18.66–88.86] per 100,000), and 50–54 (43.88 [17.91–83.57] per 100,000) years.

The second identified risk factor was bullying victimization, which leads to depressive and anxiety disorders. For depression, 1.71 (0.35–4.00) million, or 22.46 (4.77–52.18) per 100,000, were ascribed to bullying victimization in 2019, making up 3.87% (0.96–8.08) of global DALYs. Similarly, the DALY rate caused by bullying victimization dramatically increased from 1990 to 2019 by nearly 200%. The burden of depression in females was still much higher than in males. Predictably, mostly children, adolescents, and younger adults (0–39 years) were affected by this factor, particularly the age groups 15–19 (80.17 [24.94–164.39]), 20–24 (79.72 [13.74–180.48]), and 25–29 years (50.30 [0.00–133.36]). For anxiety, bullying victimization in 2019 was at 2.03 (0.59–4.42) million, or 26.93 (8.02–58.28) per 100,000, constituting 7.47% (2.31–15.15%) of global DALYs. Likewise, the DALY rate due to bullying victimization increased by 23.31% from 1990 to 2019. The burden of anxiety in different sex and age groups was similar to that of depression. The populations aged 15–19 (87.09 [30.71–175.22]), 20–24 (69.27 [16.96–150.86]), and 25–29 years (47.70 [3.11–121.53]) bore a heavy burden of anxiety due to bullying victimization.

Intimate partner violence largely exacerbates depressive disorders. It is estimated that the burden of depression attributable to intimate partner violence is 3.16 (0.13–6.92) million or 38.65 (0.17–84.35) per 100,000, accounting for 6.70% (0.03–14.54%) of the total burden of depression. In particular, the percentage of DALYs resulting from intimate partner violence increased from 1990 to 2019. Only the burden of depression for women was calculated for intimate partner violence, and the highest DALY rate age group was 50–54 (135.69 [0.62–340.39] per 100,000), followed by 55–59 (134.99 [0.58–323.57] per 100,000) and 45–49 (131.77 [0.66–317.92]) years.

**Figure 4:**
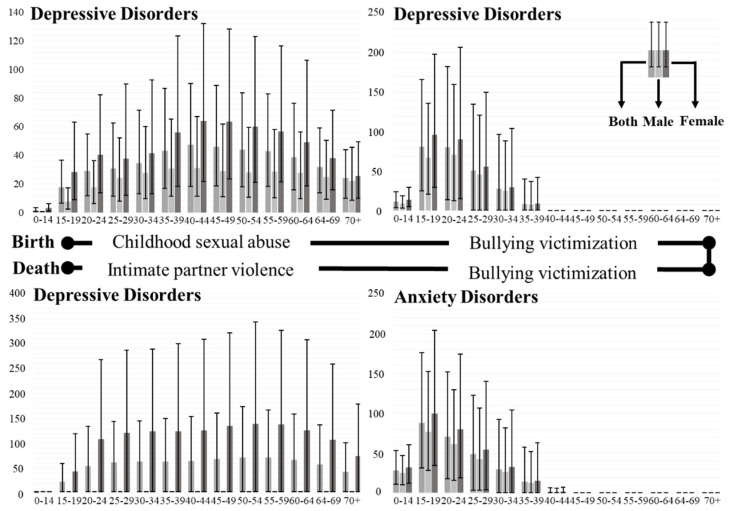
Age-standardized DALY rate attributed to various risk factors based on age and sex group in 2019.

Figure 4 shows the age-standardized DALY rate of different attributing factors according to age and sex in 2019. Specifically, the three risk factors of different age groups according to sex in 2019 are shown in Supplementary Materials S4 in the Supplementary Materials.

### 3.5. Predication of Incidence Rate of Depression and Anxiety Disorders

**Figure 5:**
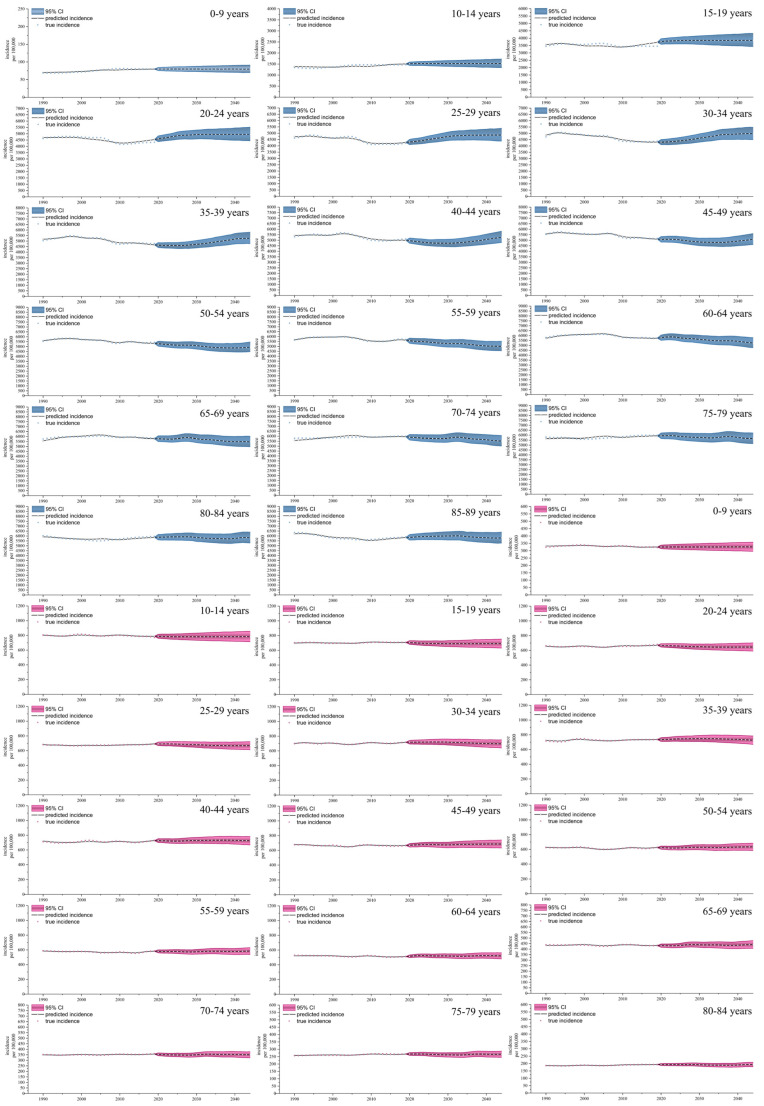
Global incidence of depression and anxiety projections for age groups, 2020–2044 (per 100,000).

Figure 5 shows the global age-specific incidence projections for depression and anxiety in 2020–2044 for the age groups 0–89 and 0–84 years. Regarding depression, the ASIR is predicted to change in a different manner compared to the 1990 to 2019 period. The depression incidence rate of the 0–14 years age group is expected to remain stable compared to that of 1990, while the depression ASIR of the 15–39 years age group will continue to rise. Furthermore, the ASIR of the 40–89 years age group will gradually decrease. Age and the predicted ASIR were positively correlated. The highest age group is 85–89 years, at 5884.61 (5293.91–6541.23) per 100,000, followed by 75–79 (5475.80 [4937.36–6083.13] per 100,000), 80–84 (5473.01 [4922.38–6086.81] per 100,000), and 70–74 years (5266.62 [4733.43–5862.00] per 100,000). The lowest rate was detected in the 0–9 years age group, at 79.78 (69.02–92.02), followed by 10–14 (1520.29 [1325.67–1734.92] per 100,000), 15–19 (3864.21 [3390.36–4370.49] per 100,000), and 25–29 years (4863.05 [4311.08–5441.05]).

Regarding anxiety disorders, the ASIR will remain stable until 2044 in different age groups. The highest rate was found in the 10–14 years age group with 781.43 (709.01–859.84) per 100,000, then the 35–39 (720.74 [661.57–784.66] per 100,000), 40–44 (711.61 [652.69–773.23] per 100,000), and 30–34 years age groups (694.68 [634.84–757.26] per 100,000). The lowest rate was in the 80–84 years age group with 192.12 (176.14–208.65) per 100,000, followed by the 75–79 (262.94 [241.06–285.92] per 100,000), 0–9 (325.87 [294.35–359.79] per 100,000), and 70–74 years age groups (356.07 [326.62–386.48] per 100,000).

**Figure 6:**
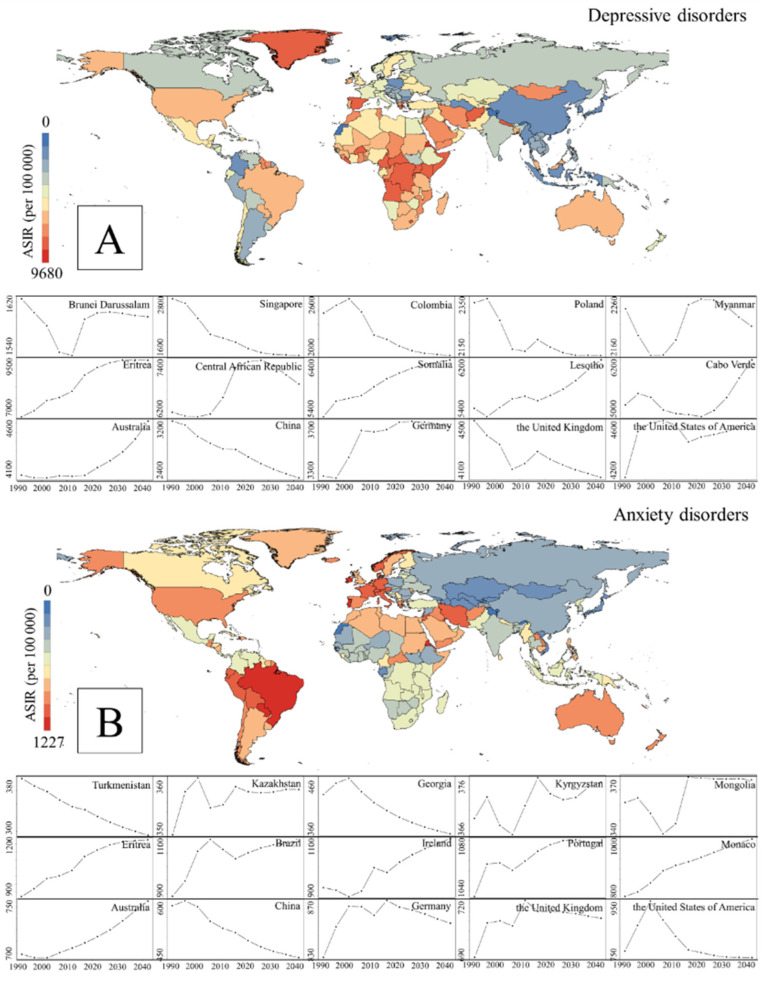
ASIR for depression and anxiety projections in all countries and trends of major economies (per 100,000). ((**A**). ASIR for depression projections in all countries and trends of major economies; (**B**). ASIR for anxiety projections in all countries and trends of major economies).

The geographical variations regarding the predicted ASIR for depression and anxiety were similar to those of the ASIR in 2019. More specifically, Figure 6 shows the ASIR of certain countries (the lowest five, highest five, and five selected countries). The depression projection revealed that Brunei Darussalam, Singapore, Colombia, Poland, and Myanmar were the countries with the lowest ASIR, while Eritrea, Central Africa Republic, Somalia, Lesotho, and Cabo Verde had the highest ASIR. Regarding anxiety, Turkmenistan, Kazakhstan, Georgia, Kyrgyzstan, and Mongolia were the five countries with the lowest ASIR, while Eritrea, Brazil, Ireland, Portugal, and Monaco had the highest ASIR. Regarding the five major economies in the world (Australia, China, Germany, the United Kingdom, and the United States), the Australian ASIR was relatively low compared with the other four countries.

The projections of depression and anxiety for all the recorded countries of the GBD can be found in Supplementary Materials S5 in the Supplementary Materials.

## 4. Discussion

In this study, we revealed several critical insights into the global, regional, and national burden of depressive and anxiety disorders from 1990 to 2019 and projected trends up to 2044. We found that while the age-standardized DALY rate for depression has generally declined by 4.97% globally, the burden of anxiety disorders has increased by 2.13%. This study also showed that women and older adults, particularly those aged 60–64, carry a significantly higher burden of depression, whereas the highest burden of anxiety was observed among middle-aged individuals (35–50 years). Furthermore, our findings underscore significant geographic disparities, with both high- and low-SDI countries showing distinct patterns of depression and anxiety burden driven by differing social, economic, and environmental factors. These results emphasize the importance of targeted public health strategies that address the specific needs of different populations and regions.

The ASIR, ASPR, and age-standardized DALY rate for depression showed a downward trend from 1990 to 2019, while the same for anxiety revealed an upward trend, which suggests that these trends align with the overall patterns observed in the GBD 2019 dataset [2]. These trends underscore the evolving landscape of mental health, where depression appears to be slightly mitigated by global health efforts, while anxiety disorders remain an area of growing concern. Specifically, the age-standardized DALY rate of depression has decreased by 4.97% since 1990 (577.75 per 100,000). This downward trend may reflect the effectiveness of global public health interventions aimed at mitigating depression. International organizations and governments have likely contributed to this reduction through enhanced mental health services, awareness campaigns, and early intervention programs [38,39]. However, this decline is not uniform across all regions, indicating that more targeted efforts are needed in areas where the burden remains high. The age-standardized DALY rate for anxiety disorders has increased by 2.13% since 1990 (360.12 per 100,000), which suggests that, unlike depression, anxiety has become a growing concern globally. This may be due to factors such as increasing life stressors, societal pressures, and possibly insufficient mental health resources or interventions specifically targeting anxiety [40]. The rise in anxiety could also be linked to the increasing awareness and diagnosis of anxiety disorders over the years [41]. Moreover, both show geographic disparities in age-standardized DALY rates. High-income regions continue to experience a significant burden of both depression and anxiety, potentially due to lifestyle factors and socioeconomic disparities [42]. Conversely, regions such as sub-Saharan Africa and Latin America exhibit high rates of depression and anxiety, respectively, highlighting the need for context-specific public health strategies.

Regarding sex differences, the burden of depressive and anxiety disorders was higher in women than in men. Previous studies have found that hormonal changes in women during puberty, menstruation, pregnancy, and perimenopause may lead to the onset or development of depressive and anxiety disorders [43]. Furthermore, hormonal fluctuations during stressful and traumatic life experiences can trigger the onset of these mental illnesses [44,45]. Apart from genetic or biological causation, recent research has increasingly documented the economic, social, and cultural factors of these differences. For example, females are likely to earn less [46], and a strong association between income and depressive and anxiety disorders has been reported [47]. Based on this analytic mechanism, females are more likely to be depressed or anxious than males.

Although the population aged 25–45 years old shared the greatest absolute burden of depression, the highest value of age-standardized DALYs was in the 60–64 years age group, followed by those aged 55–59 and 65–69 years old. The lowest ASIR was found in children aged 0–14 and adolescents aged 15–19 years. A systematic review reported that depressive symptoms in older adults were associated with sex, marital status, educational background, substance use, cognitive ability, functional ability, and comorbidities [48]. Some research found a difference between early-onset and late-onset depressive disorders [49]. Late-onset depression, in particular, is often linked to chronic physical health issues, making mental health interventions in older populations critical [50]. For instance, researchers found that elders with late-onset depressive disorders normally showed a higher risk of developing cardiovascular, gastrointestinal, and nervous system diseases compared to those with early-onset depression [49,51,52]. Meanwhile, late-life depression is often linked to the loss of a spouse, loneliness, and/or traumatic life experiences [53,54]. To some extent, these events lead to high DALYs in patients with depression. Although older adults report higher disease DALYs, younger people showed a more depressed mood in absolute burden terms. The working-age population (15–64 years old) accounted for a large proportion of the total population. Immature mindset, working stress, and family pressure are significantly associated with depression in the working-age population [55,56,57]. Notably, there are also multiple risk factors for younger people. While ASIR is lower in children and adolescents, there is a need for early preventive measures to address potential risk factors before they escalate into more severe mental health issues in later life.

Age differences regarding anxiety were completely different. The largest absolute burden of anxiety was observed in children aged 0–14 years old. However, this age group had a low anxiety DALY rate. The highest anxiety DALY rate was observed in the middle-aged group (35–50 years old). Our lives change as we age, increasing the chance of experiencing stressful life events: pressures due to raising one’s family and loneliness are closely related to anxiety disorders in the middle-aged population [58]. It is worth noting that anxiety in children and adolescents has dramatically increased since 1990 [59]. School bullying, difficulties in managing studies, worries related to weight and appearance, difficulty making friends, and poor family relationships were associated factors [60].

Regarding national differences by SDI, among both the low- and high-SDI countries, apart from high-income Asia, we found a high depression burden. The reasons behind low- and high-SDI countries’ depression burdens were different. In high-SDI countries, the high burden of depression may be related to lifestyle factors, income inequality, personal debt, and overthinking driven by high socioeconomic status. With remarkable improvement in people’s living standards, citizens begin to overthink, which can provoke anxiety and feelings of depression [61]. In addition, research indicates that while income inequality can contribute to mental health disparities [62], other factors, such as personal debt, have a much stronger association with these disorders [63]. Sedentary lifestyles, poor diet, and lack of physical activity, which are more common in affluent societies, can also negatively impact mental health [64,65]. Previous research found a “poverty trap” in low-SDI countries; poverty leads to worrying about life, poor physical health and early life conditions, social violence, high crime rates, and low social class. These issues commonly lead to depression, which could be attributed to developmental immaturity in labor supply, children and adolescents, preferences and beliefs, and economic decision-making, which also leads to poverty. This is the vicious circle of poverty and depression [66]. However, we found that low-SDI countries presented a low disease burden for anxiety, which is consistent with previous studies [62,67]. This could be attributed to different societal pressures and possibly lower rates of diagnosis and reporting [68]. In high-income countries, anxiety is more prevalent due to competitive environments and unequal living conditions [69], suggesting that mental health interventions should consider the socioeconomic dynamics of the population. Notably, East Asia, Southeast Asia, and Oceania have reported low disease burdens for both depressive and anxiety disorders. The underreporting and stigma associated with mental health in East Asia and Southeast Asia likely contribute to the lower reported burden [70]. Some research suggests that mental health has not been prioritized in Southeast Asian countries; hence, the rates of mental health in that region are typically underestimated because of data scarcity [71]. These findings suggest a need for increased mental health awareness and improved data collection in such regions to provide a more accurate assessment of the mental health burden. Meanwhile, mental health-related stigma is prevalent in East Asian countries such as China, Japan, and South Korea [72,73,74]. Recent research has revealed that 17.5% of the population in China suffers from at least one mental disease, which is much higher than the GBD data. Research indicates that the majority of mentally ill individuals have never received any form of professional help [75].

Regarding risk factors, the GBD research team only found solid causation–effect relationships between childhood sexual abuse, bullying victimization, and intimate partner violence with depressive disorders and bullying victimization with anxiety disorders. The burden of disease caused by these three risk factors has increased since 1990. Women’s mental health status is more likely to be affected by these factors. Approximately 15% of the burden of depression can be attributed to these factors. Furthermore, some studies and reviews have concluded that childhood sexual abuse is deeply linked to depressive disorders; the link is moderated by variables such as victims’ sex, age when abused, type of abuse, the extent of abuse, and the relationship between the victim and the abuser [76,77]. Research has also revealed that female victims with a history of childhood sexual abuse have a higher chance of developing depressive disorders than male victims with a similar history [22,78,79]. Our research found that the harm caused by this situation is worsening. Regarding bullying victimization, research revealed that females, rural children, and left-behind children are more susceptible to depression caused by bullying [80]. This association could be mediated by Internet addiction, sleep quality, and other factors [81,82]. Likewise, bullying has been attributed to anxiety disorders [83]. Female children and adolescents who are a sexual minority [84] and who experience parental separation tend to be more bullying-driven and anxious. In addition, evidence suggests that intimate partner violence is related to higher depression. In women, this association was extremely significant. There is no clear evidence of the same association in men [85]. This relationship could be moderated by childhood maltreatment, socioeconomic deprivation, antisocial personality, and young motherhood.

Finally, we predicted the ASIR for depression and anxiety according to age and country for 2020 to 2044. Similar to geographic variations and age differences in 2019, sub-Saharan Africa, North Africa, and the Middle East would continue to be high-risk regions for depressive disorders, while East Asia, Southeast Asia, and East Europe were estimated as low-risk regions for depressive disorders. South America, East Europe, and the Middle East were predicted as high-risk regions for anxiety disorders, while East Asia, Southeast Asia, Central Asia, and East Europe were projected as low-risk regions for anxiety disorders. Meanwhile, the depressive disease burden on children and older adults will increase based on our prediction, while the working-age population will start to decrease until 2044. Anxiety disorders will continue to increase in all age groups.

There are also some limitations to our study. First, some regions and countries were not estimated precisely because the GBD2019 database uses estimated data. There may be a gap between reported and actual data. Therefore, the analysis and prediction of depression and anxiety disease burden may not be completely accurate. To mitigate these limitations, we utilized the uncertainty intervals provided in the GBD2019 dataset, which accounts for some of the variability and potential errors in the data. These intervals offer a range of possible outcomes, allowing for a more nuanced interpretation of the results. Second, regarding association analysis, this study does not involve causation–effect research. Third, the scope of risk factors analyzed in this study is limited by the data available in the GBD2019 database, which only includes data on three specific risk factors. Although these are significant contributors to mental health disorders, the exclusion of other potential risk factors, such as socioeconomic status, environmental stressors, and access to mental health care, means that our analysis does not fully capture the complexity of the determinants of depression and anxiety. In the future, more specific research should be conducted on risk factors and mental health with a focus on sex, age, and location. Furthermore, our study employs a Bayesian age–period–cohort model to predict future trends in the incidence rates of depression and anxiety from 2020 to 2044. While this model is useful for capturing temporal trends, it is based on assumptions that past trends will continue into the future, which may not account for unforeseen changes such as global events or shifts in public health interventions, such as the COVID-19 pandemic. Additionally, the uncertainty intervals provided in our analysis, while informative, reflect the variability in the data and model assumptions, and they may not account for all sources of uncertainty, particularly in regions with less reliable data. These limitations underscore the need for caution in interpreting our findings. Future research should aim to address these shortcomings by incorporating more comprehensive data sources, improving data collection in underrepresented regions, and exploring alternative analytic approaches that better account for potential biases and uncertainties.

This study offers significant insights into the global trends and disparities in the burden of depressive and anxiety disorders, with critical implications for public health practice, research, and policy. First, our findings suggest the need for more targeted mental health interventions, particularly in regions and among populations identified as high-risk. Policymakers should consider these disparities when designing mental health programs, ensuring that resources are allocated to areas with the greatest need. Furthermore, the rising burden of anxiety disorders calls for urgent attention to preventive strategies and the development of mental health services that are better equipped to address anxiety. In terms of research, this study highlights the importance of investigating the underlying social and economic factors driving the observed trends. Future studies should focus on longitudinal analyses to establish causal relationships and evaluate the impact of various interventions over time. Additionally, there is a need for more comprehensive data collection, particularly in low-resource settings, to improve the accuracy and reliability of global mental health assessments. For policy, this study emphasizes the importance of integrating mental health into broader public health strategies. Given the complex interplay between mental health and other social determinants, policies should be holistic, addressing not only the medical aspects of mental health but also the social, economic, and environmental factors that contribute to these conditions. This approach can help to reduce the global burden of depressive and anxiety disorders and promote mental well-being across diverse populations.

## 5. Conclusions

The disease burden of depression and anxiety is still high compared to what it was in 1990. Depressive disorders have shown a downward trend, but anxiety disorders have shown an upward trend. The disease burden of depression and anxiety in women is much higher than that in men. The highest disease burden for depression was found in older adults, whereas the middle-aged group suffered the most from anxiety. Both high- and low-SDI countries were identified as high-risk regions for depression. Notably, while high-SDI countries had a high disease burden regarding anxiety, low-SDI countries were discovered to be low-risk regions for anxiety. The disease burden attributed to childhood sexual abuse, bullying victimization, and intimate partner violence has increased since 1990. Projections of depression and anxiety displayed geographic and age-related variations.

## Figures and Tables

**Table 1:** ASIR, ASPR, and age-standardized DALY rates and change for depression, 1990–2019 (per 100,000).

Region	ASIR				ASPR				Age-Standardized DALY Rate			
	Rate	Upper	Lower	Change	Rate	Upper	Lower	Change	Rate	Upper	Lower	Change
**Global**	3588.25	4060.42	3152.71	−5.79	3440.05	3817.64	3097.01	−4.22	577.75	788.88	405.79	−4.97
High SDI	4013.63	4550.43	3545.48	0.99	3581.47	3985.85	3230.01	0.50	626.84	852.48	438.47	1.03
High–middle SDI	3184.21	3583.66	2809.60	−11.16	3151.21	3472.33	2851.66	−9.04	523.01	713.05	367.02	−10.84
Middle SDI	3139.00	3540.43	2765.35	−4.79	3194.79	3534.33	2873.67	−3.36	521.68	709.93	366.80	−4.03
Low–middle SDI	4180.30	4740.48	3660.97	−11.82	3839.57	4261.99	3440.78	−7.83	654.34	897.85	458.32	−9.71
Low SDI	4770.22	5461.66	4142.24	−9.79	4283.03	4818.15	3805.88	−6.83	738.87	1011.24	514.68	−7.90
**Central Europe, Eastern Europe, and Central Asia**	3176.93	3618.38	2772.56	9.69	3081.42	3442.33	2747.08	7.15	513.51	701.96	359.29	8.95
Central Asia	3327.42	3825.36	2888.66	−3.57	3186.51	3644.09	2807.90	−2.93	534.90	741.82	372.57	−2.64
Central Europe	2436.80	2771.69	2132.45	−12.87	2601.01	2956.18	2309.75	−8.87	413.89	572.31	290.54	−11.19
Eastern Europe	3546.80	4062.82	3076.08	−9.93	3316.40	3683.91	2964.16	−7.11	562.24	771.76	391.45	−7.99
**High-income**	4179.27	4712.02	3699.39	−6.11	3659.91	4062.62	3307.44	−4.74	647.16	882.46	451.97	−4.97
Australasia	5079.18	5925.80	4368.05	−2.53	4284.28	4908.87	3764.64	−1.32	777.82	1094.07	538.62	−1.84
High-income Asia Pacific	2320.99	2600.80	2063.54	10.04	2084.27	2313.10	1885.57	5.49	365.65	499.26	253.57	7.78
High-income North America	4885.16	5532.44	4308.48	8.77	4270.27	4743.29	3867.94	4.43	753.77	1023.69	525.53	6.67
Southern Latin America	3313.55	3745.45	2925.62	4.72	2777.33	3111.55	2492.50	1.68	503.29	690.90	349.65	3.73
Western Europe	4347.46	4912.74	3841.95	28.37	3851.28	4296.63	3448.09	13.92	677.20	929.50	475.01	20.69
**Latin America and Caribbean**	3983.79	4483.33	3524.74	−8.56	3417.06	3791.43	3079.39	−5.40	607.23	824.73	423.12	−6.72
Andean Latin America	2886.58	3315.19	2499.39	−2.63	2725.64	3105.16	2379.98	−1.97	462.07	640.03	318.12	−1.96
Caribbean	4336.17	5007.13	3737.42	−5.36	3673.61	4178.74	3212.46	−3.71	657.19	905.93	454.07	−4.01
Central Latin America	3675.78	4181.96	3219.65	−7.47	3198.45	3562.30	2865.73	−4.93	563.62	771.17	392.72	−6.00
Tropical Latin America	4560.16	5058.00	4084.43	−0.63	3799.36	4168.93	3464.31	−0.94	686.08	932.46	482.44	−1.00
**North Africa and Middle East**	5098.60	5947.72	4378.86	0.59	4348.89	4971.11	3807.29	0.46	781.06	1075.62	535.18	0.52
**South Asia**	4179.15	4727.18	3668.72	−3.51	3794.72	4199.68	3416.01	−2.10	645.08	877.70	452.66	−3.01
**Southeast Asia, East Asia, and Oceania**	2274.02	2548.25	2016.08	−10.20	2723.91	3022.42	2451.50	−6.96	415.06	569.35	290.21	−8.39
East Asia	2292.26	2562.45	2043.67	−4.25	2720.13	3004.92	2449.91	−2.36	415.98	573.46	291.93	−2.85
Oceania	2711.59	3193.17	2306.26	−9.64	3044.75	3541.71	2622.94	−6.45	476.09	663.22	325.58	−8.23
Southeast Asia	2060.52	2341.00	1797.73	−9.19	2610.58	2958.36	2302.92	−9.16	389.23	536.55	270.38	−9.20
**Sub-Saharan Africa**	5072.06	5771.53	4432.41	1.06	4540.40	5112.41	4038.07	0.71	786.51	1083.35	549.28	0.13
Central Sub-Saharan Africa	6646.94	7819.84	5680.50	−4.74	5536.91	6307.63	4801.31	−3.47	1000.16	1397.69	682.15	−3.81
Eastern Sub-Saharan Africa	5466.48	6234.48	4781.02	−6.43	4849.21	5416.77	4317.19	−4.90	845.40	1154.93	589.89	−5.09
Southern Sub-Saharan Africa	4552.32	5105.97	4015.91	−4.17	4166.26	4612.26	3736.31	−3.10	705.61	958.57	497.87	−3.56
Western Sub-Saharan Africa	4407.30	5021.82	3851.35	−6.04	4075.39	4556.07	3633.02	−4.06	693.84	949.29	485.18	−4.80

**Table 2:** ASIR, ASPR, and age-standardized DALY rates and change for anxiety, 1990–2019 (per 100,000).

Region	ASIR				ASPR				Age-Standardized DALY Rate			
	Rate	Upper	Lower	Change	Rate	Upper	Lower	Change	Rate	Upper	Lower	Change
**Global**	585.45	709.53	474.21	1.04	3779.52	4473.26	3181.07	1.74	360.12	494.44	248.60	2.13
High SDI	710.54	872.80	570.38	3.37	4806.55	5782.42	4017.17	1.10	456.89	626.95	312.75	1.18
High–middle SDI	584.85	704.58	476.96	2.07	3754.47	4406.91	3188.73	2.83	359.98	494.80	250.24	2.66
Middle SDI	599.21	719.35	487.91	−0.94	3793.62	4438.95	3220.87	−1.37	363.15	497.93	252.87	−1.34
Low–middle SDI	549.87	670.04	446.34	−0.30	3470.02	4097.03	2909.21	−0.52	328.89	451.62	228.88	−0.16
Low SDI	556.08	686.33	444.56	−2.87	3494.81	4240.76	2879.71	−3.24	331.48	455.65	226.98	−2.88
**Central Europe, Eastern Europe, and Central Asia**	470.73	576.51	379.52	10.05	2993.33	3562.52	2501.28	11.64	285.83	392.62	197.74	11.98
Central Asia	371.71	464.99	290.94	−0.05	2221.55	2773.54	1751.53	−0.04	212.77	298.07	142.78	0.78
Central Europe	506.31	624.14	403.70	−5.91	3276.15	3986.55	2685.65	−9.55	313.31	435.32	212.34	−9.10
Eastern Europe	504.11	605.85	408.39	−0.53	3188.52	3719.63	2727.09	−0.89	304.66	414.36	214.57	−0.39
**High-income**	735.38	896.97	593.37	0.72	5058.29	6047.44	4242.70	0.59	480.87	657.48	330.35	1.29
Australasia	848.68	1077.15	661.32	1.06	6031.85	7447.53	4885.40	−0.32	575.65	805.72	383.79	−0.12
High-income Asia Pacific	433.60	528.98	351.23	4.57	2616.41	3108.17	2184.38	3.65	253.02	350.79	174.36	3.56
High-income North America	806.33	987.22	643.52	3.98	5559.91	6582.64	4693.53	3.63	521.52	709.05	362.54	3.53
Southern Latin America	730.02	871.88	604.80	−6.76	5125.85	5885.12	4459.77	−6.91	491.15	662.28	343.49	−6.59
Western Europe	791.24	977.02	629.18	8.12	5626.62	6814.10	4632.75	7.71	537.89	748.20	364.59	7.41
**Latin America and Caribbean**	780.33	957.51	621.61	0.61	5502.31	6588.71	4625.85	−0.92	524.13	721.08	361.15	−0.58
Andean Latin America	787.71	1006.92	610.28	10.11	5497.27	6893.06	4467.76	16.07	527.34	741.83	351.37	16.46
Caribbean	669.28	846.20	520.30	2.59	4400.69	5499.82	3522.48	2.72	419.78	587.13	279.15	3.39
Central Latin America	609.11	757.86	484.47	2.14	3930.74	4782.64	3253.45	2.51	376.21	521.35	258.09	2.95
Tropical Latin America	989.45	1208.28	791.17	2.68	7378.64	8605.85	6296.08	1.81	700.17	953.94	487.24	2.01
**North Africa and Middle East**	783.08	980.97	615.84	4.12	5135.71	6267.23	4164.90	3.74	492.15	685.31	333.99	3.81
**South Asia**	497.90	596.56	403.59	2.43	3045.53	3547.16	2594.45	2.55	286.44	390.78	200.71	2.42
**Southeast Asia, East Asia, and Oceania**	539.27	644.14	442.13	1.15	3292.85	3821.73	2801.90	0.74	317.21	434.13	222.90	1.29
East Asia	525.02	620.38	434.05	4.37	3180.75	3663.70	2712.31	4.70	307.61	421.59	215.22	5.21
Oceania	626.04	786.47	488.65	−2.43	4006.76	4990.40	3182.94	−5.56	380.21	531.82	253.59	−5.18
Southeast Asia	578.70	710.45	464.32	1.76	3633.25	4314.97	3024.10	−0.48	347.56	475.99	242.08	−0.48
**Sub-Saharan Africa**	547.81	680.19	436.12	−0.19	3462.63	4184.23	2839.15	0.45	329.74	457.57	224.61	−0.09
Central Sub-Saharan Africa	604.33	768.91	465.78	0.62	3863.99	4826.48	3089.56	0.79	366.32	516.87	244.62	1.26
Eastern Sub-Saharan Africa	575.22	719.92	453.32	14.85	3716.29	4530.63	3050.00	25.06	353.88	487.70	241.11	25.67
Southern Sub-Saharan Africa	576.08	701.31	461.61	0.08	3657.96	4307.84	3100.42	1.50	345.37	472.97	241.16	1.53
Western Sub-Saharan Africa	499.15	612.78	401.76	1.31	3066.50	3683.28	2532.61	1.58	293.35	405.93	201.14	2.02

## Data Availability

All data are accessible through the GBD query tool (http://www.healthdata.org/gbd/2019 (accessed on 15 May 2023)).

## Supplementary Materials

The following supporting information can be downloaded at: https://www.mdpi.com/article/10.3390/healthcare12171721/s1. Supplementary Materials S1. The crude and age-standardized prevalence, incidence, and DALYs rate of depression and anxiety in different counties and regions in 1990 and 2019. Supplementary Materials S2. The crude and age-standardized prevalence, incidence, and DALYs rate of depression and anxiety of different gender from 1990 to 2019. Supplementary Materials S3. The age-standardized DALY rates for depression and anxiety of different country by SDI in 2019. Supplementary Materials S4. The age-standardized DALYs rate of three risk factors of different age groups by gender in 2019. Supplementary Materials S5. The projection of depression and anxiety of all recorded countries of GBD.

## Abbreviations

DALYs	disability-adjusted life years
GHDx	Global Health Data Exchange
WHO	World Health Organization
SDI	socio-demographic index
ASIR	age-standardized incidence rate
ASPR	age-standardized prevalence rate
UIs	uncertainty intervals
APC	age–period–cohort
