# Supplementary material for: Estimation of the Global Disease Burden of Depression and Anxiety between 1990 and 2044: An Analysis of the Global Burden of Disease Study 2019
Source: Healthcare (Basel). 2024 Aug 29;12(17):1721. doi: 10.3390/healthcare12171721 (PMC11395616; doi:10.3390/healthcare12171721)
Supplement: Supplementary file 1 [file healthcare-12-01721-s001.zip › healthcare-3099209-supplementary.pdf]

Supplementary Materials S1 The crude and age-standardized prevalence, incidence, and DALYs rate of depression and anxiety in different counties and regions in 1990 and 2019

Table S1. ASIR, ASPR, and age-standardized DALY rate for depression in 2019 and percentage change of age-standardized rates by region.

Region	ASIR				ASPR				Age-standardized DALY rate			
	Rate	Upper	Lower	change	Rate	Upper	Lower	change	Rate	Upper	Lower	change
**Global**	3588.25	4060.42	3152.71	-5.79	3440.05	3817.64	3097.01	-4.22	577.75	788.88	405.79	-4.97
High SDI	4013.63	4550.43	3545.48	0.99	3581.47	3985.85	3230.01	0.50	626.84	852.48	438.47	1.03
High-middle SDI	3184.21	3583.66	2809.60	-11.16	3151.21	3472.33	2851.66	-9.04	523.01	713.05	367.02	-10.84
Middle SDI	3139.00	3540.43	2765.35	-4.79	3194.79	3534.33	2873.67	-3.36	521.68	709.93	366.80	-4.03
Low-middle SDI	4180.30	4740.48	3660.97	-11.82	3839.57	4261.99	3440.78	-7.83	654.34	897.85	458.32	-9.71
Low SDI	4770.22	5461.66	4142.24	-9.79	4283.03	4818.15	3805.88	-6.83	738.87	1011.24	514.68	-7.90
**Central Europe, Eastern Europe, and Central Asia**	3176.93	3618.38	2772.56	9.69	3081.42	3442.33	2747.08	7.15	513.51	701.96	359.29	8.95
Central Asia	3327.42	3825.36	2888.66	-3.57	3186.51	3644.09	2807.90	-2.93	534.90	741.82	372.57	-2.64
Central Europe	2436.80	2771.69	2132.45	-12.87	2601.01	2956.18	2309.75	-8.87	413.89	572.31	290.54	-11.19
Eastern Europe	3546.80	4062.82	3076.08	-9.93	3316.40	3683.91	2964.16	-7.11	562.24	771.76	391.45	-7.99
**High-income**	4179.27	4712.02	3699.39	-6.11	3659.91	4062.62	3307.44	-4.74	647.16	882.46	451.97	-4.97
Australasia	5079.18	5925.80	4368.05	-2.53	4284.28	4908.87	3764.64	-1.32	777.82	1094.07	538.62	-1.84
High-income Asia Pacific	2320.99	2600.80	2063.54	10.04	2084.27	2313.10	1885.57	5.49	365.65	499.26	253.57	7.78
High-income North America	4885.16	5532.44	4308.48	8.77	4270.27	4743.29	3867.94	4.43	753.77	1023.69	525.53	6.67
Southern Latin America	3313.55	3745.45	2925.62	4.72	2777.33	3111.55	2492.50	1.68	503.29	690.90	349.65	3.73
Western Europe	4347.46	4912.74	3841.95	28.37	3851.28	4296.63	3448.09	13.92	677.20	929.50	475.01	20.69
**Latin America and Caribbean**	3983.79	4483.33	3524.74	-8.56	3417.06	3791.43	3079.39	-5.40	607.23	824.73	423.12	-6.72
Andean Latin America	2886.58	3315.19	2499.39	-2.63	2725.64	3105.16	2379.98	-1.97	462.07	640.03	318.12	-1.96
Caribbean	4336.17	5007.13	3737.42	-5.36	3673.61	4178.74	3212.46	-3.71	657.19	905.93	454.07	-4.01
Central Latin America	3675.78	4181.96	3219.65	-7.47	3198.45	3562.30	2865.73	-4.93	563.62	771.17	392.72	-6.00
Tropical Latin America	4560.16	5058.00	4084.43	-0.63	3799.36	4168.93	3464.31	-0.94	686.08	932.46	482.44	-1.00
**North Africa and Middle East**	5098.60	5947.72	4378.86	0.59	4348.89	4971.11	3807.29	0.46	781.06	1075.62	535.18	0.52
**South Asia**	4179.15	4727.18	3668.72	-3.51	3794.72	4199.68	3416.01	-2.10	645.08	877.70	452.66	-3.01

**Southeast Asia, East Asia, and Oceania**	2274.02	2548.25	2016.08	-10.20	2723.91	3022.42	2451.50	-6.96	415.06	569.35	290.21	-8.39
East Asia	2292.26	2562.45	2043.67	-4.25	2720.13	3004.92	2449.91	-2.36	415.98	573.46	291.93	-2.85
Oceania	2711.59	3193.17	2306.26	-9.64	3044.75	3541.71	2622.94	-6.45	476.09	663.22	325.58	-8.23
Southeast Asia	2060.52	2341.00	1797.73	-9.19	2610.58	2958.36	2302.92	-9.16	389.23	536.55	270.38	-9.20
**Sub-Saharan Africa**	5072.06	5771.53	4432.41	1.06	4540.40	5112.41	4038.07	0.71	786.51	1083.35	549.28	0.13
Central Sub-Saharan Africa	6646.94	7819.84	5680.50	-4.74	5536.91	6307.63	4801.31	-3.47	1000.16	1397.69	682.15	-3.81
Eastern Sub-Saharan Africa	5466.48	6234.48	4781.02	-6.43	4849.21	5416.77	4317.19	-4.90	845.40	1154.93	589.89	-5.09
Southern Sub-Saharan Africa	4552.32	5105.97	4015.91	-4.17	4166.26	4612.26	3736.31	-3.10	705.61	958.57	497.87	-3.56
Western Sub-Saharan Africa	4407.30	5021.82	3851.35	-6.04	4075.39	4556.07	3633.02	-4.06	693.84	949.29	485.18	-4.80

Table S2. ASIR, ASPR, and age-standardized DALYs rate for anxiety in 2019 and percentage change of age-standardized rates by region.

Region	ASIR				ASPR				Age-standardized DALY rate			
	Rate	Upper	Lower	change	Rate	Upper	Lower	change	Rate	Upper	Lower	change
**Global**	585.45	709.53	474.21	1.04	3779.52	4473.26	3181.07	1.74	360.12	494.44	248.60	2.13
High SDI	710.54	872.80	570.38	3.37	4806.55	5782.42	4017.17	1.10	456.89	626.95	312.75	1.18
High-middle SDI	584.85	704.58	476.96	2.07	3754.47	4406.91	3188.73	2.83	359.98	494.80	250.24	2.66
Middle SDI	599.21	719.35	487.91	-0.94	3793.62	4438.95	3220.87	-1.37	363.15	497.93	252.87	-1.34
Low-middle SDI	549.87	670.04	446.34	-0.30	3470.02	4097.03	2909.21	-0.52	328.89	451.62	228.88	-0.16
Low SDI	556.08	686.33	444.56	-2.87	3494.81	4240.76	2879.71	-3.24	331.48	455.65	226.98	-2.88
**Central Europe, Eastern Europe, and Central Asia**	470.73	576.51	379.52	10.05	2993.33	3562.52	2501.28	11.64	285.83	392.62	197.74	11.98
Central Asia	371.71	464.99	290.94	-0.05	2221.55	2773.54	1751.53	-0.04	212.77	298.07	142.78	0.78
Central Europe	506.31	624.14	403.70	-5.91	3276.15	3986.55	2685.65	-9.55	313.31	435.32	212.34	-9.10
Eastern Europe	504.11	605.85	408.39	-0.53	3188.52	3719.63	2727.09	-0.89	304.66	414.36	214.57	-0.39
**High-income**	735.38	896.97	593.37	0.72	5058.29	6047.44	4242.70	0.59	480.87	657.48	330.35	1.29
Australasia	848.68	1077.15	661.32	1.06	6031.85	7447.53	4885.40	-0.32	575.65	805.72	383.79	-0.12
High-income Asia Pacific	433.60	528.98	351.23	4.57	2616.41	3108.17	2184.38	3.65	253.02	350.79	174.36	3.56
High-income North America	806.33	987.22	643.52	3.98	5559.91	6582.64	4693.53	3.63	521.52	709.05	362.54	3.53

Southern Latin America	730.02	871.88	604.80	-6.76	5125.85	5885.12	4459.77	-6.91	491.15	662.28	343.49	-6.59
Western Europe	791.24	977.02	629.18	8.12	5626.62	6814.10	4632.75	7.71	537.89	748.20	364.59	7.41
**Latin America and Caribbean**	780.33	957.51	621.61	0.61	5502.31	6588.71	4625.85	-0.92	524.13	721.08	361.15	-0.58
Andean Latin America	787.71	1006.92	610.28	10.11	5497.27	6893.06	4467.76	16.07	527.34	741.83	351.37	16.46
Caribbean	669.28	846.20	520.30	2.59	4400.69	5499.82	3522.48	2.72	419.78	587.13	279.15	3.39
Central Latin America	609.11	757.86	484.47	2.14	3930.74	4782.64	3253.45	2.51	376.21	521.35	258.09	2.95
Tropical Latin America	989.45	1208.28	791.17	2.68	7378.64	8605.85	6296.08	1.81	700.17	953.94	487.24	2.01
**North Africa and Middle East**	783.08	980.97	615.84	4.12	5135.71	6267.23	4164.90	3.74	492.15	685.31	333.99	3.81
**South Asia**	497.90	596.56	403.59	2.43	3045.53	3547.16	2594.45	2.55	286.44	390.78	200.71	2.42
**Southeast Asia, East Asia, and Oceania**	539.27	644.14	442.13	1.15	3292.85	3821.73	2801.90	0.74	317.21	434.13	222.90	1.29
East Asia	525.02	620.38	434.05	4.37	3180.75	3663.70	2712.31	4.70	307.61	421.59	215.22	5.21
Oceania	626.04	786.47	488.65	-2.43	4006.76	4990.40	3182.94	-5.56	380.21	531.82	253.59	-5.18
Southeast Asia	578.70	710.45	464.32	1.76	3633.25	4314.97	3024.10	-0.48	347.56	475.99	242.08	-0.48
**Sub-Saharan Africa**	547.81	680.19	436.12	-0.19	3462.63	4184.23	2839.15	0.45	329.74	457.57	224.61	-0.09
Central Sub-Saharan Africa	604.33	768.91	465.78	0.62	3863.99	4826.48	3089.56	0.79	366.32	516.87	244.62	1.26
Eastern Sub-Saharan Africa	575.22	719.92	453.32	14.85	3716.29	4530.63	3050.00	25.06	353.88	487.70	241.11	25.67
Southern Sub-Saharan Africa	576.08	701.31	461.61	0.08	3657.96	4307.84	3100.42	1.50	345.37	472.97	241.16	1.53
Western Sub-Saharan Africa	499.15	612.78	401.76	1.31	3066.50	3683.28	2532.61	1.58	293.35	405.93	201.14	2.02

Table S3 The crude and age-standardized prevalence, incidence, and DALYs rate of depression in different counties and regions in 2019

region	Prevalence rate		DALY rate		Incidence rate	
	Crude	AS	Crude	AS	Crude	AS
Global	3613.667944	3440.052654	605.6718108	577.7469649	3750.398299	3588.24898
Central Europe, Eastern Europe, and Central Asia	3650.773624	3081.424926	604.6402284	513.510682	3755.742681	3176.926324
Central Asia	3105.245018	3186.512986	520.2559022	534.9000277	3187.153437	3327.423102
Armenia	3491.915393	2983.651594	574.9864351	492.7479055	3538.805147	3025.044685
Azerbaijan	2966.673199	2752.631401	476.9879557	445.1082383	2813.094294	2676.214794
Georgia	4072.843574	3323.312992	687.5849763	561.3585932	4398.857835	3533.482773
Kazakhstan	3493.707763	3441.205529	593.482215	586.6018031	3711.289278	3709.138077
Kyrgyzstan	3102.794834	3381.698539	530.0152979	577.7669659	3278.963496	3621.31047
Mongolia	3827.71634	3963.209397	675.1794273	700.2730927	4256.815768	4488.331145
Tajikistan	2433.620033	2817.280365	396.4458734	459.0752932	2349.287704	2778.858809
Turkmenistan	2913.236711	3049.741466	484.7111081	508.8932833	2936.073721	3129.08934
Uzbekistan	2941.513174	3164.448257	493.1395358	530.9521544	3000.333569	3293.660167
Central Europe	3300.781179	2601.012539	522.1686054	413.8898055	3114.131462	2436.798367
Albania	2917.385479	2460.203054	456.7974229	386.4192442	2641.996141	2221.470561
Bosnia and Herzegovina	3529.13546	2762.449719	565.7397242	445.3090162	3441.60546	2674.071597
Bulgaria	3588.896301	2691.660762	575.4563202	431.7863701	3518.161412	2575.207916
Croatia	3970.34322	3007.607735	651.8658964	495.5906651	4101.411488	3043.215742
Czechia	3666.454231	2878.851785	591.9581225	469.2108875	3659.745296	2853.514514
Hungary	3724.929016	2823.370375	603.4188768	458.0626278	3725.861768	2770.173558
Montenegro	3415.897647	2817.865742	553.64055	458.3941972	3362.392566	2761.024254
North Macedonia	3152.284345	2570.90609	495.8729504	406.8020874	2918.942181	2394.977554
Poland	2836.156251	2277.957345	429.8960962	349.1334543	2407.850341	1954.477095
Romania	3368.023209	2650.162106	536.8498985	424.4387533	3221.480714	2506.509783

Serbia	3532.759351	2814.907106	572.2382172	456.6331808	3522.057741	2758.206313
Slovakia	3408.379288	2695.262018	544.4018468	432.4310314	3269.572863	2577.156067
Slovenia	4096.246798	3115.223088	679.8101535	519.8060288	4278.924128	3205.197194
Eastern Europe	4084.172811	3316.402695	687.0933551	562.2431068	4358.054155	3546.796642
Belarus	4804.327393	3824.538982	835.2087	668.0857867	5427.006155	4307.32835
Estonia	4854.26433	3693.642591	840.0427135	639.9750164	5515.467941	4113.959241
Latvia	4929.632626	3747.82854	852.5669351	650.9173356	5599.118477	4188.665672
Lithuania	5201.262422	3991.194749	909.6780843	702.4349785	5991.951762	4551.804893
Republic of Moldova	4078.213433	3220.740104	683.3084627	540.8286163	4321.578495	3397.982788
Russian Federation	3717.232005	3079.915744	612.9987333	513.0766988	3812.789517	3183.179904
Ukraine	5021.115162	3897.303428	876.4133626	683.4210548	5754.822012	4449.257614
High-income	4119.717765	3659.909604	713.7135735	647.1613303	4600.445212	4179.272143
Australasia	4579.673925	4284.282011	817.3884675	777.8239842	5298.229467	5079.181708
Australia	4722.78768	4382.457817	847.0861567	798.717266	5514.826091	5234.479524
New Zealand	3797.579767	3723.863871	655.0952676	658.1421701	4114.562988	4187.429126
High-income Asia Pacific	2532.713707	2084.27223	433.6854091	365.6517289	2773.035146	2320.9928
Brunei Darussalam	1711.261324	1603.124123	277.9643164	260.2862311	1641.858884	1575.579992
Japan	2548.709038	2103.280393	439.1513779	373.8521977	2836.086326	2391.475544
Republic of Korea	2524.204963	2091.18118	425.1813849	357.6355519	2657.507405	2241.213426
Singapore	2315.580162	2008.131481	402.5746267	355.2164518	2527.109345	2264.446066
High-income North America	4546.832233	4270.266838	785.6278931	753.7696919	5063.596632	4885.164958
Canada	3509.761402	3290.465741	577.3978429	556.0067841	3440.005004	3371.668142
Greenland	6187.258953	5835.711773	1152.301794	1098.691711	7483.064636	7225.500015
United States of America	4662.027167	4375.99802	808.7511163	774.9743791	5243.966136	5047.932696
Southern Latin America	2988.823991	2777.333074	538.6578466	503.2936381	3538.628773	3313.547851
Argentina	2553.885337	2407.509792	453.719591	429.6158893	2959.594371	2807.758892
Chile	4036.902252	3643.512404	745.3602539	677.5855771	4949.260814	4516.868898
Uruguay	3148.61873	2840.908153	559.1369311	508.7870055	3670.204829	3330.820627

Western Europe	4586.465563	3851.283655	793.7077091	677.195169	5113.868762	4347.46126
Andorra	4434.062177	3607.207159	759.2485182	626.0285968	4783.308253	3963.785135
Austria	3751.763911	3125.200991	617.6198637	523.2630936	3825.537452	3240.0709
Belgium	4111.33349	3542.022099	707.1739973	619.5704672	4553.991244	3983.009179
Cyprus	3611.446615	3112.702207	596.2566035	521.4466375	3634.837521	3214.222535
Denmark	4146.113076	3482.89949	703.1514948	597.0995279	4464.242747	3774.606183
Finland	4730.676646	4139.295159	805.4356597	722.9019871	5073.143785	4569.012271
France	4456.68545	3835.741073	772.558735	673.525754	4957.05751	4302.017432
Germany	4113.351718	3376.794323	696.9751091	583.5696847	4465.906525	3723.997153
Greece	6222.767934	5042.25068	1128.049253	927.7547671	7466.355424	6104.394449
Iceland	3298.728414	2936.593769	538.966539	485.7566305	3270.911352	2955.212354
Ireland	4645.655482	4216.930921	815.966	749.2042872	5242.457796	4822.711932
Israel	4206.697605	4127.254739	745.7259833	734.1882163	4814.307443	4724.385845
Italy	4455.240253	3561.657813	762.2682592	620.3781957	4911.377155	3957.016224
Luxembourg	3790.059599	3219.307459	629.7893873	542.150451	3908.761067	3377.574509
Malta	3739.210607	3131.181427	614.6356605	525.5109729	3792.74643	3246.273276
Monaco	4967.084386	4056.450078	862.7128975	719.1516592	5589.58063	4633.007273
Netherlands	4158.753147	3534.401445	715.0586662	616.8133721	4547.344825	3916.897163
Norway	3655.031581	3238.202342	611.0805488	552.0508661	3753.913744	3416.350718
Portugal	5618.012751	4394.06635	995.0115048	787.151771	6569.431106	5142.334992
San Marino	4809.984196	4091.882758	843.0350222	726.7097578	5442.755959	4681.845887
Spain	5714.388504	4704.454619	1021.476834	854.1966286	6688.608441	5570.034386
Sweden	4716.598275	4116.486019	819.9646765	728.2468165	5238.494054	4652.213282
Switzerland	4456.380338	3705.446686	753.182227	635.3109417	4710.246911	3978.533773
United Kingdom	4727.080397	4097.567498	819.3436945	721.2923785	5300.116522	4669.115703
Latin America and Caribbean	3580.978811	3417.055126	636.085354	607.2314995	4159.453166	3983.785385
Andean Latin America	2691.091978	2725.636669	457.0621179	462.071416	2845.7999	2886.578282
Bolivia (Plurinational State of)	3100.054121	3399.432132	549.108642	600.0662215	3544.437115	3892.568395

Ecuador	3275.330499	3330.697213	581.0563213	588.5182907	3728.073427	3784.30827
Peru	2244.3207	2211.669424	360.3873106	355.2181077	2142.480333	2120.161713
Caribbean	3880.899334	3673.611561	693.3339441	657.1881941	4571.126902	4336.170986
Antigua and Barbuda	3453.131361	3028.679581	595.2119213	524.0586733	3799.527363	3363.156896
Bahamas	3391.43583	3062.336325	587.347492	531.3212993	3753.827908	3412.994779
Barbados	3835.116966	3178.277641	665.4497938	556.7190794	4304.195487	3589.01282
Belize	3165.836267	3309.06497	563.319568	583.7451609	3647.779372	3787.3403
Bermuda	4057.058769	3177.547899	702.9502939	558.2398121	4554.127904	3592.119797
Cuba	4786.079995	3802.294221	856.8286801	684.9163685	5711.266561	4535.02869
Dominica	3440.504838	3084.768717	594.5859363	535.4501054	3842.313914	3452.558026
Dominican Republic	3770.366746	3823.102905	682.8998009	691.1017282	4489.381574	4562.939682
Grenada	3545.284237	3234.760068	621.0306281	567.0778223	4011.687106	3677.831101
Guyana	4978.331634	4927.69208	930.7860312	915.442371	6286.965629	6206.878165
Haiti	3246.67175	3635.117319	581.4920552	644.3858522	3838.012279	4271.103864
Jamaica	3313.131529	3064.670549	575.1995257	532.1287898	3686.839075	3420.267077
Puerto Rico	3555.108045	2882.805692	600.6667659	493.6627228	3863.478887	3141.814572
Saint Kitts and Nevis	4476.65961	3926.992275	809.1031247	711.2915041	5345.996604	4723.08253
Saint Lucia	3823.833455	3316.274391	668.0619705	582.3665439	4340.652934	3797.128689
Saint Vincent and the Grenadines	3704.083327	3367.614194	651.4392594	594.3807782	4243.609249	3874.136074
Suriname	4909.758672	4688.168316	909.7997377	869.9253738	6110.412436	5850.971726
Trinidad and Tobago	4356.553757	3826.370141	780.1148628	691.4383985	5131.721619	4560.355856
United States Virgin Islands	3969.896608	3356.748445	693.3872048	593.1120517	4540.091838	3853.114895
Central Latin America	3279.02055	3198.453356	578.8252009	563.6177864	3764.785252	3675.775141
Colombia	2389.172184	2219.627819	394.1260629	366.7472832	2430.785677	2265.124249
Costa Rica	3501.705565	3213.285655	618.8461471	568.7636506	4003.868606	3692.098596
El Salvador	3424.131017	3407.369046	612.4741751	607.1445479	4011.208362	3975.086762
Guatemala	3232.854103	3667.931078	585.2948533	658.2619058	3862.402093	4369.610962

Honduras	2722.642966	3155.114096	479.6103959	552.3764494	3114.404029	3616.264642
Mexico	3649.923021	3535.829652	652.2574334	630.5776321	4288.708507	4159.227213
Nicaragua	3074.448703	3254.86528	549.7225531	576.9308877	3558.373429	3755.142282
Panama	2973.218189	2936.914213	517.4519872	510.9937095	3330.570113	3290.393967
Venezuela (Bolivarian Republic of)	3389.480621	3214.794378	598.5597661	568.5660978	3886.051362	3701.341427
Tropical Latin America	4108.459691	3799.355424	738.9544607	686.080748	4887.55503	4560.162235
Brazil	4122.286598	3801.556341	741.3294659	686.5581873	4904.77968	4564.972055
Paraguay	3676.193086	3694.57673	664.7053609	663.8951839	4349.065767	4353.14385
North Africa and Middle East	4334.009695	4348.89165	783.2540839	781.0614669	5093.80641	5098.604936
Afghanistan	3749.035847	4945.1681	691.5728913	890.1888115	4670.689135	5985.107604
Algeria	4175.155397	4129.680812	746.2306596	736.5148725	4797.057689	4763.394221
Bahrain	5367.20745	4528.088795	977.2125519	823.251147	6306.3703	5415.292306
Egypt	3673.017125	3939.757741	654.7467474	696.6078076	4210.112626	4487.849603
Iran (Islamic Republic of)	5260.144526	4860.691959	964.9603896	890.3244831	6342.666459	5895.484506
Iraq	3675.96812	3932.648077	655.2305849	690.8721769	4238.517728	4465.112992
Jordan	3929.575449	4152.110145	711.6732004	743.5319997	4609.398715	4819.98442
Kuwait	4652.342599	4060.300389	830.8959761	723.9469881	5277.849467	4677.721402
Lebanon	4780.800761	4652.712102	863.6859354	842.0056893	5656.316575	5536.835381
Libya	4832.5778	4406.426761	872.2681423	791.4713568	5668.060491	5183.185339
Morocco	5256.908716	5078.539509	970.1688708	934.5825146	6405.751625	6189.883961
Oman	4165.484256	3962.997694	751.3886724	707.795995	4756.856301	4584.85939
Palestine	5368.670378	6198.952064	1027.137733	1168.681168	6918.705462	7864.198801
Qatar	4698.36213	3951.378419	853.023326	708.3772241	5408.152525	4616.484058
Saudi Arabia	4719.362155	4216.06946	845.78928	748.7056294	5384.934775	4848.404082
Sudan	3793.921055	4423.096847	696.3946266	797.85691	4566.750502	5201.329493
Syrian Arab Republic	4167.909564	4132.855115	742.872036	731.2494056	4843.196748	4747.267959
Tunisia	5486.138328	5049.405416	1007.285983	930.1461119	6616.366102	6138.574144

Turkey	4416.501804	3942.268049	777.5895361	696.2926862	4958.367684	4471.830716
United Arab Emirates	4504.054495	3578.490406	794.3127667	628.6290613	4972.444262	4031.524457
Yemen	4041.551016	4895.367641	747.3801489	890.9881965	4995.191393	5910.915545
South Asia	3631.499774	3794.717238	619.7891018	645.0848813	3988.388632	4179.152603
Bangladesh	4533.470456	4619.809404	809.6140478	822.1980964	5321.933807	5423.437113
Bhutan	3930.584297	4024.815079	681.9360533	696.861613	4399.062494	4537.950335
India	3599.563432	3659.838915	608.0415822	616.1300058	3895.507541	3975.680517
Nepal	4471.468351	4897.598325	800.4312763	874.0445333	5316.452103	5836.384344
Pakistan	3073.584828	3942.525712	533.047855	678.2659636	3435.354237	4399.135799
Southeast Asia, East Asia, and Oceania	3265.910759	2723.907237	491.4806531	415.0617756	2640.508094	2274.017305
East Asia	3511.789854	2720.13208	529.9915791	415.982921	2868.89176	2292.263074
China	3519.2619	2724.015591	531.6541792	416.948722	2882.923902	2301.414715
Democratic People's Republic of Korea	3169.355025	2676.924669	469.9785095	401.1114946	2450.832536	2129.828436
Taiwan (Province of China)	3442.153705	2564.064964	496.525138	378.7589246	2488.212644	1957.196072
Oceania	2647.480434	3044.753401	426.1480555	476.0881891	2474.346766	2711.592675
American Samoa	2581.804156	2659.042019	386.4232253	393.942046	2103.943158	2123.051481
Cook Islands	3476.708312	3160.885597	538.8626266	500.3680786	3040.740984	2863.320244
Fiji	2893.007571	2917.754794	446.6434742	448.3900166	2493.03045	2513.683145
Guam	3318.212376	3197.231897	529.1184632	513.9477171	2998.663042	2936.518171
Kiribati	2624.27411	2970.307965	414.6165632	457.4796125	2362.82621	2563.806488
Marshall Islands	2680.241791	2887.396396	419.3738395	442.7343429	2366.581391	2474.627344
Micronesia (Federated States of)	2756.25462	2909.946945	432.84796	448.3578182	2442.683534	2498.092594
Nauru	2651.223	3149.646795	435.2417172	497.9471845	2548.159746	2845.861438
Niue	3410.461166	3140.699924	527.9506335	495.4394883	2994.517972	2843.555516
Northern Mariana Islands	3325.740166	2780.080821	496.8094387	424.8915124	2636.93075	2323.866868
Palau	3665.455811	3089.023309	565.426069	488.6608091	3141.371469	2805.812323

Papua New Guinea	2627.153042	3072.68611	425.8902039	481.8961943	2490.647111	2756.741896
Samoa	2490.215958	2802.756148	389.4386862	428.0298547	2192.905759	2350.395729
Solomon Islands	2463.291968	3003.944776	396.9824539	468.5739444	2301.887398	2646.197739
Tokelau	2936.602964	3122.21342	464.86459	494.8562409	2655.173612	2825.452298
Tonga	2399.999525	2694.796798	366.4171355	404.0946377	2010.383969	2169.171034
Tuvalu	3066.932514	3125.087328	490.7260718	494.9348984	2827.156829	2830.077282
Vanuatu	2533.399716	2974.423444	405.591676	462.5570114	2340.908884	2604.305226
Southeast Asia	2740.854415	2610.579084	408.6221971	389.2293919	2144.767326	2060.515251
Cambodia	2801.161407	2950.099406	440.1715551	456.3553528	2457.142629	2529.956314
Indonesia	2537.768585	2418.471208	368.587479	350.2641861	1874.018965	1794.073089
Lao People's Democratic Republic	2555.427634	2746.891166	398.7580885	417.5355019	2186.773518	2244.922338
Malaysia	3783.49353	3663.094613	634.9630858	611.6648046	3736.761647	3619.101811
Maldives	3029.677452	2912.272417	480.4647833	459.4513144	2645.596561	2572.326078
Mauritania	2839.076589	3655.844701	474.1028124	609.0790529	2904.048447	3760.989418
Myanmar	2210.772562	2193.303578	302.1845476	298.1868621	1413.191736	1393.922528
Philippines	2571.931107	2779.254087	398.7812768	424.6935795	2217.49751	2344.825581
Seychelles	2984.324629	2644.614894	441.8808666	396.9892911	2302.484055	2119.776152
Sri Lanka	3125.604843	2866.229678	473.8938526	440.8700874	2549.512904	2412.359035
Thailand	3586.159648	2847.038071	540.7317258	438.0735981	2881.0901	2393.681009
Timor-Leste	2319.032781	2804.641739	366.3651482	427.7052318	2077.323207	2341.126342
Viet Nam	2728.049187	2473.061002	394.8896892	360.2321977	1971.523707	1832.794054
Sub-Saharan Africa	3368.1534	4540.39874	585.7596798	786.5107233	3737.846847	5072.056501
Central Sub-Saharan Africa	4199.208004	5536.907687	768.003916	1000.155227	5104.231616	6646.944067
Angola	4140.433495	5744.193769	764.2565695	1046.882404	5078.454737	6951.372919
Central African Republic	4598.359375	5923.846525	848.3577494	1078.692093	5685.568515	7230.553777
Congo	4686.85347	5525.740662	854.3890724	999.4770392	5639.968992	6634.84071

Democratic Republic of the Congo	4144.91423	5443.060181	755.3237571	979.4431092	5020.176396	6507.685065
Equatorial Guinea	4495.248375	5728.945341	831.9637013	1041.594073	5543.559047	6941.005919
Gabon	5015.014699	5545.971179	912.5989243	1001.940191	6030.008387	6654.440945
Eastern Sub-Saharan Africa	3494.347358	4849.21069	609.5930286	845.399692	3888.764447	5466.483312
Burundi	3298.279099	4705.152669	568.381045	811.3560261	3624.018112	5240.031057
Comoros	3817.591878	4263.159554	647.8616428	724.8236481	4033.55888	4565.68149
Djibouti	3824.045119	4461.265719	656.7965897	769.6965465	4074.569739	4888.469366
Eritrea	3696.064155	4841.218263	641.8800535	840.2726417	4075.356505	5439.120496
Ethiopia	3312.816656	4649.720438	576.1328435	808.7015027	3652.843421	5200.846929
Kenya	3672.87202	4723.163015	637.6295157	820.1963489	4053.421686	5307.040333
Madagascar	3566.922593	4737.186817	619.8126217	823.2692924	3913.163667	5283.040013
Malawi	2975.580122	4100.135766	497.5779543	684.9062913	3083.89424	4303.016436
Mozambique	3372.829495	4992.67129	586.032616	865.3615931	3777.589791	5645.458036
Rwanda	4035.758879	5097.020729	708.6554183	894.7885148	4542.387266	5818.521191
Somalia	3214.080857	4846.649926	558.6704629	842.2483809	3567.933472	5453.692461
South Sudan	3374.058224	4749.942128	580.4299417	812.5912455	3747.645119	5291.401033
Uganda	4365.939808	6584.519721	806.1057856	1212.094495	5321.510782	8062.759321
United Republic of Tanzania	3388.785047	4651.241606	584.4898783	802.2688011	3700.641885	5139.838283
Zambia	3068.915403	4219.489283	514.4816488	708.9351169	3201.920794	4496.997953
Southern Sub-Saharan Africa	3936.568322	4166.256515	667.6485289	705.6059473	4255.831963	4552.320458
Botswana	3962.162462	4257.260381	673.0897026	721.8573946	4262.121036	4655.865002
Eswatini	3696.721224	4425.529629	633.1584364	752.9644999	4068.11734	4900.231539
Lesotho	4938.235913	5511.564101	882.224551	979.2817465	5834.577394	6548.414831
Namibia	3126.871439	3616.461543	514.5845817	592.7308054	3158.739539	3683.193079
South Africa	4300.784575	4298.678046	734.8713635	733.9973146	4722.715633	4769.633321
Zimbabwe	2592.113813	3395.47547	415.0900201	544.5315987	2495.81985	3351.167513
Western Sub-Saharan Africa	2916.823395	4075.394169	497.6147079	693.8359816	3118.566465	4407.298763

Benin	3062.895441	4447.893004	533.9170782	772.8768609	3388.475163	4959.756163
Burkina Faso	3013.891939	4378.302439	522.6419489	757.6519502	3304.161442	4845.580197
Cabo Verde	4676.967134	4842.931771	832.5859044	861.7239828	5321.538987	5567.45647
Cameroon	3412.489834	4604.639835	597.5859928	803.6870381	3814.686212	5204.112061
Chad	3193.276566	5133.411783	573.1650814	915.2911081	3741.928974	6002.416912
C??te d'Ivoire	2974.204824	3935.077603	502.1001722	664.3284312	3111.122535	4203.618024
Gambia	4052.912865	5501.878916	734.277916	994.1931566	4820.100951	6582.284862
Ghana	3607.585425	4362.028427	624.351585	754.257997	3930.565356	4824.332914
Guinea	3084.693173	4373.116618	535.469731	756.1246555	3405.065299	4844.878016
Guinea-Bissau	3288.889084	4494.280441	572.4116365	780.0024958	3632.271861	5025.955731
Liberia	3560.269489	4556.289145	616.7086828	786.622444	3976.481304	5147.98341
Mali	2398.62373	3541.384191	397.2883676	583.2501596	2431.031894	3593.085951
Mauritius	4514.765837	3804.76283	741.265628	636.8143035	4357.852002	3813.885612
Niger	2581.800021	4235.214954	447.7192287	730.0041983	2834.303621	4641.347909
Nigeria	2699.887809	3817.397677	452.6770271	639.5515639	2805.136716	4025.567692
Sao Tome and Principe	3129.080824	3709.464359	526.7466626	620.8862873	3230.870437	3850.262365
Senegal	3075.213633	4029.399835	522.407033	683.3914469	3271.675092	4330.552008
Sierra Leone	3325.865454	4392.156899	577.6566577	760.3615284	3665.776061	4886.116437
Togo	3484.32317	4500.815615	606.5876823	783.3257567	3840.141252	5034.254256

Table S4 The crude and age-standardized prevalence, incidence, and DALYs rate of anxiety in different counties and regions in 2019

Region	Prevalence rate		DALY rate		incidence rate	
	Crude	AS	Crude	AS	Crude	AS
Global	3895.208644	3779.522033	3895.208644	3779.522033	592.1976745	585.4523962
Central Europe, Eastern Europe, and Central Asia	3261.456616	2993.326957	3261.456616	2993.326957	474.0138537	470.7318055
Central Asia	2207.032253	2221.552329	2207.032253	2221.552329	377.199292	371.7148009
Armenia	3397.552135	3150.597768	3397.552135	3150.597768	485.3884667	479.8834345
Azerbaijan	2608.031286	2477.538333	2608.031286	2477.538333	422.7636914	405.6585127
Georgia	2683.643323	2495.047473	2683.643323	2495.047473	403.9153659	406.7469674
Kazakhstan	2122.363729	2115.630296	2122.363729	2115.630296	362.932632	360.1411344
Kyrgyzstan	1991.854317	2059.919609	1991.854317	2059.919609	353.5315103	352.6868614
Mongolia	2091.907576	2142.922888	2091.907576	2142.922888	365.7722328	364.0451747
Tajikistan	2277.579707	2403.539396	2277.579707	2403.539396	399.6421415	398.1139256
Turkmenistan	2302.950967	2341.513394	2302.950967	2341.513394	392.4110409	388.9298332
Uzbekistan	1991.24467	2025.65463	1991.24467	2025.65463	355.5964369	348.5267154
Central Europe	3704.83931	3276.14563	3704.83931	3276.14563	503.0416925	506.3097888
Albania	4258.95208	3837.537019	4258.95208	3837.537019	562.2528947	562.2435231
Bosnia and Herzegovina	4042.133821	3537.194928	4042.133821	3537.194928	533.5016029	531.6914234
Bulgaria	3898.441489	3397.369724	3898.441489	3397.369724	503.7193162	515.2716263
Croatia	4117.393554	3595.011355	4117.393554	3595.011355	525.6366041	536.9908947
Czechia	3539.398289	3153.907323	3539.398289	3153.907323	481.2203356	491.5905329
Hungary	3898.373592	3406.722985	3898.373592	3406.722985	508.5069393	516.8926862
Montenegro	4071.71464	3658.64768	4071.71464	3658.64768	547.0656636	543.4276891
North Macedonia	4023.540695	3591.532249	4023.540695	3591.532249	545.8152291	536.7892185
Poland	3489.300662	3082.49674	3489.300662	3082.49674	494.0927016	490.6938045
Romania	3710.606341	3285.616934	3710.606341	3285.616934	498.6645577	505.7681559

Serbia	3742.156535	3335.390253	3742.156535	3335.390253	507.4714017	510.8863069
Slovakia	3857.823952	3425.665576	3857.823952	3425.665576	519.1961885	519.0256631
Slovenia	3837.091308	3389.172798	3837.091308	3389.172798	502.7000628	516.6766306
Eastern Europe	3489.947457	3188.518301	3489.947457	3188.518301	501.3485194	504.113466
Belarus	3808.629114	3483.199112	3808.629114	3483.199112	519.4958788	529.7849486
Estonia	3725.45987	3378.816187	3725.45987	3378.816187	496.8548675	518.8833049
Latvia	4168.194521	3748.034178	4168.194521	3748.034178	527.6948488	556.4467221
Lithuania	4775.859237	4254.187588	4775.859237	4254.187588	575.7834312	606.7859377
Republic of Moldova	4550.180406	4078.754789	4550.180406	4078.754789	587.1334699	588.95485
Russian Federation	3407.512038	3133.695707	3407.512038	3133.695707	497.18403	499.313387
Ukraine	3488.934853	3133.127631	3488.934853	3133.127631	498.3887912	499.0760496
High-income	5400.645343	5058.286252	5400.645343	5058.286252	682.9105808	735.3836891
Australasia	6201.254937	6031.850732	6201.254937	6031.850732	788.1478667	848.6768888
Australia	5986.321535	5815.164611	5986.321535	5815.164611	768.2706185	825.9644136
New Zealand	7375.832169	7263.469444	7375.832169	7263.469444	896.7739011	974.9916605
High-income Asia Pacific	2752.51089	2616.41188	2752.51089	2616.41188	395.3924175	433.6045866
Brunei Darussalam	3035.588861	2856.927928	3035.588861	2856.927928	489.3898855	468.9651325
Japan	2438.500632	2324.125489	2438.500632	2324.125489	355.8923018	396.2354371
Republic of Korea	3487.415314	3221.324194	3487.415314	3221.324194	484.2669305	507.9303044
Singapore	2886.702029	2708.086159	2886.702029	2708.086159	441.4140439	452.5765217
High-income North America	6022.237453	5559.908264	6022.237453	5559.908264	785.2842919	806.3293419
Canada	4683.029634	4320.606081	4683.029634	4320.606081	633.216862	659.9051356
Greenland	5242.617648	4856.122436	5242.617648	4856.122436	723.9037444	717.4666943
United States of America	6171.489391	5697.747631	6171.489391	5697.747631	802.2272416	822.0716145
Southern Latin America	5350.565771	5125.848292	5350.565771	5125.848292	722.6245709	730.0155698
Argentina	5162.388885	4982.973251	5162.388885	4982.973251	724.0351472	727.4356775
Chile	5812.082033	5461.949327	5812.082033	5461.949327	724.8418908	738.6538994
Uruguay	5376.995195	5153.944653	5376.995195	5153.944653	692.3608937	724.2872656

Western Europe	5972.349268	5626.623055	5972.349268	5626.623055	707.7063765	791.2436856
Andorra	5860.766431	5453.409949	5860.766431	5453.409949	729.539683	791.3220982
Austria	6277.715001	6006.434302	6277.715001	6006.434302	736.9310322	843.3329699
Belgium	5251.203925	5047.239889	5251.203925	5047.239889	683.0238012	756.7104695
Cyprus	6847.34939	6467.176335	6847.34939	6467.176335	815.1658428	881.0676055
Denmark	5103.450434	4873.904312	5103.450434	4873.904312	660.079106	730.564345
Finland	4143.321038	3993.12122	4143.321038	3993.12122	573.7520303	641.4763973
France	6576.43736	6274.255491	6576.43736	6274.255491	762.0617514	850.9869877
Germany	6739.136345	6208.144991	6739.136345	6208.144991	736.733766	846.0520594
Greece	6098.624172	5779.041611	6098.624172	5779.041611	708.543802	816.8022952
Iceland	5304.770735	5103.768277	5304.770735	5103.768277	709.0426322	754.8681294
Ireland	7091.965786	6907.819414	7091.965786	6907.819414	861.8557411	918.3343755
Israel	3712.262862	3739.261475	3712.262862	3739.261475	588.5230166	605.3156106
Italy	5935.74744	5562.460292	5935.74744	5562.460292	705.8988745	806.3234335
Luxembourg	5558.410698	5277.022556	5558.410698	5277.022556	714.3481085	772.455394
Malta	6605.414173	6247.596071	6605.414173	6247.596071	750.2562337	861.2896051
Monaco	5766.137072	5501.611225	5766.137072	5501.611225	667.2104355	792.1794071
Netherlands	7205.326559	6754.801055	7205.326559	6754.801055	700.4310553	792.4321506
Norway	7125.287652	6735.84154	7125.287652	6735.84154	876.297499	944.9754918
Portugal	8671.283993	8027.609527	8671.283993	8027.609527	863.1228455	1006.231053
San Marino	5842.025557	5542.621955	5842.025557	5542.621955	721.5066876	795.8641329
Spain	5128.716453	4861.172994	5128.716453	4861.172994	630.5896229	709.0588504
Sweden	4978.752374	4795.127063	4978.752374	4795.127063	669.0521155	734.8049655
Switzerland	7144.273191	6790.609648	7144.273191	6790.609648	795.56525	903.9846012
United Kingdom	4688.923353	4460.202698	4688.923353	4460.202698	648.2637863	695.8392432
Latin America and Caribbean	5734.895558	5502.313658	5734.895558	5502.313658	800.5816663	780.3319336
Andean Latin America	5519.299168	5497.268145	5519.299168	5497.268145	800.4223126	787.7143233
Bolivia (Plurinational State of)	5503.204018	5740.54604	5503.204018	5740.54604	811.7006123	811.1489717

Ecuador	5253.538039	5231.148256	5253.538039	5231.148256	781.4493553	764.6848067
Peru	5662.48449	5551.791336	5662.48449	5551.791336	806.2534463	791.3787604
Caribbean	4549.140379	4400.693978	4549.140379	4400.693978	675.2513443	669.2842895
Antigua and Barbuda	4750.959541	4334.172861	4750.959541	4334.172861	688.3487782	660.8646581
Bahamas	4718.294037	4349.017463	4718.294037	4349.017463	696.2624243	661.6391176
Barbados	4778.529271	4328.705576	4778.529271	4328.705576	656.533185	659.3329498
Belize	4209.554999	4184.063643	4209.554999	4184.063643	676.2800122	647.4601279
Bermuda	4846.749073	4329.228849	4846.749073	4329.228849	638.9571712	660.4113029
Cuba	4700.580437	4246.879069	4700.580437	4246.879069	645.9361366	651.0358947
Dominica	4573.748176	4279.683478	4573.748176	4279.683478	666.0443812	657.1531056
Dominican Republic	4529.638871	4500.350209	4529.638871	4500.350209	690.4857436	679.1030955
Grenada	4575.337529	4282.116156	4575.337529	4282.116156	676.1132884	655.2571479
Guyana	4467.4833	4381.501358	4467.4833	4381.501358	686.5230637	665.6142726
Haiti	4424.43164	4635.317822	4424.43164	4635.317822	706.6872169	692.0262405
Jamaica	4312.030906	4054.724656	4312.030906	4054.724656	654.3880268	632.4645233
Puerto Rico	4924.069053	4442.560873	4924.069053	4442.560873	650.6190181	670.7544048
Saint Kitts and Nevis	4699.590399	4308.868669	4699.590399	4308.868669	688.5912262	660.2465634
Saint Lucia	4741.56644	4307.125921	4741.56644	4307.125921	681.8462652	658.0634428
Saint Vincent and the Grenadines	4568.233625	4299.325433	4568.233625	4299.325433	671.6883564	658.900268
Suriname	4292.055602	4141.954414	4292.055602	4141.954414	653.5844073	639.9651883
Trinidad and Tobago	4190.608027	3871.368675	4190.608027	3871.368675	623.0953007	611.7741652
United States Virgin Islands	4688.627428	4365.09318	4688.627428	4365.09318	639.3158135	663.3945817
Central Latin America	4038.9483	3930.737617	4038.9483	3930.737617	622.6796886	609.1089627
Colombia	4914.838308	4701.662221	4914.838308	4701.662221	696.5291392	690.8855607
Costa Rica	4426.330632	4195.216378	4426.330632	4195.216378	647.9932344	635.8213198
El Salvador	4466.871416	4409.786404	4466.871416	4409.786404	664.7699015	655.6390239
Guatemala	4161.927974	4263.225886	4161.927974	4263.225886	662.2662663	643.3928974

Honduras	3981.556924	4090.964617	3981.556924	4090.964617	643.7872879	626.2969206
Mexico	3628.730866	3513.281376	3628.730866	3513.281376	581.8271354	566.2333561
Nicaragua	4347.113214	4365.797144	4347.113214	4365.797144	676.9953032	653.575515
Panama	3695.626826	3656.682648	3695.626826	3656.682648	584.089227	579.8594301
Venezuela (Bolivarian Republic of)	4135.154616	3999.513443	4135.154616	3999.513443	625.8573771	615.6436292
Tropical Latin America	7942.728072	7378.64183	7942.728072	7378.64183	1025.992134	989.4471214
Brazil	7993.813981	7410.113883	7993.813981	7410.113883	1030.256133	993.5229331
Paraguay	6345.64405	6304.891431	6345.64405	6304.891431	892.687952	862.6583778
North Africa and Middle East	5236.788169	5135.705552	5236.788169	5135.705552	812.5654978	783.0814965
Afghanistan	4485.959646	4851.034741	4485.959646	4851.034741	789.7722027	750.9572834
Algeria	4817.413598	4771.975842	4817.413598	4771.975842	753.8049704	743.4445032
Bahrain	5249.047534	4937.836144	5249.047534	4937.836144	787.2390336	773.2713778
Egypt	4608.694807	4588.392624	4608.694807	4588.392624	762.9901331	727.5203053
Iran (Islamic Republic of)	7537.069867	7268.080256	7537.069867	7268.080256	1040.53155	1027.45011
Iraq	5405.589598	5337.990167	5405.589598	5337.990167	845.2684119	794.335062
Jordan	5033.941412	4933.855189	5033.941412	4933.855189	811.7075532	762.9629845
Kuwait	4955.240655	4599.967648	4955.240655	4599.967648	757.6769932	729.9138759
Lebanon	6073.418397	6051.034588	6073.418397	6051.034588	856.2936036	866.0503414
Libya	5732.676497	5324.334282	5732.676497	5324.334282	844.5195348	804.4529544
Morocco	4955.565982	4799.729282	4955.565982	4799.729282	767.1830924	746.6594721
Oman	4797.687426	4611.540279	4797.687426	4611.540279	760.3431266	734.7694438
Palestine	5450.481814	5545.804272	5450.481814	5545.804272	880.15758	822.8090338
Qatar	4634.567232	4263.869864	4634.567232	4263.869864	724.0006406	703.4296004
Saudi Arabia	4969.793887	4554.428555	4969.793887	4554.428555	768.0453657	724.9686809
Sudan	4655.580754	4745.971051	4655.580754	4745.971051	788.7381272	741.3203726
Syrian Arab Republic	5848.643518	5607.569635	5848.643518	5607.569635	874.9121666	830.3290031
Tunisia	5326.953046	5164.141312	5326.953046	5164.141312	777.8789506	782.0168235

Turkey	4820.31086	4513.604403	4820.31086	4513.604403	723.2247503	714.9819052
United Arab Emirates	4514.070603	4243.272142	4514.070603	4243.272142	720.2314341	702.1563913
Yemen	4605.572102	4783.715579	4605.572102	4783.715579	795.710055	745.0692677
South Asia	3028.34156	3045.525918	3028.34156	3045.525918	510.9719087	497.9035511
Bangladesh	3208.876413	3180.923164	3208.876413	3180.923164	530.7113245	513.4816044
Bhutan	3342.756489	3267.825492	3342.756489	3267.825492	552.2716168	524.3655792
India	3006.064507	2967.435731	3006.064507	2967.435731	504.4235437	486.5398223
Nepal	3176.533946	3277.316167	3176.533946	3277.316167	531.2668034	523.7798669
Pakistan	3017.113794	3474.194048	3017.113794	3474.194048	534.6916504	558.0440276
Southeast Asia, East Asia, and Oceania	3504.780782	3292.851994	3504.780782	3292.851994	541.428206	539.2704252
East Asia	3381.892465	3180.749171	3381.892465	3180.749171	513.9819884	525.0182713
China	3363.598178	3164.182174	3363.598178	3164.182174	512.2250033	522.9718395
Democratic People's Republic of Korea	3804.448087	3612.120163	3804.448087	3612.120163	566.8222207	575.8869666
Taiwan (Province of China)	4014.233324	3665.485418	4014.233324	3665.485418	561.0982707	585.3591777
Oceania	3735.881733	4006.759967	3735.881733	4006.759967	631.0687064	626.0359184
American Samoa	4073.158672	4044.244746	4073.158672	4044.244746	649.4016061	630.7075482
Cook Islands	4280.270171	4085.789945	4280.270171	4085.789945	630.3175615	634.6758944
Fiji	4022.386674	4039.824865	4022.386674	4039.824865	642.8212637	628.2207495
Guam	4073.59833	4013.985059	4073.59833	4013.985059	625.9283388	627.6338271
Kiribati	3876.129088	4098.131534	3876.129088	4098.131534	647.9432963	633.1436976
Marshall Islands	3925.334957	4016.424778	3925.334957	4016.424778	651.350407	628.9452795
Micronesia (Federated States of)	4023.389994	4034.394485	4023.389994	4034.394485	654.6361131	627.5397009
Nauru	3781.416646	4056.545512	3781.416646	4056.545512	648.2414334	629.6910861
Niue	4203.066728	4022.455521	4203.066728	4022.455521	626.9588991	625.8551947
Northern Mariana Islands	4393.867729	4004.408091	4393.867729	4004.408091	643.5270316	625.4246991
Palau	4252.646599	3946.690541	4252.646599	3946.690541	635.4930357	617.8162331

Papua New Guinea	3686.492326	3995.10705	3686.492326	3995.10705	627.9221582	625.2174559
Samoa	3933.757945	4027.853913	3933.757945	4027.853913	656.5328281	628.3853273
Solomon Islands	3688.755937	4028.562574	3688.755937	4028.562574	637.0683597	628.7801226
Tokelau	3850.327926	4003.854175	3850.327926	4003.854175	618.454246	625.4854591
Tonga	3893.173334	4062.97594	3893.173334	4062.97594	638.834315	631.2863275
Tuvalu	4042.010156	4007.320965	4042.010156	4007.320965	638.388401	622.2393264
Vanuatu	3783.426483	4029.280168	3783.426483	4029.280168	643.5823259	630.680931
Southeast Asia	3768.736207	3633.247406	3768.736207	3633.247406	599.6314695	578.6972311
Cambodia	4468.483782	4579.761532	4468.483782	4579.761532	686.8086417	672.8691441
Indonesia	3694.87859	3548.003621	3694.87859	3548.003621	600.1502408	571.6446714
Lao People's Democratic Republic	4419.67414	4570.675361	4419.67414	4570.675361	696.7244499	680.1948149
Malaysia	4757.446501	4599.253126	4757.446501	4599.253126	706.5057962	681.9458311
Maldives	3706.066375	3579.633851	3706.066375	3579.633851	599.4837891	572.533296
Mauritania	2618.960256	2746.234123	2618.960256	2746.234123	475.4109266	461.0460029
Myanmar	4026.228612	3977.017463	4026.228612	3977.017463	630.6670322	613.8678407
Philippines	4398.163776	4563.436938	4398.163776	4563.436938	701.3471952	692.2236839
Seychelles	3993.034028	3737.558727	3993.034028	3737.558727	605.7130512	587.9625824
Sri Lanka	4275.898463	4035.547557	4275.898463	4035.547557	633.6153068	620.063622
Thailand	3843.576766	3399.738242	3843.576766	3399.738242	567.7685862	550.3304039
Timor-Leste	3467.8897	3795.526156	3467.8897	3795.526156	592.8667249	596.0000702
Viet Nam	2427.338743	2287.339705	2427.338743	2287.339705	420.6770046	403.1677035
Sub-Saharan Africa	3106.00276	3462.633915	3106.00276	3462.633915	547.0998724	547.8105551
Central Sub-Saharan Africa	3408.1728	3863.993726	3408.1728	3863.993726	601.7554271	604.3340674
Angola	3354.408511	3934.095509	3354.408511	3934.095509	599.8345568	611.3027624
Central African Republic	3906.823087	4405.564002	3906.823087	4405.564002	660.1480035	659.932905
Congo	3502.101318	3736.703418	3502.101318	3736.703418	601.5597075	591.6787668

Democratic Republic of the Congo	3379.731411	3815.13223	3379.731411	3815.13223	597.9557862	599.2567804
Equatorial Guinea	3512.50334	3804.929931	3512.50334	3804.929931	616.528067	596.1049947
Gabon	3881.489016	3985.120659	3881.489016	3985.120659	636.9498605	618.855984
Eastern Sub-Saharan Africa	3250.797065	3716.292328	3250.797065	3716.292328	568.4412269	575.2188853
Burundi	3100.985025	3638.564344	3100.985025	3638.564344	548.3543645	565.7581478
Comoros	3469.814658	3554.55426	3469.814658	3554.55426	569.5533292	556.0598624
Djibouti	3126.898949	3254.649978	3126.898949	3254.649978	531.1775712	522.3458606
Eritrea	3401.018719	3732.315647	3401.018719	3732.315647	581.0195146	571.3498097
Ethiopia	3035.619082	3465.638475	3035.619082	3465.638475	544.9656018	553.9465448
Kenya	3171.163908	3418.039979	3171.163908	3418.039979	560.0962182	550.7007438
Madagascar	3547.704345	3963.210085	3547.704345	3963.210085	598.5974694	596.778789
Malawi	3397.68534	3845.652595	3397.68534	3845.652595	590.5648378	584.8723071
Mozambique	3357.950876	4035.233578	3357.950876	4035.233578	585.4750645	604.6714664
Rwanda	3376.232879	3653.361704	3376.232879	3653.361704	573.6264526	564.5008606
Somalia	2722.516798	3272.863173	2722.516798	3272.863173	500.8190988	521.9907696
South Sudan	4220.692386	4977.677076	4220.692386	4977.677076	682.7820473	693.4352731
Uganda	3418.107302	4080.157066	3418.107302	4080.157066	596.7341497	611.0138154
United Republic of Tanzania	3301.582362	3812.586228	3301.582362	3812.586228	568.2751088	582.0268613
Zambia	3498.461336	3969.206769	3498.461336	3969.206769	597.1127689	597.5810328
Southern Sub-Saharan Africa	3637.648751	3657.957054	3637.648751	3657.957054	591.4648247	576.080871
Botswana	3560.053324	3553.391325	3560.053324	3553.391325	585.437199	559.6970475
Eswatini	3317.411795	3491.964684	3317.411795	3491.964684	565.0142057	551.1472596
Lesotho	3514.834811	3555.491527	3514.834811	3555.491527	579.3987671	557.721784
Namibia	3333.873276	3491.890128	3333.873276	3491.890128	561.1131508	552.0979271
South Africa	3868.631352	3786.143955	3868.631352	3786.143955	613.9493677	593.9963874
Zimbabwe	2884.47011	3137.016837	2884.47011	3137.016837	517.691674	513.907839
Western Sub-Saharan Africa	2796.684575	3066.502202	2796.684575	3066.502202	504.4460223	499.1539698

Benin	2878.695123	3228.849245	2878.695123	3228.849245	520.4158606	519.0951962
Burkina Faso	2766.201661	3116.734153	2766.201661	3116.734153	503.8272059	504.786482
Cabo Verde	3139.042089	3085.489836	3139.042089	3085.489836	516.2272239	501.8445935
Cameroon	3003.345386	3199.99225	3003.345386	3199.99225	532.1514284	516.1234881
Chad	2861.480137	3369.651449	2861.480137	3369.651449	529.4947946	536.2880966
C??te d'Ivoire	3013.103901	3240.277817	3013.103901	3240.277817	530.7251074	521.2024531
Gambia	3114.612957	3312.686395	3114.612957	3312.686395	543.7706611	528.4637729
Ghana	2919.284572	3000.541584	2919.284572	3000.541584	506.2597618	490.9277668
Guinea	2992.585764	3338.884244	2992.585764	3338.884244	535.221767	532.6329715
Guinea-Bissau	2879.646515	3093.451165	2879.646515	3093.451165	516.2567514	503.4194971
Liberia	3362.291202	3506.651826	3362.291202	3506.651826	574.3893316	550.8355237
Mali	2408.721357	2726.008787	2408.721357	2726.008787	454.9890563	458.9624435
Mauritius	4259.599967	3815.028115	4259.599967	3815.028115	615.4130188	597.4083329
Niger	2497.170738	2967.846173	2497.170738	2967.846173	478.0851688	488.7104784
Nigeria	2722.691218	2991.777576	2722.691218	2991.777576	494.3653102	489.83245
Sao Tome and Principe	3036.578645	3083.788605	3036.578645	3083.788605	527.4424693	502.4320315
Senegal	2729.488586	2893.435513	2729.488586	2893.435513	490.2907218	479.4840308
Sierra Leone	3438.106781	3663.322544	3438.106781	3663.322544	582.0090774	567.7745214
Togo	3369.578995	3592.497291	3369.578995	3592.497291	574.5492712	558.9732867

Table S5 The crude and age-standardized prevalence, incidence, and DALYs rate of depression in different counties and regions in 1990

region	Prevalence rate		DALY rate		Incidence rate	
	Crude	AS	Crude	AS	Crude	AS
Global	3192.572589	3486.16503	543.7401057	588.5658682	3405.393367	3681.235069
Central Europe, Eastern Europe, and Central Asia	3519.059098	3307.317516	592.543053	557.551402	3741.942174	3521.702268
Central Asia	2807.982643	3297.148953	476.5676809	557.382025	2967.844314	3494.68692
Armenia	2674.808807	2836.833658	434.5881285	460.9209836	2608.51924	2801.482649
Azerbaijan	2447.587796	2767.033566	397.0999077	447.9168532	2362.940381	2694.249541
Georgia	3536.371234	3301.654791	599.7339151	559.1785293	3752.290978	3497.354288
Kazakhstan	3235.540372	3496.055083	553.1904425	596.6857594	3488.409286	3797.867464
Kyrgyzstan	3038.077373	3651.578915	527.1591996	631.0263036	3349.709176	4026.344002
Mongolia	3061.586468	3973.933615	548.1005657	702.2300561	3523.767612	4507.335253
Tajikistan	2223.065886	3004.994936	371.9565937	498.9466504	2274.33122	3058.492653
Turkmenistan	2504.504848	3245.990154	426.1635161	548.2977482	2634.618519	3413.547069
Uzbekistan	2558.893426	3322.635149	436.5929595	563.3164797	2723.49423	3531.949099
Central Europe	3077.521457	2822.098168	498.7098586	458.3895995	3007.750078	2763.442345
Albania	2127.173815	2381.43084	332.2818606	369.0236435	1889.897423	2106.356613
Bosnia and Herzegovina	3421.396862	3276.404537	579.7437876	554.7443765	3559.472265	3438.573023
Bulgaria	3496.674838	2994.460815	576.0048308	494.3703456	3543.435389	3024.24903
Croatia	4006.225941	3468.402993	680.2639859	590.7199428	4294.303997	3727.463129
Czechia	3721.502185	3281.647752	624.6708421	552.5180402	3924.437136	3451.794492
Hungary	3950.504652	3414.825179	668.3023307	579.9260418	4230.292318	3651.029539
Montenegro	2959.222114	2863.193597	484.4092944	468.7971191	2910.490169	2827.577664
North Macedonia	2755.309542	2710.867826	442.649324	435.782155	2623.239177	2603.135711
Poland	2431.424675	2279.973045	368.4028994	346.8835223	2066.929398	1952.977365
Romania	2953.268472	2717.38089	474.2435916	436.441773	2842.058807	2606.804321

Serbia	3448.040513	3098.019184	573.7600363	516.7272837	3526.767542	3179.525165
Slovakia	3104.405483	2926.542863	507.826512	479.2960277	3093.938875	2918.303001
Slovenia	4270.428251	3779.249076	740.1746878	656.8340941	4720.167039	4190.23429
Eastern Europe	3976.215541	3570.146839	678.9498672	611.0568112	4377.249655	3937.847831
Belarus	4353.8628	3923.491195	760.4116985	686.3478197	4935.846642	4446.95455
Estonia	5288.758421	4681.627041	951.6211513	844.5547706	6320.808132	5593.35158
Latvia	5025.524043	4365.632607	890.3296806	774.9431878	5905.506988	5116.507964
Lithuania	4709.411117	4258.719035	832.740209	754.6372087	5475.40474	4954.272204
Republic of Moldova	3762.200516	3692.289388	647.8954975	635.9761803	4169.415112	4105.759871
Russian Federation	3576.782179	3241.062011	596.7832925	542.3216103	3770.418103	3432.120977
Ukraine	4921.451037	4264.884354	871.4855007	756.6148112	5812.314385	5021.055683
High-income	3861.520965	3504.753695	664.7072594	606.7167068	4199.613144	3842.26343
Australasia	4552.932939	4262.865207	819.763298	769.9290161	5334.343647	5029.445213
Australia	4705.749193	4398.444884	852.4958742	799.4735835	5569.443148	5244.396944
New Zealand	3798.972697	3586.067553	658.2682975	622.4955522	4174.416793	3955.959168
High-income Asia Pacific	2266.690345	2049.876993	388.719336	352.4992373	2437.368901	2216.420988
Brunei Darussalam	1439.276842	1600.544645	236.7174639	259.0280681	1408.142955	1571.847422
Japan	2336.360547	2061.494082	401.5027087	356.7335101	2528.932344	2248.717557
Republic of Korea	2018.94948	1965.027167	340.9832011	329.0890223	2096.502175	2043.505893
Singapore	3063.827999	2845.313903	568.1663786	524.551748	3702.325513	3465.61307
High-income North America	4037.899069	3748.412113	668.2835146	624.5262456	4046.94101	3805.680569
Canada	3608.721242	3296.184594	606.1143242	556.9018525	3643.103387	3378.047713
Greenland	7158.456106	6378.719917	1377.953415	1214.379475	9048.842996	8070.440907
United States of America	4083.342114	3796.486771	674.8097733	631.6150409	4089.248261	3849.980715
Southern Latin America	2985.40314	3057.347621	542.3803156	554.2616515	3573.13906	3648.766848
Argentina	2523.084407	2591.440121	445.1521191	456.586582	2889.944161	2957.881793
Chile	4215.498734	4364.126235	802.6098472	826.4844197	5405.523446	5578.45509
Uruguay	2659.369052	2572.944278	467.3261991	453.4505091	3029.572973	2931.238096

Western Europe	4528.604641	3974.364656	794.1905349	702.174053	5127.047596	4536.449907
Andorra	4174.093491	3668.154064	727.0341131	640.4367721	4553.827959	4058.742953
Austria	4154.86764	3631.615942	712.6909768	628.7108888	4513.466176	3996.177288
Belgium	3784.438256	3304.333	647.2211521	570.2161018	4120.584641	3635.173324
Cyprus	3231.986058	3111.477467	540.7608213	521.6363857	3321.871944	3216.619944
Denmark	5119.605461	4390.982325	911.7115882	787.0272398	5952.031599	5138.944931
Finland	5483.094554	4936.904762	980.1806277	892.3039758	6262.149069	5744.871326
France	4958.125505	4436.945328	887.5894984	798.6950606	5790.967485	5210.345986
Germany	3740.362269	3187.438785	630.3131441	542.2140542	3973.084124	3422.970337
Greece	5702.393792	4992.988787	1042.77903	917.4786027	6863.396384	6034.198657
Iceland	3342.198151	3207.431923	564.4232711	542.607023	3495.950625	3365.110527
Ireland	4088.860628	4037.525411	721.7522132	713.2540384	4625.704833	4557.890447
Israel	4218.474786	4364.162128	761.0154465	785.7321886	4953.583341	5097.056993
Italy	4413.392768	3734.004885	765.8106071	652.2629159	4962.763568	4218.487752
Luxembourg	4417.106894	3833.399308	767.4673145	671.7313999	4883.632282	4301.354875
Malta	3404.928739	3187.724843	570.8375629	537.1562982	3520.469744	3325.78344
Monaco	5044.365063	4066.072269	881.8230444	723.2556353	5667.88167	4646.795493
Netherlands	4131.969134	3620.623228	723.4523799	636.6277647	4616.05316	4075.205719
Norway	3299.802494	2961.463098	542.4501203	493.0140427	3276.355881	2992.501337
Portugal	5489.085404	4921.019456	996.2931358	896.7570713	6597.357231	5927.217387
San Marino	4534.666758	4049.459997	801.7724479	719.8169348	5142.826785	4625.135028
Spain	4548.441622	4083.787841	798.0538633	720.1941003	5101.93951	4600.782415
Sweden	4799.262524	4192.653084	842.7210711	744.3420753	5427.59519	4792.788087
Switzerland	5079.505356	4394.794207	894.0571728	780.504751	5760.141504	5054.306673
United Kingdom	5128.465279	4559.13013	914.1783523	819.9012343	5994.924783	5387.91914
Latin America and Caribbean	2954.693494	3485.596614	531.5834663	619.395954	3509.294496	4091.364913
Andean Latin America	2319.77368	2845.58948	401.3585049	486.2326986	2537.848233	3064.130348
Bolivia (Plurinational State of)	2804.589609	3676.683358	504.6355084	655.2812153	3315.759082	4301.226212

Ecuador	2738.815565	3282.507486	490.7057019	579.0774196	3161.916052	3712.497065
Peru	1983.167982	2416.980209	329.6160643	397.1335919	2020.036012	2426.738708
Caribbean	3722.957091	4038.701356	684.6200611	737.1062347	4534.556993	4880.624666
Antigua and Barbuda	2857.435492	3026.534632	497.6595611	524.8227767	3188.622947	3361.333655
Bahamas	2848.013129	3097.491827	501.0044782	539.8712179	3202.423275	3466.237634
Barbados	3260.093598	3144.17727	571.0510107	550.988197	3667.81671	3536.644997
Belize	2454.209715	3207.269791	439.5349481	565.0926865	2856.011463	3636.973392
Bermuda	3975.940954	3583.931681	713.1737355	645.147064	4595.241614	4198.351011
Cuba	5131.913818	4879.077665	967.8915347	916.2566645	6478.45845	6147.219102
Dominica	2811.631124	3068.698524	491.6440626	533.479316	3183.226782	3426.246735
Dominican Republic	3102.714269	3767.858656	567.5825368	681.4603993	3733.941246	4479.055485
Grenada	2777.728875	3235.959189	491.4314872	568.1936385	3207.395988	3673.774684
Guyana	3946.884581	4598.088787	737.7709045	845.6526838	4994.290143	5720.208427
Haiti	2953.603378	3722.677957	534.1990155	664.4446983	3557.872585	4407.849885
Jamaica	2664.729008	3030.774838	468.3783201	527.2721088	3010.419116	3370.180178
Puerto Rico	3003.039965	2980.282162	519.2143305	514.6364454	3320.457032	3287.585037
Saint Kitts and Nevis	3544.528275	3954.31365	645.9795696	717.1376299	4304.817808	4760.906317
Saint Lucia	2751.278374	3282.894929	488.8216229	575.237227	3194.452762	3743.026916
Saint Vincent and the Grenadines	2739.080344	3261.215659	487.9287791	573.270868	3181.404212	3713.920035
Suriname	4066.419904	4468.259877	760.7897739	827.3334604	5085.059974	5526.194284
Trinidad and Tobago	3691.962491	4013.620289	679.6376765	732.2460199	4488.985473	4840.194873
United States Virgin Islands	3183.892998	3263.83167	563.222648	576.1069376	3621.860553	3714.272226
Central Latin America	2391.913623	2985.130396	420.1473874	517.2979616	2714.319564	3351.05484
Colombia	2064.809907	2414.381023	351.7324506	406.021062	2205.876119	2557.275828
Costa Rica	2601.995659	3083.564601	461.8195783	541.9845689	2975.61323	3502.705867
El Salvador	3063.011356	3710.512824	562.6583876	670.6708727	3753.026312	4431.313835
Guatemala	2700.040292	3785.57564	490.3931569	680.8441257	3280.680635	4549.44915

Honduras	2099.181492	3036.260255	369.8381696	528.838708	2406.095555	3439.390831
Mexico	2408.370593	3049.038853	422.1823505	526.6436297	2730.058335	3418.43782
Nicaragua	2318.173766	3218.756766	417.6113211	567.7691158	2749.640464	3702.404501
Panama	2575.023974	2971.804232	452.805049	518.3373579	2913.867403	3342.710987
Venezuela (Bolivarian Republic of)	2595.781296	3176.540893	461.811692	559.4687848	2993.811566	3644.444617
Tropical Latin America	3540.149332	3995.247362	648.4238798	722.907614	4368.760097	4873.331896
Brazil	3557.87705	4007.266862	651.8523511	725.3328987	4394.116276	4893.004089
Paraguay	2887.941605	3488.879521	522.2894509	620.2532364	3435.899077	4044.949646
North Africa and Middle East	3523.507589	4329.061034	642.6896064	777.0002645	4226.938208	5068.916261
Afghanistan	3851.845502	4996.11804	697.6386196	895.2256481	4769.058164	6046.05048
Algeria	3384.34678	4252.145623	617.9653319	763.0016436	4040.406016	4942.95879
Bahrain	4770.885015	5038.744037	898.4136249	932.7826018	5885.24114	6171.474578
Egypt	3161.008936	3792.095539	561.5947493	664.784009	3613.79149	4253.716976
Iran (Islamic Republic of)	3663.502855	4661.584351	679.9558953	848.1241935	4539.979238	5599.496189
Iraq	2961.957401	3922.524799	529.0894916	686.4964324	3473.650695	4448.655461
Jordan	3452.990945	4553.896416	639.958359	826.3324877	4232.959991	5415.263894
Kuwait	3717.337058	3911.983495	673.0647241	698.1641832	4302.532972	4492.339694
Lebanon	3843.843201	4439.65776	695.2893007	796.5959274	4569.268315	5216.691791
Libya	3288.116173	4226.450171	602.56272	759.4305914	3960.045172	4927.865227
Morocco	4357.345665	5183.346448	814.347124	957.8304701	5416.79874	6341.629715
Oman	3157.412561	3946.900605	573.1140412	703.2550247	3706.642357	4544.165933
Palestine	4497.298044	6296.82671	863.554087	1189.645775	5872.62949	8000.346164
Qatar	4262.086649	4199.023185	785.1353324	760.1460423	5054.657801	4961.326867
Saudi Arabia	3265.067344	4015.477277	589.8359247	709.3937221	3803.109241	4555.994392
Sudan	3500.298588	4505.252266	644.9813557	815.3429729	4260.75397	5319.242053
Syrian Arab Republic	3042.903369	4112.071202	554.3861392	733.5463525	3634.967957	4737.032556
Tunisia	4303.742571	5053.801597	804.0324543	933.9708009	5310.81832	6154.561751

Turkey	3605.181763	4044.074859	648.3191269	717.2095242	4217.352019	4641.146898
United Arab Emirates	3661.615797	3820.061493	663.1152364	680.0463895	4221.802065	4382.357536
Yemen	3322.953676	4857.034889	615.9498359	882.9604444	4149.789278	5854.237084
South Asia	3208.92213	4078.714742	559.878341	704.1527947	3682.399046	4653.993959
Bangladesh	3470.376349	4759.04936	629.4883198	850.6158635	4182.388244	5641.320284
Bhutan	3016.651563	4262.521007	529.5312196	744.3196073	3455.669224	4899.219488
India	3219.444488	3984.668341	558.4961142	683.7624236	3671.069649	4520.75858
Nepal	3382.397575	4655.906558	599.4967949	819.7419422	3982.423375	5479.420312
Pakistan	2847.454434	4055.531241	496.3877496	702.8732258	3234.360114	4588.143597
Southeast Asia, East Asia, and Oceania	2697.046723	2911.754232	429.1674264	452.2914899	2428.950055	2516.512061
East Asia	2873.413967	2984.92484	460.8999416	468.3923865	2624.256506	2630.788229
China	2884.520352	2995.187186	463.5325559	470.6505209	2644.581637	2647.717378
Democratic People's Republic of Korea	2708.279595	2895.301435	418.0038818	444.1300014	2272.042347	2425.058567
Taiwan (Province of China)	2399.395651	2464.388305	352.414378	358.3720423	1808.403152	1831.871782
Oceania	2504.942767	3109.937566	411.9070592	490.8686723	2443.659242	2810.217969
American Samoa	2177.204756	2681.32058	341.0767412	402.7207135	1906.540018	2172.365257
Cook Islands	2747.105765	3121.219892	448.20941	497.3238353	2608.350811	2833.971677
Fiji	2452.575814	2904.687985	391.8261903	447.7043284	2245.170867	2494.132428
Guam	3014.885841	3165.896817	500.8524543	509.986239	2899.165856	2905.000531
Kiribati	2569.980068	3131.921546	418.7432885	491.1567386	2470.588558	2813.406021
Marshall Islands	2042.136186	2967.658651	336.0766189	462.4002198	1993.954782	2598.182246
Micronesia (Federated States of)	2222.792626	3017.695803	363.7714926	473.0378065	2149.032415	2669.702875
Nauru	2367.892847	3108.945645	391.4732761	494.5585654	2293.892195	2816.18669
Niue	2812.208052	3148.049726	448.4453937	499.5500717	2592.022305	2849.650996
Northern Mariana Islands	2581.422028	2659.017973	406.6354474	404.9631972	2228.238389	2185.68552
Palau	2914.497424	3124.222559	475.4163962	496.0782179	2762.15271	2829.609581

Papua New Guinea	2551.609735	3180.106712	422.2509023	504.700736	2528.751933	2916.056411
Samoa	2373.493639	2975.174659	388.4525335	465.5594763	2280.033527	2603.17701
Solomon Islands	2228.182126	3081.796177	370.1161115	488.5575588	2211.869866	2780.782707
Tokelau	2542.362454	3171.111363	409.4472684	503.9064563	2376.823005	2865.045054
Tonga	2147.998685	2716.464149	336.4355501	409.0139623	1898.472488	2200.998086
Tuvalu	2843.539776	3185.174048	456.405827	504.4686383	2616.220037	2870.268373
Vanuatu	2334.987474	3084.83451	384.6157277	487.5339249	2283.811592	2783.268564
Southeast Asia	2236.816316	2673.655739	346.121582	400.644135	1916.145375	2151.867331
Cambodia	2361.671185	3245.886649	389.7518292	514.1767698	2324.252958	2951.643359
Indonesia	2035.89992	2434.134004	303.9795231	351.4124033	1619.97553	1812.254655
Lao People's Democratic Republic	2241.704586	2946.111983	360.493297	456.4048944	2080.577622	2531.262806
Malaysia	2704.742156	3284.33966	444.3615544	527.0059576	2610.061429	3051.157205
Maldives	2539.589245	3545.870406	437.5440286	586.9974384	2681.632473	3470.817753
Mauritania	2680.411446	3762.786023	450.8768536	630.5862883	2787.003689	3920.450797
Myanmar	1779.350495	2196.305176	250.2203193	299.0623864	1236.640204	1415.496988
Philippines	2418.987314	3092.799604	394.1647989	488.1489055	2338.59472	2824.339673
Seychelles	2527.097757	2811.202252	396.6209738	431.385004	2207.80067	2343.152163
Sri Lanka	3148.331767	3368.703689	526.2819374	548.8710479	3111.117397	3182.321844
Thailand	2682.291777	2848.442139	422.416031	436.7725331	2357.139027	2391.660768
Timor-Leste	2352.057067	3071.99937	385.9931477	483.3445805	2262.982745	2744.079121
Viet Nam	2115.387483	2624.291598	325.0974809	389.6939627	1770.821058	2037.795266
Sub-Saharan Africa	3310.082585	4703.68226	578.5965169	817.6514745	3746.833038	5324.45267
Central Sub-Saharan Africa	4105.082645	5703.800265	749.1516564	1027.240775	5039.8336	6893.341549
Angola	4338.185552	5950.868984	804.2205445	1089.210868	5385.191421	7280.191306
Central African Republic	4491.942846	6007.172537	825.7397737	1089.947668	5571.382151	7350.114132
Congo	4382.606008	5957.725348	810.7252753	1087.834889	5447.840916	7275.261573

Democratic Republic of the Congo	3985.527708	5590.444768	722.6810311	1000.019282	4868.154733	6718.616677
Equatorial Guinea	4300.509185	6070.371906	788.6876779	1100.547947	5346.27137	7433.642547
Gabon	4492.691656	5746.222188	825.3884711	1045.667813	5517.612446	6966.357391
Eastern Sub-Saharan Africa	3407.569581	5090.677181	596.867508	889.5819822	3873.75143	5822.005466
Burundi	3752.380121	5548.222592	669.0244009	988.2973089	4363.64543	6500.259917
Comoros	3105.706019	4422.405068	530.5940591	754.120439	3354.130841	4791.082438
Djibouti	3001.469715	4437.88401	517.0586388	764.3280463	3225.593656	4861.761877
Eritrea	3340.944018	5040.201424	578.9812089	874.3765056	3748.963378	5733.138658
Ethiopia	3385.353912	5088.258326	594.0294905	889.5628603	3848.485066	5818.3326
Kenya	3179.495012	4998.429483	558.3046777	876.1053681	3615.796918	5741.044338
Madagascar	3373.887369	4868.791049	587.2860178	846.3119403	3774.052721	5482.949929
Malawi	2950.856603	4274.144	496.0714407	717.5922313	3123.622627	4570.240372
Mozambique	3400.22105	4837.167915	585.5204792	830.8291825	3784.908873	5406.386958
Rwanda	3869.699334	5769.220252	694.8168834	1034.552847	4559.800961	6842.890244
Somalia	3227.320514	4874.212763	560.2184779	846.281294	3606.513883	5493.38388
South Sudan	3264.270433	4626.156159	559.8600532	789.0798744	3606.314531	5146.720214
Uganda	4367.611188	6775.589772	800.7380195	1237.965057	5386.773097	8364.866701
United Republic of Tanzania	3286.065595	4822.181247	566.8090401	830.4493549	3662.312905	5397.886017
Zambia	2809.333835	4218.682513	472.5189697	708.296878	2972.171602	4493.320355
Southern Sub-Saharan Africa	3302.480895	4136.873518	566.5841058	704.669459	3603.864413	4504.429015
Botswana	3017.824883	4167.696443	517.3527438	708.8997237	3279.562678	4507.708675
Eswatini	2777.419448	4150.68063	477.6947815	706.8799057	3013.290451	4477.991263
Lesotho	4156.811959	5434.980811	754.4355679	978.571974	4965.054148	6435.490894
Namibia	2758.241993	3655.459723	458.9834231	602.82664	2846.814875	3743.590886
South Africa	3608.535411	4277.711699	624.2924656	734.4757728	4001.445492	4729.34579
Zimbabwe	2213.846951	3308.466048	355.9681922	530.8154387	2139.687304	3231.213596
Western Sub-Saharan Africa	2986.695969	4247.926491	514.6588491	728.8385073	3287.711295	4690.813635

Benin	2827.727743	4349.002761	487.7055883	746.1523521	3118.89194	4798.728062
Burkina Faso	2942.067313	4462.98578	507.9142044	766.7846445	3280.032764	4964.858128
Cabo Verde	3392.37567	4599.740321	600.3382069	808.380862	3868.014246	5160.391846
Cameroon	3091.523911	4488.010981	535.8932291	774.1982629	3447.650557	5020.034557
Chad	3372.382764	5033.885493	601.2685798	892.7021152	3927.520962	5838.399326
C??te d'Ivoire	2641.513478	3927.703626	442.9033788	655.3462321	2782.371009	4193.80972
Gambia	3667.118957	5524.969482	667.7445507	1002.371116	4387.292835	6630.345551
Ghana	3091.742333	4356.544935	535.7895606	751.713824	3404.573067	4820.72057
Guinea	3051.989679	4239.600184	524.9671994	726.4640229	3341.166477	4639.195596
Guinea-Bissau	2949.69573	4362.630326	510.2411926	750.3547857	3256.285141	4820.392677
Liberia	3330.330392	4579.233246	574.146858	785.6925522	3784.587899	5169.22896
Mali	2507.770612	3610.786075	413.6407098	592.8745336	2558.573973	3689.079256
Mauritius	4036.325847	4198.978431	707.9089397	721.5347843	4351.298228	4396.182981
Niger	2650.86563	4153.608074	455.792689	710.7829228	2881.041423	4524.019654
Nigeria	3081.144745	4239.531356	532.6385673	729.8503815	3413.994367	4713.619489
Sao Tome and Principe	2617.099494	3694.460703	442.1814332	617.1156936	2755.00868	3813.350381
Senegal	2681.648182	4003.507179	454.819066	676.249869	2863.575964	4286.271562
Sierra Leone	3100.83276	4179.010092	530.2330263	712.416365	3371.340384	4556.635098
Togo	2846.20754	4380.63118	493.2311612	755.6875131	3138.950713	4848.223999

Table S6 The crude and age-standardized prevalence, incidence, and DALYs rate of anxiety in different counties and regions in 1990

Region	Prevalence rate		DALY rate		incidence rate	
	Crude	Age-standardized	Crude	Age-standardized	Crude	Age-standardized
Global	3643.529929	3791.585446	348.814014	360.5469957	581.810232	579.3010609
Central Europe, Eastern Europe, and Central Asia	3201.177044	3093.641942	303.567135	294.3041993	490.3702198	484.6334042
Central Asia	2090.714597	2252.440086	201.7253485	215.6643812	367.7967777	375.2463261
Armenia	3045.695156	3116.635847	292.4326689	297.8680571	478.1087886	475.5749248
Azerbaijan	2419.092955	2535.779981	234.0198258	243.5272286	410.5326574	412.0030794
Georgia	2618.377689	2530.586467	251.0236978	243.150821	417.3076686	411.2175418
Kazakhstan	2061.842511	2120.996883	198.0002867	202.5289727	359.6011452	358.9520633
Kyrgyzstan	1890.045148	2056.024023	182.3087517	196.7719205	342.5468171	351.7903479
Mongolia	1862.984614	2098.903865	180.5724011	200.8494752	347.4964885	358.2057028
Tajikistan	2047.3683	2373.344103	198.8057791	227.8921906	373.1236334	393.2703645
Turkmenistan	2026.069676	2281.090187	196.6391957	218.8211649	367.953393	380.5547795
Uzbekistan	1792.645158	2022.093319	173.5440667	193.7010417	334.3143022	348.1745682
Central Europe	3479.55087	3293.185954	330.1337601	313.8161597	518.5767797	507.8085526
Albania	3466.205624	3659.14498	334.4391781	349.9860111	550.2210185	545.2166468
Bosnia and Herzegovina	3672.554903	3538.239762	351.0634703	337.4102348	549.7837528	530.4768477
Bulgaria	3748.901287	3429.313005	354.9615575	327.6553669	530.0339493	520.7510268
Croatia	3840.036019	3531.348994	363.2510068	336.484238	542.8495513	530.0014703
Czechia	3448.169705	3193.5308	326.4376312	304.5332994	504.6779528	494.8661359
Hungary	3756.72866	3436.695554	354.4898224	327.2231827	530.4032224	520.1728636
Montenegro	3743.017134	3643.487041	358.2113674	348.574326	551.6532901	540.7332095
North Macedonia	3656.613485	3581.323103	350.0338168	342.2063622	549.7461763	537.019589
Poland	3246.854798	3115.519914	307.3752074	296.0129418	503.3913074	493.5784729

Romania	3457.229556	3276.012116	328.576894	312.6035194	515.8206796	505.8401801
Serbia	3517.359091	3298.80498	334.7343087	315.4299107	521.30035	507.9560243
Slovakia	3597.110633	3454.837857	342.2438867	329.5381142	531.1877057	522.4345482
Slovenia	3687.214343	3420.122371	349.3282174	325.8024668	530.5697546	517.9053594
Eastern Europe	3389.635913	3217.251521	320.2881333	305.8500269	512.5407581	506.7938036
Belarus	3704.453899	3531.769141	351.5124316	337.0843276	537.0983834	535.4648056
Estonia	3611.748239	3425.218364	342.0963569	326.899296	522.5659172	523.9649228
Latvia	3987.997242	3760.461408	376.0621276	357.789742	554.2947437	558.4421883
Lithuania	4495.123711	4270.562395	425.7279473	406.7719162	608.3387102	608.6436558
Republic of Moldova	4152.43215	4115.173379	395.3730991	391.9045731	600.0050939	595.0932953
Russian Federation	3332.868082	3170.2214	314.7316247	300.9555123	509.3889232	502.308217
Ukraine	3311.49836	3109.181908	312.854982	296.2823881	500.2232853	495.8881329
High-income	5181.918207	4881.19181	490.2665794	464.4819485	701.3749558	707.2199517
Australasia	6249.493112	5965.958054	594.5341634	568.9282706	824.2844429	821.027703
Australia	6033.826216	5746.219249	574.4395758	548.3905774	800.2017547	795.5174415
New Zealand	7313.543964	7046.516143	693.6762358	669.78437	943.1028862	946.1054422
High-income Asia Pacific	2989.479092	2810.56332	287.2381054	270.8706131	472.609908	465.0173313
Brunei Darussalam	2770.756413	2849.610466	269.8736418	273.6138646	487.674472	469.498552
Japan	2850.964653	2667.616981	273.2281839	257.2677269	449.4947959	446.9692683
Republic of Korea	3384.918755	3238.053064	327.0085826	310.7197045	537.4573718	508.7337041
Singapore	2975.690036	2774.093444	288.7269401	268.0029576	482.5647975	460.7338645
High-income North America	5534.368766	5161.897303	518.4462619	485.5309461	761.654261	745.7960253
Canada	4793.55785	4435.20982	458.1292331	424.9902147	686.9719667	664.4616518
Greenland	5033.425392	4732.386228	477.0924876	445.8359245	754.8715867	698.1160412
United States of America	5614.098966	5241.197177	524.938041	492.1435516	769.682405	754.720056
Southern Latin America	5104.222337	5150.6547	489.899205	493.5396463	723.6385531	717.4024719
Argentina	5004.911776	5072.14727	480.7284795	486.7415589	726.7971416	723.907146
Chile	5357.945293	5387.169504	513.6666848	513.9577733	723.1825383	704.6789636

Uruguay	5078.660817	5023.510295	486.1101937	482.3503038	692.2445799	705.356341
Western Europe	5867.319307	5543.381508	555.8296641	529.8053231	751.2039318	790.5893625
Andorra	5734.495106	5327.680282	550.2988804	512.1325963	785.1738926	786.0333044
Austria	6063.484709	5717.314342	574.2259904	546.7811263	765.2977559	815.6158861
Belgium	5239.221419	4990.336623	497.2845737	478.3910963	710.8975622	753.5717316
Cyprus	6464.498371	6309.203227	619.5092964	605.4040859	871.1987624	872.758637
Denmark	5098.695055	4809.989292	482.436698	459.7939121	685.8038751	728.1114044
Finland	4344.975215	4278.275307	412.9642249	410.3809362	649.7078977	693.4584347
France	6550.906371	6260.384793	621.9535978	598.7178006	812.7949028	854.858662
Germany	6309.65396	5887.692701	596.0504406	562.5493458	759.3868168	819.2577145
Greece	5875.786883	5549.188071	559.0590537	531.918904	776.0943511	802.3413895
Iceland	5193.792695	5073.173464	497.256987	486.5319047	749.3693397	755.6160564
Ireland	6628.230424	6467.678953	633.7424196	618.8947732	899.5281606	886.787495
Israel	3677.472172	3692.708252	353.923406	354.7707646	613.6142909	605.5531082
Italy	6438.179628	5948.303368	607.4473418	566.8319996	818.5991317	851.9184567
Luxembourg	5604.086939	5320.415926	531.2539595	509.0577601	732.9861908	778.8810542
Malta	6412.328529	6221.290368	612.4970654	596.3471816	850.9600649	863.6105899
Monaco	5809.017998	5436.551169	546.3069439	521.5486258	677.4347372	789.1493704
Netherlands	6798.165327	6378.220278	648.4654486	612.219057	725.6945535	765.0019349
Norway	7558.177376	7164.134492	715.1181	684.7443138	922.5452152	979.5791213
Portugal	8260.401531	7763.925127	782.9025091	740.3142735	970.0472282	993.1059588
San Marino	5746.338404	5426.943926	548.4001641	521.2289018	762.8540779	790.120484
Spain	4929.472534	4609.905172	469.3763714	441.6863889	677.3238412	686.7786794
Sweden	4936.638327	4726.138827	467.3597053	452.893083	674.754647	726.0210394
Switzerland	6844.667131	6455.808754	645.1337329	614.6183459	821.3532576	883.767517
United Kingdom	4284.450526	4181.258192	405.2986957	399.1226897	635.3138302	674.7434385
Latin America and Caribbean	4351.593335	4740.587037	417.6458411	450.0592936	708.8406346	708.6668688
Andean Latin America	4854.203604	5403.048871	468.3378211	516.361444	776.7213453	779.6245709

Bolivia (Plurinational State of)	4875.129797	5653.580145	468.3288958	537.2925376	781.277096	801.856736
Ecuador	4636.900899	5117.839671	448.3535528	490.0772965	752.9361205	752.0945161
Peru	4948.283395	5463.79034	477.5611888	522.5464769	786.3496396	785.9577894
Caribbean	4127.444119	4279.433861	396.6067698	408.9146507	658.4025435	655.6809206
Antigua and Barbuda	4176.175066	4282.0877	401.5848354	410.2783923	652.3466318	652.8430862
Bahamas	4199.579402	4266.156774	405.7117549	408.7633679	678.1167277	652.4584833
Barbados	4392.775229	4263.395198	421.6903032	409.5162689	655.3624601	649.7089524
Belize	3521.560072	4059.113868	342.1289274	390.0755948	617.6014437	633.4554521
Bermuda	4587.682862	4249.513956	440.4112557	408.5205486	674.0834764	649.8890003
Cuba	4382.679742	4198.104651	419.9799202	401.5994964	664.1440706	646.8380974
Dominica	4030.855315	4232.819109	387.5851538	405.7621634	633.6035255	644.9788585
Dominican Republic	4082.412961	4474.08026	395.3313991	429.1400149	672.9921807	676.4637635
Grenada	3887.1762	4251.86765	373.8483102	406.799097	632.7003455	648.8618234
Guyana	4034.948441	4344.071693	386.0487449	410.5331476	670.3493154	663.3053851
Haiti	3886.107845	4452.076875	371.8757259	421.6695269	654.9060209	673.5604779
Jamaica	3692.827371	3926.454243	356.7085205	376.9368287	613.7751617	617.014275
Puerto Rico	4390.195665	4329.330639	420.411162	414.3389427	666.338497	657.8786247
Saint Kitts and Nevis	4041.601051	4264.953983	388.1749357	407.7123085	643.8810804	651.8608566
Saint Lucia	3880.117891	4258.564608	373.1231953	405.6545792	645.1363383	652.7673833
Saint Vincent and the Grenadines	3900.838664	4237.14125	376.8986455	405.5399085	649.2118133	649.1712049
Suriname	3920.32878	4107.149754	376.9440853	392.0602156	643.1180417	636.8782824
Trinidad and Tobago	3624.66046	3765.744685	348.9372811	360.0651339	606.4891367	598.2475368
United States Virgin Islands	4260.839983	4264.891395	410.1669699	409.4128523	668.9462438	654.5416982
Central Latin America	3222.348437	3520.921252	311.5483459	335.9550795	552.74633	553.4678949
Colombia	4368.632501	4605.985149	423.0212971	441.2516939	694.3249596	679.8807497
Costa Rica	3788.311418	4031.589753	366.9713529	386.7852508	624.3347946	617.7755099
El Salvador	3850.553785	4208.30661	370.3092824	400.6453836	640.4702749	635.791053

Guatemala	3483.104988	4067.127935	334.6403647	385.9581799	605.5844955	622.9187337
Honduras	3309.241359	3874.954004	320.9942079	370.7896385	588.2124543	600.6927979
Mexico	2611.232446	2883.438692	252.3969756	274.3020386	470.3358708	477.6913477
Nicaragua	3606.864737	4229.373881	348.4824389	402.8356103	630.4445507	637.6284013
Panama	3360.18951	3490.355395	324.8866439	334.7925187	569.2326343	559.1616009
Venezuela (Bolivarian Republic of)	3519.444789	3754.991405	340.5807632	359.4246253	596.719381	587.5709871
Tropical Latin America	5490.084144	5900.307168	523.7408385	557.158286	871.099794	861.541329
Brazil	5495.189239	5894.824362	524.1115131	556.5016265	872.3362455	861.8447759
Paraguay	5302.266244	6134.64505	510.103613	584.8534675	825.6103946	848.7456979
North Africa and Middle East	4660.785291	4950.322459	451.5075293	474.0867837	785.5594457	752.092109
Afghanistan	4263.01142	4713.313908	402.3185411	440.3300005	727.6487288	723.2728438
Algeria	4390.298852	4626.097028	427.5591426	445.0698101	753.6921653	712.7627323
Bahrain	4923.426917	4947.829996	477.4588398	474.4963434	792.1254766	756.0577212
Egypt	4189.757154	4415.51307	406.1427097	424.0810817	713.1909512	690.6152549
Iran (Islamic Republic of)	6415.52643	7036.18162	621.5068119	672.0669323	1042.669828	988.8285206
Iraq	4557.973363	5046.905924	439.9786107	480.1305127	776.8060578	748.7878622
Jordan	4501.59922	4790.484745	439.3567219	460.3041771	774.6875568	730.4382187
Kuwait	4310.068771	4246.118937	419.5511986	409.6323482	719.2681116	680.8576266
Lebanon	5645.467282	6009.57584	542.7146112	573.891777	857.2634058	846.0578705
Libya	4735.036222	5080.207755	461.6584274	488.6931484	806.2402279	763.8129475
Morocco	4476.80203	4634.342312	433.1003375	443.9742817	748.0339834	715.0263707
Oman	4249.685638	4581.310556	414.4911777	440.6920524	751.2193925	714.9003767
Palestine	4756.064357	5437.921262	459.7294095	518.0701552	809.5258254	792.8888665
Qatar	4340.39878	4299.359742	422.0699288	413.6101365	716.6201171	688.8868644
Saudi Arabia	4150.188961	4352.494933	405.2160378	418.6919015	729.0270949	691.4712241
Sudan	4245.55081	4666.293646	411.2662167	446.8927844	734.0624668	718.3157785
Syrian Arab Republic	4762.719571	5254.510067	464.1010057	504.8922143	819.0816587	779.5663023

Tunisia	4764.488453	4916.477556	463.6861078	474.157593	776.0413874	743.1492755
Turkey	4045.968315	4109.111427	391.5824839	394.0034554	680.5831847	652.775173
United Arab Emirates	4079.639606	4131.554602	397.2664729	397.680771	702.5019959	671.4470656
Yemen	4068.83048	4771.014676	393.2106539	453.7242681	745.8588183	726.1528258
South Asia	2601.476191	3023.124366	246.6162349	282.7780678	464.0065815	492.2207158
Bangladesh	2472.956216	3082.626223	237.223068	291.3476337	451.8702554	496.528751
Bhutan	2629.365624	3183.615562	252.3669901	300.7529038	473.8579768	505.8878929
India	2616.195515	2980.043374	247.3183179	277.9933059	464.4729571	486.4399738
Nepal	2416.58547	2945.894877	230.1715825	276.9489666	439.0626584	480.9326229
Pakistan	2645.939124	3334.210888	253.1869945	315.1365553	476.46511	537.042266
Southeast Asia, East Asia, and Oceania	3436.830249	3486.697372	332.8977507	334.5430097	562.1291717	552.6951546
East Asia	3528.973893	3516.437396	342.4546444	338.4075078	564.5354571	557.9990364
China	3521.842373	3507.308547	341.7419478	337.4740831	563.2842783	556.5579011
Democratic People's Republic of Korea	3479.014445	3644.351304	337.3249405	351.3055215	563.4913027	574.5696506
Taiwan (Province of China)	3994.354316	3914.046822	389.1043203	379.1216768	638.2143158	620.3370531
Oceania	3545.011564	3907.297429	341.956746	371.2097771	616.5595841	611.1767029
American Samoa	3578.112365	3928.317743	347.239031	375.3582385	611.5214923	612.1032199
Cook Islands	3722.428431	3899.136682	360.2319237	373.5711725	618.6900901	609.8772878
Fiji	3691.882903	3927.765949	357.2202327	374.3817362	630.2950206	611.5233567
Guam	3763.267688	3878.764499	365.6111642	373.0026685	613.7861688	606.6918226
Kiribati	3585.530931	3977.061179	344.6581996	376.8341895	613.2846688	615.176775
Marshall Islands	3354.819642	3916.695615	326.515379	373.4499952	620.2500326	610.3477291
Micronesia (Federated States of)	3469.678561	3935.191229	336.2804595	375.1988181	620.6693369	612.7083623
Nauru	3395.846836	3912.044238	330.5747552	374.5814045	605.8766522	613.6989173
Niue	3760.503077	3949.802502	360.254085	377.568467	610.1636907	612.8209991
Northern Mariana Islands	3839.787511	3858.363376	373.8754118	370.8512029	626.0644085	610.229244

Palau	3861.28693	3921.430184	373.149681	375.0247354	625.2244361	610.5718564
Papua New Guinea	3519.429814	3902.068661	338.689393	369.7613186	613.9636034	611.1877255
Samoa	3646.22504	3905.236448	354.2648948	373.8920574	632.4151037	609.5885915
Solomon Islands	3382.977399	3888.352602	328.7371794	371.5245712	609.3367526	610.9659191
Tokelau	3514.977722	3976.42541	338.0255292	380.4366418	586.8694931	614.7422848
Tonga	3644.417841	3956.937876	353.5108719	378.8301859	627.7587757	614.2958948
Tuvalu	3740.840917	4021.92333	359.9306856	384.3229829	608.9376478	617.8401073
Vanuatu	3424.398814	3903.248269	331.2302386	371.8596614	609.0984827	612.5290284
Southeast Asia	3193.490862	3470.05613	307.6891922	330.3425891	555.0592233	554.4560686
Cambodia	3609.059507	4427.379091	346.3060544	418.8175728	622.1886046	653.7383989
Indonesia	3141.226209	3398.063783	302.5371015	322.8643956	551.5820158	549.285245
Lao People's Democratic Republic	3579.120339	4229.666783	344.5694182	401.9971211	617.3999983	639.8802233
Malaysia	4026.839795	4427.407385	388.9008822	422.3806893	660.455591	658.2875253
Maldives	2949.998877	3592.24056	285.8818706	343.1499814	544.4002924	574.8804377
Mauritania	2411.712213	2729.957396	233.9948926	261.9819278	448.4252596	457.7730358
Myanmar	3413.754015	3784.325296	328.3835265	359.8622947	584.2034242	590.0630321
Philippines	3915.269922	4470.290697	376.7454652	424.178624	665.4964046	678.5692172
Seychelles	3518.657449	3672.449739	339.6266777	352.2657707	578.0951965	576.2684272
Sri Lanka	3672.695907	3803.307999	353.727388	363.0849697	608.5902912	592.4389514
Thailand	3237.506277	3267.376587	312.3483129	312.1532103	551.5778032	530.4642816
Timor-Leste	3067.819997	3635.601991	295.769345	345.2933013	549.6197493	573.7675716
Viet Nam	2061.510646	2271.03754	199.3983418	217.564108	391.1258629	398.2651639
Sub-Saharan Africa	2939.447679	3435.413849	282.1561491	325.6237372	525.6831115	544.4484412
Central Sub-Saharan Africa	3240.150311	3865.358262	308.7531777	363.4809011	577.1976329	604.6584093
Angola	3337.623423	3947.545193	320.226061	373.8350064	591.822078	615.0856486
Central African Republic	3586.824097	4196.239082	341.3187343	394.0299944	619.0909072	639.9036022
Congo	3211.957059	3728.009646	308.1684603	352.7486134	573.5554448	588.8428629

Democratic Republic of the Congo	3187.157606	3825.407919	302.9903677	358.8353802	570.3847899	600.1443604
Equatorial Guinea	3146.44762	3857.197435	298.6190623	361.5534381	561.0367389	603.1000482
Gabon	3439.064458	3918.704145	329.3670888	371.5727214	590.2229371	609.8860558
Eastern Sub-Saharan Africa	3030.233247	3694.411724	290.4923026	349.3903155	539.1498475	571.1193105
Burundi	2914.889609	3568.507529	279.7983839	338.2218607	517.047056	553.564146
Comoros	2907.533251	3464.445633	280.1560486	329.9084122	518.0291318	544.0585711
Djibouti	2725.740707	3215.538169	264.863013	307.7741467	497.5058165	514.5611109
Eritrea	3041.03934	3689.315058	289.4909959	346.4005733	542.0597462	565.3063675
Ethiopia	2971.527689	3635.471615	285.4627946	344.5339831	534.9135495	571.039096
Kenya	2735.074876	3369.208716	264.4879744	320.5794055	509.5142076	543.4885834
Madagascar	3187.291575	3819.986949	306.2012383	362.5160754	555.8108457	583.4549302
Malawi	3059.671281	3694.375849	292.739376	348.9871638	538.4640932	567.8910024
Mozambique	3290.871641	3962.340838	313.6028252	373.1928961	566.4361533	595.7048667
Rwanda	2896.158273	3527.106419	277.853296	333.9064617	518.5744405	549.299064
Somalia	2731.915604	3333.052319	262.2569033	315.6872405	502.661178	528.2917557
South Sudan	4021.349831	4733.887668	384.5506312	445.5200126	657.4611194	670.1198606
Uganda	3110.360218	3879.358972	296.7447188	364.6902828	548.0812751	587.7301813
United Republic of Tanzania	3049.888828	3700.188687	290.9498431	348.7953095	538.4825044	568.94465
Zambia	3093.528454	3753.815953	296.9612589	355.4245853	548.9806474	575.0980734
Southern Sub-Saharan Africa	3339.812094	3641.735114	321.0047046	345.6756018	577.683718	577.1845651
Botswana	3106.574685	3510.917424	299.4743577	333.6358155	557.080661	557.6170108
Eswatini	2931.631893	3490.084304	284.7338284	333.164662	538.5406939	554.7611581
Lesotho	3117.766601	3492.596442	300.0243787	332.2139718	548.6737259	554.8872778
Namibia	3031.207456	3409.756768	291.5484014	323.8078015	537.4934856	546.7186842
South Africa	3559.669891	3775.131595	341.399702	357.9683108	602.3664889	594.6593809
Zimbabwe	2698.688914	3145.76151	261.5734042	300.3094667	505.9520215	518.5344034
Western Sub-Saharan Africa	2653.979422	3018.907382	255.6675226	287.5421883	483.3597128	492.6950385

Benin	2649.217706	3151.481332	255.8535885	300.3020936	492.7725003	508.2639726
Burkina Faso	2626.130342	3078.220429	252.2143796	292.2272381	489.0757333	499.5854157
Cabo Verde	2761.273074	3076.921484	268.6572898	296.7369193	493.1887023	499.8957595
Cameroon	2776.417852	3178.256066	267.0328666	301.8026126	504.4823565	512.4048528
Chad	2727.539874	3211.085449	263.434569	306.4544966	497.6634055	515.5894813
C??te d'Ivoire	2794.625744	3191.501386	268.753692	302.268928	510.0528024	514.399092
Gambia	2837.568793	3263.985201	274.4580037	311.7904195	515.1924593	522.6060339
Ghana	2646.594945	2961.369916	256.2124174	283.2473523	485.2628018	487.174837
Guinea	2864.357101	3316.960178	275.7351471	316.4034926	509.280225	528.4362596
Guinea-Bissau	2677.965732	3057.475156	258.7320601	291.4282703	493.0499765	497.5341259
Liberia	3023.831432	3419.236962	287.0495607	321.1845155	540.2872712	540.3737005
Mali	2262.462441	2633.105162	217.6988132	250.7948524	429.7157659	444.9587911
Mauritius	3748.725196	3795.989587	360.3358417	361.5818197	609.7678872	588.1390723
Niger	2370.730605	2813.859774	229.0944642	268.7255107	456.0836904	469.3260338
Nigeria	2637.096427	2957.419394	253.8884701	281.731962	475.3671019	484.6887236
Sao Tome and Principe	2723.032989	3070.492526	264.5964635	295.0346846	498.7357956	499.3490016
Senegal	2517.182238	2891.670989	243.051359	275.8535096	471.1952714	478.766725
Sierra Leone	3198.402177	3597.029843	307.0016814	342.0987613	550.8213559	558.6236698
Togo	3044.499525	3519.178253	294.5079396	335.8768661	549.8939309	550.8745248

Supplementary Materials S2 The crude and age-standardized prevalence, incidence, and DALYs rate of depression and anxiety of different gender from 1990 to 2019

Table S7 The crude and age-standardized prevalence, incidence, and DALYs rate of depression of different gender of world from 1990 to 2019

Year	DALY rate						Incidence rate						Prevalence rate					
	Both		Male		Female		Both		Male		Female		Both		Male		Female	
	AS	Crude	AS	Crude	AS	Crude	AS	Crude	AS	Crude	AS	Crude	AS	Crude	AS	Crude	AS	Crude
1990	588.57	543.74	726.32	680.06	449.36	409.33	3681.24	3405.39	4613.68	4330.63	2735.94	2493.09	3486.17	3192.57	4262.52	3961.70	2700.71	2434.19
1991	595.87	552.85	734.42	690.25	456.00	417.49	3733.30	3467.50	4672.14	4400.72	2782.60	2548.14	3520.83	3239.26	4301.42	4014.53	2731.95	2475.51
1992	602.11	561.02	741.31	699.43	461.72	424.78	3777.77	3522.90	4721.73	4463.33	2822.83	2597.20	3550.15	3281.31	4334.11	4062.26	2758.62	2512.59
1993	606.99	567.98	746.54	707.18	466.34	431.05	3812.64	3569.73	4759.75	4516.06	2855.22	2638.84	3573.19	3317.77	4359.04	4103.46	2780.24	2544.92
1994	610.35	573.53	749.98	713.36	469.65	436.05	3836.29	3606.41	4784.09	4556.84	2878.50	2671.86	3589.19	3347.90	4375.67	4137.50	2795.85	2571.50
1995	611.89	577.44	751.18	717.54	471.52	439.69	3846.74	3631.08	4791.95	4582.92	2891.53	2695.27	3596.67	3370.33	4381.70	4162.24	2804.73	2591.77
1996	611.84	579.87	750.42	719.98	472.15	442.13	3846.56	3646.20	4786.87	4597.75	2896.11	2710.69	3596.45	3385.70	4378.00	4178.30	2807.85	2606.45
1997	610.86	581.54	748.63	721.61	471.95	443.81	3840.61	3656.76	4775.33	4607.78	2895.68	2721.63	3590.88	3396.52	4367.65	4188.92	2806.95	2617.37
1998	609.45	582.94	746.37	722.98	471.36	445.20	3831.57	3665.47	4760.41	4615.98	2892.43	2730.55	3582.88	3405.85	4354.41	4197.81	2804.05	2626.88
1999	608.08	584.55	744.20	724.61	470.76	446.72	3822.58	3675.24	4745.85	4625.57	2888.89	2740.07	3575.57	3416.78	4342.36	4208.83	2801.30	2637.36
2000	607.23	586.81	742.75	727.08	470.47	448.71	3816.50	3688.49	4735.13	4639.41	2887.23	2752.26	3571.47	3431.71	4334.95	4225.21	2800.25	2650.47
2001	607.32	590.14	742.40	730.76	470.95	451.60	3816.11	3708.22	4731.22	4660.75	2890.19	2769.89	3572.63	3452.66	4334.25	4248.98	2802.98	2668.21
2002	607.97	594.16	742.68	735.20	471.96	455.16	3819.67	3732.57	4731.91	4687.23	2896.58	2791.63	3576.41	3476.96	4336.45	4276.29	2808.19	2689.13
2003	608.82	598.39	743.23	739.87	473.09	458.86	3824.19	3757.74	4733.98	4714.71	2903.50	2814.00	3581.08	3502.20	4339.85	4304.69	2813.98	2710.81
2004	609.41	602.28	743.55	744.19	473.93	462.26	3826.76	3780.35	4734.34	4739.48	2908.22	2833.93	3584.59	3525.95	4342.35	4331.57	2818.36	2731.02
2005	609.21	605.24	743.07	747.48	473.99	464.80	3824.02	3796.45	4729.33	4757.22	2907.60	2847.85	3584.35	3545.32	4341.06	4353.71	2818.99	2747.18
2006	605.00	603.89	737.82	745.61	470.79	463.90	3791.56	3780.79	4688.41	4736.23	2883.45	2836.99	3565.12	3544.24	4317.45	4351.51	2803.96	2746.81
2007	595.88	597.31	726.63	737.35	463.72	458.92	3722.93	3726.52	4602.84	4667.31	2831.68	2796.90	3522.32	3517.97	4265.56	4318.84	2770.14	2726.60
2008	585.18	588.88	713.48	726.82	455.44	452.53	3642.85	3659.09	4503.02	4582.08	2771.15	2746.72	3471.83	3482.78	4204.41	4275.40	2730.14	2699.29
2009	576.20	582.10	702.48	718.37	448.44	447.33	3575.67	3604.10	4419.18	4512.46	2720.37	2705.70	3429.49	3455.39	4153.30	4241.64	2696.30	2677.75
2010	572.12	580.32	697.47	716.06	445.24	445.98	3545.42	3586.82	4381.19	4489.95	2697.63	2692.98	3410.27	3451.66	4130.38	4236.85	2680.54	2674.55
2011	571.99	582.54	697.28	718.64	445.18	447.77	3544.55	3599.56	4379.24	4504.36	2697.83	2703.52	3409.92	3466.96	4130.46	4255.31	2679.68	2686.25
2012	572.43	585.23	697.72	721.72	445.63	450.00	3547.70	3615.94	4381.70	4522.70	2701.80	2717.50	3412.01	3484.16	4133.25	4275.81	2681.09	2699.77
2013	573.20	588.19	698.51	725.09	446.37	452.46	3553.50	3634.79	4387.15	4543.85	2708.06	2733.59	3415.63	3502.50	4137.84	4297.70	2683.73	2714.18
2014	574.17	591.35	699.56	728.72	447.29	455.10	3560.51	3655.08	4394.14	4566.78	2715.18	2750.80	3420.46	3522.08	4143.70	4320.86	2687.57	2729.81
2015	575.12	594.46	700.55	732.26	448.21	457.73	3567.05	3674.74	4400.94	4589.31	2721.65	2767.22	3425.54	3541.72	4149.47	4343.69	2692.05	2745.93
2016	575.67	597.05	701.15	735.24	448.76	459.88	3570.87	3691.17	4405.02	4608.46	2725.50	2780.66	3429.36	3559.33	4154.04	4364.38	2695.33	2760.22
2017	576.23	599.71	701.67	738.20	449.42	462.19	3574.94	3708.50	4409.13	4628.37	2729.87	2795.11	3433.32	3577.41	4158.33	4385.16	2699.21	2775.36
2018	577.02	602.73	702.15	741.27	450.56	465.12	3581.29	3729.11	4413.67	4649.77	2738.45	2814.60	3437.48	3596.44	4160.74	4404.72	2705.38	2793.55
2019	577.75	605.67	702.08	743.70	452.17	468.52	3588.25	3750.40	4416.34	4669.07	2750.27	2837.52	3440.05	3613.67	4158.38	4418.92	2713.27	2813.50

Table S8 The crude and age-standardized prevalence, incidence, and DALYs rate of depression of different gender of Central Europe, Eastern Europe, and Central Asia from 1990 to 2019

Year	DALY rate						Incidence rate						Prevalence rate					
	Both		Male		Female		Both		Male		Female		Both		Male		Female	
	AS	Crude	AS	Crude	AS	Crude	AS	Crude	AS	Crude	AS	Crude	AS	Crude	AS	Crude	AS	Crude
1990	557.55	592.54	666.67	735.30	427.80	436.33	3521.70	3741.94	4278.18	4745.86	2616.27	2643.38	3307.32	3519.06	3891.72	4299.91	2612.27	2664.59
1991	560.02	597.23	670.57	741.44	429.28	439.54	3539.49	3775.28	4306.19	4789.66	2627.12	2666.05	3318.56	3543.64	3909.61	4331.05	2619.23	2682.61
1992	561.99	602.01	673.22	747.20	430.98	443.29	3553.71	3808.80	4325.65	4830.69	2638.95	2691.58	3327.55	3569.27	3922.04	4361.45	2626.89	2703.19
1993	563.53	606.45	674.99	752.34	432.50	446.89	3564.92	3839.32	4338.14	4865.84	2650.46	2716.66	3334.76	3593.81	3930.06	4389.21	2634.37	2723.93
1994	564.56	610.38	675.86	756.56	433.80	450.40	3572.02	3865.66	4343.97	4893.96	2659.79	2740.32	3339.28	3615.72	3933.68	4412.50	2640.37	2743.73
1995	564.91	613.94	675.77	760.10	434.65	453.84	3573.87	3888.55	4342.15	4915.75	2665.92	2763.41	3340.37	3635.99	3932.45	4432.60	2644.14	2763.42
1996	564.50	617.14	673.97	762.41	435.76	457.92	3571.42	3910.00	4329.44	4930.55	2674.63	2791.47	3338.56	3655.72	3923.94	4448.28	2649.76	2787.07
1997	563.66	620.32	670.61	763.88	437.58	462.95	3565.42	3930.67	4304.81	4938.29	2688.42	2826.11	3334.48	3675.48	3907.89	4460.15	2658.54	2815.32
1998	562.43	623.26	666.43	764.73	439.41	468.18	3557.53	3949.96	4275.17	4942.00	2703.21	2862.48	3328.91	3694.75	3888.00	4469.43	2668.06	2845.55
1999	560.94	626.09	662.33	765.64	440.70	473.09	3547.51	3968.11	4245.66	4945.72	2713.90	2896.30	3321.88	3713.56	3868.49	4479.31	2674.47	2874.02
2000	559.13	628.63	658.83	766.94	440.80	476.95	3534.34	3983.00	4220.42	4951.55	2714.39	2920.76	3312.93	3730.62	3851.46	4490.66	2674.74	2897.05
2001	556.16	629.72	654.79	767.15	439.27	478.92	3512.00	3986.63	4190.44	4948.76	2702.45	2930.89	3298.15	3740.31	3831.85	4496.75	2666.68	2910.26
2002	551.81	628.96	649.21	765.25	436.65	479.33	3477.79	3974.60	4147.61	4928.62	2680.53	2927.24	3276.07	3739.99	3804.05	4491.43	2652.84	2915.04
2003	546.76	626.81	642.93	761.90	433.25	478.44	3438.52	3952.05	4099.76	4897.29	2653.06	2913.83	3250.60	3732.66	3773.00	4478.83	2635.09	2913.09
2004	541.58	624.22	636.67	758.15	429.56	477.03	3399.19	3927.29	4053.13	4864.70	2623.94	2897.05	3224.68	3723.21	3742.32	4464.55	2615.91	2908.45
2005	537.16	622.35	631.51	755.58	426.15	475.82	3365.09	3907.67	4014.14	4840.24	2596.75	2881.97	3202.50	3717.27	3717.01	4455.86	2598.24	2904.92
2006	532.54	619.98	626.49	752.95	422.15	473.65	3329.68	3884.72	3976.27	4815.81	2565.46	2860.08	3179.52	3708.77	3692.30	4446.56	2578.16	2896.84
2007	526.84	615.88	620.54	748.81	416.97	469.55	3286.86	3850.11	3932.00	4781.27	2525.96	2825.12	3151.43	3691.62	3663.38	4429.76	2552.26	2879.09
2008	521.20	611.11	614.70	744.06	411.71	464.73	3244.22	3810.69	3888.01	4742.21	2485.85	2785.13	3123.89	3670.99	3634.97	4409.56	2526.46	2857.86
2009	516.45	606.94	609.65	739.76	407.45	460.72	3208.01	3776.12	3850.00	4706.89	2452.96	2751.51	3100.50	3652.79	3610.53	4391.17	2505.22	2839.96
2010	513.37	604.48	606.11	736.84	405.17	458.82	3184.85	3755.67	3823.79	4683.52	2435.12	2734.64	3085.19	3642.75	3593.22	4379.28	2493.58	2832.26
2011	512.37	603.97	605.03	736.05	404.70	458.70	3175.17	3748.23	3813.43	4673.80	2429.55	2730.23	3079.35	3641.26	3587.02	4376.39	2490.57	2832.73
2012	512.30	603.98	605.62	736.26	404.58	458.59	3172.66	3745.82	3815.05	4672.24	2427.37	2727.63	3077.99	3641.89	3588.85	4377.91	2489.19	2832.97
2013	512.85	604.28	607.04	736.90	404.84	458.63	3174.36	3745.53	3822.98	4674.43	2426.92	2725.42	3079.49	3643.44	3594.54	4381.03	2489.44	2833.43
2014	513.41	604.43	608.40	737.36	405.11	458.58	3176.72	3745.31	3831.25	4676.50	2427.05	2723.53	3081.24	3644.29	3600.26	4383.43	2489.85	2833.24
2015	513.46	604.04	608.92	737.12	405.07	458.12	3176.09	3742.54	3833.55	4674.36	2426.29	2720.82	3080.76	3642.66	3602.02	4382.44	2489.16	2831.50
2016	513.41	603.67	608.66	736.25	405.63	458.40	3175.06	3740.70	3830.91	4669.25	2429.49	2723.28	3080.23	3641.55	3600.71	4379.25	2491.41	2833.25
2017	513.25	603.37	608.20	735.46	406.19	458.76	3174.13	3741.01	3827.89	4666.66	2433.58	2727.56	3079.77	3641.62	3599.08	4377.27	2494.33	2836.19
2018	513.21	603.60	608.33	736.11	406.25	458.64	3174.20	3745.27	3829.61	4674.81	2433.98	2728.43	3079.84	3644.22	3600.23	4382.11	2494.82	2837.03
2019	513.51	604.64	609.29	738.36	406.10	458.49	3176.93	3755.74	3837.17	4694.41	2433.44	2729.79	3081.42	3650.77	3605.13	4394.59	2494.29	2837.79

Table S9 The crude and age-standardized prevalence, incidence, and DALYs rate of depression of different gender of High-income from 1990 to 2019

Year	DALY rate						Incidence rate						Prevalence rate					
	Both		Male		Female		Both		Male		Female		Both		Male		Female	
	AS	Crude	AS	Crude	AS	Crude	AS	Crude	AS	Crude	AS	Crude	AS	Crude	AS	Crude	AS	Crude
1990	606.72	664.71	751.95	826.08	456.28	496.27	3842.26	4199.61	4841.73	5320.88	2801.67	3029.30	3504.75	3861.52	4300.55	4758.35	2679.97	2901.50
1991	609.03	668.12	756.30	831.13	456.93	498.05	3855.90	4219.11	4868.93	5351.20	2804.46	3038.05	3514.99	3879.49	4319.87	4783.39	2683.06	2955.19
1992	612.09	672.20	761.41	836.89	458.26	500.47	3875.86	4244.55	4903.23	5388.38	2812.54	3051.75	3528.42	3900.27	4342.69	4811.60	2688.87	3012.71
1993	615.68	676.77	766.96	843.09	460.16	503.40	3900.89	4274.94	4942.46	5430.63	2825.56	3070.21	3544.37	3923.40	4367.60	4842.01	2697.43	3060.62
1994	619.65	681.80	772.68	849.60	462.65	506.94	3929.97	4309.79	4984.74	5476.78	2843.35	3093.70	3562.42	3949.01	4393.69	4874.38	2708.80	3084.05
1995	623.85	687.14	778.33	856.18	465.61	511.03	3961.31	4347.91	5027.48	5525.01	2864.94	3121.69	3581.65	3976.43	4419.60	4907.54	2722.57	3083.21
1996	629.90	694.16	786.24	864.81	470.00	516.46	4012.42	4405.43	5094.65	5596.82	2901.50	3164.79	3604.96	4006.62	4450.80	4944.48	2739.09	3071.19
1997	638.47	703.33	797.62	876.30	475.98	523.28	4088.87	4486.74	5195.38	5699.34	2955.19	3224.52	3632.93	4038.98	4488.80	4984.71	2758.15	3054.24
1998	647.61	712.92	809.82	888.40	482.29	530.34	4171.93	4574.05	5305.62	5810.24	3012.71	3287.86	3661.03	4070.46	4527.91	5024.64	2776.44	3038.42
1999	655.38	721.11	820.33	898.82	487.52	536.28	4242.65	4648.76	5400.58	5906.08	3060.62	3341.04	3685.20	4098.11	4562.18	5059.98	2791.59	3029.54
2000	659.66	726.04	826.48	905.30	490.16	539.67	4280.10	4691.05	5453.50	5962.22	3084.05	3369.47	3699.55	4117.48	4584.02	5085.35	2799.53	3028.60
2001	660.71	727.76	828.84	908.15	490.10	540.30	4287.59	4702.82	5470.88	5982.84	3083.21	3372.62	3703.54	4126.86	4593.90	5099.63	2798.75	3030.70
2002	660.31	727.69	829.90	909.27	488.46	539.07	4284.47	4701.69	5478.31	5990.49	3071.19	3362.87	3700.67	4127.64	4597.66	5105.62	2790.41	3034.36
2003	659.15	726.69	830.14	909.36	486.09	536.98	4276.09	4694.06	5480.08	5990.84	3054.24	3347.36	3694.20	4123.94	4597.53	5106.68	2778.67	3038.72
2004	657.98	725.73	830.27	909.44	483.81	535.01	4267.47	4686.41	5480.12	5989.99	3038.42	3333.07	3688.06	4120.81	4597.19	5107.99	2767.73	3041.57
2005	657.45	725.64	830.59	910.06	482.59	534.24	4263.15	4684.90	5481.56	5993.36	3029.54	3326.87	3685.16	4122.16	4598.31	5112.63	2761.66	3035.68
2006	657.83	726.68	831.77	911.95	482.28	534.44	4266.93	4693.21	5490.85	6007.76	3028.60	3329.23	3686.10	4128.37	4602.96	5122.92	2759.43	3018.95
2007	658.81	728.19	833.84	914.71	482.24	534.71	4276.32	4706.87	5508.06	6030.57	3030.70	3333.75	3688.35	4135.30	4609.87	5135.59	2757.37	2998.05
2008	660.08	729.95	836.16	917.68	482.53	535.25	4287.88	4722.24	5528.03	6055.62	3034.36	3339.44	3691.74	4143.01	4617.77	5148.98	2756.53	2979.61
2009	661.24	731.48	838.25	920.24	482.79	535.73	4298.84	4736.28	5545.90	6077.28	3038.72	3345.61	3695.12	4150.33	4624.96	5160.84	2756.28	2969.80
2010	661.87	732.23	839.29	921.30	483.05	536.15	4304.89	4743.69	5555.62	6087.73	3041.57	3349.78	3697.10	4154.98	4628.83	5166.95	2756.64	2961.36
2011	660.58	730.64	837.49	918.69	482.38	535.60	4292.93	4729.59	5538.45	6065.12	3035.68	3344.45	3693.77	4152.50	4624.32	5161.00	2755.07	2955.93
2012	657.06	726.36	832.43	911.98	480.61	533.87	4260.69	4691.91	5492.17	6007.40	3018.95	3327.85	3683.46	4141.36	4609.19	5141.02	2750.60	2965.43
2013	652.62	721.00	826.05	903.69	478.37	531.64	4220.89	4645.63	5435.29	5937.07	2998.05	3307.06	3670.00	4126.33	4589.83	5115.57	2744.31	2985.70
2014	648.75	716.32	820.64	896.56	476.29	529.61	4186.31	4605.74	5386.45	5876.72	2979.61	3289.07	3658.23	4113.29	4573.41	5093.64	2738.57	3029.30
2015	646.77	713.92	818.06	892.93	475.17	528.59	4169.41	4586.69	5364.22	5848.45	2969.80	3280.35	3651.72	4106.85	4565.45	5082.83	2734.83	3038.05
2016	645.91	712.85	818.04	892.14	473.74	527.33	4164.60	4581.38	5364.98	5845.05	2961.36	3273.89	3648.52	4104.39	4566.54	5081.98	2728.77	3051.75
2017	645.39	712.21	818.17	891.61	472.82	526.69	4162.31	4579.66	5367.71	5844.75	2955.93	3271.38	3647.09	4104.28	4568.33	5082.38	2725.49	3070.21
2018	645.85	712.62	818.08	891.08	474.05	528.15	4167.67	4587.13	5370.31	5847.99	2965.43	3283.78	3651.11	4110.04	4570.18	5084.14	2732.76	3093.70
2019	647.16	713.71	818.00	890.39	476.92	531.15	4179.27	4600.45	5374.34	5852.84	2985.70	3306.29	3659.91	4119.72	4572.14	5085.08	2749.29	3121.69

Table S10 The crude and age-standardized prevalence, incidence, and DALYs rate of depression of different gender of Latin America and Caribbean from 1990 to 2019

Year	DALY rate						Incidence rate						Prevalence rate					
	Both		Male		Female		Both		Male		Female		Both		Male		Female	
	AS	Crude	AS	Crude	AS	Crude	AS	Crude	AS	Crude	AS	Crude	AS	Crude	AS	Crude	AS	Crude
1990	619.40	531.58	801.70	702.88	427.74	356.42	4091.36	3509.29	5414.55	4747.98	2700.57	2242.66	3485.60	2954.69	4418.26	3826.92	2504.20	2062.79
1991	618.14	534.11	799.49	705.95	427.14	358.24	4080.60	3523.06	5396.73	4765.12	2694.64	2251.83	3478.80	2970.09	4406.78	3845.61	2500.47	2074.01
1992	617.64	537.39	798.28	709.95	427.09	360.63	4075.47	3542.45	5386.21	4789.16	2692.94	2265.41	3475.39	2988.93	4399.90	3868.64	2499.10	2087.81
1993	617.66	541.19	797.80	714.61	427.38	363.41	4074.74	3566.21	5381.47	4818.49	2694.54	2282.49	3474.45	3010.35	4396.11	3894.59	2499.76	2103.91
1994	618.13	545.48	797.87	719.80	428.03	366.65	4077.26	3593.40	5381.09	4851.95	2698.46	2302.35	3476.01	3034.41	4395.42	3923.46	2502.46	2122.39
1995	618.86	550.10	798.32	725.40	428.87	370.15	4081.90	3623.04	5383.76	4888.35	2703.78	2324.22	3478.99	3060.16	4396.98	3954.61	2505.90	2142.01
1996	623.53	558.43	802.37	734.38	434.01	377.70	4115.39	3680.14	5412.84	4950.05	2740.66	2375.76	3500.92	3103.82	4415.83	4002.18	2530.15	2181.08
1997	633.42	571.76	811.21	747.98	444.89	390.65	4188.16	3774.99	5477.81	5046.53	2820.73	2468.17	3548.18	3171.70	4458.01	4072.20	2582.04	2246.22
1998	645.32	587.17	822.03	763.63	457.83	405.73	4274.86	3884.43	5556.20	5157.15	2915.53	2575.69	3604.91	3249.39	4509.77	4152.12	2643.47	2321.12
1999	655.62	601.31	831.51	778.23	468.91	419.30	4350.07	3984.60	5625.46	5260.69	2996.53	2671.76	3653.92	3321.03	4554.81	4226.87	2696.23	2389.11
2000	660.81	610.83	836.52	788.82	474.25	427.64	4388.33	4050.94	5663.13	5335.39	3035.05	2729.02	3678.88	3371.13	4579.13	4283.23	2721.55	2432.41
2001	662.51	617.26	838.83	797.11	475.28	432.12	4400.86	4094.29	5680.60	5392.60	3042.24	2757.77	3686.59	3406.18	4590.07	4328.37	2725.71	2456.86
2002	664.09	623.78	841.45	805.94	475.79	436.22	4414.12	4139.55	5701.57	5454.64	3047.56	2785.44	3693.68	3441.68	4602.16	4375.81	2727.55	2479.84
2003	665.47	630.15	843.97	814.70	476.03	440.08	4425.39	4183.13	5721.77	5516.16	3049.74	2810.26	3699.30	3476.02	4613.84	4423.09	2726.93	2500.64
2004	666.29	635.90	846.05	822.95	475.56	443.22	4431.89	4221.68	5736.86	5572.02	3047.55	2830.65	3702.22	3507.50	4622.84	4467.45	2723.61	2518.61
2005	666.17	640.59	846.85	829.73	474.50	445.69	4431.12	4252.10	5742.79	5617.49	3039.92	2845.17	3701.13	3534.38	4626.15	4505.27	2717.99	2533.95
2006	661.31	640.34	841.84	830.39	469.83	444.45	4395.33	4246.37	5705.22	5617.56	3006.12	2833.00	3677.56	3538.09	4601.43	4513.92	2695.67	2532.26
2007	650.71	634.13	829.90	823.79	460.64	438.57	4316.34	4195.86	5614.51	5562.02	2939.53	2787.15	3625.94	3512.89	4542.62	4487.04	2651.66	2508.40
2008	637.83	625.43	815.20	814.13	449.71	430.78	4220.93	4127.38	5503.75	5484.25	2860.34	2727.62	3563.34	3475.67	4470.35	4445.21	2599.28	2475.49
2009	626.38	618.01	802.24	806.02	439.85	423.95	4135.62	4067.96	5405.60	5417.87	2788.63	2674.66	3507.47	3444.28	4406.22	4410.48	2552.12	2447.03
2010	619.80	615.46	795.23	803.93	433.73	420.80	4086.76	4045.09	5352.43	5397.01	2744.29	2648.86	3475.62	3436.77	4371.61	4405.36	2523.13	2436.42
2011	617.07	616.84	792.76	806.47	430.75	420.87	4065.32	4050.49	5332.16	5409.85	2721.88	2645.70	3462.44	3448.24	4359.54	4423.07	2508.90	2440.82
2012	614.63	618.47	790.38	808.96	428.32	421.50	4045.25	4057.02	5311.44	5421.32	2703.04	2646.33	3450.94	3461.13	4347.97	4440.57	2497.78	2448.39
2013	612.52	620.27	788.05	811.25	426.51	422.68	4026.97	4064.29	5290.48	5430.77	2688.18	2650.48	3440.88	3474.66	4336.70	4457.07	2489.35	2458.23
2014	610.72	622.21	785.82	813.37	425.26	424.32	4011.55	4073.18	5270.57	5439.45	2678.17	2658.79	3432.40	3488.88	4325.89	4472.69	2483.75	2470.41
2015	609.21	624.27	783.49	815.16	424.71	426.54	3999.77	4084.55	5252.59	5448.59	2673.54	2671.65	3425.34	3503.66	4314.64	4486.73	2481.48	2485.38
2016	607.51	626.06	780.01	815.62	424.96	429.53	3987.89	4095.44	5227.31	5449.54	2676.30	2691.67	3418.53	3518.48	4299.14	4495.91	2484.18	2505.19
2017	606.36	628.39	777.42	816.97	425.37	432.70	3980.01	4110.47	5209.00	5457.62	2679.73	2712.52	3414.61	3536.23	4288.44	4509.92	2487.61	2525.83
2018	606.51	631.99	777.36	820.90	425.76	435.79	3980.48	4133.77	5208.31	5483.82	2681.61	2731.69	3415.01	3558.04	4287.83	4534.05	2489.19	2544.41
2019	607.23	636.09	778.07	825.53	426.53	439.20	3983.79	4159.45	5211.58	5513.59	2685.13	2752.13	3417.06	3580.98	4289.85	4560.29	2491.41	2563.20

Table S11 The crude and age-standardized prevalence, incidence, and DALYs rate of depression of different gender of North Africa and Middle East from 1990 to 2019

Year	DALY rate						Incidence rate						Prevalence rate					
	Both		Male		Female		Both		Male		Female		Both		Male		Female	
	AS	Crude	AS	Crude	AS	Crude	AS	Crude	AS	Crude	AS	Crude	AS	Crude	AS	Crude	AS	Crude
1990	619.40	531.58	801.70	702.88	427.74	356.42	4091.36	3509.29	5414.55	4747.98	2700.57	2242.66	3485.60	2954.69	4418.26	3826.92	2504.20	2062.79
1991	618.14	534.11	799.49	705.95	427.14	358.24	4080.60	3523.06	5396.73	4765.12	2694.64	2251.83	3478.80	2970.09	4406.78	3845.61	2500.47	2074.01
1992	617.64	537.39	798.28	709.95	427.09	360.63	4075.47	3542.45	5386.21	4789.16	2692.94	2265.41	3475.39	2988.93	4399.90	3868.64	2499.10	2087.81
1993	617.66	541.19	797.80	714.61	427.38	363.41	4074.74	3566.21	5381.47	4818.49	2694.54	2282.49	3474.45	3010.35	4396.11	3894.59	2499.76	2103.91
1994	618.13	545.48	797.87	719.80	428.03	366.65	4077.26	3593.40	5381.09	4851.95	2698.46	2302.35	3476.01	3034.41	4395.42	3923.46	2502.46	2122.39
1995	618.86	550.10	798.32	725.40	428.87	370.15	4081.90	3623.04	5383.76	4888.35	2703.78	2324.22	3478.99	3060.16	4396.98	3954.61	2505.90	2142.01
1996	623.53	558.43	802.37	734.38	434.01	377.70	4115.39	3680.14	5412.84	4950.05	2740.66	2375.76	3500.92	3103.82	4415.83	4002.18	2530.15	2181.08
1997	633.42	571.76	811.21	747.98	444.89	390.65	4188.16	3774.99	5477.81	5046.53	2820.73	2468.17	3548.18	3171.70	4458.01	4072.20	2582.04	2246.22
1998	645.32	587.17	822.03	763.63	457.83	405.73	4274.86	3884.43	5556.20	5157.15	2915.53	2575.69	3604.91	3249.39	4509.77	4152.12	2643.47	2321.12
1999	655.62	601.31	831.51	778.23	468.91	419.30	4350.07	3984.60	5625.46	5260.69	2996.53	2671.76	3653.92	3321.03	4554.81	4226.87	2696.23	2389.11
2000	660.81	610.83	836.52	788.82	474.25	427.64	4388.33	4050.94	5663.13	5335.39	3035.05	2729.02	3678.88	3371.13	4579.13	4283.23	2721.55	2432.41
2001	662.51	617.26	838.83	797.11	475.28	432.12	4400.86	4094.29	5680.60	5392.60	3042.24	2757.77	3686.59	3406.18	4590.07	4328.37	2725.71	2456.86
2002	664.09	623.78	841.45	805.94	475.79	436.22	4414.12	4139.55	5701.57	5454.64	3047.56	2785.44	3693.68	3441.68	4602.16	4375.81	2727.55	2479.84
2003	665.47	630.15	843.97	814.70	476.03	440.08	4425.39	4183.13	5721.77	5516.16	3049.74	2810.26	3699.30	3476.02	4613.84	4423.09	2726.93	2500.64
2004	666.29	635.90	846.05	822.95	475.56	443.22	4431.89	4221.68	5736.86	5572.02	3047.55	2830.65	3702.22	3507.50	4622.84	4467.45	2723.61	2518.61
2005	666.17	640.59	846.85	829.73	474.50	445.69	4431.12	4252.10	5742.79	5617.49	3039.92	2845.17	3701.13	3534.38	4626.15	4505.27	2717.99	2533.95
2006	661.31	640.34	841.84	830.39	469.83	444.45	4395.33	4246.37	5705.22	5617.56	3006.12	2833.00	3677.56	3538.09	4601.43	4513.92	2695.67	2532.26
2007	650.71	634.13	829.90	823.79	460.64	438.57	4316.34	4195.86	5614.51	5562.02	2939.53	2787.15	3625.94	3512.89	4542.62	4487.04	2651.66	2508.40
2008	637.83	625.43	815.20	814.13	449.71	430.78	4220.93	4127.38	5503.75	5484.25	2860.34	2727.62	3563.34	3475.67	4470.35	4445.21	2599.28	2475.49
2009	626.38	618.01	802.24	806.02	439.85	423.95	4135.62	4067.96	5405.60	5417.87	2788.63	2674.66	3507.47	3444.28	4406.22	4410.48	2552.12	2447.03
2010	619.80	615.46	795.23	803.93	433.73	420.80	4086.76	4045.09	5352.43	5397.01	2744.29	2648.86	3475.62	3436.77	4371.61	4405.36	2523.13	2436.42
2011	617.07	616.84	792.76	806.47	430.75	420.87	4065.32	4050.49	5332.16	5409.85	2721.88	2645.70	3462.44	3448.24	4359.54	4423.07	2508.90	2440.82
2012	614.63	618.47	790.38	808.96	428.32	421.50	4045.25	4057.02	5311.44	5421.32	2703.04	2646.33	3450.94	3461.13	4347.97	4440.57	2497.78	2448.39
2013	612.52	620.27	788.05	811.25	426.51	422.68	4026.97	4064.29	5290.48	5430.77	2688.18	2650.48	3440.88	3474.66	4336.70	4457.07	2489.35	2458.23
2014	610.72	622.21	785.82	813.37	425.26	424.32	4011.55	4073.18	5270.57	5439.45	2678.17	2658.79	3432.40	3488.88	4325.89	4472.69	2483.75	2470.41
2015	609.21	624.27	783.49	815.16	424.71	426.54	3999.77	4084.55	5252.59	5448.59	2673.54	2671.65	3425.34	3503.66	4314.64	4486.73	2481.48	2485.38
2016	607.51	626.06	780.01	815.62	424.96	429.53	3987.89	4095.44	5227.31	5449.54	2676.30	2691.67	3418.53	3518.48	4299.14	4495.91	2484.18	2505.19
2017	606.36	628.39	777.42	816.97	425.37	432.70	3980.01	4110.47	5209.00	5457.62	2679.73	2712.52	3414.61	3536.23	4288.44	4509.92	2487.61	2525.83
2018	606.51	631.99	777.36	820.90	425.76	435.79	3980.48	4133.77	5208.31	5483.82	2681.61	2731.69	3415.01	3558.04	4287.83	4534.05	2489.19	2544.41
2019	607.23	636.09	778.07	825.53	426.53	439.20	3983.79	4159.45	5211.58	5513.59	2685.13	2752.13	3417.06	3580.98	4289.85	4560.29	2491.41	2563.20

Table S12 The crude and age-standardized prevalence, incidence, and DALYs rate of depression of different gender of South Asia from 1990 to 2019

Year	DALY rate						Incidence rate						Prevalence rate					
	Both		Male		Female		Both		Male		Female		Both		Male		Female	
	AS	Crude	AS	Crude	AS	Crude	AS	Crude	AS	Crude	AS	Crude	AS	Crude	AS	Crude	AS	Crude
1990	704.15	559.88	857.52	683.57	563.94	445.65	4653.99	3682.40	5760.39	4569.59	3643.03	2863.07	4078.71	3208.92	4949.15	3905.75	3282.83	2565.39
1991	727.45	583.48	892.10	717.63	576.72	459.64	4824.62	3854.21	6018.25	4821.59	3732.51	2961.18	4189.32	3323.44	5114.68	4071.70	3342.20	2632.70
1992	747.76	604.39	922.14	747.93	587.87	471.93	4974.04	4006.85	6241.73	5044.89	3812.23	3048.86	4285.66	3425.12	5257.72	4219.17	3394.32	2692.31
1993	763.97	621.67	945.53	772.65	597.14	482.35	5093.05	4132.37	6416.44	5227.16	3877.64	3122.12	4362.25	3509.44	5368.70	4340.08	3437.47	2742.95
1994	774.88	634.26	960.67	790.42	603.70	490.13	5172.53	4222.65	6528.09	5355.83	3924.20	3176.71	4413.45	3571.54	5439.74	4427.85	3467.87	2781.16
1995	779.11	640.89	965.55	799.34	606.78	494.57	5203.11	4269.11	6562.55	5417.78	3947.23	3208.31	4432.62	3605.20	5461.21	4473.25	3481.85	2803.54
1996	777.74	642.32	961.66	800.48	607.14	496.15	5192.23	4276.97	6531.55	5421.40	3950.65	3219.24	4425.51	3614.77	5441.00	4482.18	3483.52	2813.08
1997	773.75	641.12	953.82	797.81	606.12	496.15	5162.27	4265.55	6469.68	5395.65	3946.00	3219.95	4405.77	3611.81	5401.06	4472.60	3479.14	2815.40
1998	769.01	639.21	944.65	793.88	604.94	495.92	5125.92	4247.89	6396.80	5359.25	3939.59	3218.34	4382.16	3605.25	5354.19	4456.44	3473.97	2816.70
1999	765.03	638.25	936.48	791.01	604.37	496.54	5095.96	4237.69	6332.79	5331.85	3937.79	3222.69	4362.30	3603.46	5312.36	4445.64	3471.75	2822.22
2000	763.64	640.12	932.09	792.01	605.29	499.02	5085.44	4248.40	6297.33	5333.05	3947.11	3240.74	4355.03	3615.49	5289.16	4453.68	3476.58	2836.79
2001	766.34	646.02	932.79	797.82	609.36	504.77	5105.09	4288.57	6301.06	5370.88	3978.15	3281.41	4368.90	3648.25	5292.63	4486.72	3497.39	2868.00
2002	772.07	654.80	936.83	806.58	616.21	513.30	5146.04	4348.44	6330.14	5430.36	4026.94	3339.85	4397.98	3695.31	5313.19	4534.58	3531.86	2912.93
2003	778.73	664.62	942.25	816.61	623.61	522.66	5192.19	4414.27	6367.09	5496.89	4078.55	3403.08	4431.60	3747.59	5340.11	4588.38	3569.28	2962.27
2004	783.97	673.46	946.37	825.60	629.48	531.06	5227.31	4472.47	6394.44	5556.17	4117.91	3458.23	4458.11	3795.14	5360.94	4637.57	3598.75	3006.69
2005	785.36	679.15	946.76	831.45	631.43	536.31	5235.07	4508.58	6394.66	5592.96	4129.89	3491.60	4465.97	3827.76	5363.72	4671.89	3609.20	3036.09
2006	771.01	670.45	928.14	819.62	620.78	530.27	5124.09	4436.64	6249.64	5495.16	4048.62	3441.84	4399.84	3792.97	5278.14	4624.31	3559.49	3011.69
2007	738.55	644.75	887.02	786.33	596.25	511.42	4875.33	4235.54	5930.87	5231.92	3864.12	3297.17	4249.60	3679.16	5089.09	4478.19	3444.34	2926.66
2008	699.77	612.87	837.96	745.19	566.98	487.98	4580.49	3989.40	5554.12	4911.37	3645.14	3119.25	4069.60	3536.26	4863.15	4295.57	3306.35	2819.63
2009	666.93	586.41	796.42	711.05	542.15	468.52	4330.42	3783.83	5233.77	4643.01	3459.92	2971.20	3916.65	3418.52	4671.00	4144.83	3189.02	2731.55
2010	651.78	576.74	776.75	697.94	531.02	461.87	4215.19	3705.56	5083.10	4537.25	3376.28	2917.28	3845.78	3379.27	4579.41	4091.40	3136.11	2704.32
2011	650.00	579.99	773.38	700.75	530.47	465.31	4201.04	3724.47	5056.70	4552.28	3371.69	2938.36	3836.84	3400.63	4562.52	4111.63	3133.06	2725.43
2012	649.12	584.25	770.50	704.17	531.24	470.17	4193.13	3750.37	5034.38	4572.03	3375.69	2968.70	3832.13	3427.01	4548.00	4134.98	3136.27	2753.49
2013	648.78	589.14	768.27	708.29	532.50	475.61	4189.49	3780.94	5016.54	4596.55	3383.97	3003.77	3829.79	3456.29	4536.57	4161.86	3141.28	2783.96
2014	648.76	594.45	766.67	713.13	533.80	481.19	4187.86	3813.94	5003.34	4626.02	3391.95	3039.03	3829.23	3487.92	4528.21	4192.42	3147.04	2815.66
2015	648.56	599.71	765.57	718.60	534.30	486.13	4185.70	3847.20	4994.84	4660.78	3394.66	3069.92	3827.89	3519.53	4522.55	4226.23	3148.94	2844.35
2016	648.23	604.99	765.26	724.99	533.82	490.23	4183.21	3881.04	4992.49	4702.53	3391.03	3095.40	3826.41	3551.90	4521.20	4265.22	3146.54	2869.70
2017	647.92	610.45	765.01	731.63	533.32	494.45	4181.17	3916.30	4991.46	4746.79	3387.14	3121.27	3824.87	3585.19	4520.21	4305.75	3143.78	2895.41
2018	646.89	615.45	763.19	736.97	532.96	499.01	4179.56	3952.06	4986.35	4787.27	3388.17	3151.78	3814.95	3612.63	4503.30	4332.78	3140.07	2922.60
2019	645.08	619.79	759.56	740.55	532.85	503.98	4179.15	3988.39	4979.09	4824.97	3393.85	3186.11	3794.72	3631.50	4467.91	4342.48	3134.12	2949.67

Table S13 The crude and age-standardized prevalence, incidence, and DALYs rate of depression of different gender of Southeast Asia, East Asia, and Oceania from 1990 to 2019

Year	DALY rate						Incidence rate						Prevalence rate					
	Both		Male		Female		Both		Male		Female		Both		Male		Female	
	AS	Crude	AS	Crude	AS	Crude	AS	Crude	AS	Crude	AS	Crude	AS	Crude	AS	Crude	AS	Crude
1990	452.29	429.17	570.03	547.80	336.76	315.11	2516.51	2428.95	3225.18	3157.21	1818.52	1728.77	2911.75	2697.05	3633.49	3408.68	2201.67	2012.85
1991	460.36	438.91	573.89	553.47	348.92	328.94	2574.53	2489.45	3253.77	3186.65	1905.03	1820.19	2949.46	2750.57	3651.32	3445.28	2258.72	2083.70
1992	466.34	446.65	576.02	557.50	358.63	340.38	2617.03	2535.17	3269.21	3203.83	1973.78	1894.21	2976.95	2794.36	3660.53	3473.64	2304.06	2143.21
1993	470.13	452.38	576.37	559.97	365.75	349.36	2643.95	2566.88	3272.05	3210.62	2023.93	1950.48	2994.30	2829.23	3661.59	3495.48	2337.16	2191.27
1994	471.67	456.29	575.09	561.39	369.99	355.72	2654.69	2585.47	3262.29	3208.90	2054.47	1988.94	3001.10	2856.13	3654.94	3512.84	2357.01	2227.77
1995	470.99	458.42	572.09	561.71	371.50	359.61	2649.04	2591.25	3240.14	3199.73	2064.52	2009.19	2997.69	2875.02	3640.46	3525.51	2364.00	2252.77
1996	466.48	457.06	565.52	559.02	368.93	359.51	2614.70	2571.43	3191.42	3168.79	2043.89	1999.87	2977.60	2879.11	3610.18	3525.43	2353.46	2260.69
1997	458.23	452.05	555.25	552.93	362.61	355.47	2551.64	2524.16	3114.12	3112.14	1994.54	1961.22	2941.22	2867.07	3563.12	3509.73	2327.25	2251.77
1998	448.62	445.78	543.76	545.86	354.78	349.87	2478.09	2467.24	3027.23	3047.66	1933.75	1910.98	2898.92	2849.86	3510.32	3489.82	2294.82	2236.54
1999	439.91	440.69	533.43	540.29	347.57	345.11	2411.74	2418.58	2949.24	2993.31	1878.47	1867.08	2860.29	2838.31	3462.85	3477.53	2264.41	2224.93
2000	434.36	439.03	526.58	538.46	343.18	343.49	2370.42	2395.93	2898.74	2967.11	1845.55	1847.09	2835.32	2842.28	3430.92	3482.69	2245.53	2226.91
2001	431.98	440.98	523.29	540.62	341.56	345.10	2353.14	2399.19	2875.01	2969.32	1833.84	1850.62	2824.15	2862.75	3415.28	3506.89	2237.81	2242.96
2002	430.68	444.20	521.32	544.30	340.79	347.76	2343.94	2411.33	2861.00	2981.98	1828.65	1861.56	2818.20	2889.58	3406.00	3538.65	2234.28	2264.25
2003	429.88	447.98	519.90	548.60	340.46	350.94	2338.24	2426.99	2851.36	2998.85	1826.14	1875.42	2814.70	2919.33	3399.51	3573.69	2232.91	2288.19
2004	428.90	451.59	518.37	552.75	339.92	353.93	2331.33	2441.08	2840.60	3013.97	1822.48	1887.96	2810.31	2948.45	3392.45	3608.23	2230.47	2311.43
2005	427.06	454.17	515.82	555.66	338.68	356.08	2318.73	2448.14	2823.35	3021.03	1813.86	1894.50	2801.81	2972.51	3380.54	3636.93	2224.63	2330.41
2006	423.47	454.66	511.40	556.29	335.83	356.38	2293.57	2440.42	2791.67	3010.84	1794.68	1888.80	2784.46	2986.21	3359.04	3653.93	2210.74	2340.49
2007	418.24	453.10	505.18	554.59	331.50	354.91	2258.18	2419.89	2748.43	2986.01	1766.68	1872.18	2758.36	2989.03	3328.17	3658.72	2188.83	2341.12
2008	412.82	451.27	498.92	552.80	326.81	352.98	2221.70	2397.71	2704.92	2960.65	1736.61	1852.71	2730.86	2990.20	3296.90	3662.96	2164.39	2338.89
2009	408.48	450.68	494.12	552.71	322.81	351.81	2193.30	2384.91	2672.37	2948.24	1711.74	1839.04	2708.60	2997.43	3273.00	3675.70	2143.07	2340.20
2010	406.56	452.70	492.42	556.04	320.59	352.48	2182.49	2391.46	2661.99	2960.70	1699.98	1839.42	2698.05	3016.79	3263.98	3704.57	2130.35	2349.79
2011	407.41	457.56	494.47	563.34	320.17	354.91	2193.39	2421.62	2681.29	3006.23	1702.08	1854.28	2699.65	3047.95	3271.92	3750.75	2125.04	2365.90
2012	409.84	463.87	498.95	573.12	320.46	357.77	2218.13	2466.41	2721.15	3074.82	1711.28	1875.56	2706.82	3082.83	3288.96	3804.74	2121.71	2381.76
2013	412.86	470.75	504.20	583.75	321.17	360.93	2247.92	2517.08	2768.04	3152.22	1723.51	1899.83	2716.43	3119.36	3309.35	3861.59	2119.91	2398.03
2014	415.55	477.35	508.81	593.75	321.90	364.15	2273.67	2564.57	2808.09	3223.50	1734.69	1923.73	2725.30	3155.48	3327.26	3916.12	2119.35	2415.72
2015	416.93	482.37	511.05	600.90	322.37	367.02	2286.32	2596.79	2827.42	3270.32	1740.43	1941.30	2730.61	3186.87	3336.56	3959.91	2120.36	2434.55
2016	416.64	485.21	510.66	604.72	322.13	368.84	2284.92	2611.99	2825.77	3291.72	1738.96	1950.10	2729.83	3209.26	3335.58	3989.52	2119.47	2449.46
2017	416.15	487.70	509.87	607.87	321.92	370.61	2281.92	2624.93	2821.44	3309.35	1737.10	1958.11	2728.07	3229.77	3332.56	4015.74	2118.73	2463.99
2018	415.77	490.03	509.23	610.69	321.86	372.40	2279.26	2635.89	2816.51	3322.08	1737.19	1966.95	2726.91	3250.07	3330.19	4040.65	2119.07	2479.35
2019	415.06	491.48	508.05	612.03	321.80	373.89	2274.02	2640.51	2807.01	3323.86	1737.36	1973.94	2723.91	3265.91	3324.78	4058.08	2119.30	2493.20

Table S14 The crude and age-standardized prevalence, incidence, and DALYs rate of depression of different gender of Sub-Saharan Africa from 1990 to 2019

Year	DALY rate						Incidence rate						Prevalence rate					
	Both		Male		Female		Both		Male		Female		Both		Male		Female	
	AS	Crude	AS	Crude	AS	Crude	AS	Crude	AS	Crude	AS	Crude	AS	Crude	AS	Crude	AS	Crude
1990	817.65	578.60	967.55	692.36	663.35	462.40	5324.45	3746.83	6338.35	4511.69	4280.53	2965.63	4703.68	3310.08	5560.82	3962.03	3820.93	2644.21
1991	815.69	577.91	965.16	691.60	661.78	461.81	5310.96	3742.38	6321.72	4506.34	4270.08	2962.27	4695.07	3308.07	5550.28	3960.15	3814.07	2642.20
1992	813.99	577.54	962.94	691.13	660.63	461.62	5298.77	3739.37	6306.03	4502.56	4261.51	2960.50	4687.21	3307.08	5540.11	3959.26	3808.56	2641.51
1993	812.56	577.34	961.03	690.88	659.73	461.57	5288.69	3737.53	6292.26	4499.90	4255.58	2960.23	4680.50	3306.87	5530.99	3959.25	3804.56	2641.71
1994	811.82	577.50	960.06	691.32	659.19	461.52	5284.40	3738.76	6286.62	4502.93	4252.35	2960.13	4677.55	3308.51	5527.14	3962.90	3802.24	2641.73
1995	811.65	578.06	960.00	692.51	658.79	461.52	5284.24	3742.76	6287.65	4511.40	4250.21	2960.01	4677.61	3312.32	5527.77	3970.41	3801.02	2642.15
1996	814.08	580.64	963.16	696.39	660.33	462.86	5303.43	3761.68	6313.06	4539.99	4262.13	2969.64	4689.87	3325.57	5543.28	3990.17	3809.22	2649.24
1997	819.59	585.66	970.00	703.40	664.32	465.93	5345.13	3797.92	6365.41	4590.84	4291.84	2991.57	4716.30	3349.88	5575.19	4023.93	3829.16	2664.42
1998	825.93	591.27	977.64	711.03	669.16	469.56	5393.32	3838.88	6424.52	4647.13	4327.80	3017.50	4746.34	3376.70	5610.30	4060.08	3853.12	2682.20
1999	831.14	595.93	983.76	717.38	673.27	472.57	5433.02	3872.75	6471.85	4693.47	4358.49	3039.14	4771.10	3399.21	5638.29	4090.44	3873.59	2697.14
2000	833.04	597.96	985.73	720.27	674.90	473.80	5447.99	3886.84	6487.19	4712.97	4371.83	3048.15	4780.24	3409.38	5647.01	4104.65	3882.13	2703.53
2001	832.53	597.97	984.30	720.14	675.16	474.00	5443.67	3885.17	6476.14	4709.96	4373.13	3048.20	4777.46	3409.74	5639.76	4105.00	3882.91	2704.22
2002	831.68	597.66	982.16	719.35	675.44	474.22	5435.79	3880.39	6458.38	4700.88	4374.08	3048.10	4772.32	3408.09	5628.12	4101.48	3883.34	2704.71
2003	830.65	597.15	979.72	718.22	675.67	474.37	5425.91	3873.73	6437.27	4688.36	4374.40	3047.54	4765.95	3405.22	5614.50	4095.81	3883.33	2704.84
2004	829.60	596.59	977.22	716.92	675.90	474.56	5415.51	3866.56	6415.96	4675.25	4373.75	3046.41	4759.61	3402.17	5601.00	4089.63	3883.16	2704.95
2005	828.59	596.13	975.03	715.94	675.84	474.61	5405.75	3860.25	6397.10	4664.23	4371.67	3044.71	4753.41	3399.62	5588.79	4084.78	3881.75	2704.62
2006	825.94	594.21	970.92	713.15	674.42	473.51	5384.14	3843.43	6364.73	4640.71	4359.26	3034.34	4739.49	3390.17	5567.75	4071.59	3873.61	2698.65
2007	820.68	590.05	963.49	707.44	671.09	470.83	5342.55	3809.25	6307.27	4594.88	4331.94	3011.39	4712.59	3369.66	5530.58	4044.32	3855.59	2684.52
2008	814.01	584.76	954.28	700.30	666.71	467.31	5290.98	3767.08	6237.08	4538.55	4297.33	2982.84	4678.74	3343.76	5484.67	4010.06	3832.30	2666.44
2009	807.35	579.65	945.13	693.39	662.27	463.88	5239.71	3726.53	6167.19	4484.16	4262.85	2955.46	4645.50	3319.38	5439.43	3977.52	3809.42	2649.56
2010	802.03	576.03	937.61	688.32	658.84	461.60	5198.74	3697.18	6110.22	4443.92	4235.82	2936.16	4618.95	3302.51	5402.27	3954.15	3791.66	2638.40
2011	797.19	573.36	930.82	684.45	655.65	459.98	5161.58	3674.89	6058.73	4412.68	4210.83	2921.92	4595.46	3290.87	5369.41	3937.45	3775.65	2630.97
2012	791.84	570.75	923.60	680.80	651.82	458.27	5119.52	3652.28	6002.30	4381.36	4181.00	2907.08	4568.99	3279.56	5333.79	3921.50	3756.41	2623.44
2013	786.77	568.75	916.85	677.96	648.12	456.96	5080.31	3634.59	5950.27	4356.67	4152.44	2895.44	4544.14	3271.50	5300.48	3909.77	3738.08	2618.14
2014	783.16	568.19	911.92	676.93	645.49	456.71	5052.12	3627.42	5912.44	4345.64	4131.69	2891.10	4526.21	3270.60	5276.17	3907.27	3724.60	2617.89
2015	782.15	569.91	910.24	678.83	644.80	458.08	5043.00	3636.34	5898.66	4355.49	4124.83	2898.05	4520.88	3280.91	5267.46	3918.93	3720.61	2625.91
2016	783.80	573.62	912.39	683.46	645.50	460.71	5054.68	3660.63	5915.34	4386.98	4128.35	2914.01	4528.77	3301.38	5278.15	3944.88	3723.21	2639.93
2017	785.46	577.67	914.56	688.49	646.26	463.63	5066.67	3687.27	5931.73	4420.82	4133.06	2932.49	4536.47	3323.54	5288.40	3972.64	3726.11	2655.66
2018	786.09	581.62	914.50	692.84	647.34	467.08	5070.35	3712.23	5930.03	4447.75	4140.58	2954.76	4539.14	3345.51	5287.25	3996.90	3731.25	2674.68
2019	786.51	585.76	913.51	696.89	649.05	471.23	5072.06	3737.85	5921.13	4471.96	4152.20	2981.24	4540.40	3368.15	5281.28	4019.73	3738.99	2696.61

Table S15 The crude and age-standardized prevalence, incidence, and DALYs rate of anxiety of different gender of world from 1990 to 2019

Year	DALY rate						Incidence rate						Prevalence rate					
	Both		Male		Female		Both		Male		Female		Both		Male		Female	
	AS	Crude	AS	Crude	AS	Crude	AS	Crude	AS	Crude	AS	Crude	AS	Crude	AS	Crude	AS	Crude
1990	360.55	348.81	447.59	434.98	272.74	263.85	579.30	581.81	680.83	685.27	481.24	479.80	3791.59	3643.53	4732.19	4574.00	2839.18	2726.06
1991	360.08	349.01	446.98	435.24	272.50	264.05	578.77	581.85	680.01	685.10	481.05	480.13	3785.87	3645.60	4724.69	4576.75	2836.26	2728.28
1992	359.61	349.29	446.49	435.73	272.14	264.20	578.37	582.20	679.59	685.50	480.74	480.52	3780.29	3648.69	4718.31	4581.63	2832.44	2730.36
1993	359.19	349.72	446.07	436.40	271.80	264.46	578.08	582.83	679.44	686.34	480.37	481.01	3774.99	3653.04	4712.76	4588.54	2828.15	2732.82
1994	358.75	350.21	445.66	437.18	271.37	264.70	577.84	583.56	679.43	687.36	479.92	481.50	3769.84	3658.31	4707.67	4596.80	2823.44	2735.50
1995	358.34	350.79	445.28	438.07	270.93	264.98	577.60	584.31	679.48	688.44	479.40	481.94	3764.91	3664.43	4703.04	4606.29	2818.45	2738.44
1996	358.83	352.32	446.76	440.82	270.45	265.30	578.29	585.96	681.58	691.47	478.71	482.22	3769.93	3680.85	4718.33	4635.81	2813.22	2741.98
1997	360.66	355.21	450.91	446.26	269.94	265.67	580.29	588.88	686.51	697.27	477.80	482.31	3789.02	3711.51	4761.73	4693.32	2807.84	2746.10
1998	363.02	358.69	456.09	452.81	269.46	266.12	582.68	592.19	692.36	703.98	476.76	482.24	3813.58	3748.39	4815.91	4762.62	2802.48	2750.81
1999	365.06	361.93	460.63	458.82	268.99	266.59	584.61	594.95	697.25	709.68	475.71	482.05	3835.22	3783.10	4863.97	4826.99	2797.34	2755.86
2000	366.05	364.14	463.05	462.80	268.52	267.01	585.20	596.20	699.35	712.46	474.75	481.74	3845.55	3806.89	4889.16	4869.46	2792.55	2760.73
2001	365.55	364.85	462.74	464.05	267.81	267.13	583.94	595.36	698.07	711.57	473.46	480.88	3840.14	3815.11	4885.92	4883.83	2784.93	2762.32
2002	364.08	364.55	460.90	463.70	266.72	266.82	581.38	593.02	694.78	708.43	471.58	479.26	3824.21	3812.43	4865.86	4880.76	2773.24	2759.46
2003	362.15	363.71	458.25	462.44	265.52	266.35	578.36	590.04	690.70	704.27	469.57	477.39	3803.79	3804.46	4837.84	4868.70	2760.57	2754.95
2004	360.41	362.93	455.77	461.18	264.53	265.99	575.76	587.32	687.07	700.33	467.95	475.80	3784.99	3796.89	4810.87	4856.10	2750.07	2751.72
2005	359.38	362.74	454.18	460.65	264.07	266.06	574.35	585.69	684.98	697.80	467.18	475.01	3773.68	3795.37	4793.50	4851.39	2744.88	2752.72
2006	359.61	363.66	453.94	461.32	264.78	267.20	575.04	586.06	685.10	697.27	468.43	476.21	3775.45	3805.62	4790.12	4859.06	2751.99	2765.03
2007	360.87	365.53	454.56	462.71	266.72	269.50	577.47	588.07	686.66	698.02	471.71	479.42	3787.67	3825.40	4795.69	4874.36	2771.34	2788.88
2008	362.54	367.75	455.53	464.37	269.12	272.25	580.57	590.72	688.71	699.16	475.87	483.53	3804.03	3848.72	4804.42	4891.90	2795.80	2817.55
2009	363.94	369.66	456.19	465.63	271.29	274.73	583.28	592.97	690.27	699.78	479.69	487.33	3817.85	3869.14	4810.28	4905.90	2817.86	2843.74
2010	364.37	370.58	455.91	465.93	272.43	276.20	584.43	593.75	690.33	699.00	481.90	489.58	3821.85	3879.61	4806.80	4910.21	2829.47	2859.60
2011	363.41	370.09	453.94	464.48	272.49	276.61	583.78	592.73	688.36	696.19	482.52	490.27	3811.40	3875.55	4785.72	4896.36	2829.93	2864.63
2012	361.54	368.65	450.65	461.60	272.10	276.56	582.21	590.76	685.10	692.02	482.63	490.43	3791.23	3861.38	4750.12	4866.96	2825.74	2865.02
2013	359.46	366.98	447.00	458.31	271.64	276.44	580.45	588.61	681.56	687.58	482.62	490.50	3768.75	3844.55	4711.08	4833.42	2820.40	2864.23
2014	357.83	365.74	444.06	455.72	271.35	276.50	579.15	586.95	678.71	683.87	482.86	490.83	3751.25	3832.69	4679.68	4807.54	2817.32	2865.79
2015	357.30	365.60	442.80	454.83	271.60	277.06	578.95	586.37	677.47	681.77	483.69	491.72	3745.80	3832.67	4666.85	4800.35	2819.68	2872.45
2016	358.62	367.32	443.89	456.35	273.17	278.94	580.51	587.51	677.93	681.35	486.34	494.37	3759.97	3852.40	4678.90	4818.79	2836.11	2893.15
2017	359.97	369.14	445.06	458.06	274.70	280.85	582.39	589.12	678.99	681.69	489.05	497.21	3774.80	3873.69	4692.30	4839.72	2852.56	2914.46
2018	360.21	370.00	445.13	458.84	275.15	281.74	583.83	590.52	680.79	683.06	490.15	498.59	3778.62	3885.34	4694.56	4851.44	2858.14	2925.68
2019	360.12	370.61	444.89	459.49	275.20	282.30	585.45	592.20	683.76	685.71	490.50	499.27	3779.52	3895.21	4694.67	4862.88	2859.85	2933.64

Table S16 The crude and age-standardized prevalence, incidence, and DALYs rate of anxiety of different gender of Central Europe, Eastern Europe, and Central Asia from 1990 to 2019

Year	DALY rate						Incidence rate						Prevalence rate					
	Both		Male		Female		Both		Male		Female		Both		Male		Female	
	AS	Crude	AS	Crude	AS	Crude	AS	Crude	AS	Crude	AS	Crude	AS	Crude	AS	Crude	AS	Crude
1990	294.30	303.57	362.46	377.03	217.96	223.18	484.63	490.37	580.73	576.69	386.98	395.92	3093.64	3201.18	3815.86	3998.56	2277.57	2328.62
1991	294.28	304.37	362.65	378.11	217.87	223.74	484.51	491.30	580.76	577.75	386.78	396.76	3092.93	3209.63	3817.19	4009.73	2276.42	2334.73
1992	294.18	305.38	362.69	379.42	217.73	224.45	484.33	492.63	580.72	579.30	386.51	397.87	3091.57	3220.65	3817.27	4023.85	2274.83	2342.53
1993	294.06	306.61	362.65	380.97	217.58	225.28	484.11	494.13	580.57	581.05	386.22	399.07	3090.00	3233.41	3816.25	4039.80	2273.11	2351.52
1994	293.90	307.79	362.48	382.41	217.42	226.13	483.85	495.43	580.29	582.53	385.91	400.11	3088.31	3246.19	3814.71	4055.54	2271.33	2360.45
1995	293.78	309.07	362.36	383.96	217.28	227.04	483.52	496.59	579.89	583.81	385.58	401.05	3086.46	3259.32	3812.78	4071.46	2269.52	2369.75
1996	293.27	310.04	361.77	385.10	216.90	227.77	482.78	497.20	578.99	584.40	384.97	401.62	3080.88	3269.64	3806.15	4083.47	2265.47	2377.68
1997	292.26	310.55	360.55	385.67	216.19	228.19	481.54	497.07	577.50	584.14	383.97	401.62	3070.18	3275.39	3792.95	4089.56	2258.38	2382.90
1998	291.11	310.85	359.14	385.99	215.39	228.49	480.10	496.41	575.77	583.24	382.86	401.24	3057.67	3278.79	3777.20	4092.56	2250.32	2386.74
1999	290.02	311.16	357.85	386.33	214.61	228.75	478.78	495.64	574.16	582.14	381.85	400.81	3046.46	3282.86	3763.21	4096.49	2243.03	2390.84
2000	289.48	311.93	357.22	387.22	214.24	229.35	477.93	495.15	573.11	581.36	381.22	400.61	3040.15	3291.03	3755.63	4105.90	2238.91	2397.33
2001	289.21	312.84	356.92	388.30	214.08	230.03	477.36	494.57	572.39	580.41	380.83	400.37	3037.07	3301.16	3752.28	4118.07	2236.95	2404.78
2002	288.96	313.58	356.55	389.11	214.01	230.67	476.67	493.56	571.55	578.95	380.36	399.81	3033.45	3309.02	3748.16	4127.55	2234.74	2410.41
2003	288.63	313.99	356.13	389.56	213.85	230.99	475.97	492.29	570.69	577.22	379.88	399.00	3029.76	3313.86	3743.65	4133.32	2232.50	2413.78
2004	288.40	314.29	355.79	389.83	213.78	231.26	475.32	490.89	569.91	575.35	379.41	398.07	3026.14	3316.66	3739.31	4136.65	2230.19	2415.46
2005	288.13	314.38	355.45	389.91	213.64	231.32	474.80	489.58	569.33	573.62	379.02	397.15	3022.87	3318.32	3735.57	4138.69	2227.99	2416.02
2006	288.17	314.62	355.60	390.28	213.63	231.37	474.82	488.61	569.57	572.41	378.90	396.39	3022.70	3321.39	3736.75	4143.54	2227.21	2416.63
2007	288.59	315.07	356.38	391.05	213.77	231.43	475.39	487.97	570.70	571.75	379.03	395.75	3026.24	3326.54	3743.80	4152.38	2228.14	2417.47
2008	289.13	315.40	357.30	391.71	213.99	231.38	476.14	487.51	572.09	571.38	379.23	395.18	3031.04	3330.47	3752.55	4160.10	2229.63	2417.08
2009	289.48	315.36	357.93	391.87	214.14	231.14	476.65	486.79	573.16	570.66	379.30	394.46	3034.31	3331.02	3759.18	4163.39	2230.44	2414.72
2010	289.44	314.81	358.05	391.36	214.06	230.57	476.56	485.46	573.33	569.11	379.09	393.41	3033.39	3326.23	3759.73	4159.11	2229.38	2409.70
2011	289.12	313.93	357.75	390.39	213.85	229.83	475.98	483.67	572.76	566.91	378.63	392.12	3029.62	3318.00	3755.95	4150.01	2227.15	2402.91
2012	288.84	313.14	357.41	389.48	213.78	229.24	475.30	481.85	572.02	564.67	378.12	390.82	3025.83	3310.17	3751.98	4141.71	2225.08	2396.26
2013	288.49	312.33	356.93	388.53	213.68	228.64	474.56	480.31	571.16	562.77	377.61	389.75	3022.02	3302.76	3747.56	4133.82	2223.13	2390.10
2014	288.21	311.58	356.55	387.68	213.60	228.08	473.78	478.84	570.21	560.90	377.10	388.78	3017.81	3294.87	3742.51	4125.11	2221.07	2383.84
2015	287.69	310.56	355.87	386.44	213.40	227.36	472.99	477.37	569.20	558.98	376.64	387.89	3012.91	3285.95	3736.48	4114.70	2218.79	2377.23
2016	287.07	309.36	354.79	384.66	213.42	226.86	471.71	475.35	566.64	555.45	376.80	387.58	3006.02	3274.40	3724.35	4097.06	2219.05	2373.00
2017	286.39	308.29	353.60	382.99	213.44	226.50	470.55	473.59	564.25	552.20	377.01	387.53	2999.19	3264.69	3712.73	4081.67	2219.17	2370.22
2018	286.04	307.85	353.37	382.53	213.16	226.15	470.31	473.32	564.22	551.76	376.73	387.51	2995.21	3261.02	3709.77	4077.77	2216.21	2367.57
2019	285.83	307.75	353.58	382.64	212.73	225.88	470.73	474.01	565.59	552.96	376.36	387.73	2993.33	3261.46	3712.42	4080.74	2212.07	2365.99

Table S17 The crude and age-standardized prevalence, incidence, and DALYs rate of anxiety of different gender of High-income from 1990 to 2019

Year	DALY rate						Incidence rate						Prevalence rate					
	Both		Male		Female		Both		Male		Female		Both		Male		Female	
	AS	Crude	AS	Crude	AS	Crude	AS	Crude	AS	Crude	AS	Crude	AS	Crude	AS	Crude	AS	Crude
1990	464.48	490.27	597.49	629.32	327.76	345.13	707.22	701.37	843.22	813.37	574.92	584.49	4881.19	5181.92	6312.04	6702.48	3404.47	3594.85
1991	464.16	490.15	598.11	630.20	326.67	344.05	705.89	698.77	842.15	810.15	573.49	582.58	4876.68	5180.98	6316.61	6711.25	3392.92	3584.51
1992	463.94	490.13	598.84	631.13	325.67	343.09	705.29	696.91	842.29	808.16	572.28	580.89	4873.43	5181.18	6322.47	6721.13	3382.49	3575.30
1993	463.76	490.18	599.49	632.04	324.83	342.30	705.33	695.81	843.50	807.37	571.29	579.52	4871.07	5182.48	6328.92	6731.74	3373.17	3567.49
1994	463.71	490.36	600.26	633.05	324.11	341.66	705.92	695.38	845.54	807.60	570.53	578.43	4869.59	5184.88	6335.60	6742.85	3365.26	3561.36
1995	463.70	490.60	600.93	633.99	323.57	341.22	707.00	695.47	848.30	808.62	570.00	577.60	4868.93	5188.37	6342.30	6754.17	3358.95	3557.21
1996	467.10	494.58	606.27	639.95	325.17	343.20	713.05	700.80	857.74	816.54	572.76	580.29	4904.58	5231.73	6397.92	6818.51	3376.11	3579.35
1997	475.20	503.67	617.85	652.60	329.90	348.64	725.79	712.85	876.06	833.12	580.03	587.66	4990.59	5330.06	6521.59	6956.09	3425.64	3637.50
1998	484.95	514.59	631.53	667.53	335.84	355.46	740.73	727.02	897.18	852.32	588.92	596.65	5093.52	5447.15	6667.33	7117.58	3487.03	3709.18
1999	493.15	523.78	642.95	680.00	340.92	361.30	753.36	738.64	914.86	867.93	596.59	604.18	5180.17	5546.20	6788.93	7252.74	3539.94	3771.25
2000	496.54	527.68	647.74	685.25	343.08	363.86	758.88	742.80	922.66	873.48	599.92	606.93	5215.90	5589.06	6839.55	7310.47	3562.58	3799.39
2001	495.21	526.28	645.76	683.02	342.59	363.40	757.49	739.67	920.44	868.74	599.41	605.53	5201.23	5575.08	6818.24	7288.01	3556.79	3795.01
2002	492.03	522.57	641.00	677.38	341.19	361.75	753.47	733.35	914.01	859.01	597.87	602.81	5167.04	5536.45	6766.93	7228.55	3542.00	3778.68
2003	488.10	517.86	635.09	670.27	339.43	359.58	748.28	725.53	905.70	847.04	595.82	599.35	5124.17	5486.39	6702.04	7151.96	3523.28	3756.70
2004	484.34	513.29	629.34	663.35	337.81	357.50	743.35	718.04	897.90	835.72	593.80	595.86	5083.09	5438.22	6639.46	7078.36	3505.61	3735.47
2005	481.71	510.12	625.31	658.57	336.71	356.04	740.11	712.52	892.89	827.77	592.29	592.90	5054.69	5405.55	6595.73	7028.35	3494.07	3721.28
2006	481.11	509.23	623.55	656.57	337.37	356.35	739.86	710.32	891.44	824.27	593.15	592.09	5048.16	5397.90	6576.91	7009.28	3500.81	3725.93
2007	482.07	509.89	623.04	655.99	339.83	358.34	741.89	710.32	891.84	823.01	596.64	593.42	5058.07	5406.98	6572.23	7006.40	3525.95	3747.84
2008	483.61	511.17	623.09	656.05	342.91	360.91	744.70	711.20	892.87	822.70	601.05	595.58	5073.72	5422.29	6572.67	7009.91	3557.32	3775.82
2009	484.65	511.93	622.79	655.72	345.29	362.81	746.94	711.61	893.41	821.91	604.78	597.22	5085.18	5433.21	6569.78	7009.44	3583.34	3798.60
2010	484.27	511.12	621.25	653.83	346.10	363.11	746.94	709.72	892.08	818.77	605.96	596.62	5081.09	5426.62	6554.25	6992.40	3591.03	3802.73
2011	481.77	508.01	618.19	649.98	344.21	360.76	743.39	704.48	887.38	811.99	603.50	592.97	5054.90	5395.53	6522.04	6953.50	3571.61	3779.69
2012	477.97	503.43	614.38	645.01	340.55	356.63	737.30	696.84	880.05	802.45	598.66	587.33	5014.79	5348.73	6481.31	6902.14	3533.59	3737.97
2013	473.94	498.61	610.64	639.99	336.41	352.08	730.55	688.61	872.08	792.22	593.20	581.22	4972.12	5298.87	6440.93	6849.65	3490.55	3691.48
2014	470.73	494.65	607.69	635.82	333.13	348.40	725.01	681.51	865.45	783.25	588.85	576.10	4938.38	5258.46	6409.90	6807.34	3456.21	3653.90
2015	469.34	492.66	606.36	633.62	331.86	346.72	722.49	677.18	862.03	777.43	587.32	573.40	4924.51	5239.80	6396.70	6786.46	3443.68	3638.50
2016	468.54	491.50	603.84	630.69	332.93	347.47	720.51	673.47	856.55	770.32	588.82	573.26	4917.87	5231.23	6373.33	6760.89	3455.46	3648.52
2017	468.16	490.82	601.91	628.44	334.25	348.51	719.39	670.53	852.15	764.07	590.97	573.80	4914.87	5227.30	6354.59	6741.23	3469.82	3661.69
2018	472.27	495.48	606.71	633.97	337.78	352.33	724.59	673.89	857.34	766.40	596.31	578.27	4961.28	5282.67	6409.88	6808.57	3508.59	3705.35
2019	480.87	505.62	617.02	646.38	344.73	360.17	735.38	682.91	869.28	774.95	606.12	587.80	5058.29	5400.65	6528.09	6955.52	3585.00	3793.93

Table S18 The crude and age-standardized prevalence, incidence, and DALYs rate of anxiety of different gender of Latin America and Caribbean from 1990 to 2019

Year	DALY rate						Incidence rate						Prevalence rate					
	Both		Male		Female		Both		Male		Female		Both		Male		Female	
	AS	Crude	AS	Crude	AS	Crude	AS	Crude	AS	Crude	AS	Crude	AS	Crude	AS	Crude	AS	Crude
1990	450.06	417.65	556.10	521.35	339.00	311.60	708.67	708.84	816.90	832.83	597.72	582.05	4740.59	4351.59	5886.92	5462.38	3538.47	3215.75
1991	451.49	421.91	557.71	526.51	340.12	314.85	711.25	714.40	820.26	839.27	599.56	586.58	4752.55	4394.39	5900.74	5514.97	3547.19	3247.49
1992	453.16	426.27	559.80	531.95	341.26	318.02	714.12	719.90	824.01	845.72	601.58	591.03	4767.77	4438.81	5919.27	5570.40	3557.82	3279.69
1993	454.92	430.51	562.02	537.28	342.45	321.05	716.83	724.85	827.59	851.52	603.44	594.99	4783.47	4481.73	5939.13	5624.80	3568.19	3309.95
1994	456.43	434.28	564.00	542.15	343.40	323.63	718.94	728.76	830.45	856.11	604.81	598.12	4797.08	4520.25	5957.14	5674.69	3576.28	3336.00
1995	457.32	437.20	565.27	546.00	343.80	325.51	720.05	731.20	832.10	858.94	605.40	600.07	4806.10	4551.66	5970.27	5716.84	3580.17	3355.62
1996	463.09	444.40	573.62	556.36	346.79	329.40	725.38	737.61	838.89	866.91	609.25	604.80	4866.39	4627.95	6057.66	5826.74	3611.03	3396.63
1997	476.54	458.73	593.13	577.37	353.77	336.80	737.85	751.33	854.54	884.32	618.49	614.65	5008.16	4779.48	6263.08	6048.79	3684.83	3474.96
1998	493.74	476.71	618.35	604.01	362.44	345.81	753.72	768.61	874.66	906.50	630.02	626.81	5190.55	4970.48	6530.29	6332.00	3776.84	3570.43
1999	510.86	494.85	644.01	631.39	370.47	354.39	769.19	785.62	894.83	928.75	640.74	638.38	5372.78	5164.08	6803.30	6624.05	3862.40	3662.07
2000	524.15	509.68	664.97	654.56	375.61	360.59	780.49	798.47	910.61	946.28	647.52	646.36	5514.07	5322.35	7026.18	6871.33	3916.84	3728.18
2001	535.41	522.87	683.93	676.06	378.73	365.16	789.14	808.65	923.88	960.93	651.58	651.89	5633.39	5463.07	7227.71	7101.47	3949.05	3776.45
2002	547.68	537.29	704.74	699.74	382.00	370.01	798.39	819.50	938.10	976.52	655.91	657.83	5763.66	5617.00	7449.90	7355.51	3982.17	3826.92
2003	558.98	550.95	724.07	722.24	384.85	374.54	807.02	829.66	951.32	990.91	660.03	663.58	5884.62	5763.92	7656.54	7597.19	4012.71	3875.84
2004	567.58	562.05	738.67	740.18	387.13	378.55	813.81	837.75	961.61	1001.97	663.43	668.58	5975.97	5882.99	7811.41	7789.27	4037.09	3919.27
2005	571.62	568.64	745.08	749.92	388.68	381.85	817.56	842.46	967.04	1007.64	665.64	672.26	6017.58	5953.30	7878.57	7894.23	4051.86	3953.32
2006	571.08	570.47	744.32	752.20	388.40	383.15	817.38	842.73	966.91	1007.06	665.60	673.36	6011.20	5974.13	7869.40	7920.36	4048.52	3968.04
2007	568.41	569.98	740.90	751.60	386.51	382.70	814.24	839.72	963.02	1002.37	663.38	672.00	5981.27	5969.53	7830.48	7914.49	4027.98	3963.98
2008	564.87	568.52	736.43	749.82	383.93	381.49	809.72	835.16	957.29	995.61	660.28	669.64	5941.74	5954.27	7778.84	7894.47	4000.96	3952.73
2009	561.65	567.35	732.17	748.20	381.76	380.69	805.43	830.78	951.60	988.78	657.56	667.70	5906.22	5942.71	7731.12	7877.72	3977.88	3945.50
2010	559.99	567.81	729.65	748.34	380.97	381.35	802.92	828.20	947.85	983.79	656.47	667.50	5888.21	5949.16	7703.92	7881.72	3969.00	3953.23
2011	555.17	564.99	721.23	742.20	379.95	381.87	798.95	823.79	941.30	975.37	655.24	667.13	5836.65	5921.01	7613.87	7818.86	3957.97	3959.73
2012	544.37	555.90	702.69	725.25	377.35	380.78	791.01	815.03	928.75	960.36	652.12	664.76	5720.80	5825.64	7415.66	7640.76	3929.57	3948.79
2013	531.54	544.49	680.81	704.46	374.13	378.98	781.86	804.92	914.41	943.38	648.37	661.66	5584.54	5706.85	7183.11	7423.02	3895.58	3931.24
2014	520.87	535.12	662.57	687.19	371.53	377.69	774.19	796.36	902.41	928.94	645.22	659.11	5471.06	5609.35	6989.44	7242.73	3867.51	3918.43
2015	516.36	532.01	654.77	680.75	370.54	377.93	770.71	792.27	896.85	921.43	643.93	658.48	5423.55	5578.88	6907.30	7178.03	3857.13	3922.45
2016	519.41	536.74	658.59	686.47	372.80	381.51	773.71	794.94	900.48	923.54	646.25	661.63	5455.26	5630.70	6946.54	7240.61	3881.04	3961.72
2017	522.67	541.69	662.76	692.56	375.12	385.13	777.48	798.43	905.43	927.06	648.78	664.95	5490.36	5686.07	6991.26	7309.01	3906.04	4001.93
2018	523.38	543.94	663.82	695.38	375.51	386.66	778.81	799.44	907.92	928.09	649.06	665.83	5496.92	5711.36	7001.27	7340.83	3909.58	4019.08
2019	524.13	546.19	665.02	698.29	375.88	388.11	780.33	800.58	911.21	929.82	648.99	666.26	5502.31	5734.90	7011.29	7371.83	3911.17	4033.67

Table S19 The crude and age-standardized prevalence, incidence, and DALYs rate of anxiety of different gender of North Africa and Middle East from 1990 to 2019

Year	DALY rate						Incidence rate						Prevalence rate					
	Both		Male		Female		Both		Male		Female		Both		Male		Female	
	AS	Crude	AS	Crude	AS	Crude	AS	Crude	AS	Crude	AS	Crude	AS	Crude	AS	Crude	AS	Crude
1990	474.09	451.51	593.28	560.52	360.33	347.72	752.09	785.56	873.21	916.38	636.99	661.00	4950.32	4660.79	6241.85	5827.06	3716.77	3550.33
1991	473.26	453.12	593.18	563.71	358.91	347.85	752.13	787.82	876.48	923.09	634.01	659.07	4941.28	4677.64	6239.52	5860.09	3702.63	3552.17
1992	472.20	454.38	592.67	566.40	357.46	347.83	751.65	789.17	878.54	928.11	631.20	657.00	4930.13	4691.34	6233.58	5888.20	3688.06	3552.80
1993	471.77	456.31	592.67	569.61	356.74	348.58	751.61	790.87	880.23	932.53	629.56	656.16	4924.71	4710.87	6231.46	5920.14	3680.66	3560.94
1994	471.76	458.57	592.87	572.89	356.53	349.84	751.81	792.67	881.38	936.10	628.81	656.25	4923.75	4734.25	6232.18	5954.16	3678.37	3574.04
1995	471.92	460.89	593.17	576.14	356.57	351.26	751.90	793.98	881.71	938.38	628.58	656.62	4924.50	4757.88	6233.63	5987.00	3678.55	3588.70
1996	471.88	462.94	592.86	578.62	356.83	352.93	751.12	794.10	879.52	937.58	629.12	657.64	4923.35	4778.96	6229.64	6012.97	3680.53	3605.27
1997	471.53	464.73	591.73	580.28	357.25	354.85	749.33	793.03	874.45	933.46	630.43	659.50	4919.23	4797.45	6218.12	6031.34	3683.89	3624.15
1998	471.35	466.61	590.74	582.02	357.81	356.87	747.35	791.54	868.53	928.14	632.12	661.64	4915.62	4816.18	6205.48	6048.33	3688.68	3644.50
1999	471.30	468.56	589.82	583.78	358.52	358.97	745.85	790.26	863.73	923.62	633.67	663.40	4914.66	4836.61	6197.13	6068.94	3694.07	3664.42
2000	471.61	470.68	589.94	586.43	358.97	360.56	745.52	789.83	862.00	921.99	634.59	664.09	4918.18	4859.72	6198.45	6097.83	3699.19	3681.79
2001	472.41	473.06	591.24	589.99	359.30	361.82	746.80	790.70	864.46	924.36	634.73	663.57	4926.57	4885.13	6212.35	6136.52	3702.37	3694.73
2002	473.56	475.61	593.49	594.33	359.44	362.75	749.23	792.57	869.75	929.44	634.50	662.44	4938.33	4912.13	6235.36	6182.00	3703.97	3704.85
2003	474.85	478.19	595.98	598.77	359.63	363.62	752.11	794.80	876.10	935.52	634.19	661.10	4952.01	4940.12	6262.42	6230.60	3705.33	3713.96
2004	475.89	480.37	598.21	602.76	359.57	364.14	754.53	796.39	881.47	940.32	633.88	659.68	4964.09	4964.77	6286.23	6274.07	3706.32	3721.24
2005	476.76	482.27	599.62	605.81	359.95	365.00	755.74	796.57	883.99	941.88	633.90	658.63	4972.13	4984.31	6300.76	6306.87	3708.53	3728.83
2006	478.42	484.81	601.78	609.31	361.36	366.81	756.75	796.58	884.43	941.27	635.69	659.45	4989.33	5012.05	6322.85	6345.11	3723.49	3748.61
2007	481.61	488.72	605.64	614.21	364.39	370.17	758.64	797.57	884.75	940.63	639.55	662.41	5022.68	5053.90	6364.27	6398.86	3754.34	3783.25
2008	485.15	492.88	609.96	619.33	367.85	373.92	760.82	798.84	884.95	939.73	644.28	666.31	5061.17	5100.01	6412.33	6456.99	3790.87	3823.51
2009	488.43	496.62	614.01	623.98	370.96	377.24	762.82	799.80	885.01	938.32	648.63	669.98	5094.95	5139.91	6454.26	6507.14	3822.91	3858.43
2010	490.27	498.67	616.25	626.63	372.65	378.94	764.09	799.73	884.89	936.18	651.34	672.06	5113.56	5162.13	6477.32	6536.40	3839.96	3876.19
2011	491.10	499.66	617.43	628.07	373.46	379.75	765.29	799.39	884.89	933.77	653.80	673.90	5123.27	5175.08	6490.41	6554.60	3849.66	3886.87
2012	492.51	501.19	619.14	629.95	374.89	381.22	767.37	800.05	885.23	931.69	657.72	677.40	5138.23	5193.29	6508.28	6576.71	3865.38	3904.34
2013	494.12	502.94	620.86	631.82	376.61	383.06	769.74	801.18	885.68	929.85	662.01	681.50	5154.66	5212.89	6526.18	6598.53	3882.77	3923.98
2014	495.33	504.34	622.16	633.32	377.86	384.49	771.70	802.17	886.10	928.19	665.49	685.07	5168.02	5229.93	6540.13	6616.83	3896.86	3941.18
2015	495.84	505.13	622.57	633.97	378.58	385.54	772.53	802.31	886.32	926.69	666.97	686.85	5173.27	5239.89	6546.24	6628.10	3902.70	3951.33
2016	492.77	502.50	619.25	631.02	375.96	383.38	771.02	800.29	888.68	927.47	662.29	682.41	5142.14	5215.10	6511.42	6599.69	3877.21	3931.79
2017	489.63	499.90	615.81	628.17	373.23	381.14	769.62	798.74	891.71	929.70	657.14	677.49	5109.42	5189.96	6474.70	6571.66	3849.68	3910.65
2018	490.09	501.12	617.17	630.54	372.89	381.34	773.66	802.99	900.68	938.79	656.60	677.31	5114.55	5204.65	6489.70	6599.53	3846.10	3913.74
2019	492.15	504.06	621.57	636.23	372.82	381.81	783.08	812.57	919.37	957.77	657.27	678.25	5135.71	5236.79	6534.57	6660.07	3845.75	3920.22

Table S20 The crude and age-standardized prevalence, incidence, and DALYs rate of anxiety of different gender of South Asia from 1990 to 2019

Year	DALY rate						Incidence rate						Prevalence rate					
	Both		Male		Female		Both		Male		Female		Both		Male		Female	
	AS	Crude	AS	Crude	AS	Crude	AS	Crude	AS	Crude	AS	Crude	AS	Crude	AS	Crude	AS	Crude
1990	282.78	246.62	333.29	292.19	236.37	204.53	492.22	464.01	557.95	534.42	431.48	398.98	3023.12	2601.48	3595.45	3107.81	2497.59	2133.87
1991	281.40	246.27	330.80	291.14	235.99	204.86	490.36	463.40	554.59	532.58	431.06	399.54	3008.77	2598.27	3569.57	3097.58	2493.43	2137.33
1992	279.84	245.74	328.40	290.12	235.15	204.79	488.16	462.47	551.21	530.67	430.00	399.53	2992.30	2593.02	3543.66	3086.77	2485.24	2137.35
1993	278.17	245.13	326.01	289.08	234.11	204.57	485.75	461.34	547.95	528.81	428.42	399.09	2974.72	2586.73	3518.85	3076.48	2473.85	2134.80
1994	276.53	244.53	323.93	288.25	232.83	204.17	483.27	460.13	544.95	527.10	426.46	398.32	2957.08	2580.31	3496.31	3067.78	2460.13	2130.37
1995	274.97	244.00	322.17	287.66	231.42	203.67	480.84	458.97	542.33	525.69	424.24	397.34	2940.39	2574.64	3477.19	3061.69	2444.98	2124.84
1996	272.03	242.00	319.82	286.14	227.86	201.20	475.85	454.96	538.70	522.60	418.02	392.44	2909.31	2554.11	3452.94	3046.92	2406.82	2098.63
1997	267.25	238.08	316.82	283.55	221.35	196.01	467.40	447.21	533.53	517.31	406.52	382.34	2858.44	2513.03	3420.02	3019.16	2338.36	2044.75
1998	262.14	233.79	313.84	280.86	214.17	190.19	458.19	438.61	528.27	511.84	393.57	370.78	2803.53	2467.51	3387.04	2990.19	2261.88	1983.31
1999	258.01	230.51	311.51	279.01	208.25	185.52	450.88	432.05	524.36	508.10	382.99	361.50	2760.17	2433.78	3362.50	2971.81	2199.63	1934.68
2000	256.52	229.97	310.90	279.40	205.82	184.05	448.13	430.39	523.22	508.01	378.61	358.27	2743.93	2428.10	3354.84	2975.70	2173.81	1919.38
2001	257.31	231.79	311.86	281.75	206.29	185.30	449.53	433.13	525.26	511.67	379.26	360.03	2751.89	2447.33	3364.68	3000.89	2178.32	1932.21
2002	258.90	234.41	313.77	285.05	207.44	187.21	452.39	437.41	529.21	517.42	380.92	362.83	2768.09	2474.95	3384.05	3036.07	2189.85	1951.86
2003	260.86	237.43	316.15	288.89	208.85	189.37	455.94	442.42	534.04	524.12	383.08	366.11	2788.30	2506.91	3408.31	3076.77	2204.48	1974.66
2004	262.87	240.55	318.66	292.94	210.23	191.52	459.39	447.33	538.71	530.64	385.21	369.36	2808.32	2539.31	3432.86	3118.58	2218.41	1997.15
2005	264.45	243.31	320.68	296.55	211.24	193.37	462.00	451.36	542.16	535.89	386.82	372.08	2823.97	2568.07	3453.07	3156.95	2227.92	2015.79
2006	267.12	247.38	323.71	301.58	213.42	196.45	466.38	457.37	546.84	542.61	390.78	377.26	2850.54	2610.09	3482.66	3208.64	2250.02	2047.58
2007	271.80	253.76	328.52	308.84	217.87	201.88	474.19	467.13	554.43	552.71	398.66	386.54	2897.41	2675.26	3530.07	3283.14	2295.04	2102.77
2008	277.15	260.90	333.87	316.83	223.11	208.12	483.14	478.14	562.81	563.69	408.04	397.40	2951.45	2748.60	3582.73	3364.43	2349.13	2167.39
2009	281.95	267.44	338.42	323.84	228.05	214.10	490.96	487.91	569.87	573.11	416.50	407.34	2999.57	2815.39	3628.15	3436.93	2398.50	2227.53
2010	284.84	271.82	341.10	328.55	231.00	218.05	495.42	494.02	573.52	578.61	421.62	413.85	3028.70	2861.04	3653.96	3485.44	2429.34	2269.23
2011	286.33	274.65	342.29	331.42	232.66	220.73	497.36	497.46	574.70	581.39	424.20	417.76	3044.10	2891.50	3666.65	3517.63	2445.88	2296.90
2012	287.82	277.54	343.70	334.59	234.12	223.27	499.10	500.83	575.67	584.16	426.58	421.56	3059.12	2922.22	3680.07	3550.94	2461.16	2324.10
2013	289.14	280.29	344.91	337.56	235.42	225.72	500.57	504.02	576.42	586.75	428.71	425.20	3072.88	2952.29	3692.90	3584.06	2474.65	2350.30
2014	290.27	282.93	346.02	340.49	236.49	228.00	501.76	506.94	576.90	589.02	430.53	428.62	3084.52	2980.86	3703.86	3615.73	2485.89	2375.05
2015	291.10	285.34	346.80	343.12	237.30	230.13	502.60	509.48	577.08	590.85	431.98	431.73	3093.05	3007.08	3711.64	3644.75	2494.29	2397.85
2016	291.86	287.72	347.40	345.61	238.16	232.35	502.87	511.26	575.72	590.82	433.81	435.18	3100.50	3032.70	3717.11	3671.75	2503.02	2421.54
2017	292.22	289.73	347.54	347.66	238.68	234.28	502.90	512.80	574.34	590.71	435.19	438.21	3104.94	3055.78	3719.55	3696.02	2508.86	2442.87
2018	290.51	289.51	344.99	346.80	237.73	234.62	501.38	512.83	572.61	590.44	433.83	438.45	3087.17	3055.01	3692.99	3689.16	2498.96	2447.38
2019	286.44	286.73	339.16	342.42	235.29	233.33	497.90	510.97	569.40	588.99	430.01	436.15	3045.53	3028.34	3633.47	3646.96	2473.82	2435.09

Table S21 The crude and age-standardized prevalence, incidence, and DALYs rate of anxiety of different gender of Southeast Asia, East Asia, and Oceania from 1990 to 2019

Year	DALY rate						Incidence rate						Prevalence rate					
	Both		Male		Female		Both		Male		Female		Both		Male		Female	
	AS	Crude	AS	Crude	AS	Crude	AS	Crude	AS	Crude	AS	Crude	AS	Crude	AS	Crude	AS	Crude
1990	334.54	332.90	416.76	417.23	253.84	251.82	552.70	562.13	665.48	682.62	445.56	446.28	3486.70	3436.83	4364.52	4331.90	2621.40	2576.27
1991	334.48	332.94	416.56	417.26	254.02	252.00	552.88	562.18	665.49	682.33	446.02	446.84	3484.89	3437.79	4360.80	4332.85	2622.65	2578.58
1992	334.50	333.27	416.65	417.83	254.07	252.21	553.26	563.01	666.02	683.23	446.35	447.77	3483.86	3441.45	4359.54	4338.62	2622.94	2581.43
1993	334.60	333.93	416.88	418.86	254.11	252.60	553.74	564.49	666.89	685.08	446.55	449.02	3483.46	3448.18	4360.10	4349.30	2622.41	2585.33
1994	334.68	334.85	417.18	420.30	254.04	253.09	554.29	566.41	667.98	687.52	446.65	450.52	3483.58	3458.42	4362.15	4364.94	2621.32	2591.02
1995	334.87	336.17	417.66	422.26	253.97	253.81	554.87	568.58	669.17	690.34	446.68	452.12	3484.49	3472.13	4365.53	4385.28	2620.07	2598.61
1996	336.03	338.66	420.36	426.71	253.59	254.41	556.93	572.27	673.49	696.29	446.59	453.61	3496.05	3498.47	4393.02	4432.24	2615.63	2605.04
1997	338.57	342.66	426.27	434.55	252.77	254.68	560.97	577.90	682.02	706.53	446.27	454.75	3521.59	3539.80	4453.15	4513.45	2606.37	2607.61
1998	341.40	347.10	433.07	443.55	251.58	254.67	565.33	583.80	691.65	717.86	445.50	455.31	3550.65	3586.44	4523.43	4607.61	2593.70	2607.76
1999	343.54	351.03	438.66	451.55	250.22	254.57	568.33	588.09	699.24	726.85	444.03	454.94	3572.25	3627.43	4581.20	4691.70	2578.58	2606.19
2000	343.89	353.18	440.92	456.23	248.62	254.15	568.32	588.87	701.67	730.12	441.59	453.15	3575.40	3650.38	4603.69	4741.18	2561.68	2602.24
2001	340.99	351.88	437.63	455.06	246.03	252.60	563.57	583.93	696.54	724.50	437.09	448.67	3544.99	3638.20	4568.82	4730.57	2534.85	2587.11
2002	335.40	347.57	430.08	449.12	242.30	249.72	555.06	574.28	685.79	712.02	430.66	441.59	3485.78	3594.00	4488.29	4669.25	2496.10	2558.08
2003	328.73	341.88	420.63	440.87	238.34	246.41	545.29	562.81	672.96	696.71	423.80	433.67	3416.24	3536.70	4389.92	4586.12	2454.64	2524.53
2004	322.94	336.90	412.32	433.52	235.02	243.61	536.84	552.53	661.64	682.74	418.10	426.82	3355.30	3486.23	4302.20	4511.29	2420.08	2496.53
2005	319.81	334.48	407.80	429.92	233.25	242.25	532.28	546.45	655.43	674.27	415.14	422.92	3322.30	3462.65	4253.82	4475.27	2402.20	2484.06
2006	319.09	334.42	406.31	429.27	233.32	242.70	531.53	544.50	653.78	670.61	415.32	422.55	3314.21	3463.53	4237.18	4470.29	2402.78	2489.94
2007	319.07	334.93	405.21	428.76	234.40	244.15	532.25	544.13	653.30	668.02	417.32	424.27	3312.97	3470.02	4224.44	4466.56	2413.47	2505.89
2008	319.37	335.71	404.34	428.29	235.91	246.07	533.63	544.51	653.33	665.85	420.11	427.04	3314.75	3478.89	4213.27	4462.77	2428.47	2526.38
2009	319.51	336.27	403.33	427.58	237.23	247.80	534.88	544.81	653.28	663.54	422.71	429.77	3315.54	3486.56	4201.50	4457.44	2442.05	2545.78
2010	319.14	336.26	401.99	426.46	237.85	248.78	535.24	544.35	652.59	660.77	424.21	431.44	3311.50	3488.58	4187.19	4448.66	2448.63	2557.52
2011	317.75	335.11	399.47	423.96	237.65	248.89	534.02	542.24	650.19	656.22	424.26	431.63	3296.10	3478.19	4158.99	4423.95	2446.66	2560.35
2012	315.43	332.80	395.45	419.51	237.11	248.59	531.72	538.84	646.29	649.84	423.63	431.05	3271.29	3455.96	4115.97	4379.83	2441.00	2558.73
2013	313.09	330.30	391.36	414.70	236.62	248.27	529.30	535.22	642.22	643.12	422.95	430.36	3245.48	3430.93	4071.23	4330.83	2435.25	2556.38
2014	311.35	328.39	388.22	410.82	236.40	248.23	527.70	532.42	639.26	637.54	422.81	430.20	3226.95	3413.13	4037.76	4292.88	2432.89	2557.54
2015	311.06	327.87	387.28	409.21	236.83	248.72	527.78	531.22	638.58	634.24	423.76	430.95	3223.89	3410.16	4028.17	4279.57	2437.21	2564.06
2016	315.08	332.15	392.01	414.00	240.16	252.45	532.54	535.02	642.57	635.73	429.43	436.96	3266.27	3457.79	4078.32	4334.10	2471.78	2604.48
2017	319.22	336.78	396.90	419.24	243.58	256.44	537.87	539.98	647.45	638.73	435.34	443.76	3310.53	3509.94	4131.25	4394.84	2507.39	2647.78
2018	319.17	337.00	396.92	419.36	243.53	256.71	539.44	541.45	650.06	640.16	436.05	445.22	3311.65	3516.63	4132.99	4401.28	2508.63	2654.23
2019	317.21	335.43	395.04	417.78	241.58	255.10	539.27	541.43	652.20	641.62	433.76	443.70	3292.85	3504.78	4114.83	4390.16	2490.07	2641.14

Table S22 The crude and age-standardized prevalence, incidence, and DALYs rate of anxiety of different gender of Sub-Saharan Africa from 1990 to 2019

Year	DALY rate						Incidence rate						Prevalence rate					
	Both		Male		Female		Both		Male		Female		Both		Male		Female	
	AS	Crude	AS	Crude	AS	Crude	AS	Crude	AS	Crude	AS	Crude	AS	Crude	AS	Crude	AS	Crude
1990	325.62	282.16	378.91	331.49	270.22	231.77	544.45	525.68	608.40	596.46	479.13	453.39	3435.41	2939.45	4020.32	3468.86	2826.77	2398.72
1991	325.34	282.22	378.15	331.05	270.42	232.37	544.13	525.86	607.48	595.84	479.42	454.40	3432.93	2940.48	4013.39	3465.19	2828.92	2404.69
1992	325.04	282.34	377.46	330.76	270.55	232.93	543.83	526.11	606.72	595.47	479.60	455.33	3430.53	2942.28	4007.47	3463.42	2830.35	2410.43
1993	324.82	282.57	377.02	330.78	270.60	233.41	543.59	526.39	606.19	595.32	479.70	456.10	3428.58	2944.63	4003.21	3463.54	2831.17	2415.55
1994	324.63	282.76	376.68	330.87	270.57	233.74	543.41	526.69	605.84	595.39	479.72	456.70	3427.34	2947.28	4000.58	3465.32	2831.41	2419.45
1995	324.48	283.04	376.44	331.12	270.51	234.08	543.28	527.08	605.63	595.68	479.68	457.21	3426.56	2950.54	3999.10	3468.83	2831.19	2422.74
1996	324.24	283.38	376.50	331.89	269.95	234.02	543.03	527.54	605.78	596.63	479.06	457.23	3425.04	2954.63	4000.52	3477.12	2826.56	2422.91
1997	323.89	283.81	376.82	333.17	268.91	233.61	542.57	528.09	606.32	598.40	477.66	456.60	3421.82	2959.33	4004.41	3490.85	2815.97	2418.79
1998	323.49	284.27	377.18	334.60	267.73	233.13	542.02	528.63	606.94	600.37	476.00	455.73	3417.95	2964.00	4008.86	3506.02	2803.53	2413.16
1999	323.20	284.72	377.60	335.96	266.72	232.68	541.51	529.07	607.35	601.96	474.62	455.04	3414.61	2968.13	4012.15	3518.88	2793.26	2408.74
2000	323.00	285.04	377.63	336.66	266.26	232.62	541.16	529.35	607.29	602.63	474.02	454.94	3412.77	2971.30	4012.35	3525.84	2789.04	2408.33
2001	322.77	285.14	377.00	336.52	266.40	232.99	540.73	529.28	606.34	602.08	474.16	455.41	3410.30	2972.24	4006.05	3524.71	2790.23	2411.61
2002	322.36	285.09	375.92	335.99	266.68	233.45	540.01	528.94	604.61	600.77	474.53	456.08	3405.19	2970.98	3993.23	3518.10	2792.79	2415.98
2003	321.97	285.03	374.70	335.32	267.12	234.03	539.22	528.51	602.63	599.20	474.98	456.82	3399.24	2968.81	3978.24	3509.31	2795.88	2420.64
2004	321.64	285.00	373.67	334.75	267.49	234.53	538.56	528.18	600.93	597.86	475.41	457.52	3394.31	2967.11	3965.42	3501.75	2798.71	2424.88
2005	321.54	285.10	373.17	334.57	267.75	234.92	538.25	528.15	600.05	597.28	475.67	458.03	3392.12	2967.27	3958.91	3498.92	2800.41	2427.99
2006	322.17	285.75	373.67	335.08	268.45	235.68	538.85	528.92	600.46	597.82	476.42	459.00	3397.08	2972.51	3962.58	3503.00	2805.88	2434.16
2007	323.51	286.94	374.91	336.09	269.78	237.02	540.42	530.60	601.90	599.29	477.99	460.83	3409.27	2983.29	3974.11	3512.39	2817.66	2445.95
2008	325.17	288.40	376.52	337.39	271.38	238.61	542.35	532.67	603.75	601.17	479.88	463.04	3424.22	2996.49	3988.78	3524.26	2831.63	2459.99
2009	326.63	289.77	377.96	338.66	272.73	240.01	544.02	534.60	605.29	602.87	481.54	465.11	3437.38	3008.89	4001.58	3535.46	2843.78	2472.96
2010	327.42	290.68	378.62	339.43	273.53	241.01	544.85	535.86	605.86	603.82	482.49	466.59	3444.12	3017.16	4007.18	3542.63	2850.24	2481.65
2011	327.74	291.24	378.73	339.83	273.93	241.65	545.18	536.71	605.82	604.34	483.07	467.70	3446.45	3022.19	4007.13	3546.06	2853.58	2487.54
2012	328.06	291.81	378.80	340.25	274.37	242.30	545.70	537.79	605.98	605.14	483.85	468.96	3449.09	3027.63	4006.50	3549.42	2858.09	2494.31
2013	328.40	292.51	378.78	340.72	274.97	243.16	546.30	539.06	606.23	606.18	484.67	470.35	3451.94	3034.24	4005.90	3554.14	2863.07	2502.05
2014	328.74	293.37	378.87	341.49	275.45	244.04	546.90	540.50	606.55	607.48	485.46	471.83	3455.21	3043.02	4006.23	3561.94	2867.99	2511.04
2015	329.13	294.53	379.08	342.69	275.90	245.08	547.40	542.07	606.85	609.01	486.04	473.36	3458.70	3054.60	4008.04	3574.23	2871.89	2521.13
2016	329.91	296.19	379.99	344.70	276.43	246.32	548.29	544.12	607.89	611.33	486.66	475.03	3466.49	3071.65	4017.72	3595.70	2876.45	2532.98
2017	330.62	297.92	380.87	346.88	276.87	247.55	549.03	546.08	608.76	613.52	487.18	476.69	3472.97	3088.93	4025.54	3617.08	2880.47	2545.49
2018	330.44	299.00	380.26	347.83	277.08	248.71	548.78	547.07	607.89	613.93	487.55	478.21	3470.88	3100.14	4018.41	3626.85	2882.92	2557.71
2019	329.74	299.64	378.57	347.87	277.37	249.93	547.81	547.10	605.64	612.62	487.92	479.57	3462.63	3106.00	3999.85	3626.66	2885.02	2569.39

Supplementary Materials S3 The age-standardized DALY rates for depression and anxiety of different country by SDI in 2019

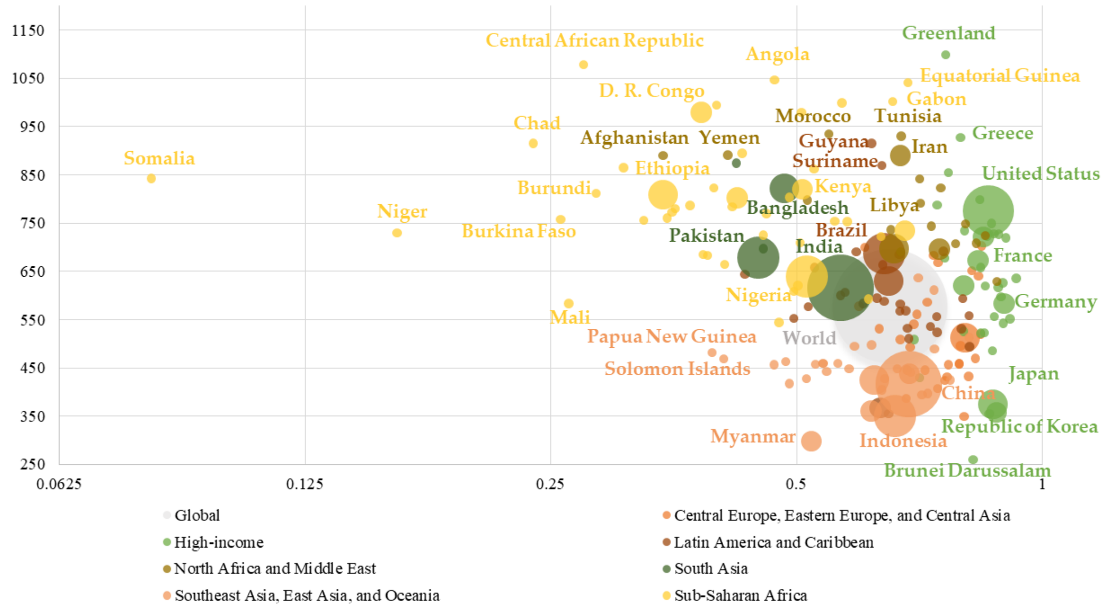

Figure S1 Age-standardized DALY rates for depression nationally by Socio-demographic Index (SDI) in 2019

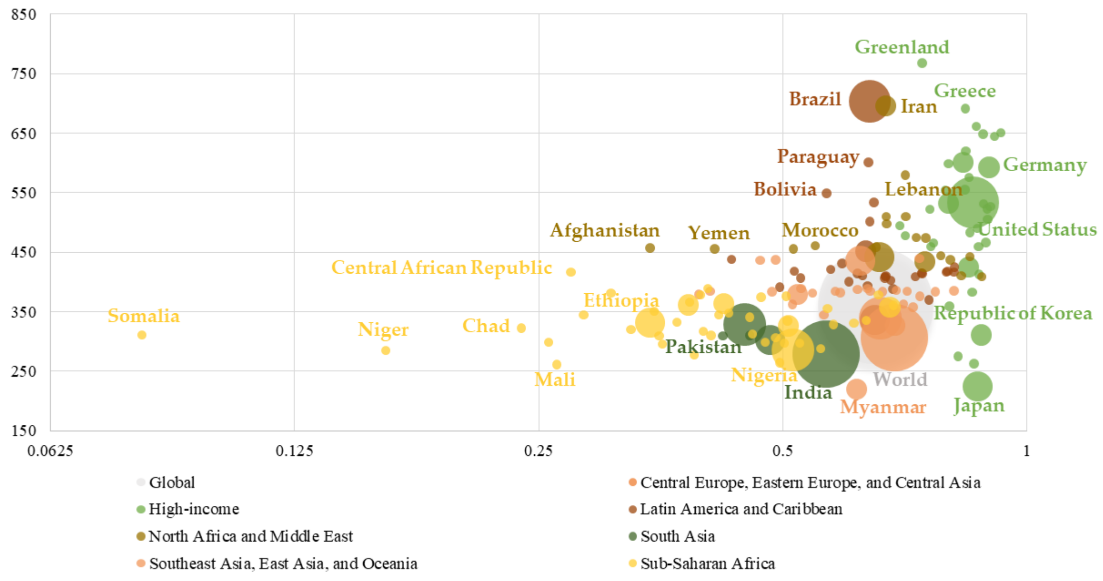

Figure S2 Age-standardized DALY rates for anxiety nationally by Socio-demographic Index (SDI) in 2019

Supplementary Materials S4 The age-standardized DALYs rate of three risk factors of different age groups by gender in 2019

Table S23 The age-standardized DALYs rate of three risk factors of different age groups by gender in 2019

Age	Childhood sexual abuse			Bullying victimization			Intimate partner violence		
	Both	Male	Female	Both	Male	Female	Both	Male	Female
0-14	1.4748	0.3095	2.7184	11.3433	8.9557	13.8912	0.0003	0.000	0.0007
15-19	17.4253	7.4165	27.9654	80.1728	66.2459	94.8392	19.8224	0.000	40.6973
20-24	28.7323	17.5554	40.2338	79.7211	70.3831	89.3302	51.7434	0.000	104.9899
25-29	30.7141	23.8757	37.6454	50.2986	45.6234	55.0374	58.5027	0.000	117.7999
30-34	34.3163	27.2935	41.4477	27.1370	25.1335	29.1714	60.1124	0.000	121.1543
35-39	43.1120	30.4206	55.9975	8.2934	7.7884	8.8060	59.9326	0.000	120.7815
40-44	47.1228	30.8615	63.6530	0.0019	0.0015	0.0024	60.9658	0.000	122.9396
45-49	45.8868	28.7042	63.2843	0.0000	0.0000	0.0000	65.4735	0.000	131.7655
50-54	43.8827	27.6857	59.9582	0.0000	0.0000	0.0000	68.1016	0.000	135.6922
55-59	42.7374	28.5606	56.5010	0.0000	0.0000	0.0000	68.4919	0.000	134.9869
60-64	38.4820	27.5742	48.8303	0.0000	0.0000	0.0000	63.1405	0.000	123.0426
65-69	31.5360	24.6766	37.8185	0.0000	0.0000	0.0000	54.2813	0.000	103.9977
70+	23.9332	22.0228	25.4263	0.0000	0.0000	0.0000	39.7983	0.000	70.9036

Table S24 The age-standardized DALYs rate of three risk factors of different age groups by gender in 2019

Age	Bullying victimization		
	Both	Male	Female
0-14	27.4166	30.9004	24.1520
15-19	87.0857	98.8375	75.9264
20-24	69.2676	78.5568	60.2407
25-29	47.6982	53.5851	41.8903
30-34	28.4774	31.5675	25.4343
35-39	13.2131	14.3795	12.0642
40-44	0.8300	0.8750	0.7858
45-49	0.0000	0.0000	0.0000
50-54	0.0000	0.0000	0.0000
55-59	0.0000	0.0000	0.0000
60-64	0.0000	0.0000	0.0000
65-69	0.0000	0.0000	0.0000
70+	0.0000	0.0000	0.0000

Supplementary Materials S5 The projection of depression and anxiety of all recorded countries of GBD

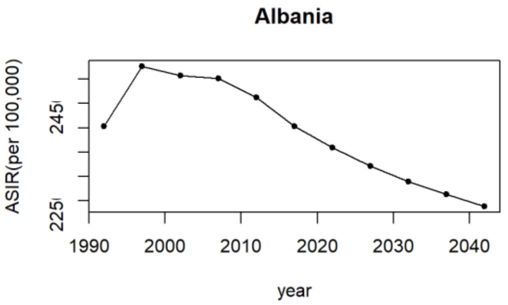

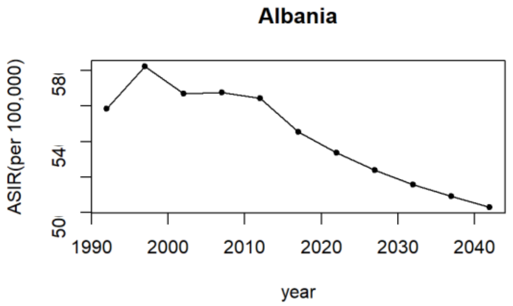

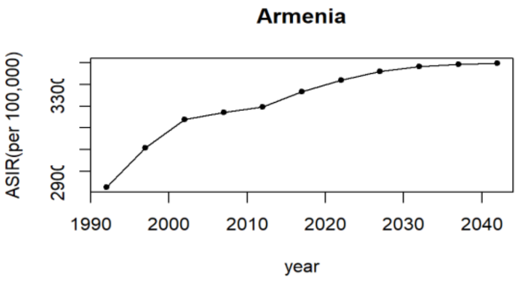

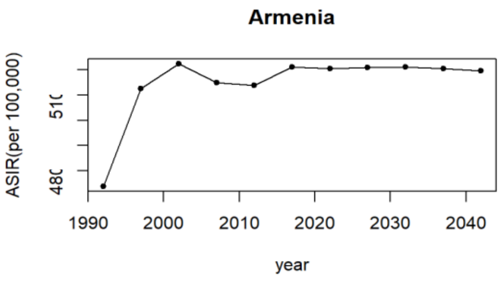

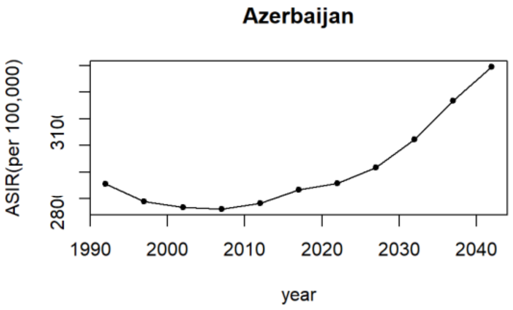

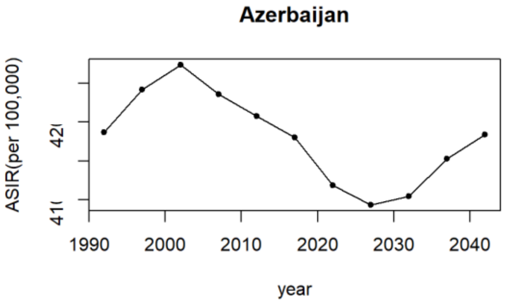

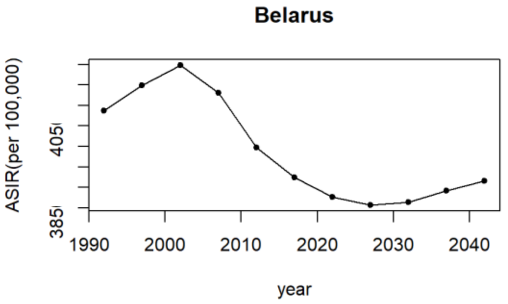

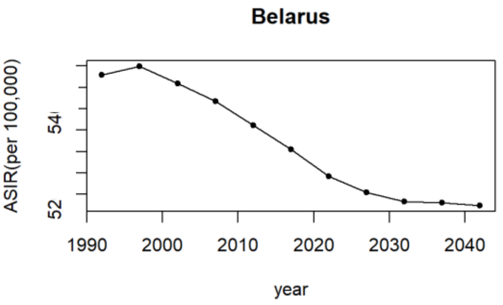

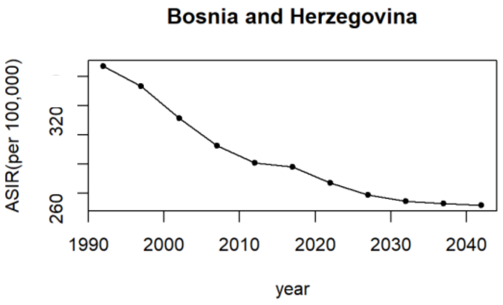
Bosnia and Herzegovina
ASIR(per 100,000)
320
260
1990
2000
2010
2020
2030
2040
year

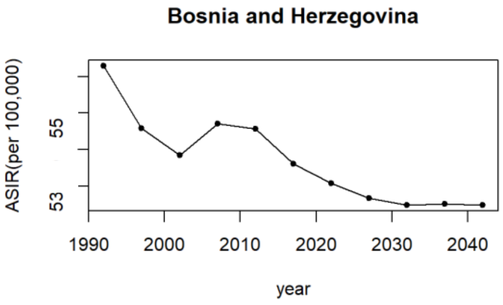
Bosnia and Herzegovina
ASIR(per 100,000)
55
53
1990
2000
2010
2020
2030
2040
year

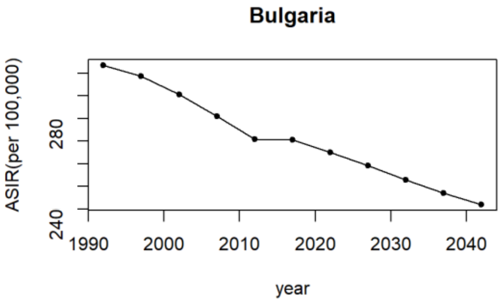
Bulgaria
ASIR(per 100,000)
280
240
1990
2000
2010
2020
2030
2040
year

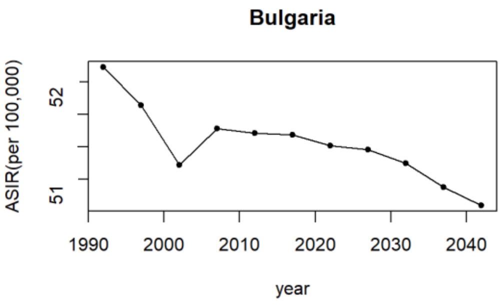
Bulgaria
ASIR(per 100,000)
52
51
1990
2000
2010
2020
2030
2040
year

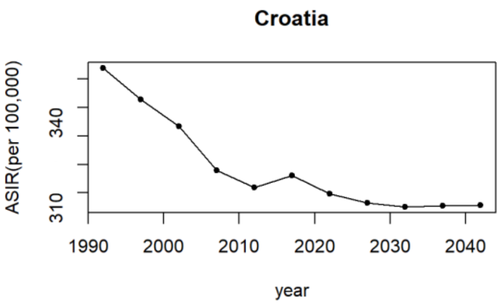
Croatia
ASIR(per 100,000)
340
310
1990
2000
2010
2020
2030
2040
year

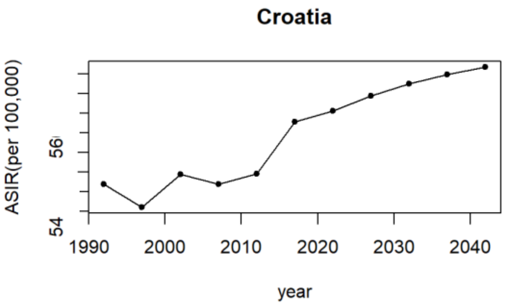
Croatia
ASIR(per 100,000)
56
54
1990
2000
2010
2020
2030
2040
year

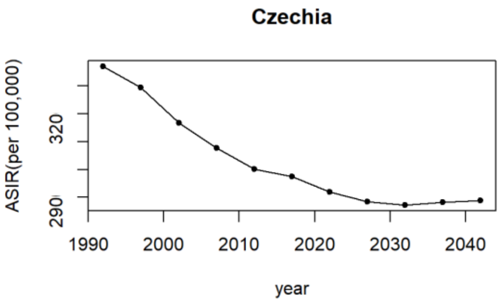
Czechia
ASIR(per 100,000)
320
290
1990
2000
2010
2020
2030
2040
year

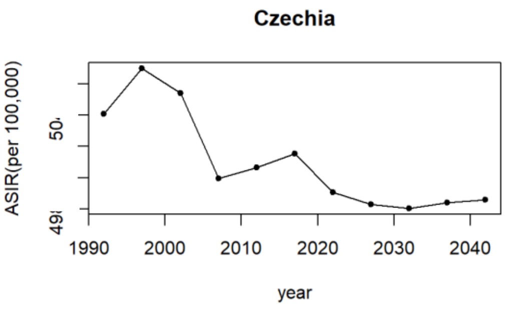
Czechia
ASIR(per 100,000)
1990
2000
2010
2020
2030
2040
year

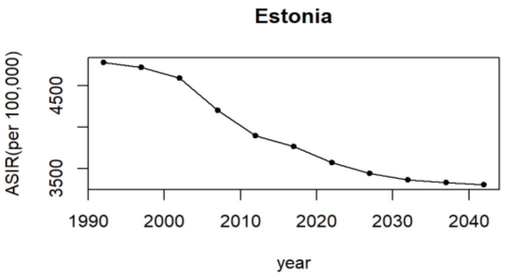
Estonia
ASIR(per 100,000)
4500
3500
1990
2000
2010
2020
2030
2040
year
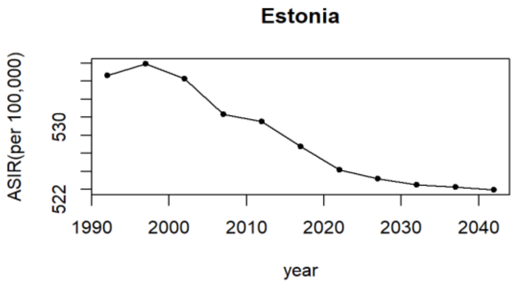
Estonia
ASIR(per 100,000)
530
522
1990
2000
2010
2020
2030
2040
year
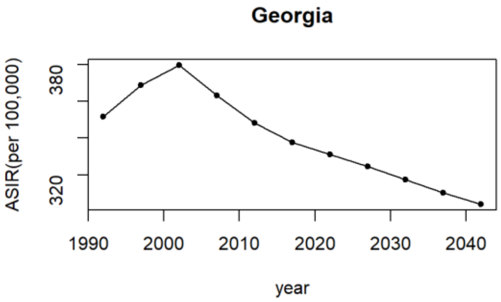
Georgia
ASIR(per 100,000)
380
320
1990
2000
2010
2020
2030
2040
year
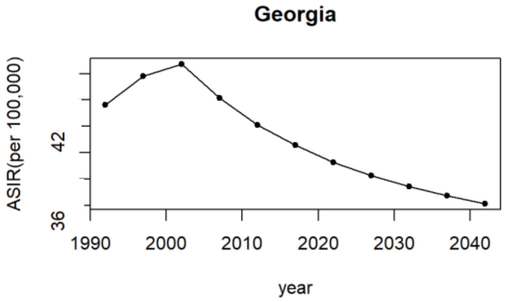
Georgia
ASIR(per 100,000)
42
36
1990
2000
2010
2020
2030
2040
year
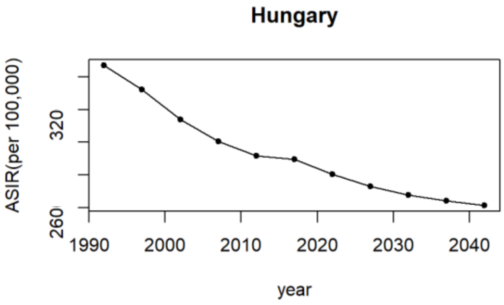
Hungary
ASIR(per 100,000)
320
260
1990
2000
2010
2020
2030
2040
year
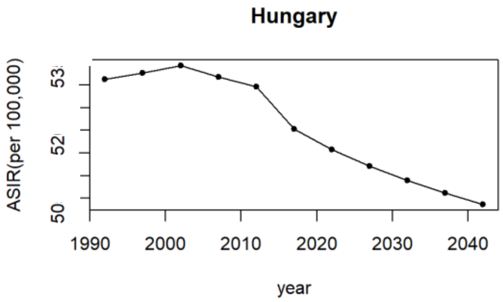
Hungary
ASIR(per 100,000
53
52
50
1990
2000
2010
2020
2030
2040
year
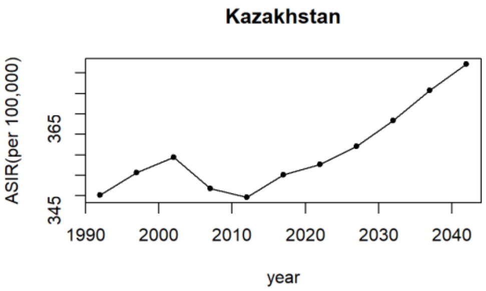
Kazakhstan
ASIR(per 100,000)
365
345
1990
2000
2010
2020
2030
2040
year
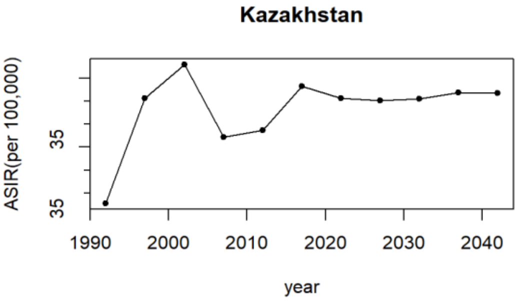
Kazakhstan
ASIR(per 100,000)
35
35
1990
2000
2010
2020
2030
2040
year

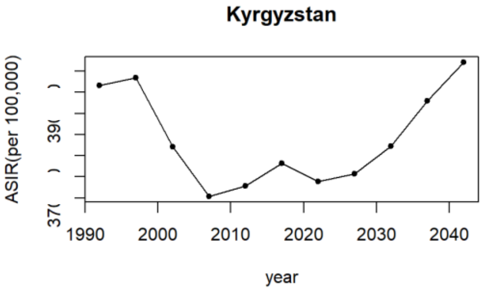
Kyrgyzstan
ASIR(per 100,000)
1990
2000
2010
2020
2030
2040
year

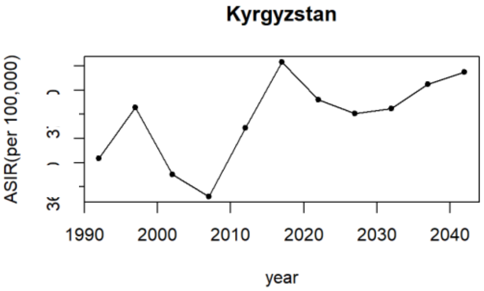
Kyrgyzstan
ASIR(per 100,000)
1990
2000
2010
2020
2030
2040
year

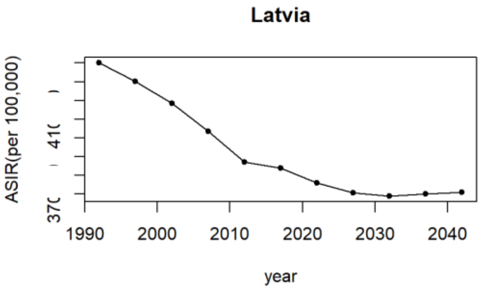
Latvia
ASIR(per 100,000)
1990
2000
2010
2020
2030
2040
year

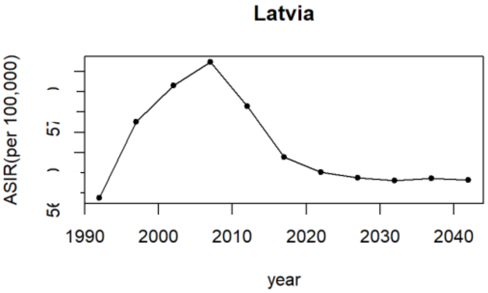
Latvia
ASIR(per 100,000)
1990
2000
2010
2020
2030
2040
year

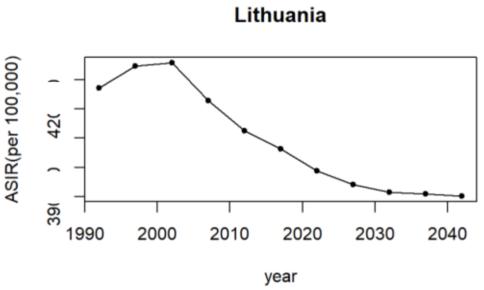
Lithuania
ASIR(per 100,000)
1990
2000
2010
2020
2030
2040
year

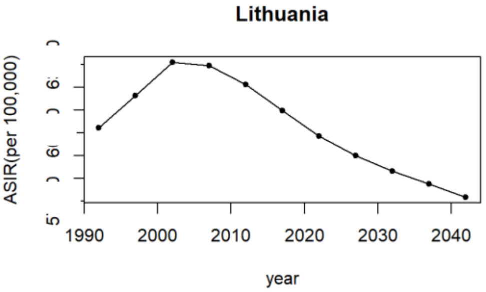
Lithuania
ASIR(per 100,000)
1990
2000
2010
2020
2030
2040
year

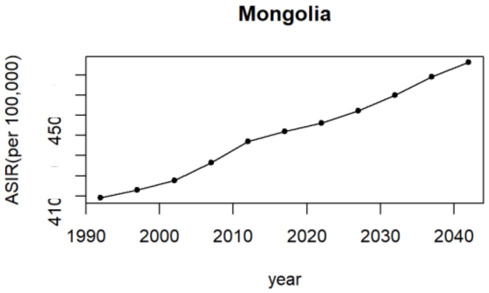
Mongolia
ASIR(per 100,000)
1990
2000
2010
2020
2030
2040
year

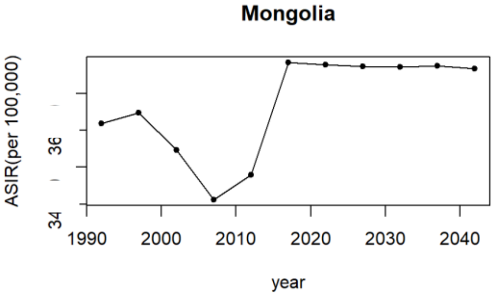
Mongolia
ASIR(per 100,000)
34
1990
2000
2010
2020
2030
2040
year

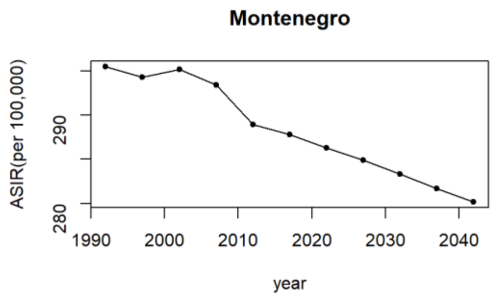
Montenegro
ASIR(per 100,000)
280
290
1990 2000 2010 2020 2030 2040
year

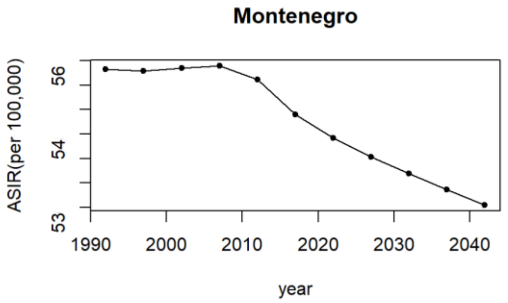
Montenegro
ASIR(per 100,000)
53
54
56
1990 2000 2010 2020 2030 2040
year

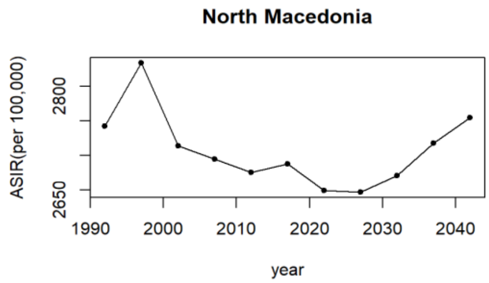
North Macedonia
ASIR(per 100,000)
2650
2800
1990 2000 2010 2020 2030 2040
year

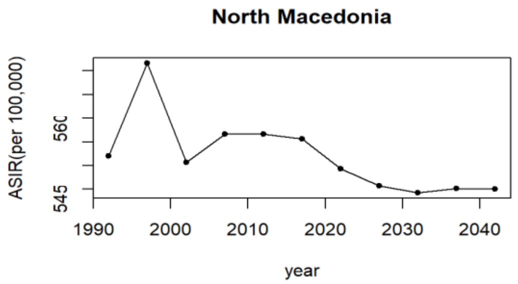
North Macedonia
ASIR(per 100,000)
545
1990 2000 2010 2020 2030 2040
year

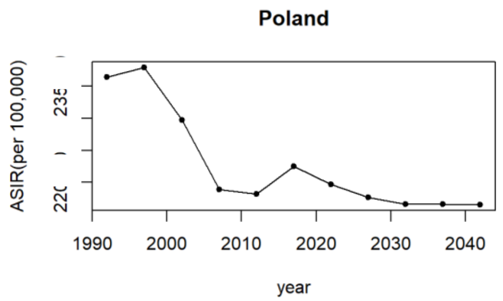
Poland
ASIR(per 100,000)
1990 2000 2010 2020 2030 2040
year

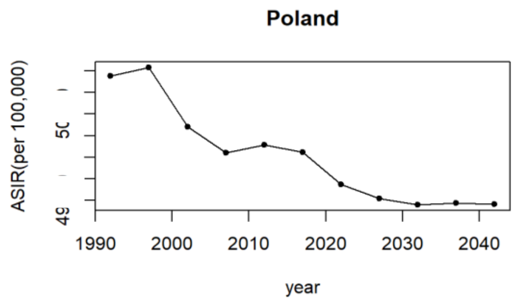
Poland
ASIR(per 100,000)
1990 2000 2010 2020 2030 2040
year

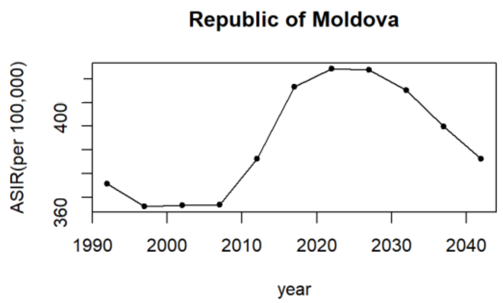
Republic of Moldova
ASIR(per 100,000)
360
400
1990 2000 2010 2020 2030 2040
year

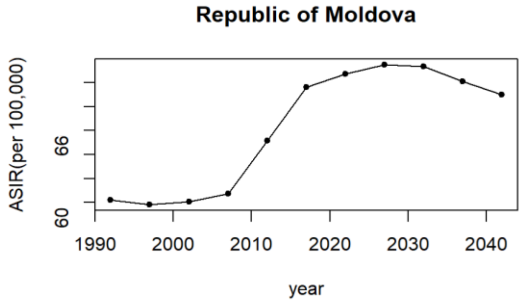
Republic of Moldova
ASIR(per 100,000)
60
66
1990 2000 2010 2020 2030 2040
year

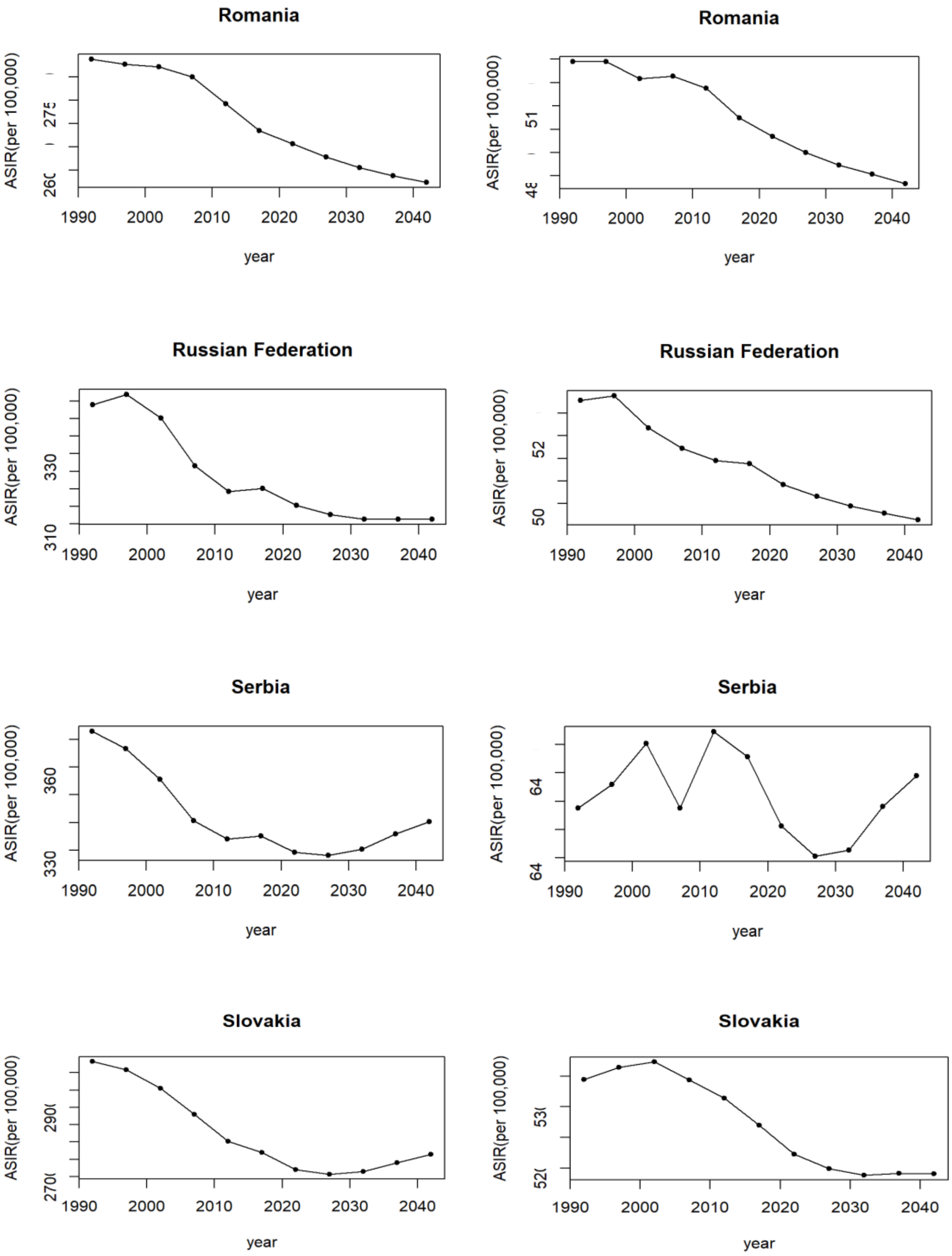
Romania
ASIR(per 100,000)
275
year
1990 2000 2010 2020 2030 2040
Romania
ASIR(per 100,000)
51
year
1990 2000 2010 2020 2030 2040
Russian Federation
ASIR(per 100,000)
310 330
year
1990 2000 2010 2020 2030 2040
Russian Federation
ASIR(per 100,000)
50 52
year
1990 2000 2010 2020 2030 2040
Serbia
ASIR(per 100,000)
330 360
year
1990 2000 2010 2020 2030 2040
Serbia
ASIR(per 100,000)
64 64
year
1990 2000 2010 2020 2030 2040
Slovakia
ASIR(per 100,000)
year
1990 2000 2010 2020 2030 2040
Slovakia
ASIR(per 100,000)
year
1990 2000 2010 2020 2030 2040

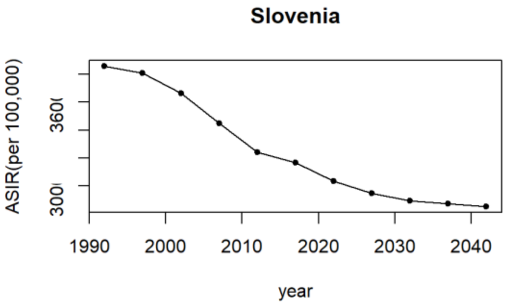
Slovenia
ASIR(per 100,000)
1990 2000 2010 2020 2030 2040
year

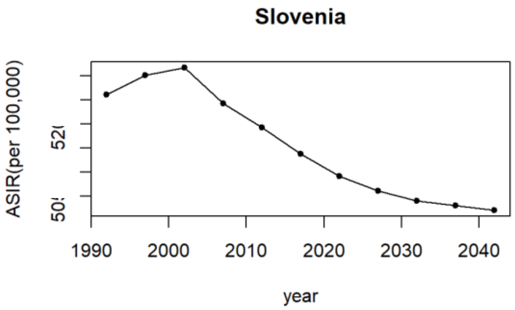
Slovenia
ASIR(per 100,000)
1990 2000 2010 2020 2030 2040
year

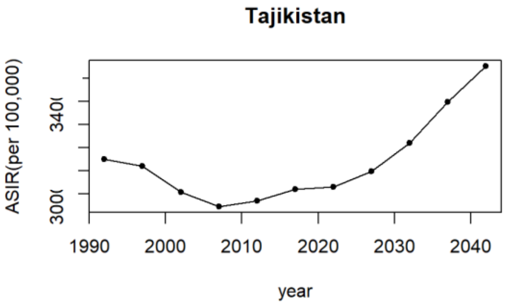
Tajikistan
ASIR(per 100,000)
1990 2000 2010 2020 2030 2040
year

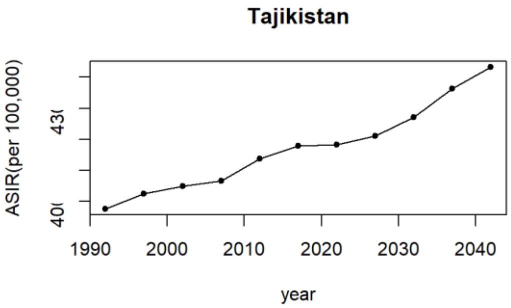
Tajikistan
ASIR(per 100,000)
1990 2000 2010 2020 2030 2040
year

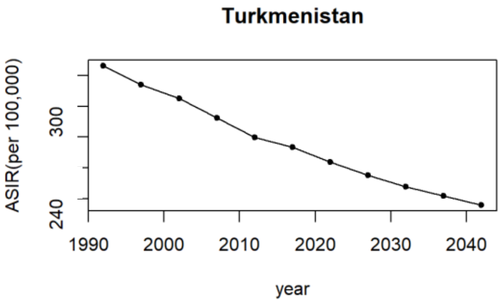
Turkmenistan
ASIR(per 100,000)
300
240
1990 2000 2010 2020 2030 2040
year

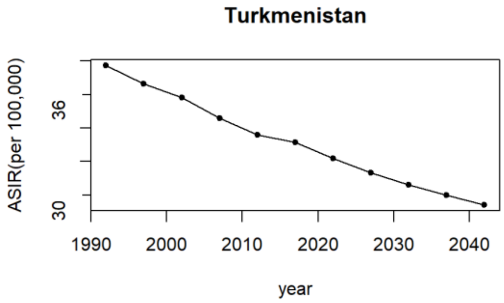
Turkmenistan
ASIR(per 100,000)
36
30
1990 2000 2010 2020 2030 2040
year

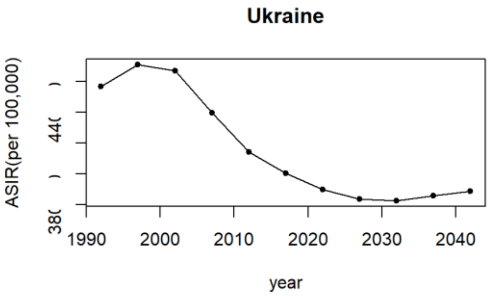
Ukraine
ASIR(per 100,000)
1990 2000 2010 2020 2030 2040
year

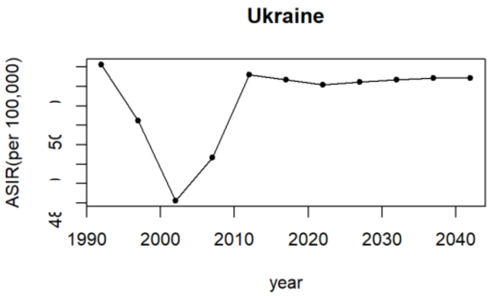
Ukraine
ASIR(per 100,000)
1990 2000 2010 2020 2030 2040
year

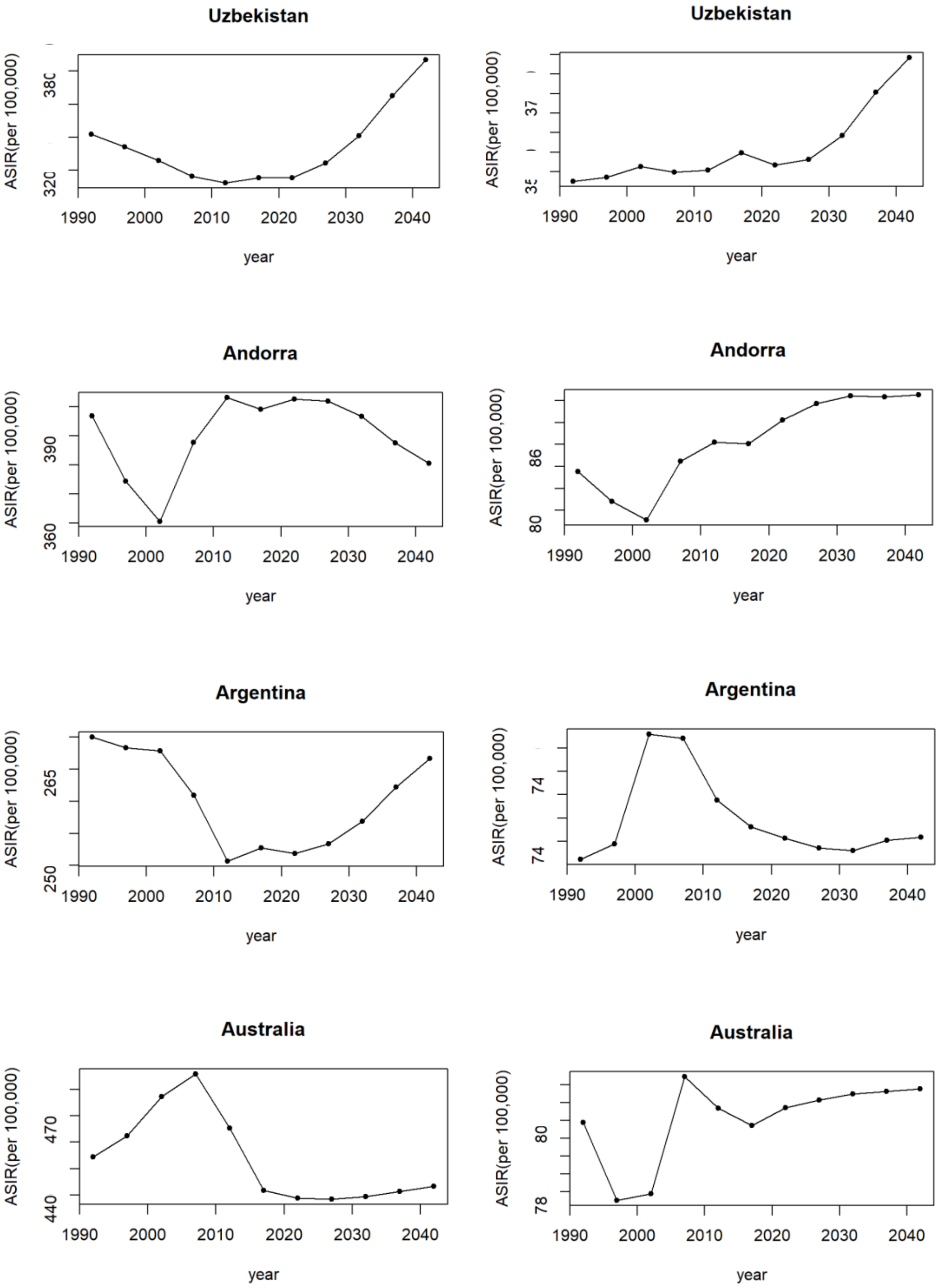
Uzbekistan
ASIR(per 100,000)
320
380
1990
2000
2010
2020
2030
2040
year
Uzbekistan
ASIR(per 100,000)
37
Andorra
ASIR(per 100,000)
360
390
year
Andorra
ASIR(per 100,000)
80
86
year
Argentina
ASIR(per 100,000)
250
265
year
Argentina
ASIR(per 100,000)
74
74
74
year
Australia
ASIR(per 100,000)
440
470
year
Australia
ASIR(per 100,000)
78
80
year

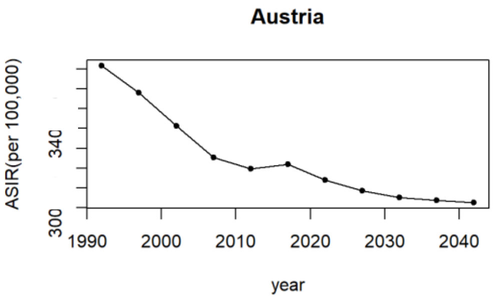
Austria
ASIR(per 100,000)
300
340
1990 2000 2010 2020 2030 2040
year

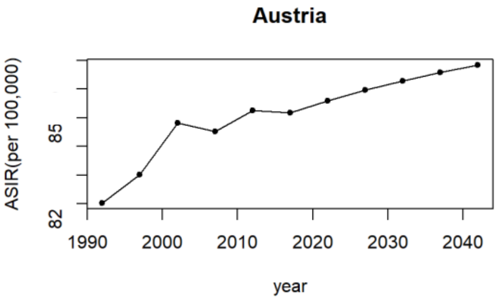
Austria
ASIR(per 100,000)
82
85
1990 2000 2010 2020 2030 2040
year

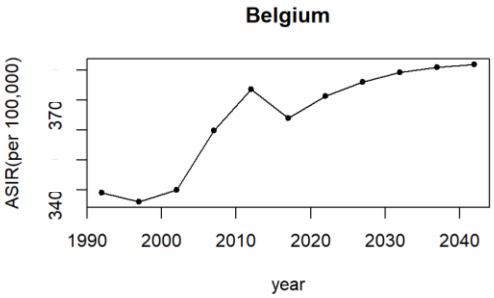
Belgium
ASIR(per 100,000)
340
370
1990 2000 2010 2020 2030 2040
year

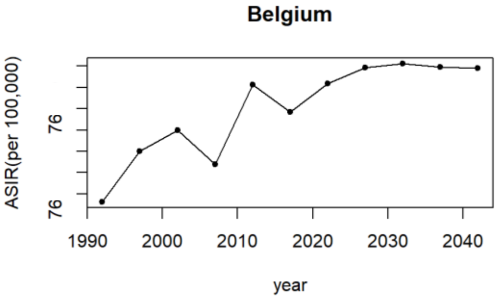
Belgium
ASIR(per 100,000)
76
76
1990 2000 2010 2020 2030 2040
year

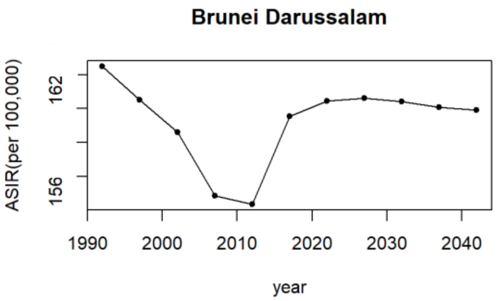
Brunei Darussalam
ASIR(per 100,000)
156
162
1990 2000 2010 2020 2030 2040
year

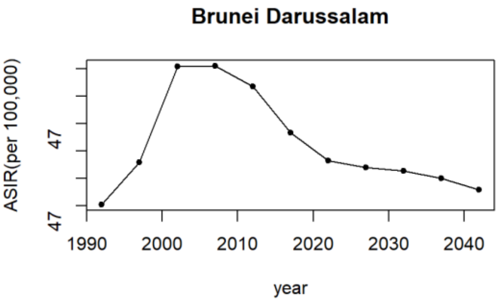
Brunei Darussalam
ASIR(per 100,000)
47
47
1990 2000 2010 2020 2030 2040
year

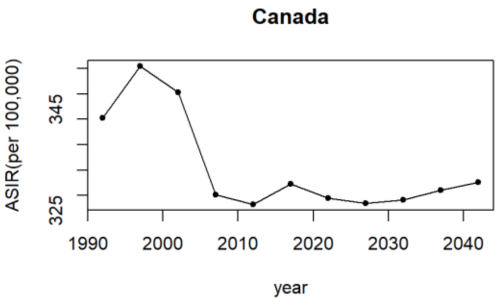
Canada
ASIR(per 100,000)
325
345
1990 2000 2010 2020 2030 2040
year

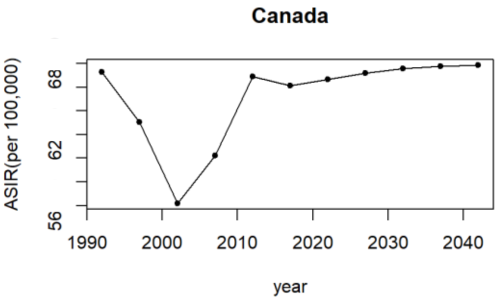
Canada
ASIR(per 100,000)
56
62
68
1990 2000 2010 2020 2030 2040
year

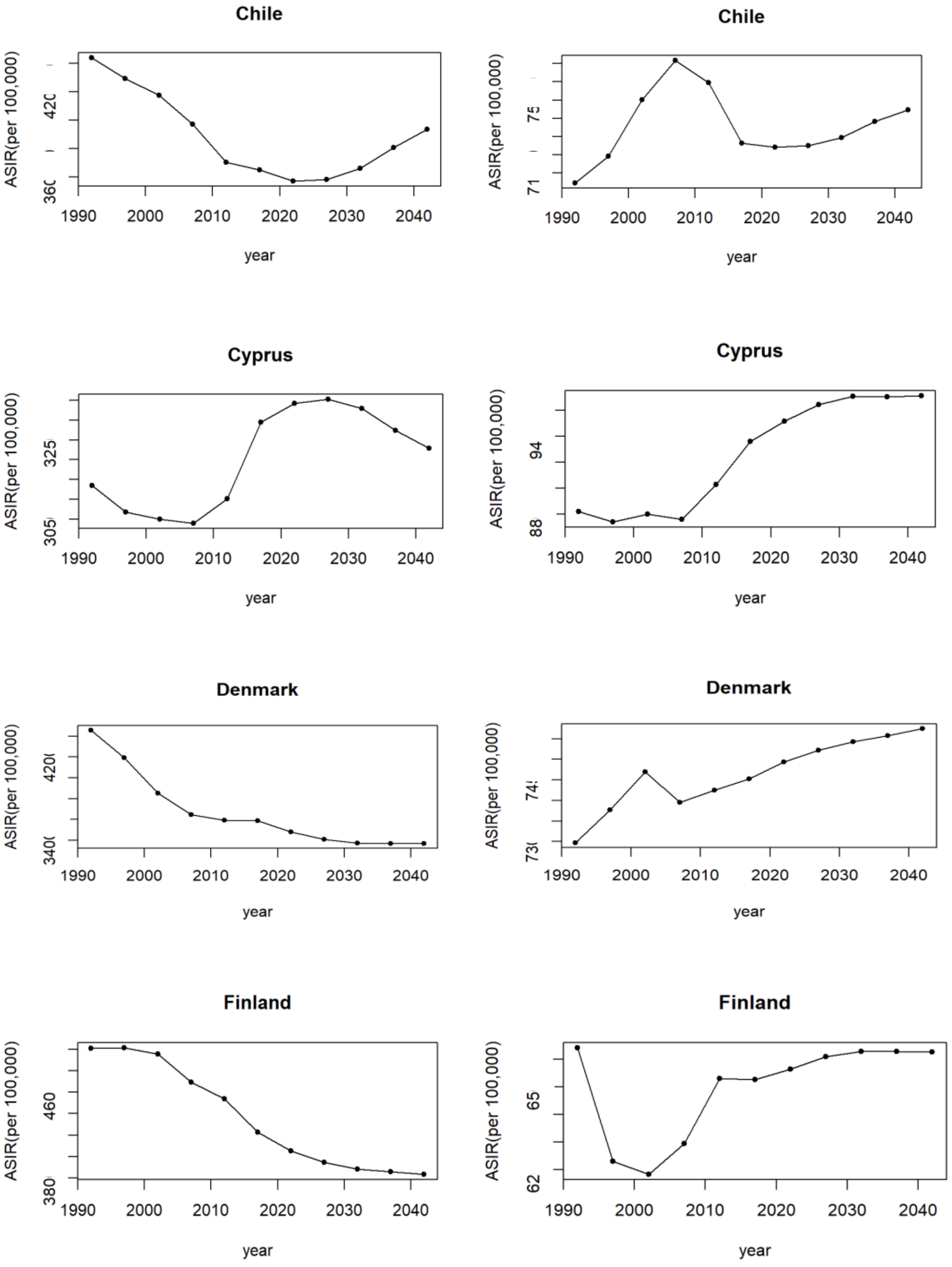
Chile
ASIR(per 100,000)
year
Chile
ASIR(per 100,000)
year
Cyprus
ASIR(per 100,000)
year
Cyprus
ASIR(per 100,000)
year
Denmark
ASIR(per 100,000)
year
Denmark
ASIR(per 100,000)
year
Finland
ASIR(per 100,000)
year
Finland
ASIR(per 100,000)
year

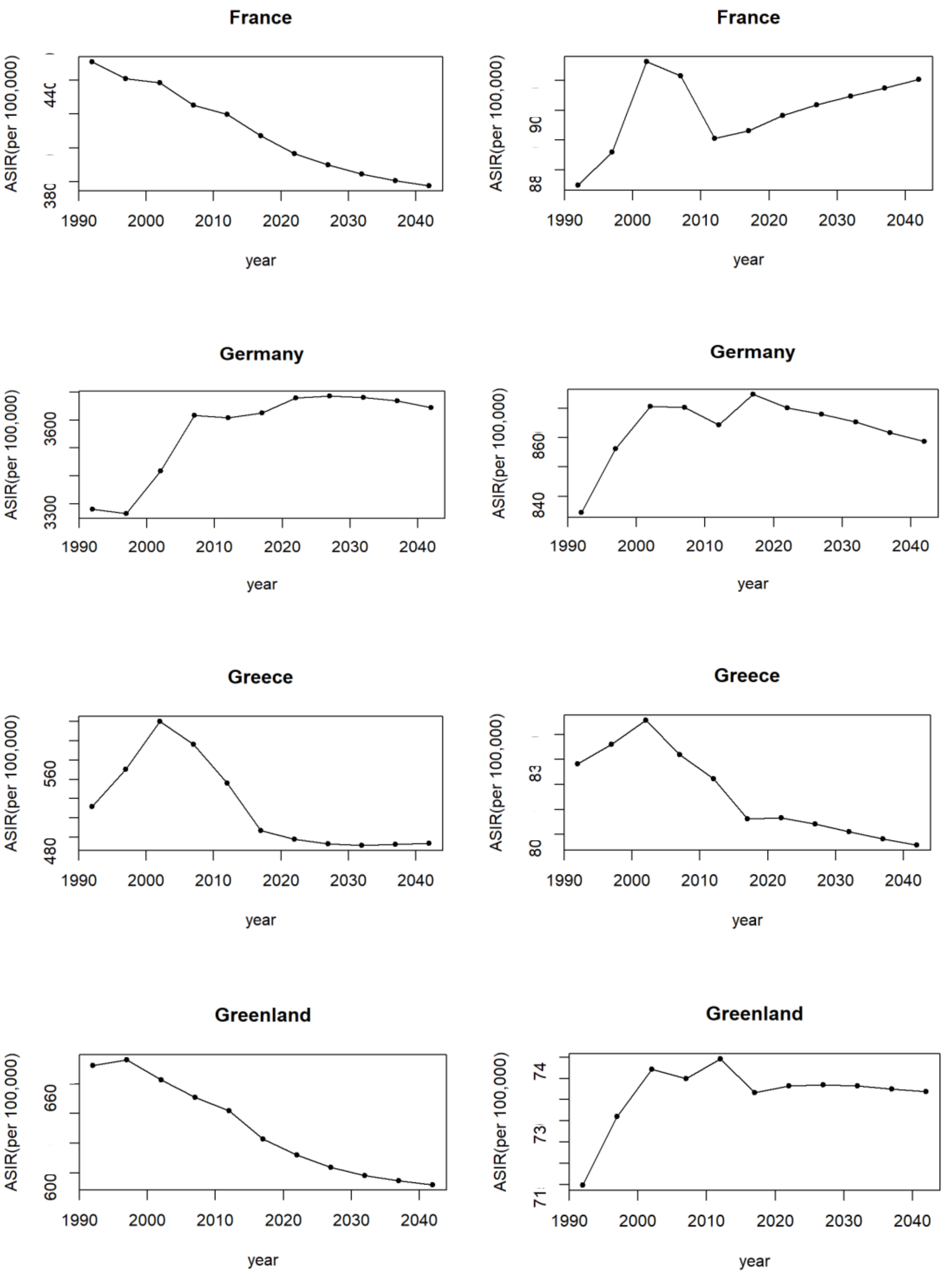
France
ASIR(per 100,000)
1990 2000 2010 2020 2030 2040
year
France
ASIR(per 100,000)
1990 2000 2010 2020 2030 2040
year
Germany
ASIR(per 100,000)
3300
3600
1990 2000 2010 2020 2030 2040
year
Germany
ASIR(per 100,000)
840
860
1990 2000 2010 2020 2030 2040
year
Greece
ASIR(per 100,000)
480
560
1990 2000 2010 2020 2030 2040
year
Greece
ASIR(per 100,000)
80
83
1990 2000 2010 2020 2030 2040
year
Greenland
ASIR(per 100,000)
600
660
1990 2000 2010 2020 2030 2040
year
Greenland
ASIR(per 100,000)
73
74
1990 2000 2010 2020 2030 2040
year

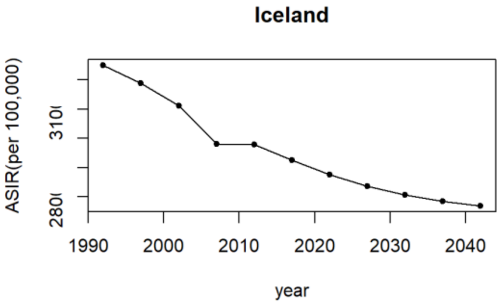
Iceland
ASIR(per 100,000)
310
280
1990
2000
2010
2020
2030
2040
year

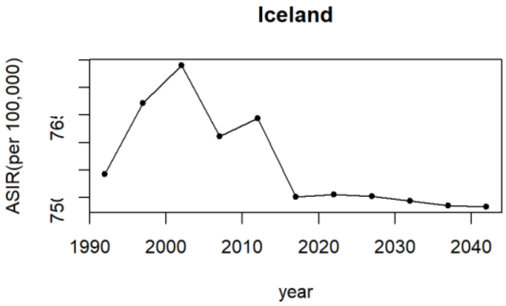
Iceland
ASIR(per 100,000)
76
75
1990
2000
2010
2020
2030
2040
year

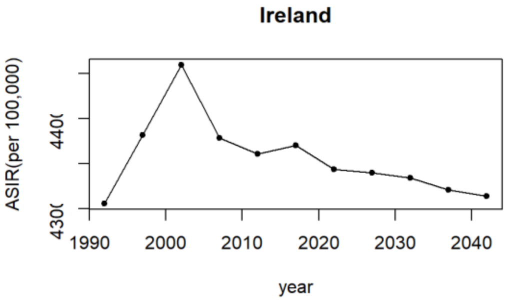
Ireland
ASIR(per 100,000)
440
430
1990
2000
2010
2020
2030
2040
year

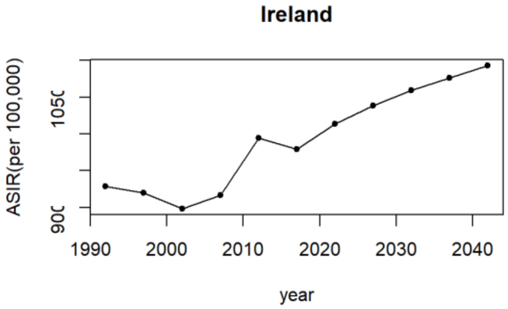
Ireland
ASIR(per 100,000)
105
900
1990
2000
2010
2020
2030
2040
year

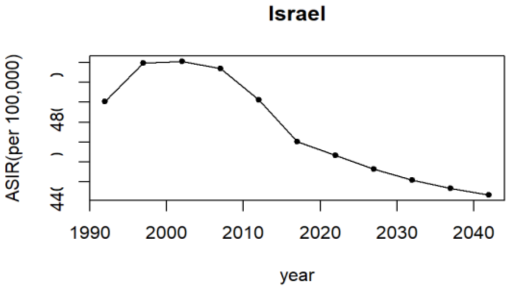
Israel
ASIR(per 100,000)
48
44
1990
2000
2010
2020
2030
2040
year

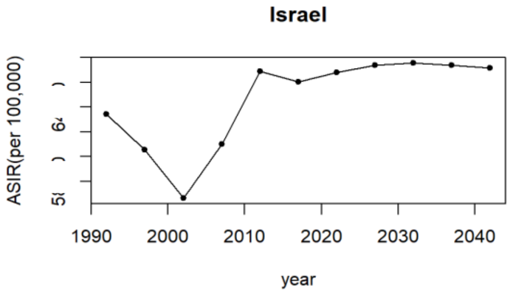
Israel
ASIR(per 100,000)
6
5
1990
2000
2010
2020
2030
2040
year

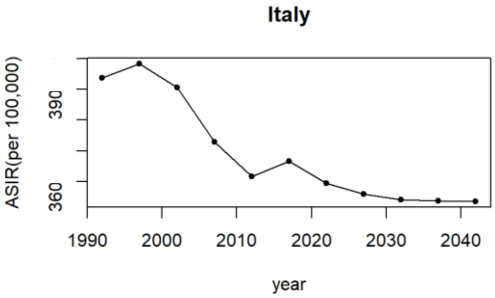
Italy
ASIR(per 100,000)
390
360
1990
2000
2010
2020
2030
2040
year

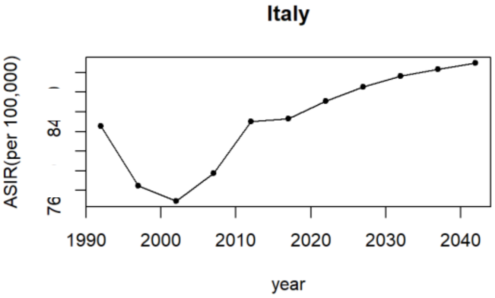
Italy
ASIR(per 100,000)
84
76
1990
2000
2010
2020
2030
2040
year

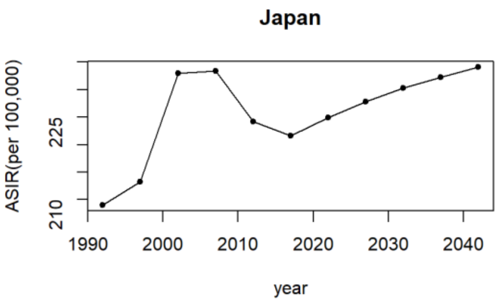
Japan
ASIR(per 100,000)
210
225
1990 2000 2010 2020 2030 2040
year

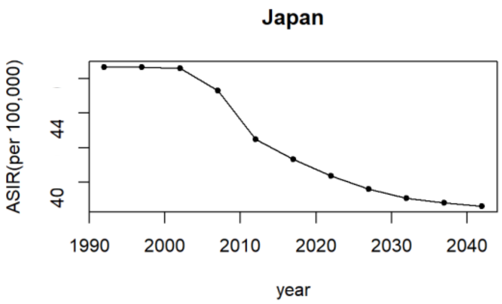
Japan
ASIR(per 100,000)
40
44
1990 2000 2010 2020 2030 2040
year

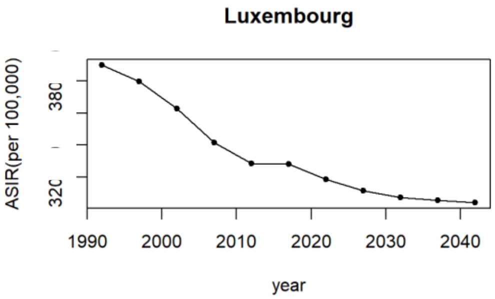
Luxembourg
ASIR(per 100,000)
320
1990 2000 2010 2020 2030 2040
year

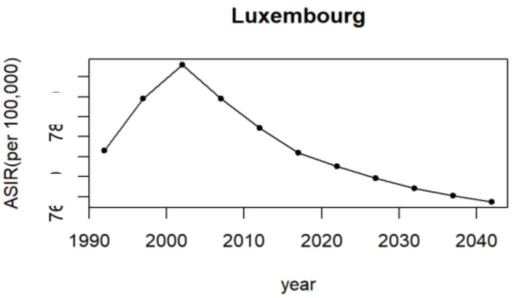
Luxembourg
ASIR(per 100,000)
1990 2000 2010 2020 2030 2040
year

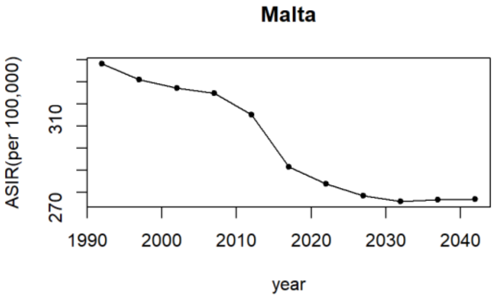
Malta
ASIR(per 100,000)
270
310
1990 2000 2010 2020 2030 2040
year

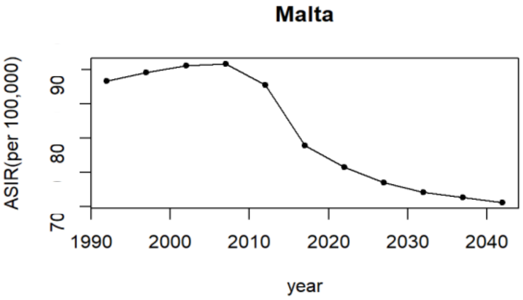
Malta
ASIR(per 100,000)
70
80
90
1990 2000 2010 2020 2030 2040
year

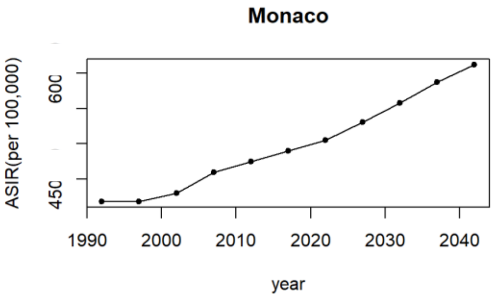
Monaco
ASIR(per 100,000)
450
1990 2000 2010 2020 2030 2040
year

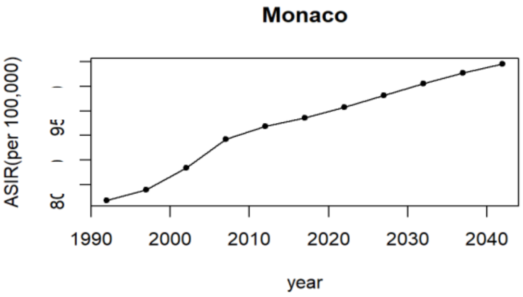
Monaco
ASIR(per 100,000)
1990 2000 2010 2020 2030 2040
year

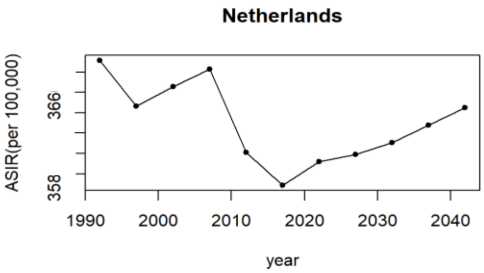
Netherlands
ASIR(per 100,000)
366
358
1990
2000
2010
2020
2030
2040
year

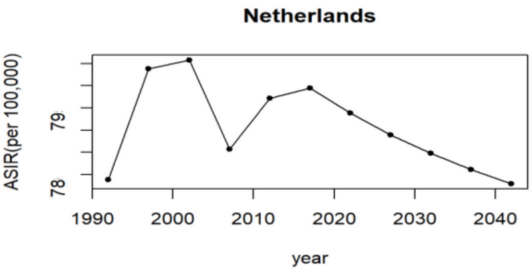
Netherlands
ASIR(per 100,000)
79
78
1990
2000
2010
2020
2030
2040
year

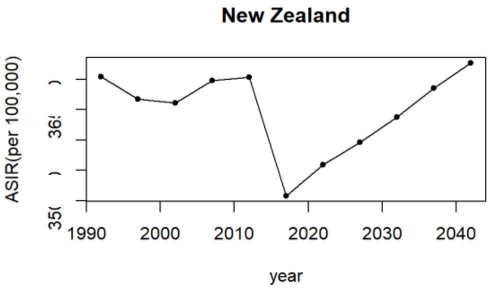
New Zealand
ASIR(per 100,000)
1990
2000
2010
2020
2030
2040
year

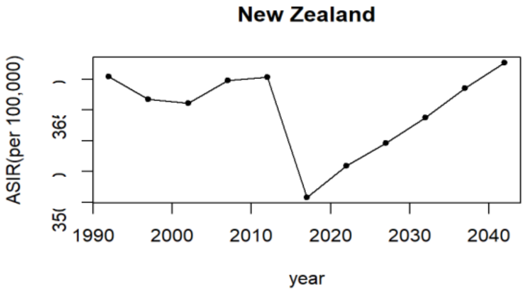
New Zealand
ASIR(per 100,000)
1990
2000
2010
2020
2030
2040
year

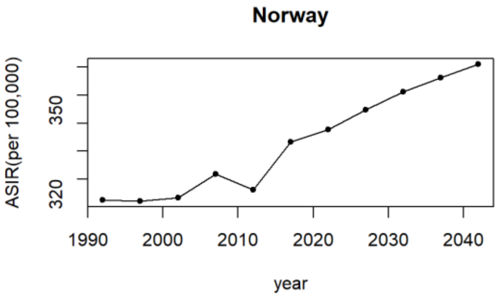
Norway
ASIR(per 100,000)
350
320
1990
2000
2010
2020
2030
2040
year

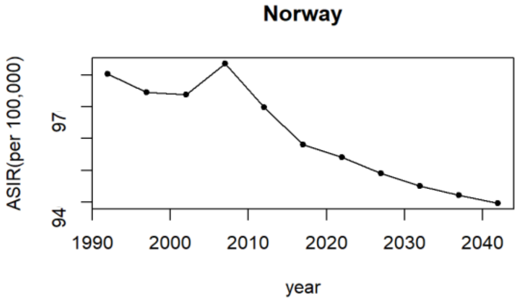
Norway
ASIR(per 100,000)
97
94
1990
2000
2010
2020
2030
2040
year

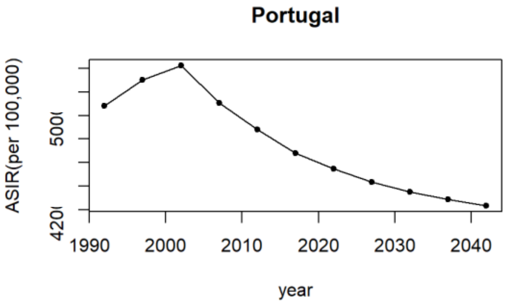
Portugal
ASIR(per 100,000)
1990
2000
2010
2020
2030
2040
year

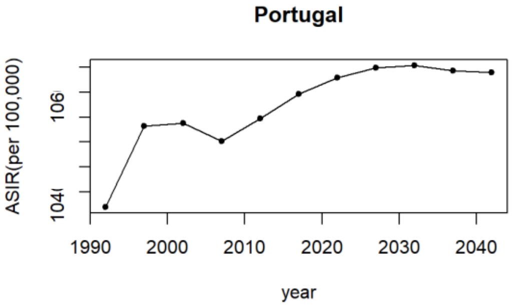
Portugal
ASIR(per 100,000)
106
106
1990
2000
2010
2020
2030
2040
year

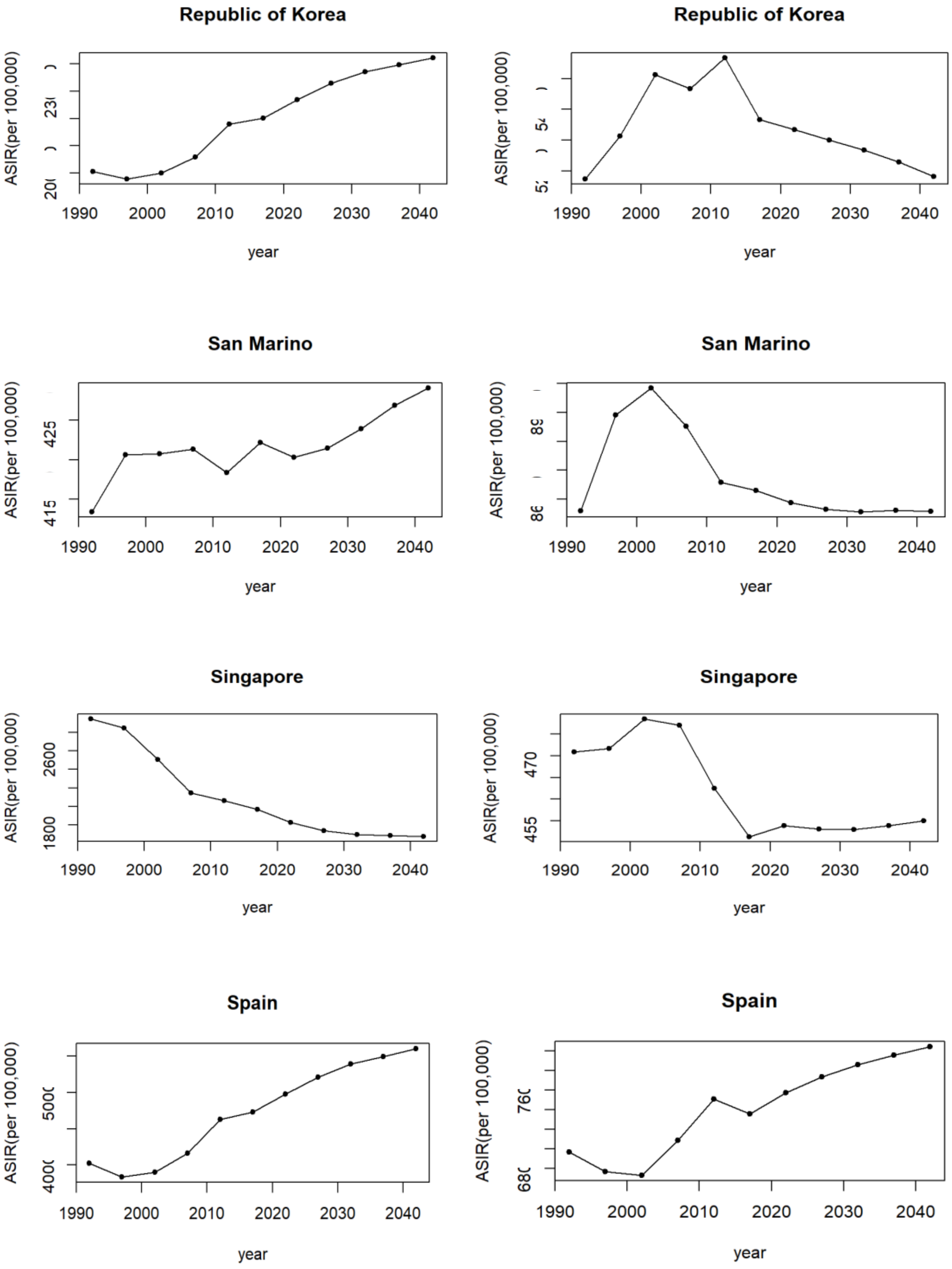
Republic of Korea
ASIR(per 100,000)
year
1990 2000 2010 2020 2030 2040
Republic of Korea
ASIR(per 100,000)
year
1990 2000 2010 2020 2030 2040
San Marino
ASIR(per 100,000)
415
425
year
1990 2000 2010 2020 2030 2040
San Marino
ASIR(per 100,000)
year
1990 2000 2010 2020 2030 2040
Singapore
ASIR(per 100,000)
1800
2600
year
1990 2000 2010 2020 2030 2040
Singapore
ASIR(per 100,000)
455
470
year
1990 2000 2010 2020 2030 2040
Spain
ASIR(per 100,000)
year
1990 2000 2010 2020 2030 2040
Spain
ASIR(per 100,000)
year
1990 2000 2010 2020 2030 2040

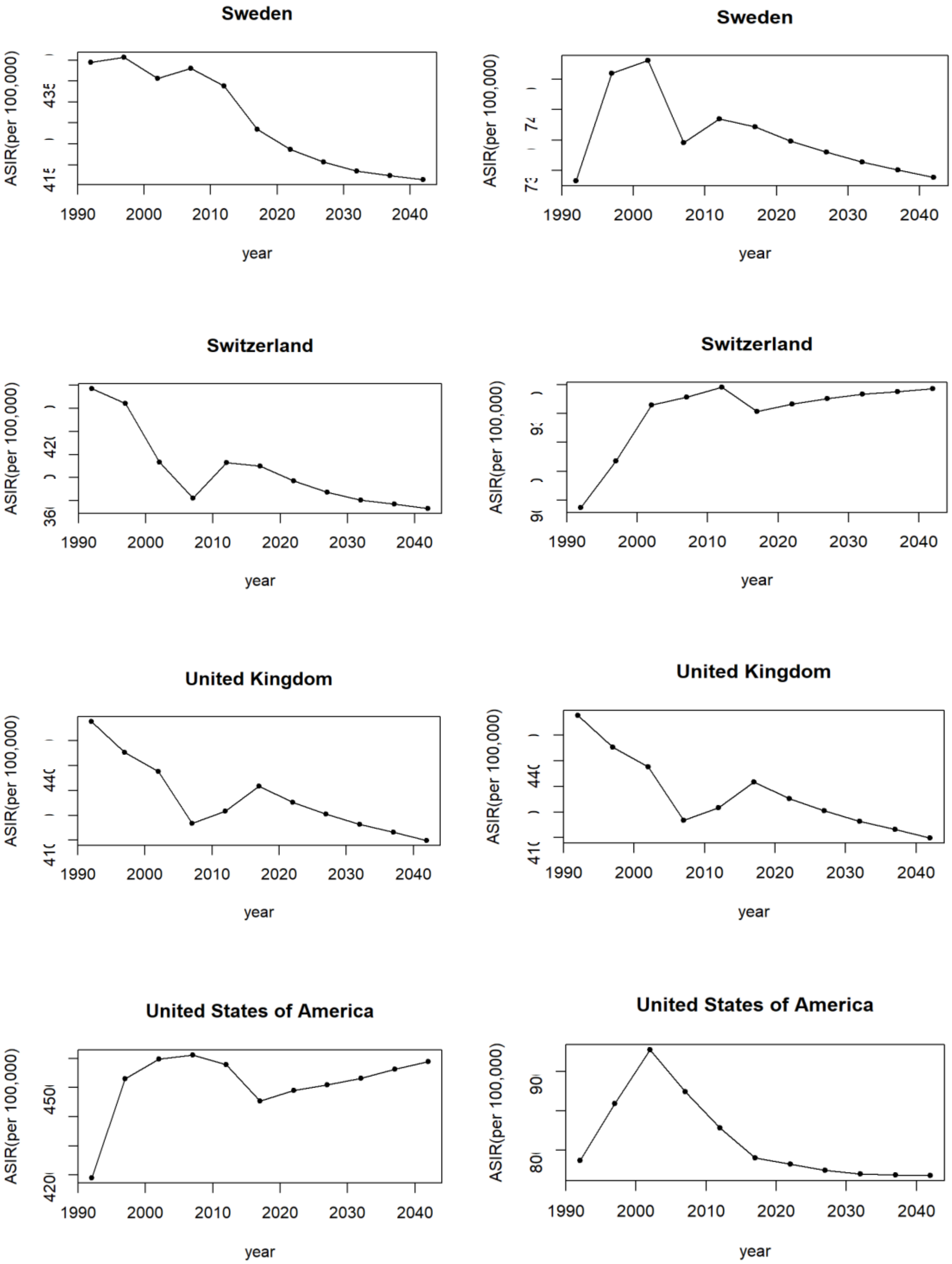
Sweden
ASIR(per 100,000)
year
Sweden
ASIR(per 100,000)
year
Switzerland
ASIR(per 100,000)
year
Switzerland
ASIR(per 100,000)
year
United Kingdom
ASIR(per 100,000)
year
United Kingdom
ASIR(per 100,000)
year
United States of America
ASIR(per 100,000)
year
United States of America
ASIR(per 100,000)
year
1990
2000
2010
2020
2030
2040

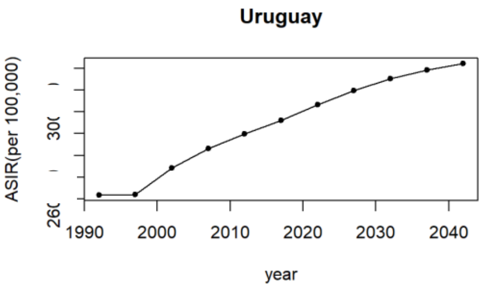
Uruguay
ASIR(per 100,000)
1990
2000
2010
2020
2030
2040
year
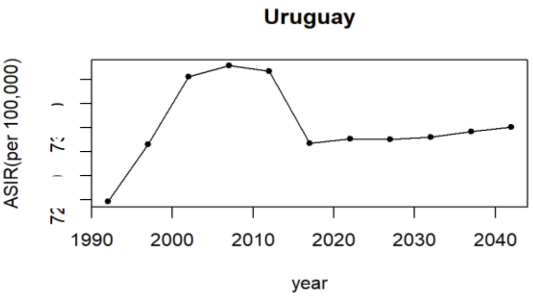
Uruguay
ASIR(per 100,000)
1990
2000
2010
2020
2030
2040
year
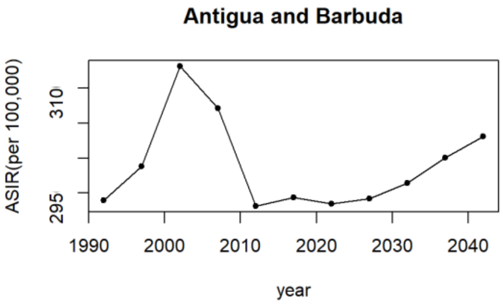
Antigua and Barbuda
ASIR(per 100,000)
310
295
1990
2000
2010
2020
2030
2040
year
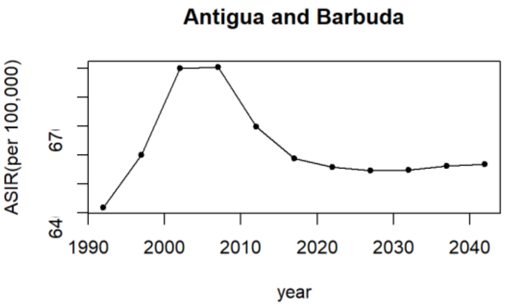
Antigua and Barbuda
ASIR(per 100,000)
1990
2000
2010
2020
2030
2040
year
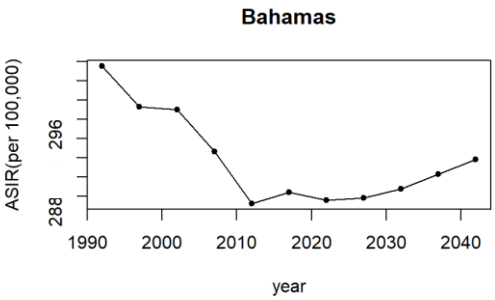
Bahamas
ASIR(per 100,000)
296
288
1990
2000
2010
2020
2030
2040
year
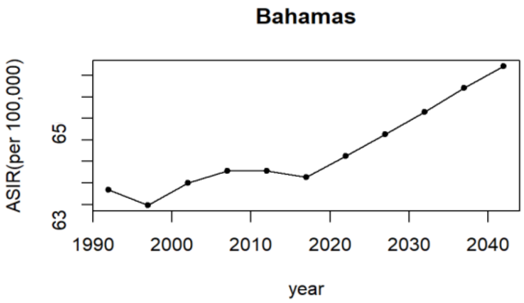
Bahamas
ASIR(per 100,000)
65
63
1990
2000
2010
2020
2030
2040
year
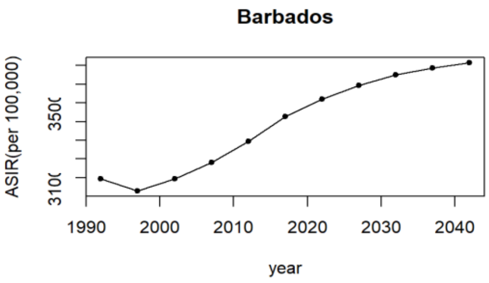
Barbados
ASIR(per 100,000)
1990
2000
2010
2020
2030
2040
year
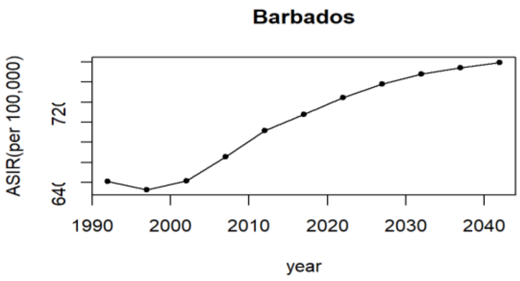
Barbados
ASIR(per 100,000)
1990
2000
2010
2020
2030
2040
year

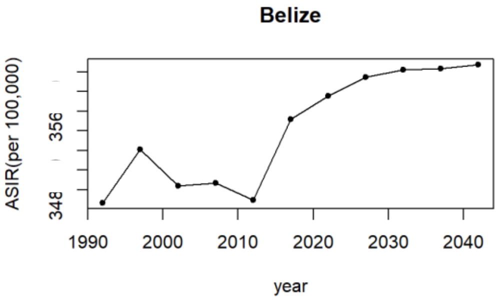
Belize
ASIR(per 100,000)
348
356
1990
2000
2010
2020
2030
2040
year

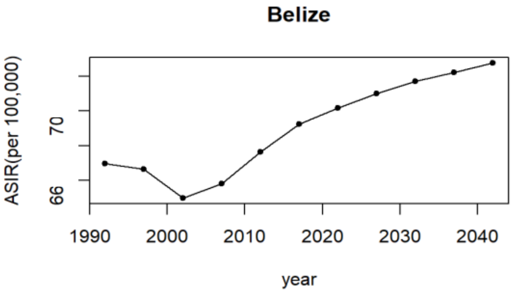
Belize
ASIR(per 100,000)
66
70
1990
2000
2010
2020
2030
2040
year

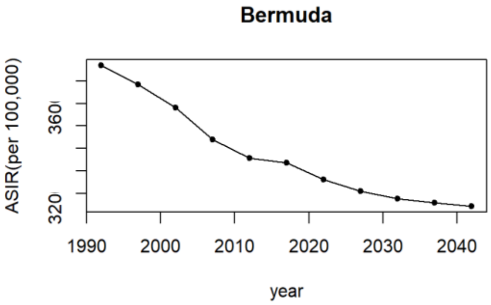
Bermuda
ASIR(per 100,000)
320
360
1990
2000
2010
2020
2030
2040
year

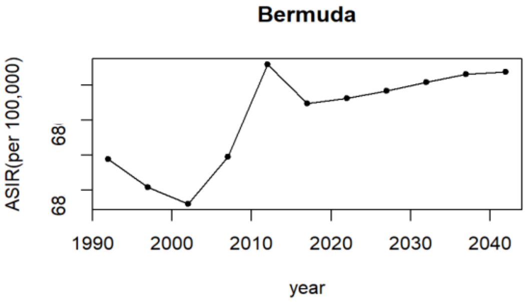
Bermuda
ASIR(per 100,000)
68
68
1990
2000
2010
2020
2030
2040
year

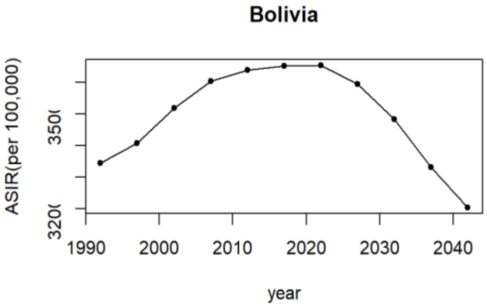
Bolivia
ASIR(per 100,000)
320
350
1990
2000
2010
2020
2030
2040
year

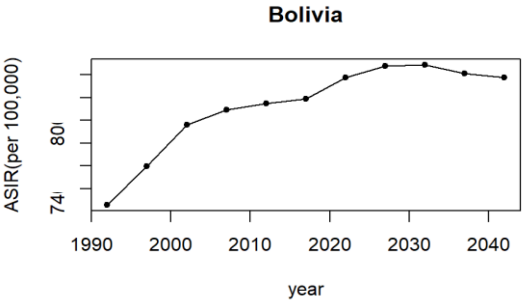
Bolivia
ASIR(per 100,000)
74
80
1990
2000
2010
2020
2030
2040
year

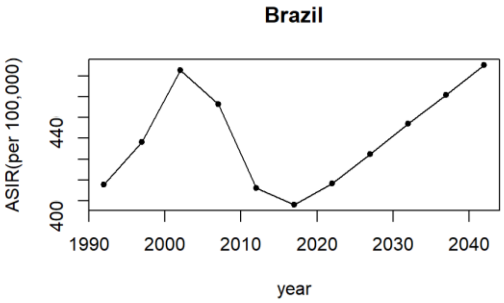
Brazil
ASIR(per 100,000)
400
440
1990
2000
2010
2020
2030
2040
year

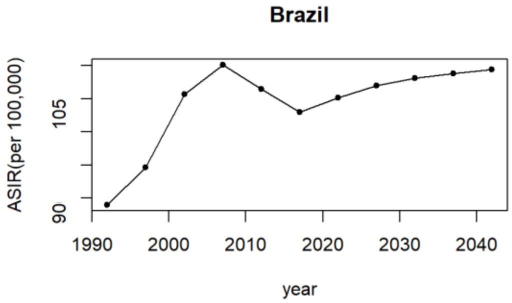
Brazil
ASIR(per 100,000)
90
105
105
1990
2000
2010
2020
2030
2040
year

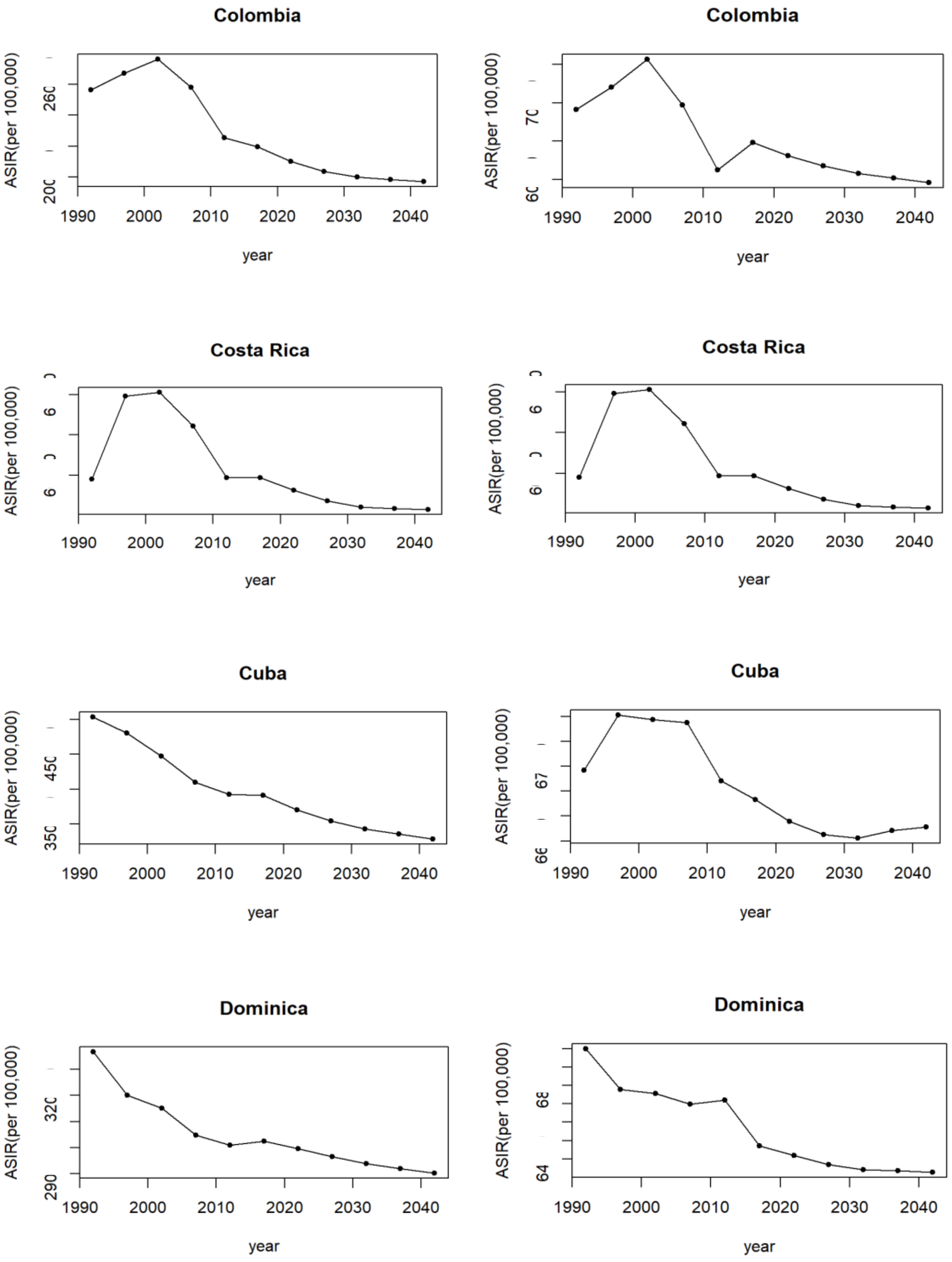
Colombia
Colombia
Costa Rica
Costa Rica
Cuba
Cuba
Dominica
Dominica
ASIR(per 100,000)
year
1990
2000
2010
2020
2030
2040

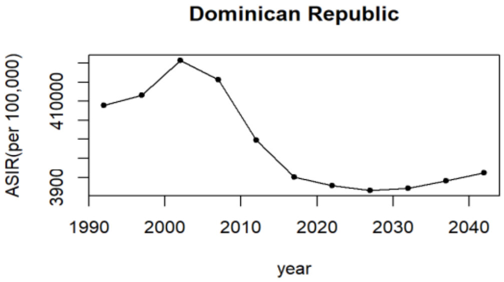
Dominican Republic
ASIR(per 100,000)
410000
3900
1990 2000 2010 2020 2030 2040
year

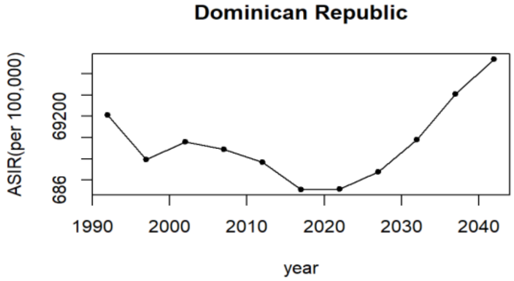
Dominican Republic
ASIR(per 100,000)
69200
686
1990 2000 2010 2020 2030 2040
year

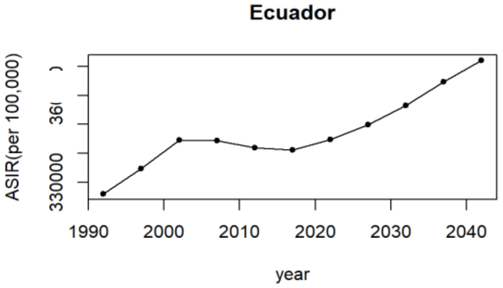
Ecuador
ASIR(per 100,000)
330000
1990 2000 2010 2020 2030 2040
year

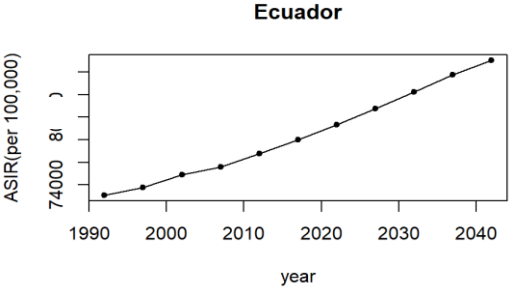
Ecuador
ASIR(per 100,000)
74000
1990 2000 2010 2020 2030 2040
year

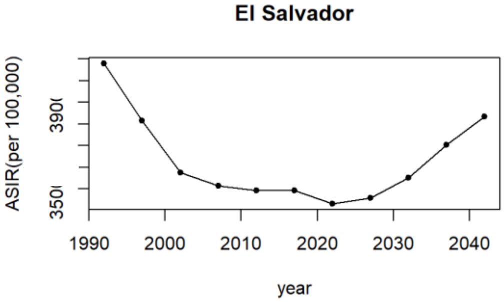
El Salvador
ASIR(per 100,000)
1990 2000 2010 2020 2030 2040
year

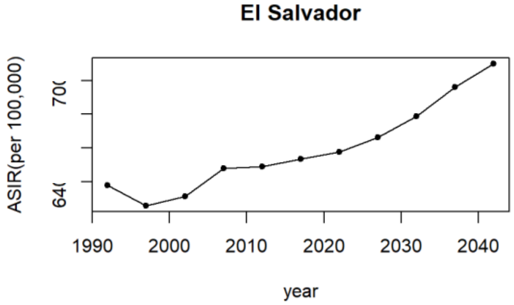
El Salvador
ASIR(per 100,000)
1990 2000 2010 2020 2030 2040
year

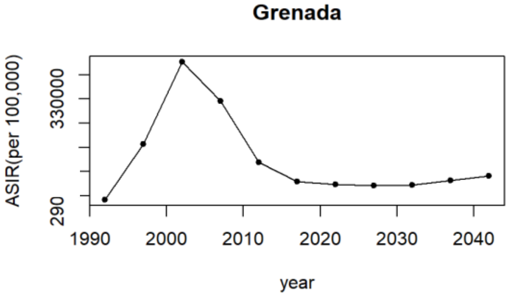
Grenada
ASIR(per 100,000)
330000
290
1990 2000 2010 2020 2030 2040
year

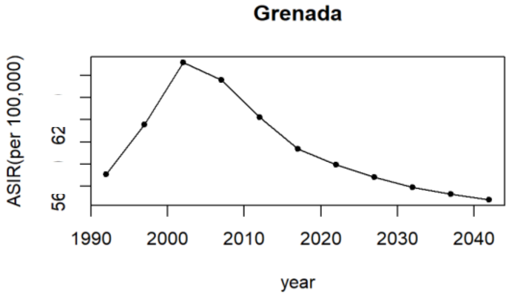
Grenada
ASIR(per 100,000)
62
1990 2000 2010 2020 2030 2040
year

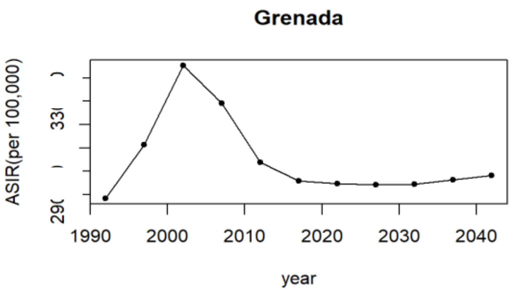
Grenada
ASIR(per 100,000)
1990 2000 2010 2020 2030 2040
year

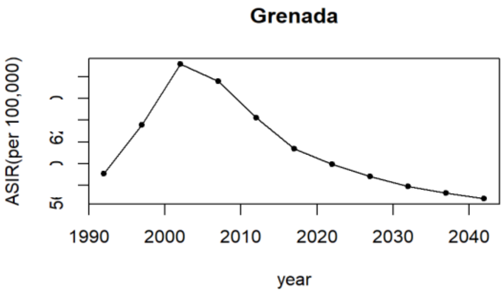
Grenada
ASIR(per 100,000)
1990 2000 2010 2020 2030 2040
year

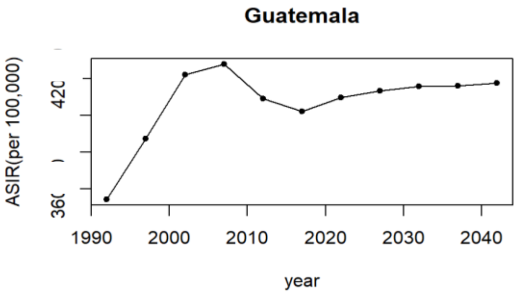
Guatemala
ASIR(per 100,000)
1990 2000 2010 2020 2030 2040
year

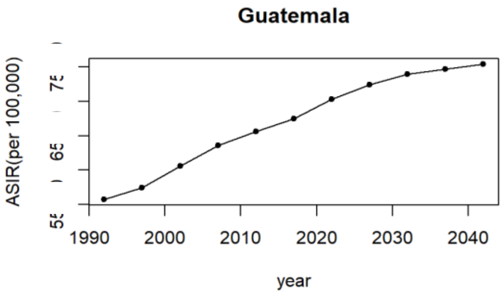
Guatemala
ASIR(per 100,000)
1990 2000 2010 2020 2030 2040
year

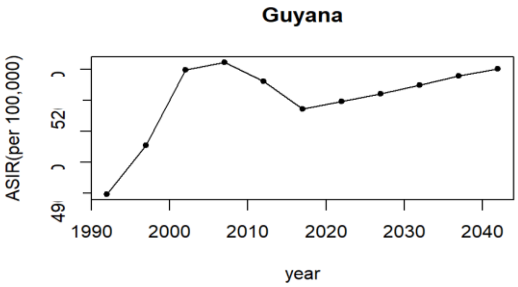
Guyana
ASIR(per 100,000)
1990 2000 2010 2020 2030 2040
year

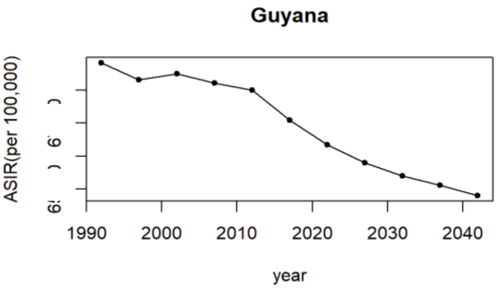
Guyana
ASIR(per 100,000)
1990 2000 2010 2020 2030 2040
year

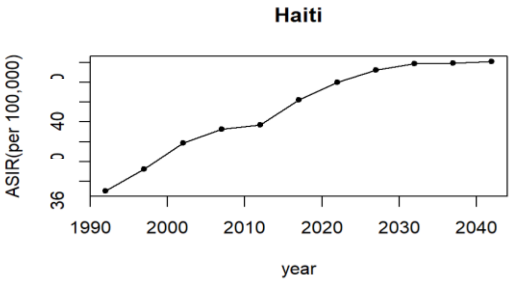
Haiti
ASIR(per 100,000)
36
40
1990 2000 2010 2020 2030 2040
year

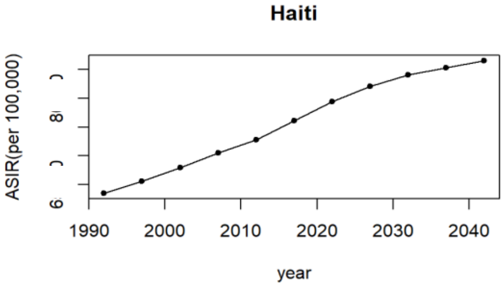
Haiti
ASIR(per 100,000)
1990 2000 2010 2020 2030 2040
year

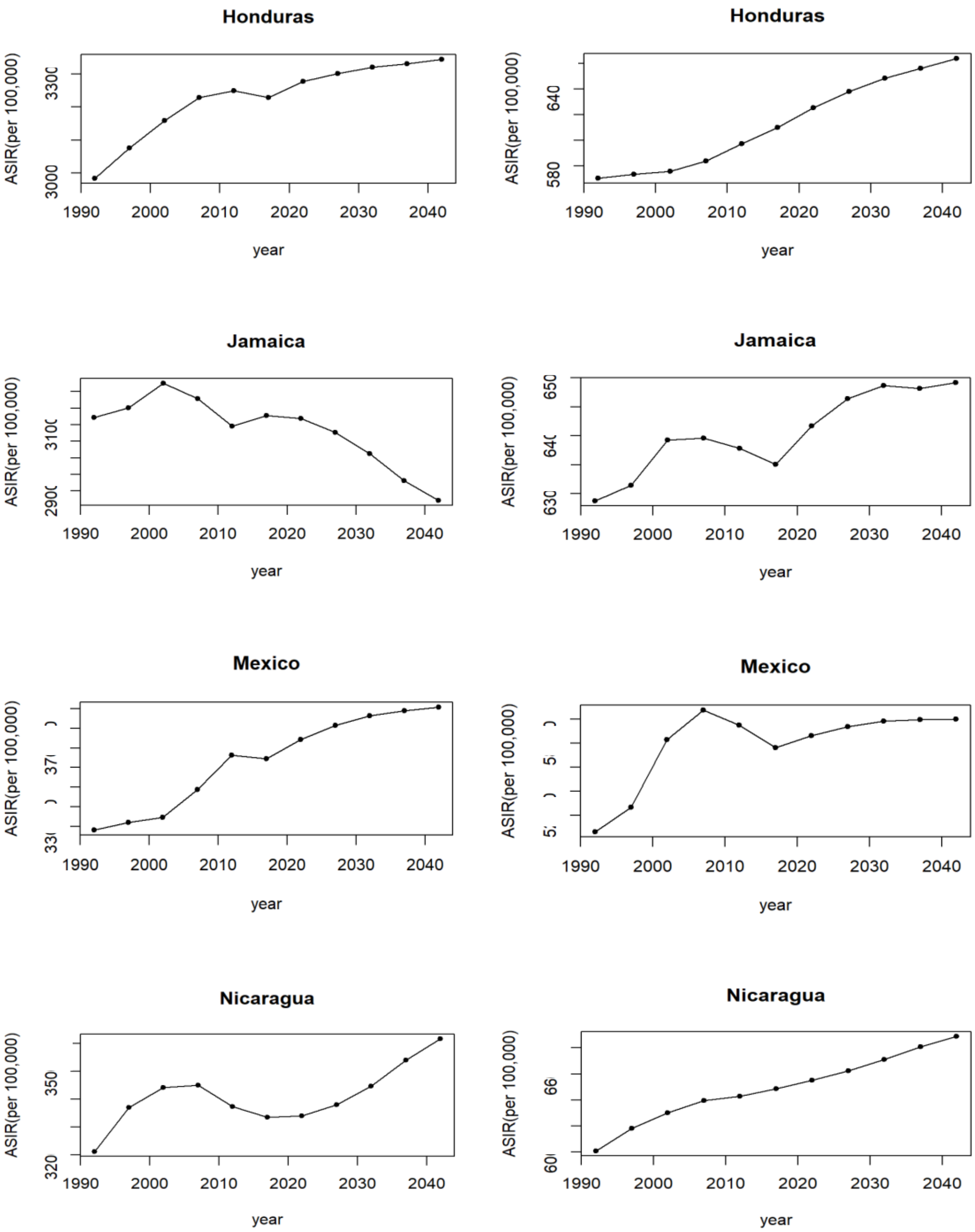

Honduras
ASIR(per 100,000)
3000
year
1990
2000
2010
2020
2030
2040
Honduras
ASIR(per 100,000)
580
640
year
Jamaica
ASIR(per 100,000)
year
Jamaica
ASIR(per 100,000)
630
650
year
Mexico
ASIR(per 100,000)
year
Mexico
ASIR(per 100,000)
year
Nicaragua
ASIR(per 100,000)
320
350
year
Nicaragua
ASIR(per 100,000)
66
year

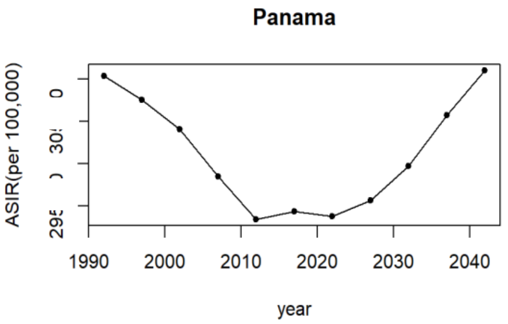
Panama
ASIR(per 100,000)
1990 2000 2010 2020 2030 2040
year

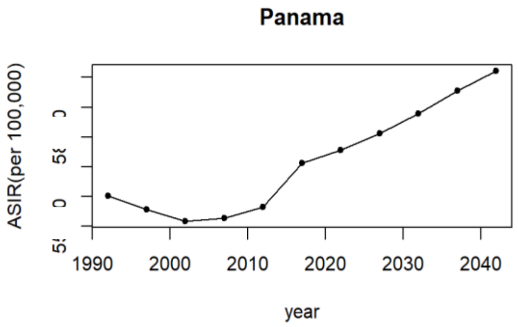
Panama
ASIR(per 100,000)
1990 2000 2010 2020 2030 2040
year

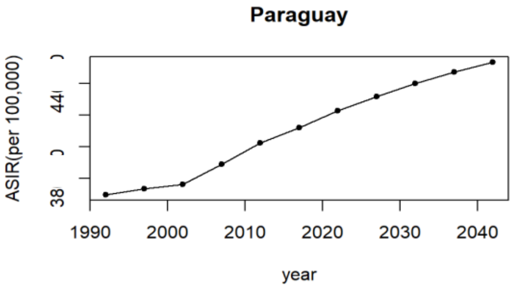
Paraguay
ASIR(per 100,000)
38
1990 2000 2010 2020 2030 2040
year

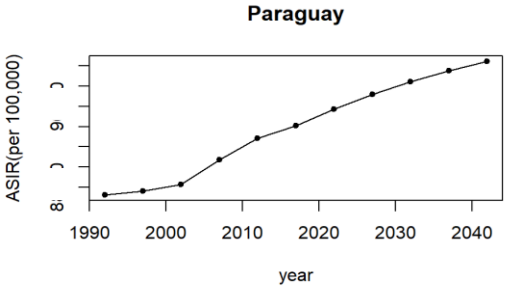
Paraguay
ASIR(per 100,000)
1990 2000 2010 2020 2030 2040
year

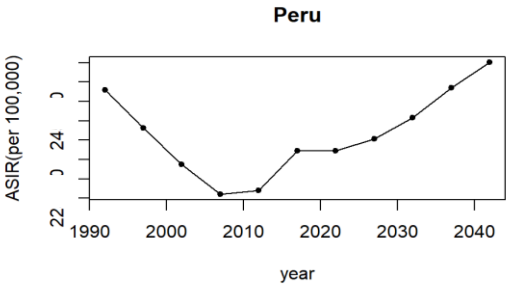
Peru
ASIR(per 100,000)
22
24
1990 2000 2010 2020 2030 2040
year

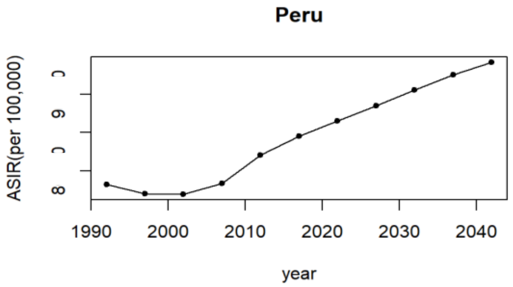
Peru
ASIR(per 100,000)
8
1990 2000 2010 2020 2030 2040
year

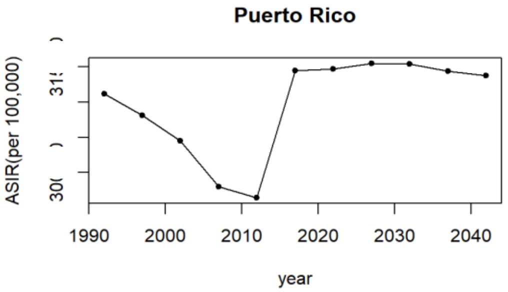
Puerto Rico
ASIR(per 100,000)
1990 2000 2010 2020 2030 2040
year

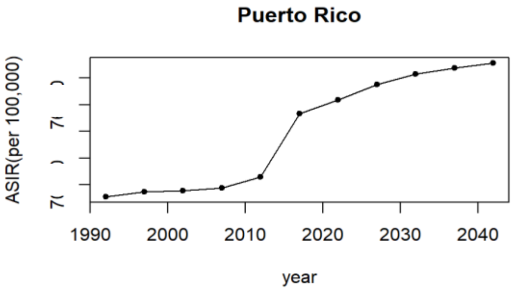
Puerto Rico
ASIR(per 100,000)
1990 2000 2010 2020 2030 2040
year

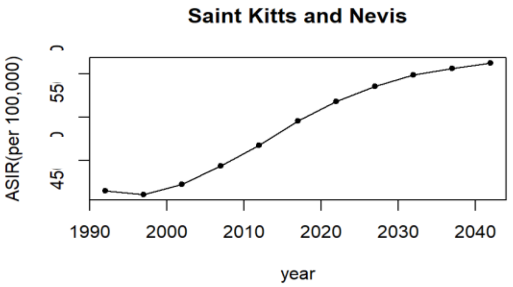
Saint Kitts and Nevis
ASIR(per 100,000)
year
1990
2000
2010
2020
2030
2040

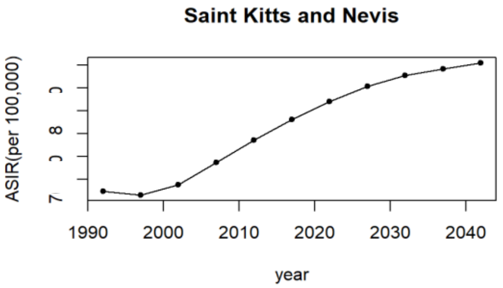
Saint Kitts and Nevis
ASIR(per 100,000)
year
1990
2000
2010
2020
2030
2040

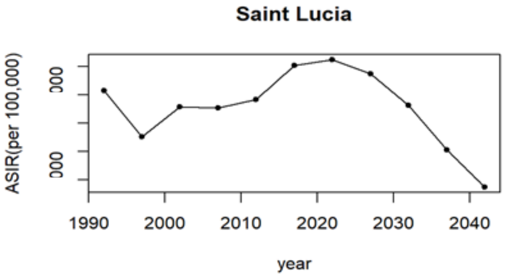
Saint Lucia
ASIR(per 100,000)
year
1990
2000
2010
2020
2030
2040

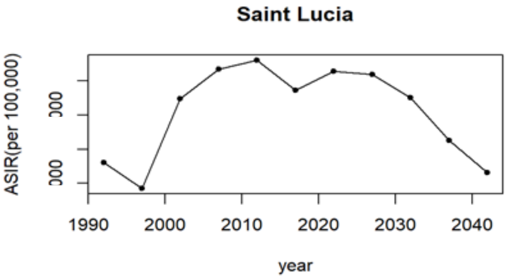
Saint Lucia
ASIR(per 100,000)
year
1990
2000
2010
2020
2030
2040

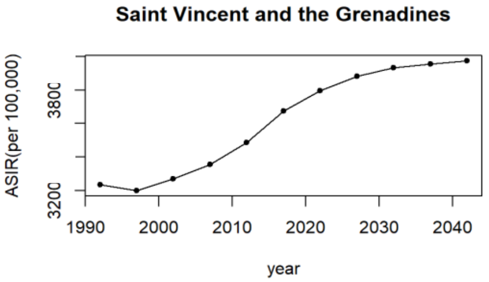
Saint Vincent and the Grenadines
ASIR(per 100,000)
3200
year
1990
2000
2010
2020
2030
2040

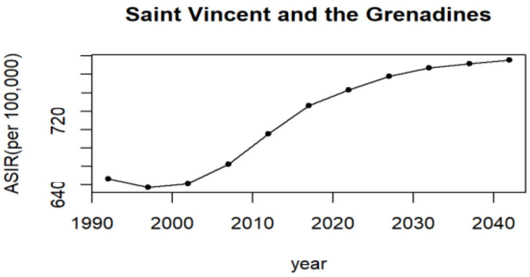
Saint Vincent and the Grenadines
ASIR(per 100,000)
720
640
year
1990
2000
2010
2020
2030
2040

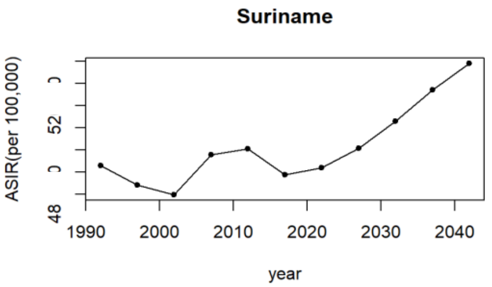
Suriname
ASIR(per 100,000)
52
48
year
1990
2000
2010
2020
2030
2040

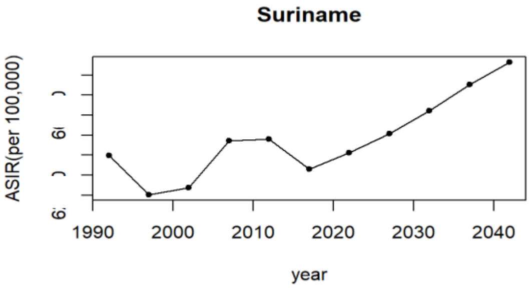
Suriname
ASIR(per 100,000)
year
1990
2000
2010
2020
2030
2040

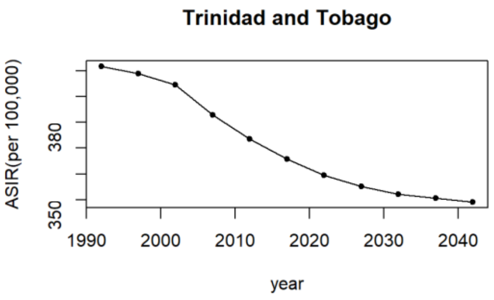
Trinidad and Tobago
ASIR(per 100,000)
350
380
1990
2000
2010
2020
2030
2040
year

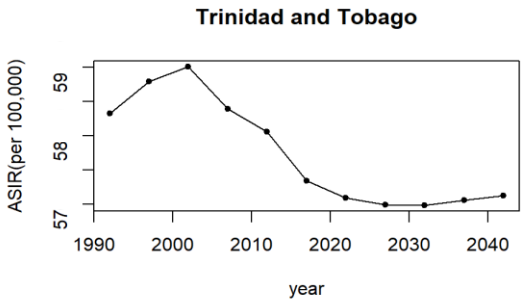
Trinidad and Tobago
ASIR(per 100,000)
57
58
59
1990
2000
2010
2020
2030
2040
year

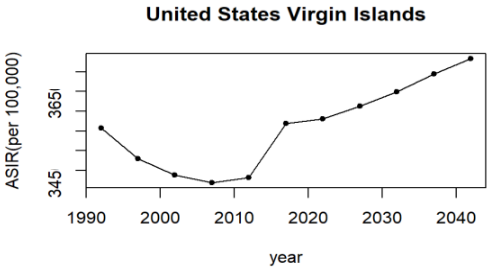
United States Virgin Islands
ASIR(per 100,000)
345
1990
2000
2010
2020
2030
2040
year

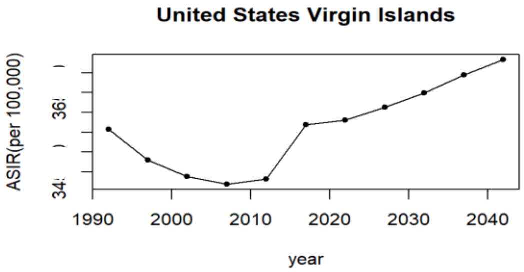
United States Virgin Islands
ASIR(per 100,000)
1990
2000
2010
2020
2030
2040
year

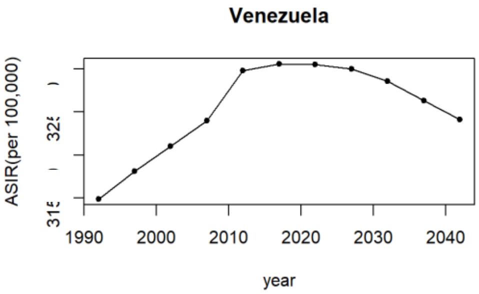
Venezuela
ASIR(per 100,000)
1990
2000
2010
2020
2030
2040
year

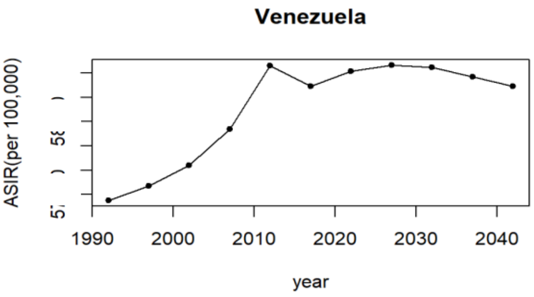
Venezuela
ASIR(per 100,000)
1990
2000
2010
2020
2030
2040
year

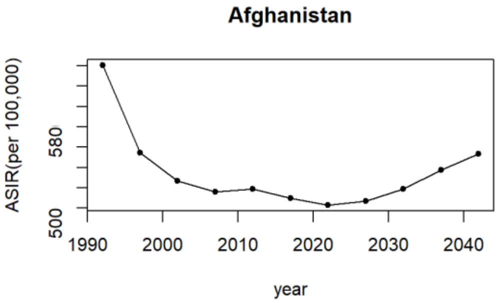
Afghanistan
ASIR(per 100,000)
500
580
1990
2000
2010
2020
2030
2040
year

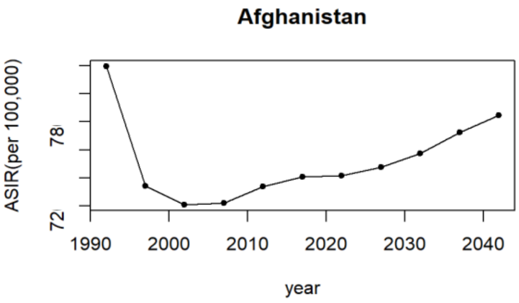
Afghanistan
ASIR(per 100,000)
72
1990
2000
2010
2020
2030
2040
year

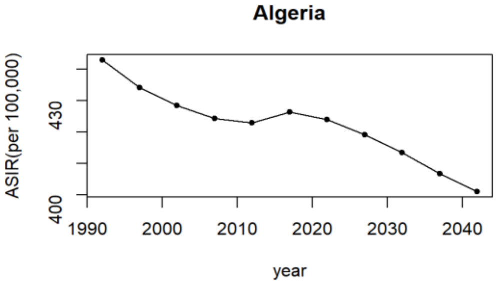
Algeria
ASIR(per 100,000)
400
430
1990
2000
2010
2020
2030
2040
year

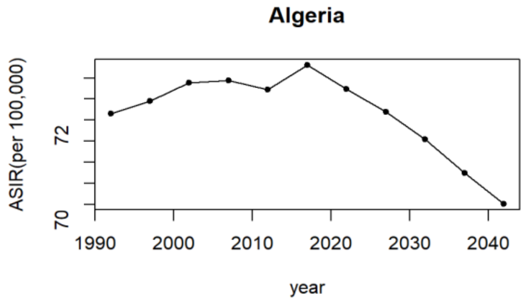
Algeria
ASIR(per 100,000)
70
72
1990
2000
2010
2020
2030
2040
year

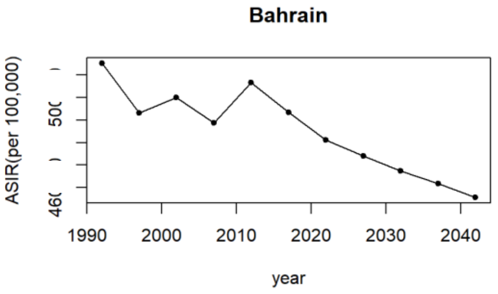
Bahrain
ASIR(per 100,000)
1990
2000
2010
2020
2030
2040
year

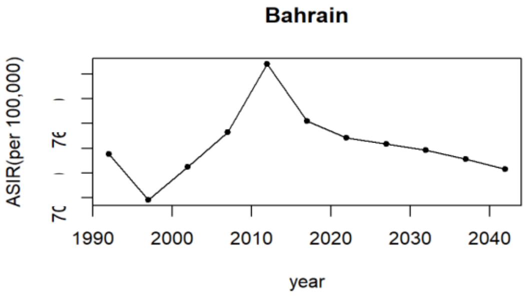
Bahrain
ASIR(per 100,000)
1990
2000
2010
2020
2030
2040
year

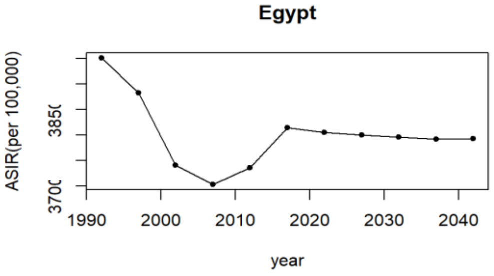
Egypt
ASIR(per 100,000)
1990
2000
2010
2020
2030
2040
year

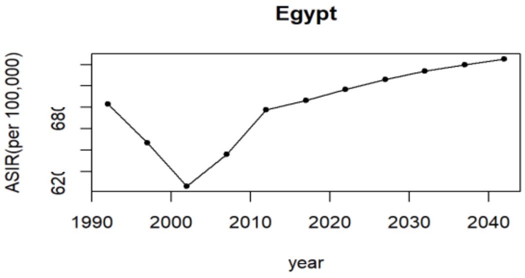
Egypt
ASIR(per 100,000)
1990
2000
2010
2020
2030
2040
year

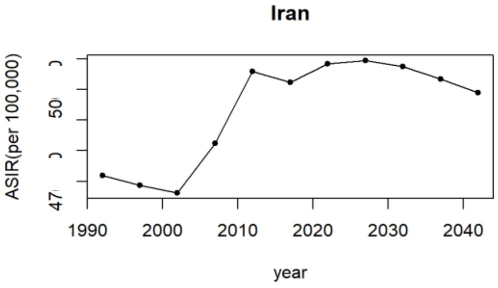
Iran
ASIR(per 100,000)
47
50
1990
2000
2010
2020
2030
2040
year

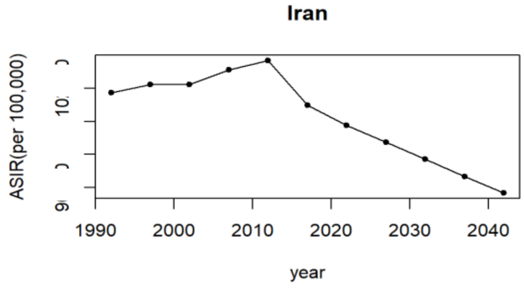
Iran
ASIR(per 100,000)
1990
2000
2010
2020
2030
2040
year

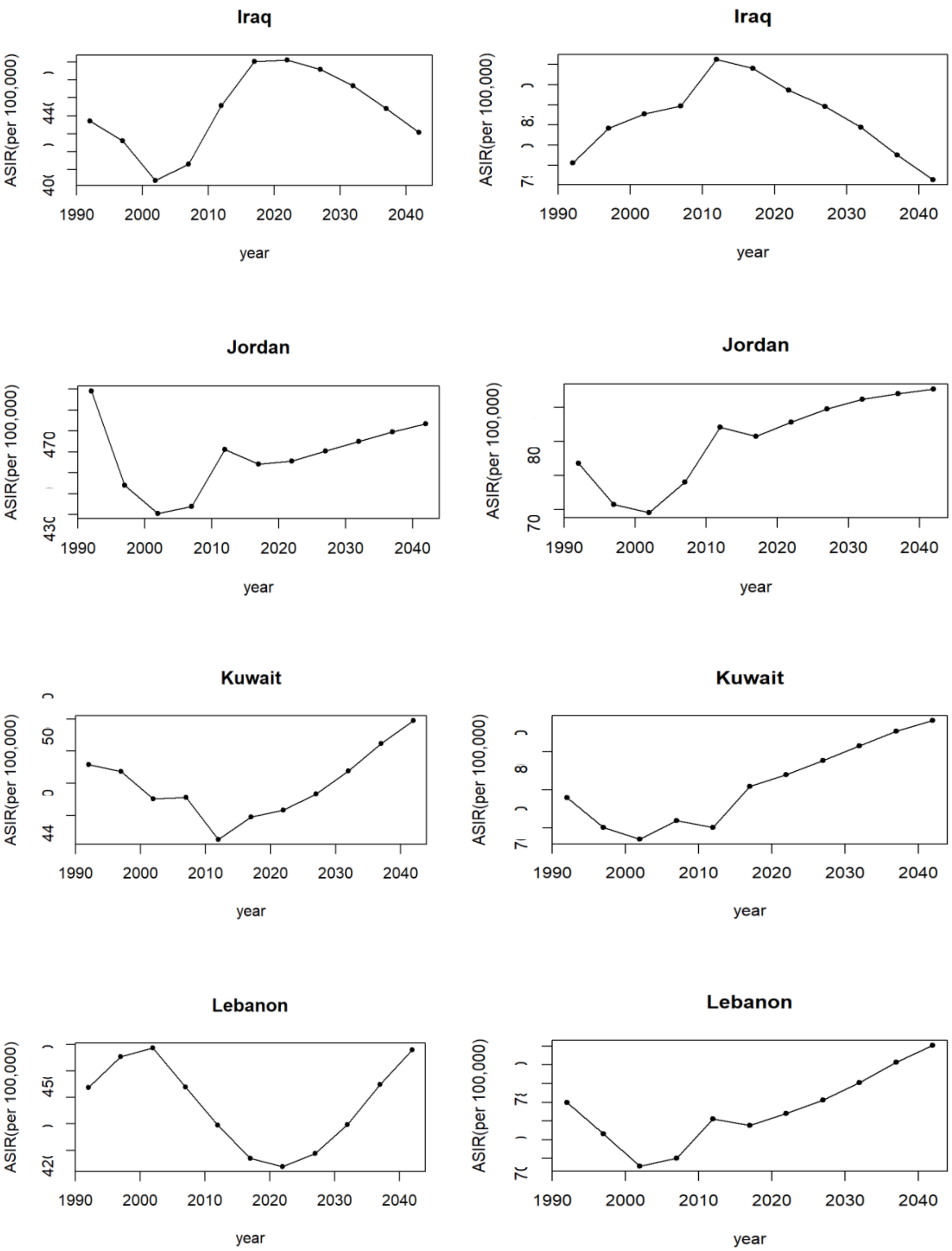
Iraq
ASIR(per 100,000)
year
Iraq
ASIR(per 100,000)
year
Jordan
ASIR(per 100,000)
470
430
year
Jordan
ASIR(per 100,000)
80
70
year
Kuwait
ASIR(per 100,000)
50
44
year
Kuwait
ASIR(per 100,000)
year
Lebanon
ASIR(per 100,000)
year
Lebanon
ASIR(per 100,000)
year
1990 2000 2010 2020 2030 2040

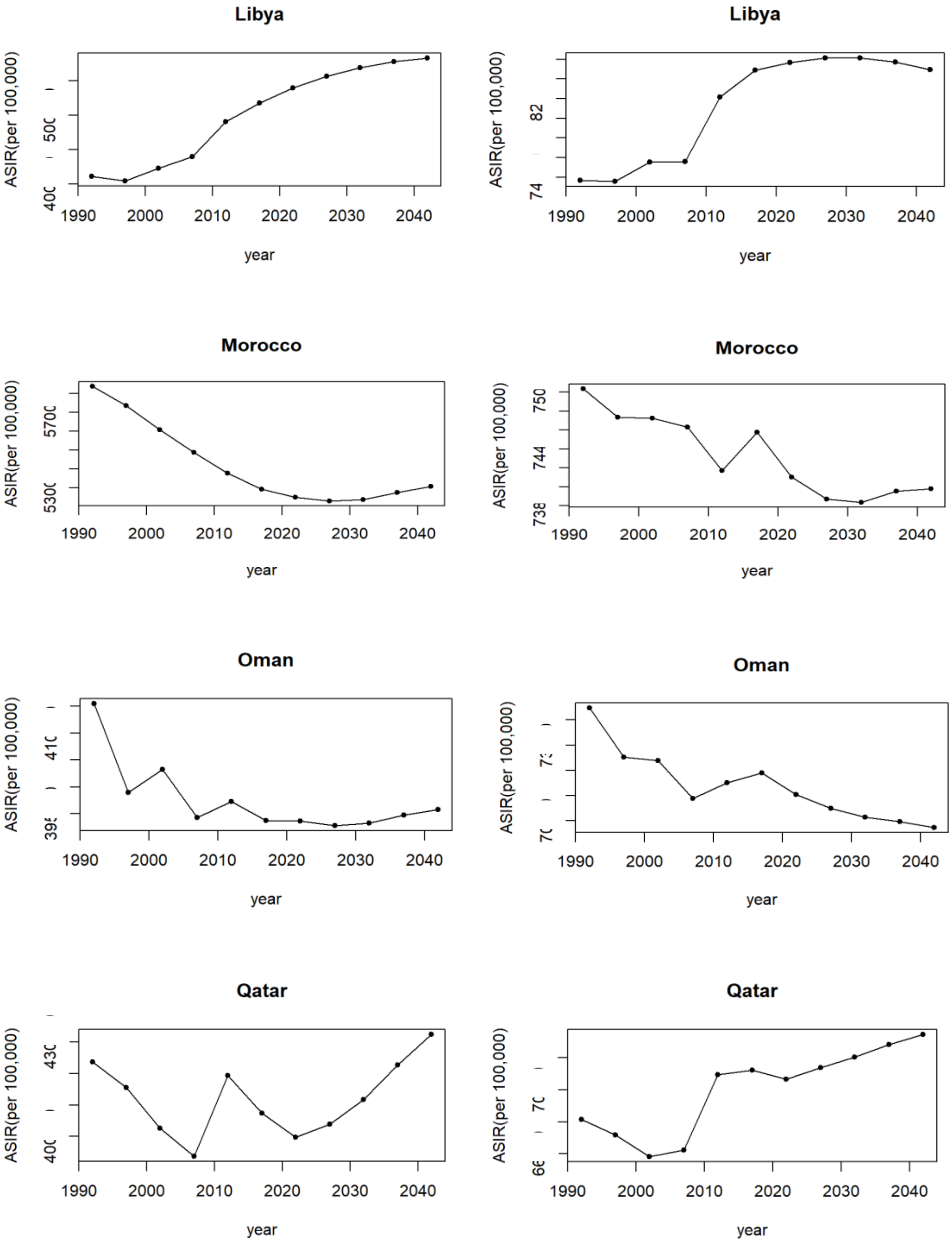

Libya
ASIR(per 100,000)
year
1990 2000 2010 2020 2030 2040
Libya
ASIR(per 100,000)
74
82
year
1990 2000 2010 2020 2030 2040
Morocco
ASIR(per 100,000)
year
1990 2000 2010 2020 2030 2040
Morocco
ASIR(per 100,000)
738
744
750
year
1990 2000 2010 2020 2030 2040
Oman
ASIR(per 100,000)
year
1990 2000 2010 2020 2030 2040
Oman
ASIR(per 100,000)
year
1990 2000 2010 2020 2030 2040
Qatar
ASIR(per 100,000)
year
1990 2000 2010 2020 2030 2040
Qatar
ASIR(per 100,000)
year
1990 2000 2010 2020 2030 2040

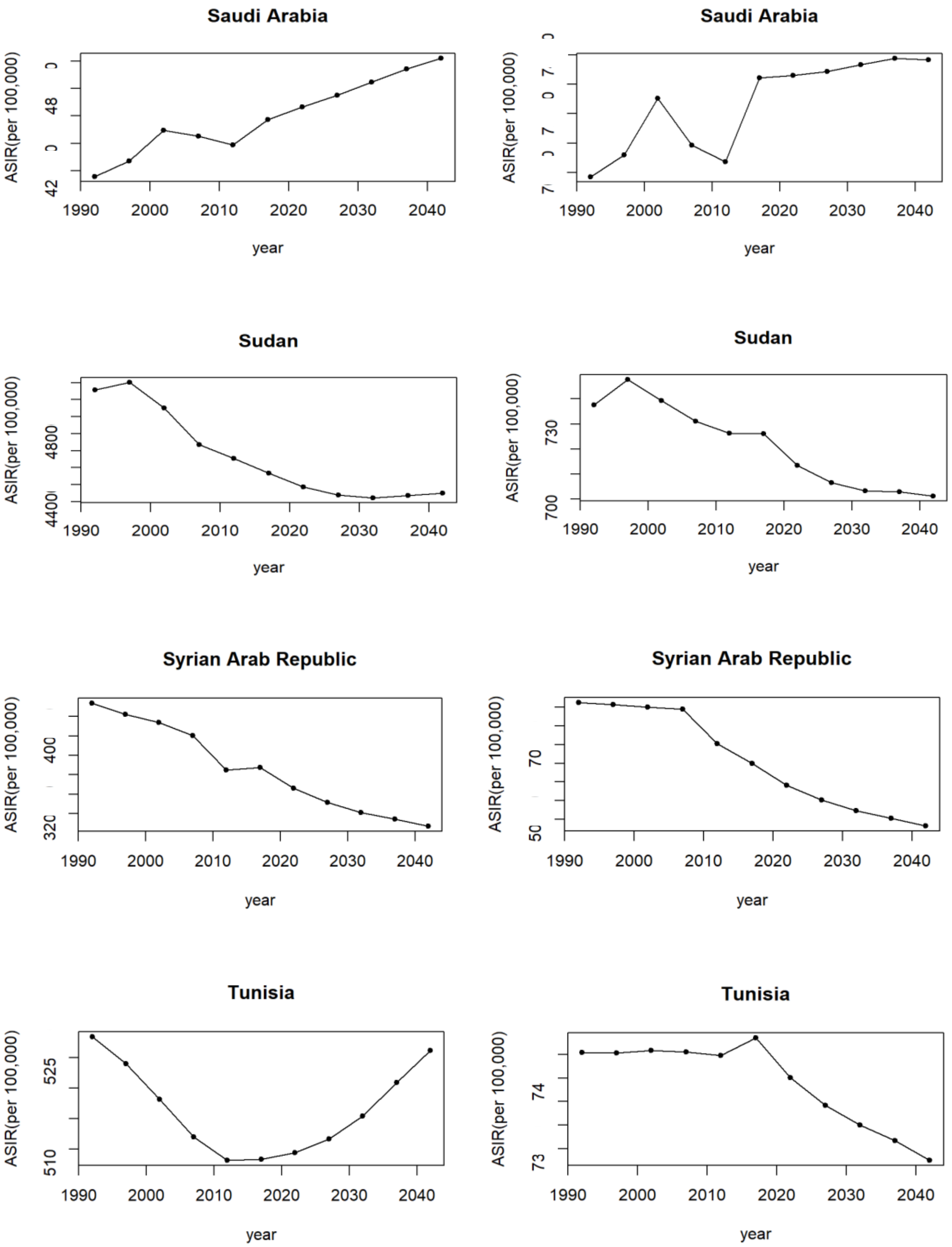
Saudi Arabia
ASIR(per 100,000)
42
48
1990
2000
2010
2020
2030
2040
year
Saudi Arabia
ASIR(per 100,000)
1990
2000
2010
2020
2030
2040
year
Sudan
ASIR(per 100,000)
4800
1990
2000
2010
2020
2030
2040
year
Sudan
ASIR(per 100,000)
700
730
1990
2000
2010
2020
2030
2040
year
Syrian Arab Republic
ASIR(per 100,000)
320
400
1990
2000
2010
2020
2030
2040
year
Syrian Arab Republic
ASIR(per 100,000)
50
70
1990
2000
2010
2020
2030
2040
year
Tunisia
ASIR(per 100,000)
510
525
1990
2000
2010
2020
2030
2040
year
Tunisia
ASIR(per 100,000)
73
74
1990
2000
2010
2020
2030
2040
year

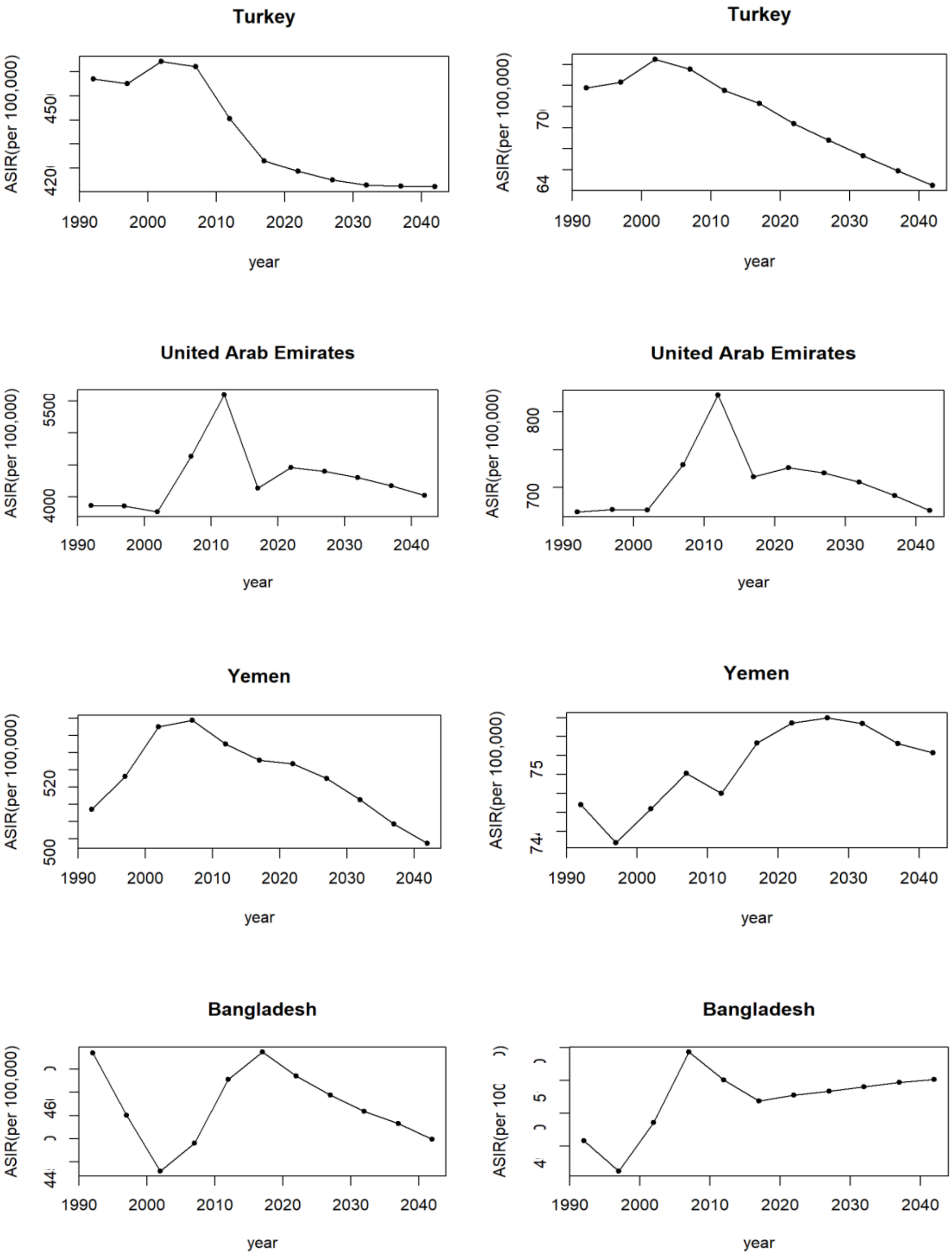
Turkey
ASIR(per 100,000)
420
450
1990
2000
2010
2020
2030
2040
year
Turkey
ASIR(per 100,000)
64
70
1990
2000
2010
2020
2030
2040
year
United Arab Emirates
ASIR(per 100,000)
4000
5500
1990
2000
2010
2020
2030
2040
year
United Arab Emirates
ASIR(per 100,000)
700
800
1990
2000
2010
2020
2030
2040
year
Yemen
ASIR(per 100,000)
500
520
1990
2000
2010
2020
2030
2040
year
Yemen
ASIR(per 100,000)
75
1990
2000
2010
2020
2030
2040
year
Bangladesh
ASIR(per 100,000)
1990
2000
2010
2020
2030
2040
year
Bangladesh
1990
2000
2010
2020
2030
2040
year

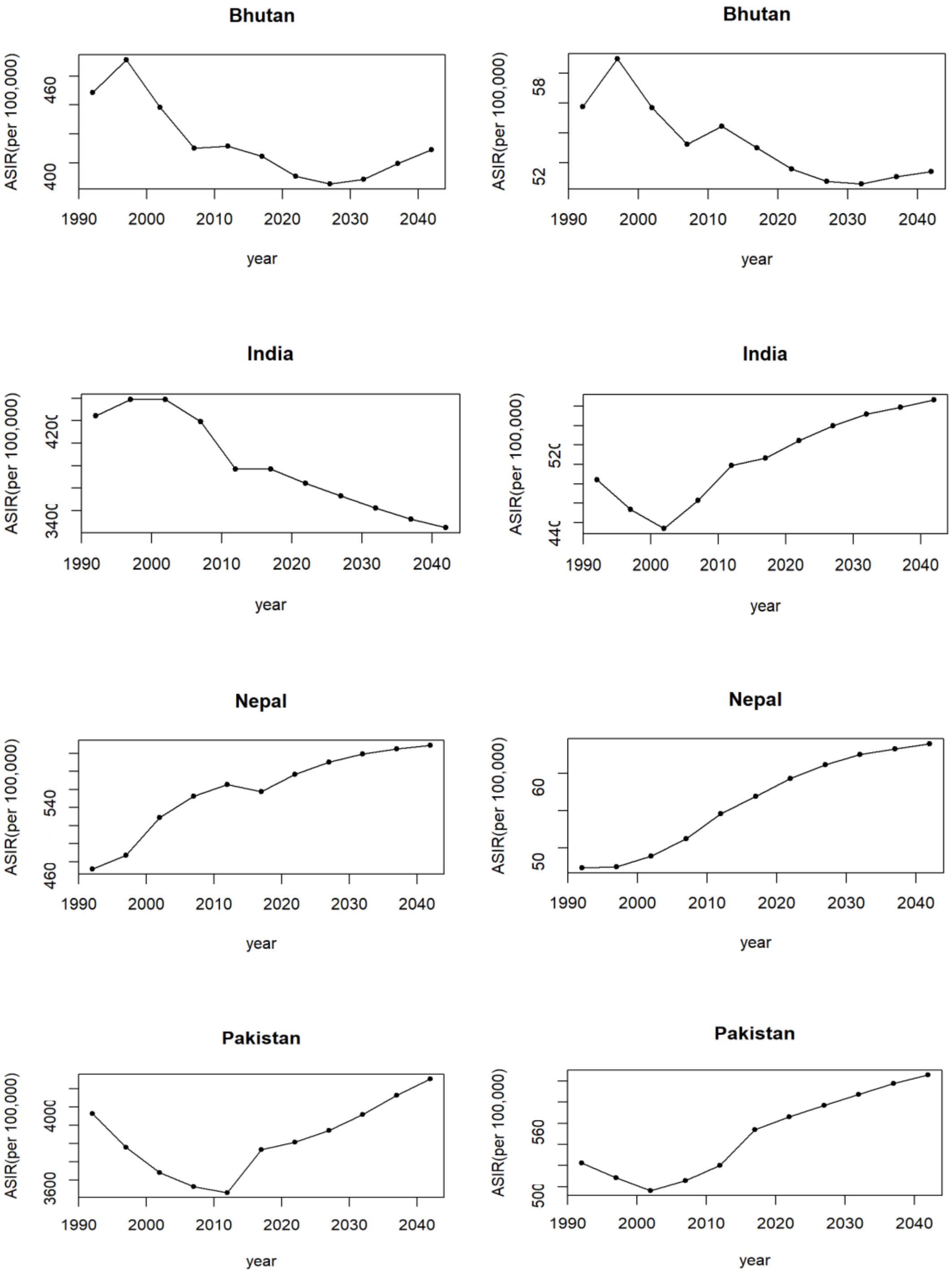
Bhutan
ASIR(per 100,000)
400
460
1990
2000
2010
2020
2030
2040
year
Bhutan
ASIR(per 100,000)
52
58
1990
2000
2010
2020
2030
2040
year
India
ASIR(per 100,000)
1990
2000
2010
2020
2030
2040
year
India
ASIR(per 100,000)
1990
2000
2010
2020
2030
2040
year
Nepal
ASIR(per 100,000)
460
540
1990
2000
2010
2020
2030
2040
year
Nepal
ASIR(per 100,000)
50
60
1990
2000
2010
2020
2030
2040
year
Pakistan
ASIR(per 100,000)
3600
1990
2000
2010
2020
2030
2040
year
Pakistan
ASIR(per 100,000)
560
1990
2000
2010
2020
2030
2040
year

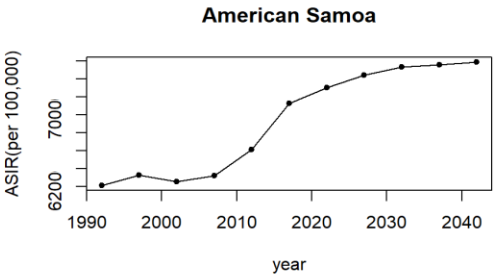
American Samoa
ASIR(per 100,000)
6200
7000
1990
2000
2010
2020
2030
2040
year

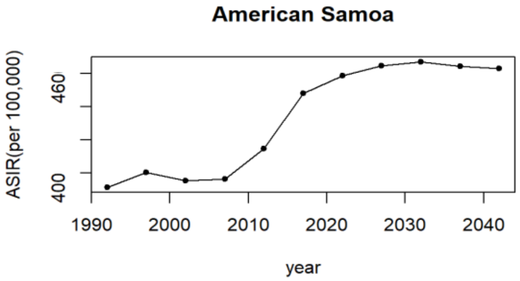
American Samoa
ASIR(per 100,000)
400
460
1990
2000
2010
2020
2030
2040
year

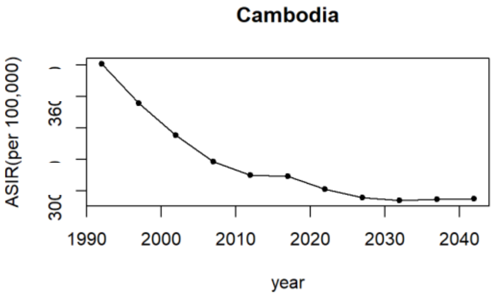
Cambodia
ASIR(per 100,000)
1990
2000
2010
2020
2030
2040
year

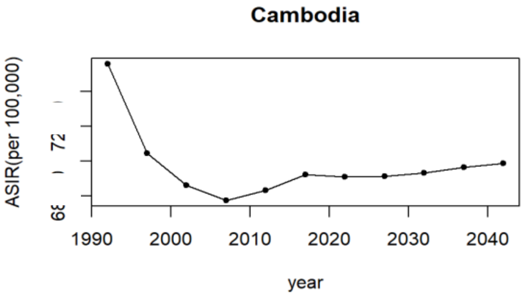
Cambodia
ASIR(per 100,000)
72
1990
2000
2010
2020
2030
2040
year

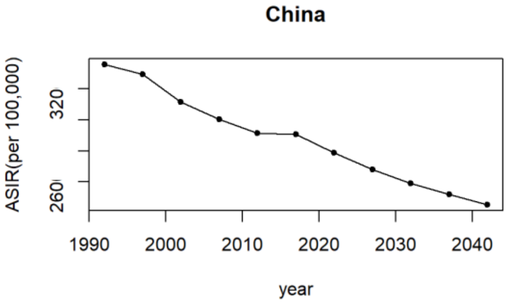
China
ASIR(per 100,000)
320
1990
2000
2010
2020
2030
2040
year

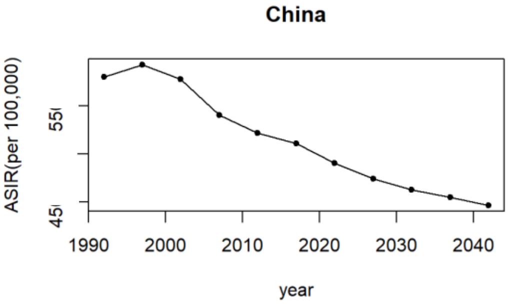
China
ASIR(per 100,000)
1990
2000
2010
2020
2030
2040
year

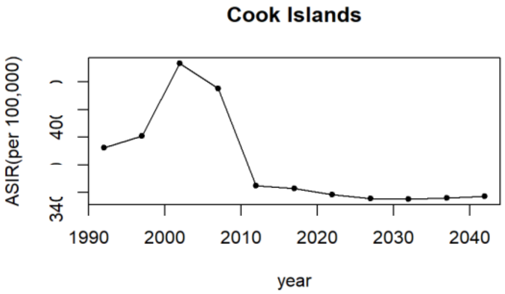
Cook Islands
ASIR(per 100,000)
1990
2000
2010
2020
2030
2040
year

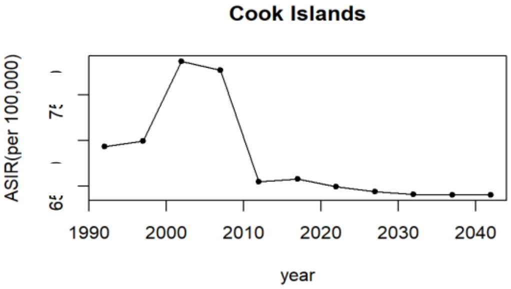
Cook Islands
ASIR(per 100,000)
1990
2000
2010
2020
2030
2040
year

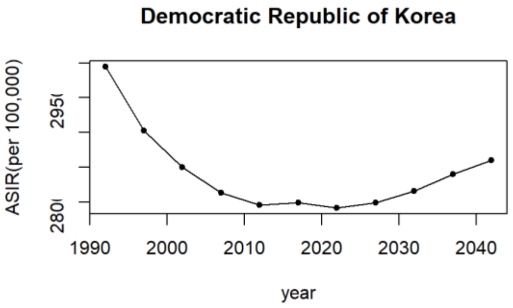
Democratic Republic of Korea
ASIR(per 100,000)
1990 2000 2010 2020 2030 2040
year

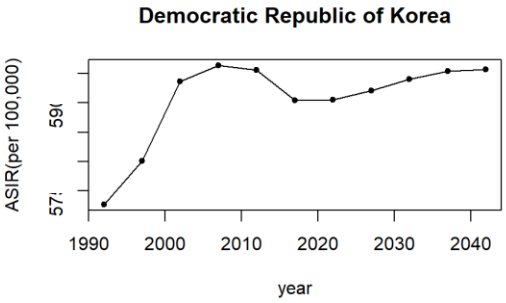
Democratic Republic of Korea
ASIR(per 100,000)
1990 2000 2010 2020 2030 2040
year

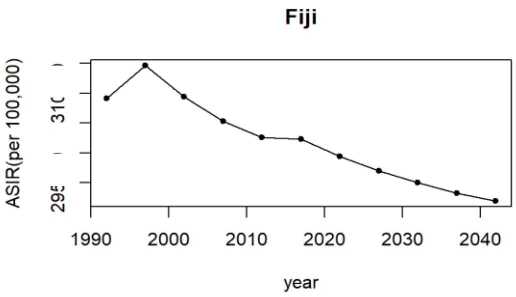
Fiji
ASIR(per 100,000)
1990 2000 2010 2020 2030 2040
year

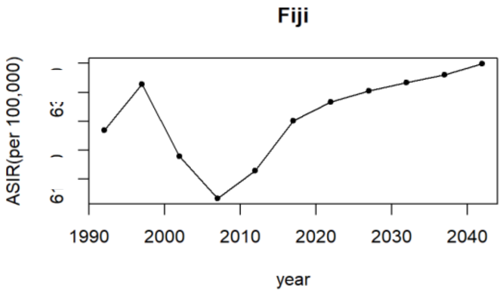
Fiji
ASIR(per 100,000)
1990 2000 2010 2020 2030 2040
year

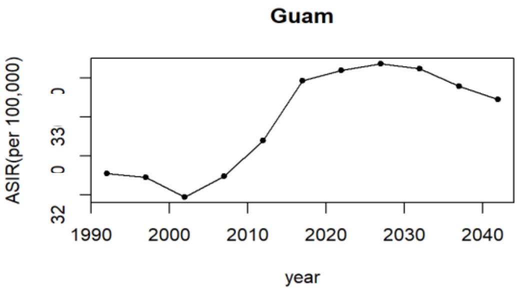
Guam
ASIR(per 100,000)
33
32
1990 2000 2010 2020 2030 2040
year

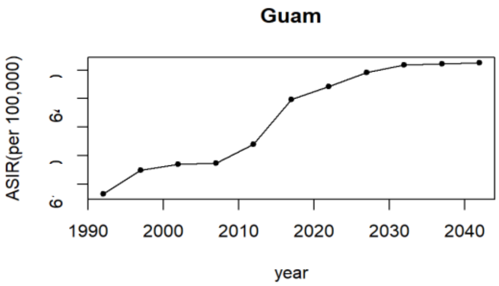
Guam
ASIR(per 100,000)
1990 2000 2010 2020 2030 2040
year

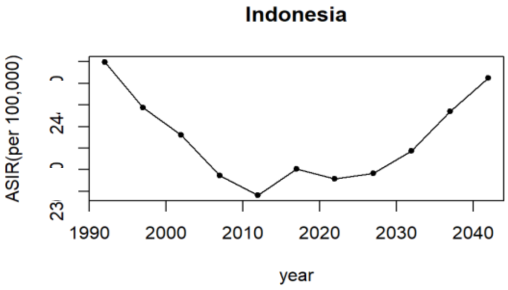
Indonesia
ASIR(per 100,000)
23
1990 2000 2010 2020 2030 2040
year

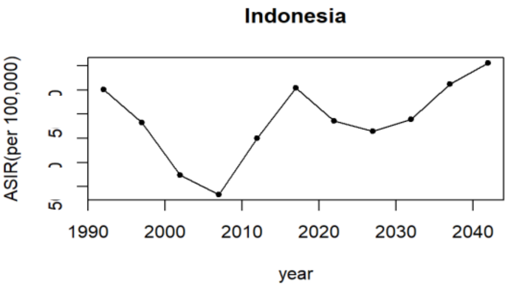
Indonesia
ASIR(per 100,000)
5
1990 2000 2010 2020 2030 2040
year

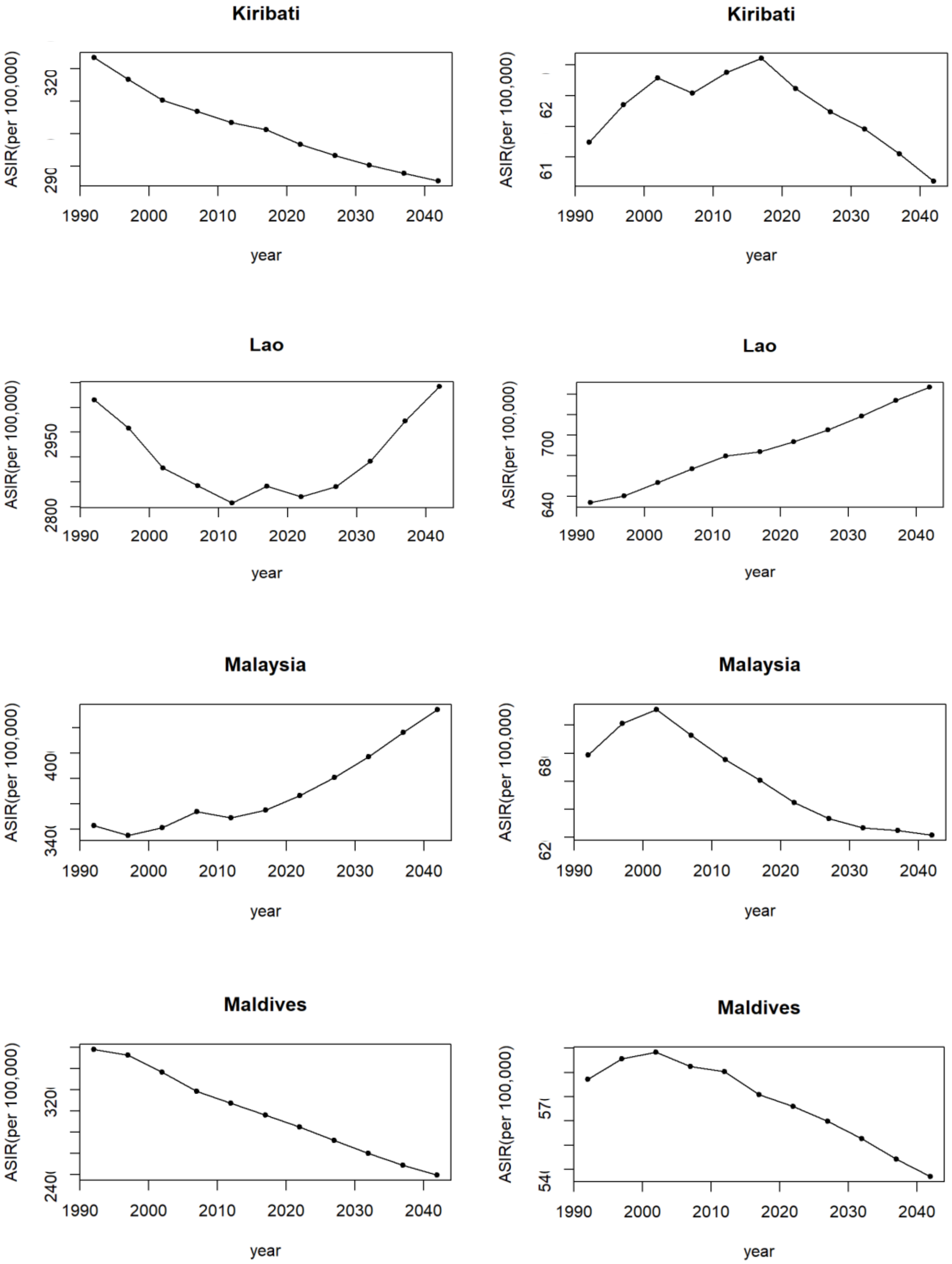
Kiribati
ASIR(per 100,000)
320
290
year
1990
2000
2010
2020
2030
2040
Kiribati
62
61
Lao
2950
2800
Lao
700
640
Malaysia
400
340
Malaysia
68
62
Maldives
320
240
Maldives
57
54

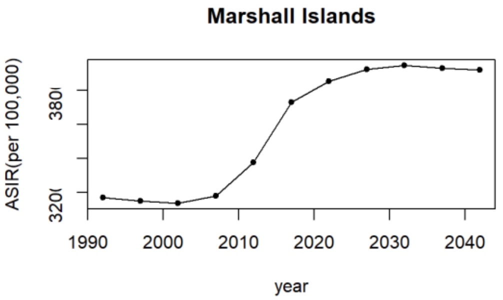
Marshall Islands
ASIR(per 100,000)
380
320
1990
2000
2010
2020
2030
2040
year

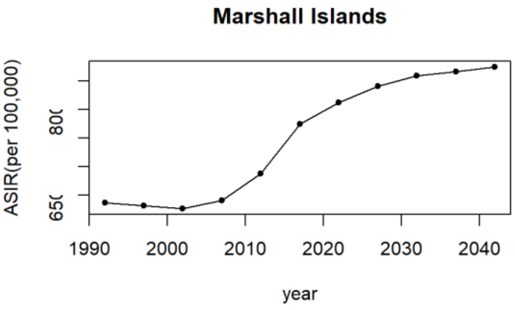
Marshall Islands
ASIR(per 100,000)
80
65
1990
2000
2010
2020
2030
2040
year

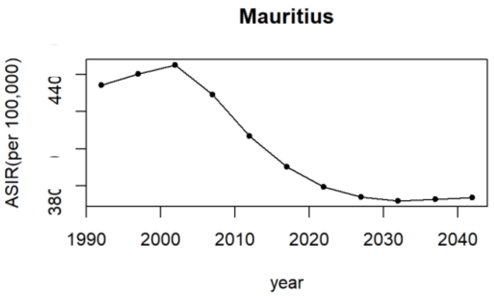
Mauritius
ASIR(per 100,000)
440
380
1990
2000
2010
2020
2030
2040
year

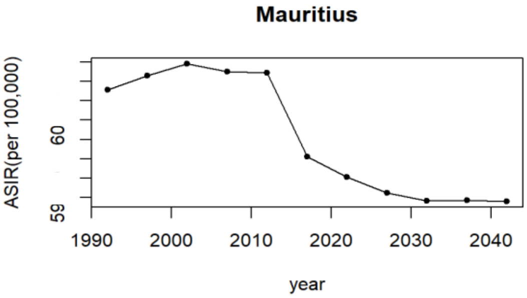
Mauritius
ASIR(per 100,000)
60
59
1990
2000
2010
2020
2030
2040
year

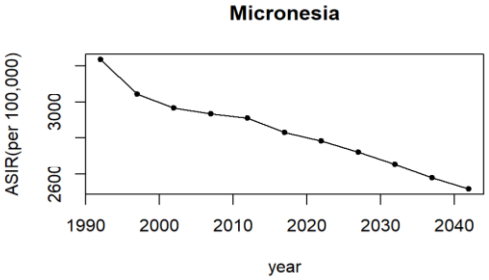
Micronesia
ASIR(per 100,000)
3000
2600
1990
2000
2010
2020
2030
2040
year

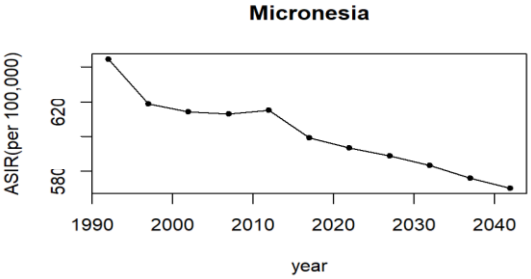
Micronesia
ASIR(per 100,000)
620
580
1990
2000
2010
2020
2030
2040
year

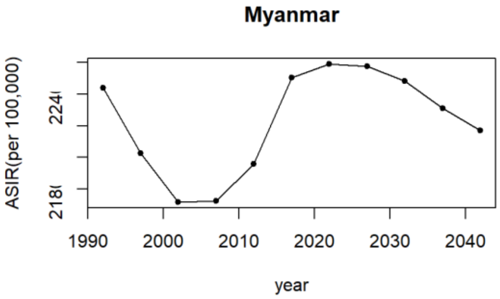
Myanmar
ASIR(per 100,000)
224
218
1990
2000
2010
2020
2030
2040
year

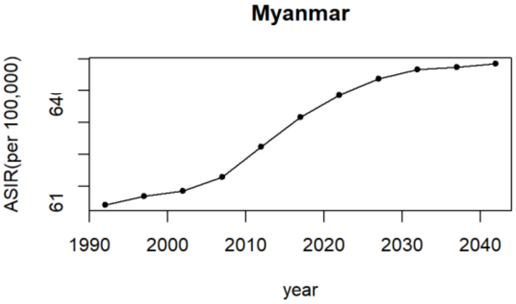
Myanmar
ASIR(per 100,000)
64
61
1990
2000
2010
2020
2030
2040
year

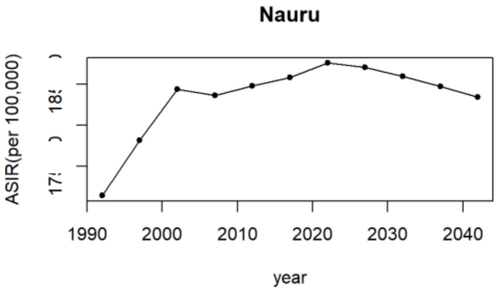
Nauru
ASIR(per 100,000)
1990 2000 2010 2020 2030 2040
year

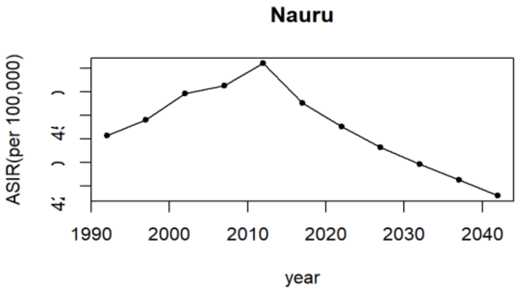
Nauru
ASIR(per 100,000)
1990 2000 2010 2020 2030 2040
year

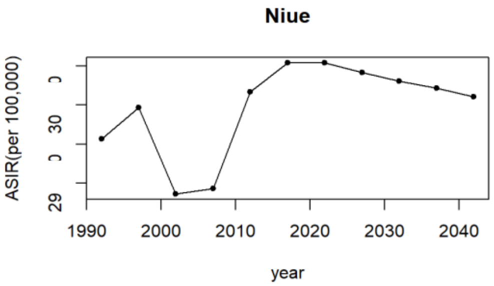
Niue
ASIR(per 100,000)
30
29
1990 2000 2010 2020 2030 2040
year

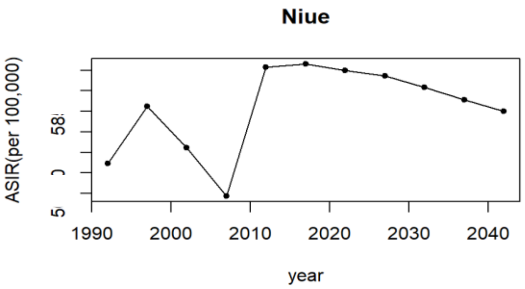
Niue
ASIR(per 100,000)
1990 2000 2010 2020 2030 2040
year

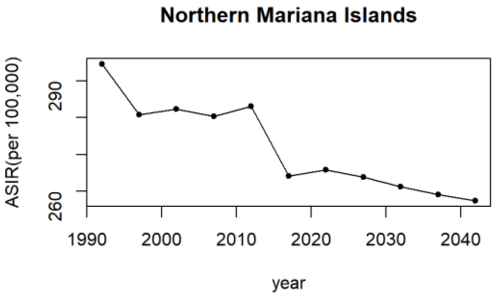
Northern Mariana Islands
ASIR(per 100,000)
290
260
1990 2000 2010 2020 2030 2040
year

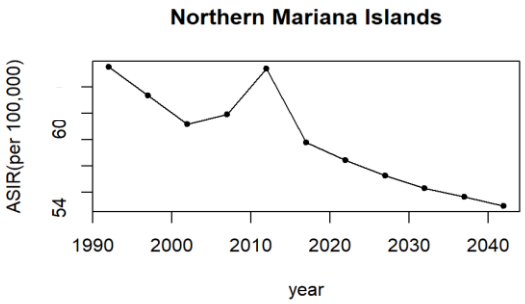
Northern Mariana Islands
ASIR(per 100,000)
60
54
1990 2000 2010 2020 2030 2040
year

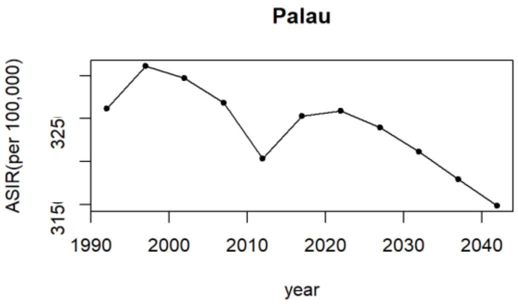
Palau
ASIR(per 100,000)
1990 2000 2010 2020 2030 2040
year

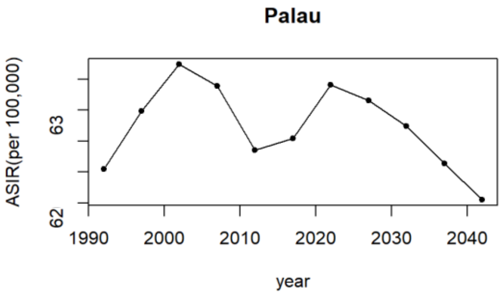
Palau
ASIR(per 100,000)
63
62
1990 2000 2010 2020 2030 2040
year

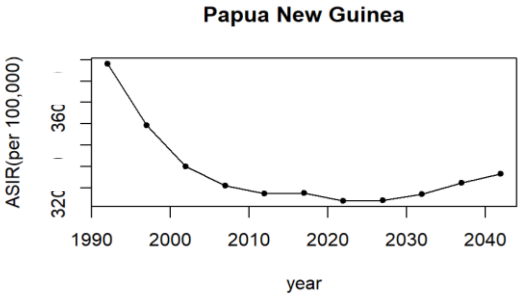
Papua New Guinea
ASIR(per 100,000)
320
360
1990
2000
2010
2020
2030
2040
year

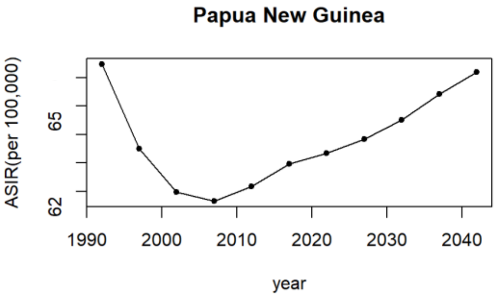
Papua New Guinea
ASIR(per 100,000)
62
65
1990
2000
2010
2020
2030
2040
year

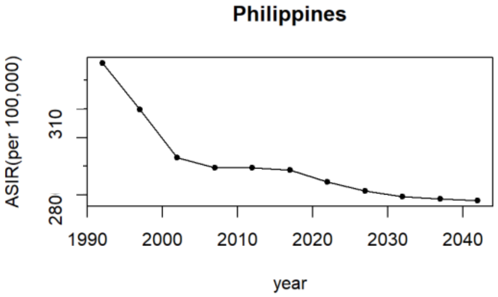
Philippines
ASIR(per 100,000)
280
310
1990
2000
2010
2020
2030
2040
year

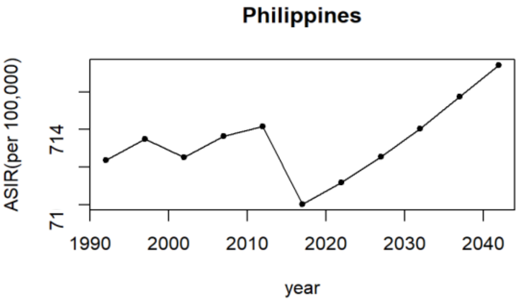
Philippines
ASIR(per 100,000)
71
714
1990
2000
2010
2020
2030
2040
year

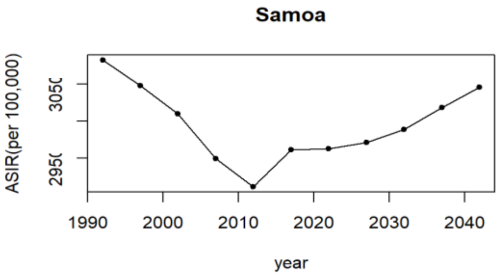
Samoa
ASIR(per 100,000)
295(
305(
1990
2000
2010
2020
2030
2040
year

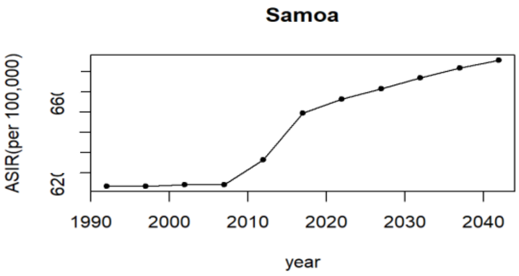
Samoa
ASIR(per 100,000)
62(
66(
1990
2000
2010
2020
2030
2040
year

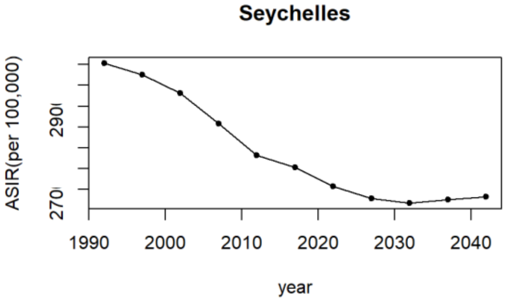
Seychelles
ASIR(per 100,000)
270
290
1990
2000
2010
2020
2030
2040
year

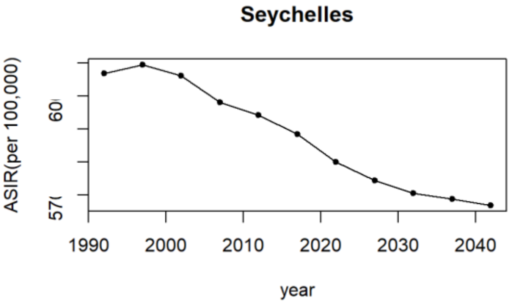
Seychelles
ASIR(per 100,000)
57(
60
1990
2000
2010
2020
2030
2040
year

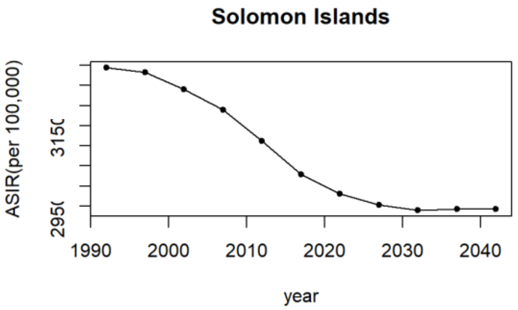
Solomon Islands
ASIR(per 100,000)
1990
2000
2010
2020
2030
2040
year

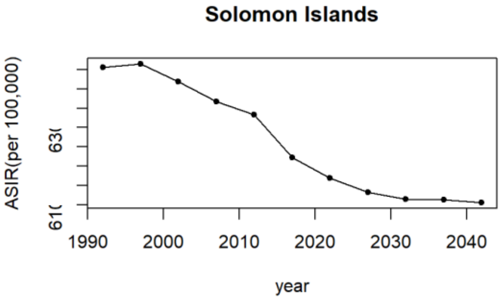
Solomon Islands
ASIR(per 100,000)
1990
2000
2010
2020
2030
2040
year

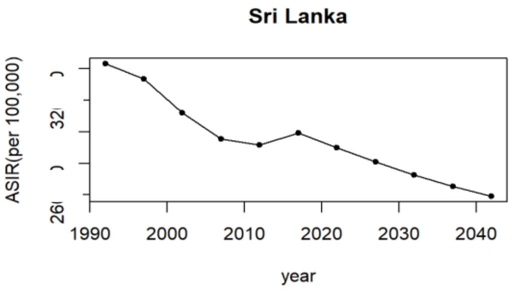
Sri Lanka
ASIR(per 100,000)
1990
2000
2010
2020
2030
2040
year

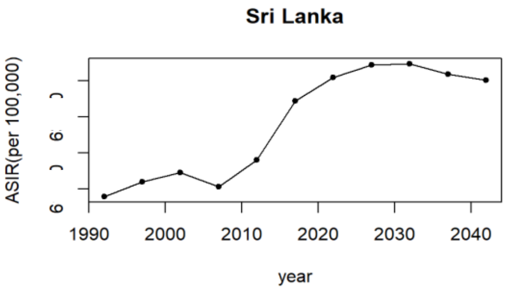
Sri Lanka
ASIR(per 100,000)
1990
2000
2010
2020
2030
2040
year

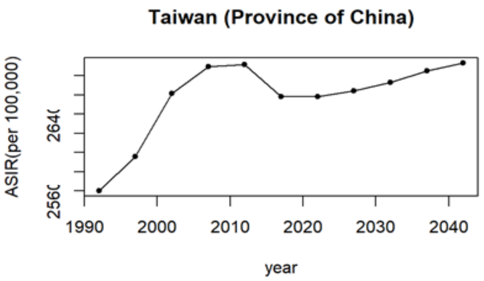
Taiwan (Province of China)
ASIR(per 100,000)
1990
2000
2010
2020
2030
2040
year

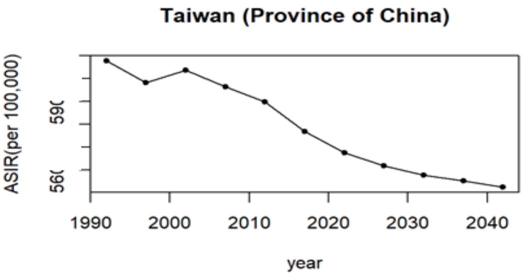
Taiwan (Province of China)
ASIR(per 100,000)
1990
2000
2010
2020
2030
2040
year

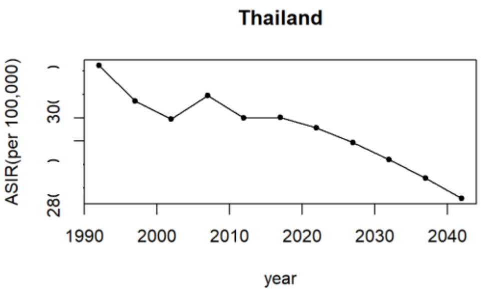
Thailand
ASIR(per 100,000)
1990
2000
2010
2020
2030
2040
year

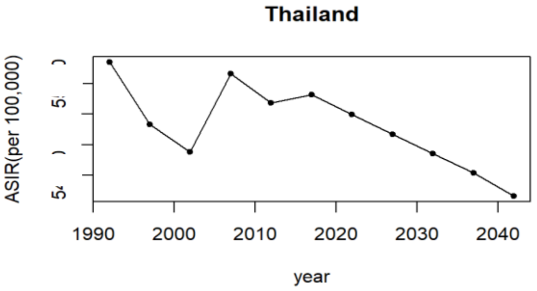
Thailand
ASIR(per 100,000)
1990
2000
2010
2020
2030
2040
year

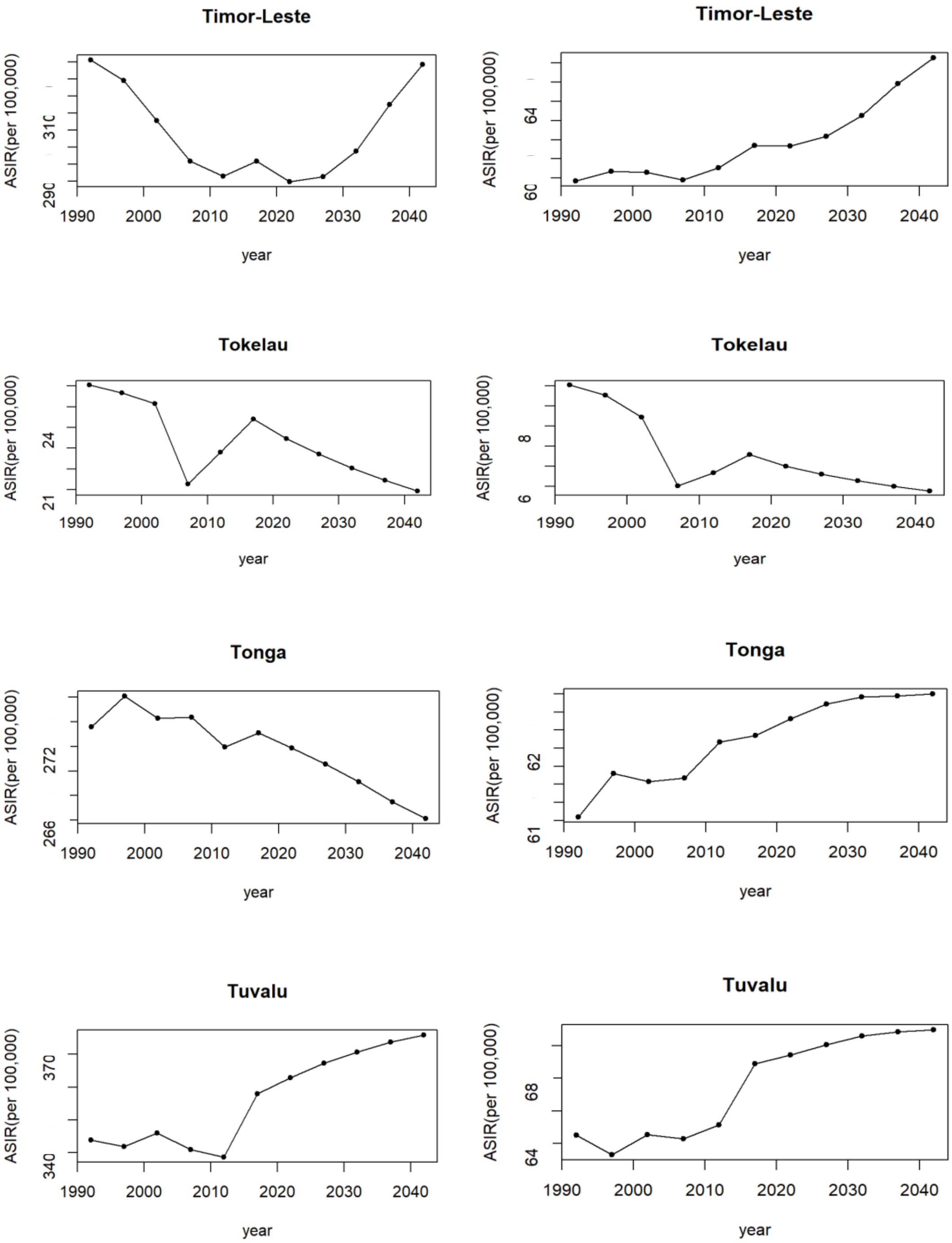
Timor-Leste
ASIR(per 100,000)
290
310
year
1990
2000
2010
2020
2030
2040
Timor-Leste
ASIR(per 100,000)
60
64
year
Tokelau
ASIR(per 100,000)
21
24
year
Tokelau
ASIR(per 100,000)
6
8
year
Tonga
ASIR(per 100,000)
266
272
year
Tonga
ASIR(per 100,000)
61
62
year
Tuvalu
ASIR(per 100,000)
340
370
year
Tuvalu
ASIR(per 100,000)
64
68
year

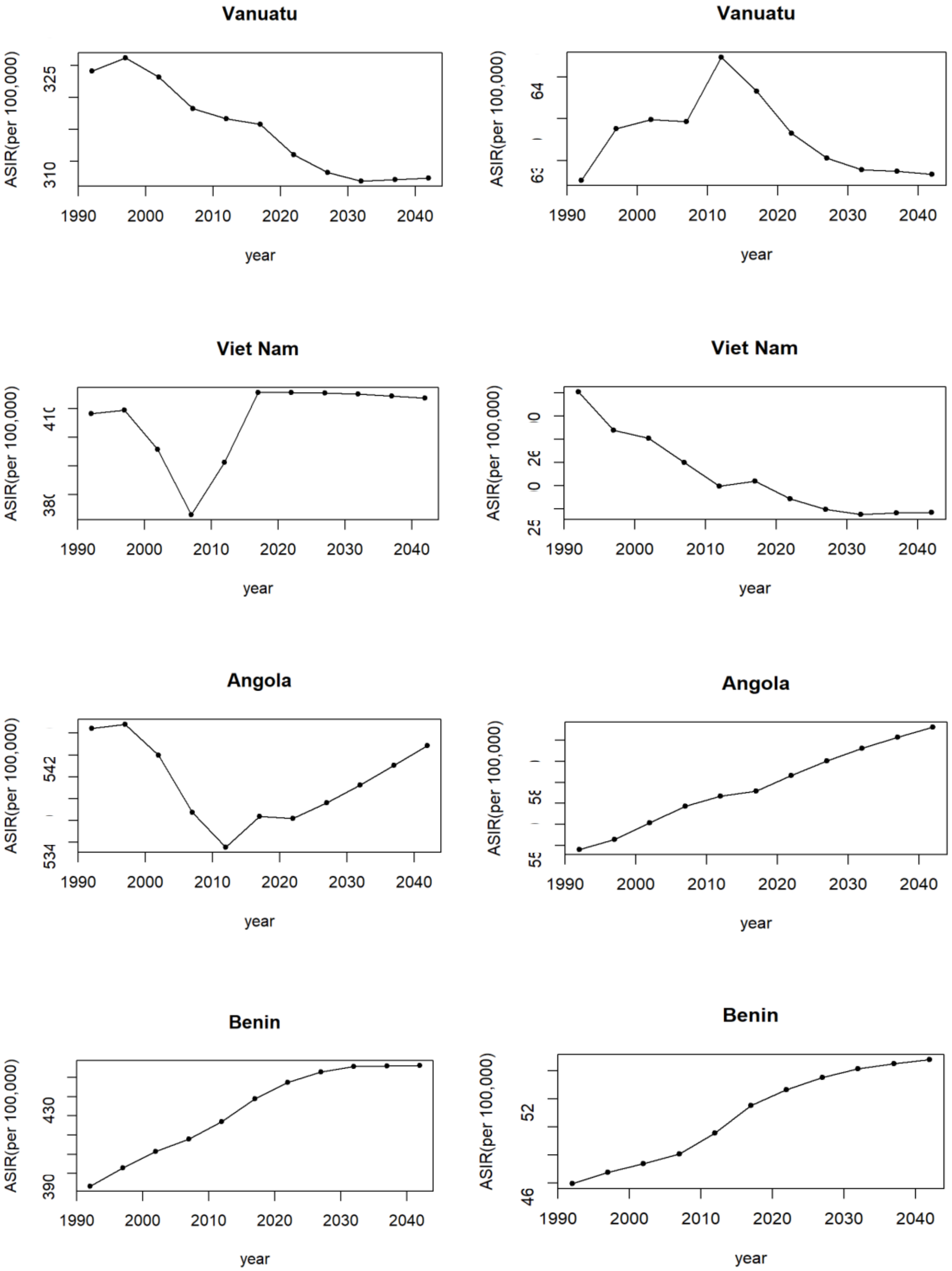
Vanuatu
ASIR(per 100,000)
year
Vanuatu
ASIR(per 100,000)
year
Viet Nam
ASIR(per 100,000)
year
Viet Nam
ASIR(per 100,000)
year
Angola
ASIR(per 100,000)
year
Angola
ASIR(per 100,000)
year
Benin
ASIR(per 100,000)
year
Benin
ASIR(per 100,000)
year

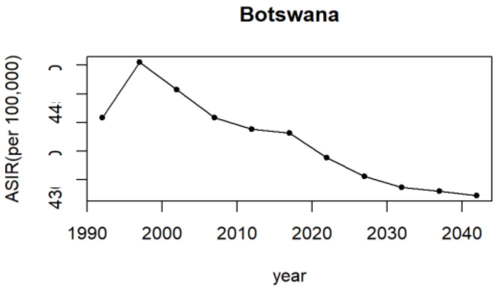
Botswana
ASIR(per 100,000)
1990 2000 2010 2020 2030 2040
year
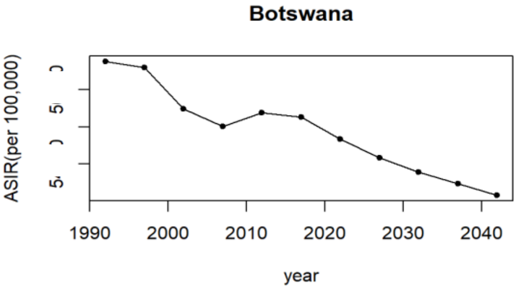
Botswana
ASIR(per 100,000)
1990 2000 2010 2020 2030 2040
year
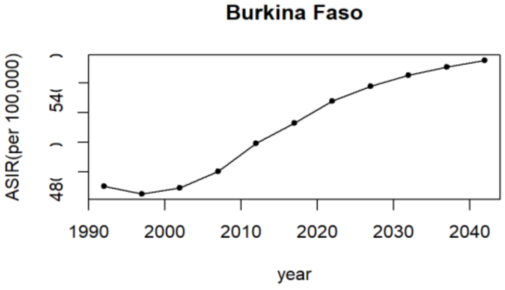
Burkina Faso
ASIR(per 100,000)
1990 2000 2010 2020 2030 2040
year
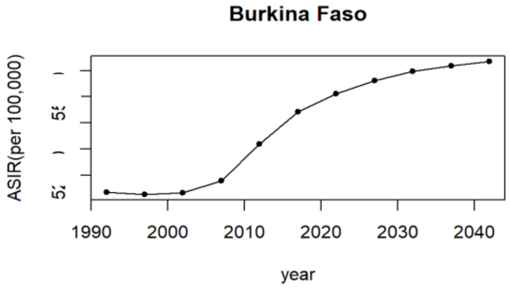
Burkina Faso
ASIR(per 100,000)
1990 2000 2010 2020 2030 2040
year
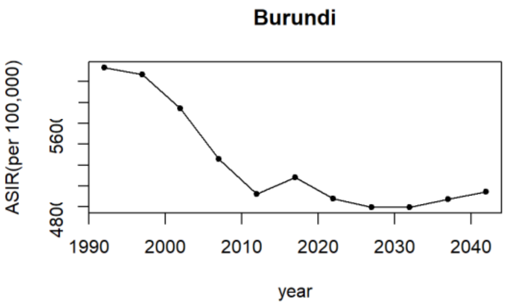
Burundi
ASIR(per 100,000)
1990 2000 2010 2020 2030 2040
year
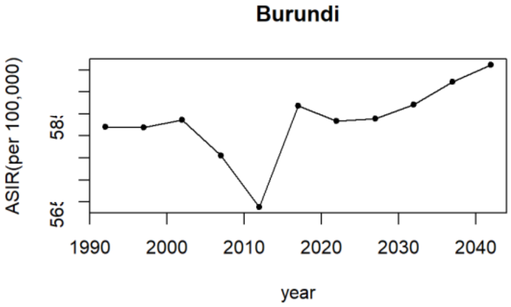
Burundi
ASIR(per 100,000)
1990 2000 2010 2020 2030 2040
year
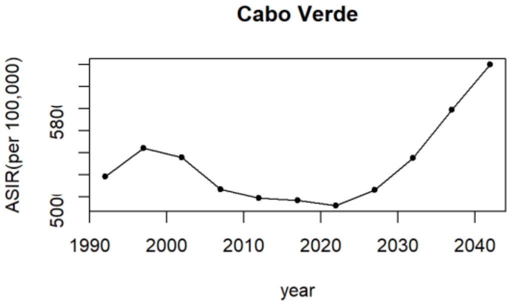
Cabo Verde
ASIR(per 100,000)
1990 2000 2010 2020 2030 2040
year
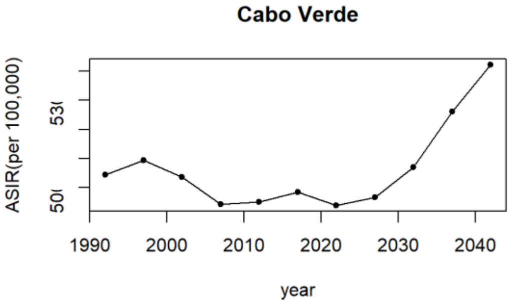
Cabo Verde
ASIR(per 100,000)
1990 2000 2010 2020 2030 2040
year

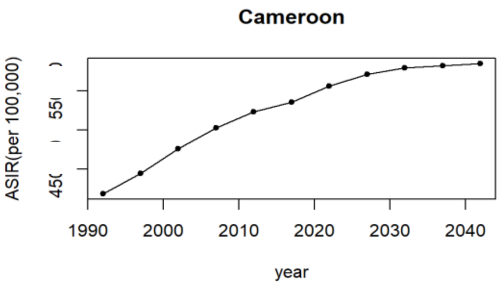
Cameroon
ASIR(per 100,000)
1990 2000 2010 2020 2030 2040
year

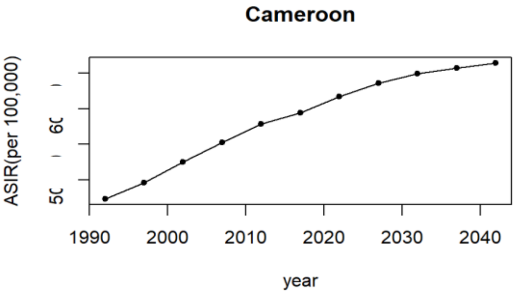
Cameroon
ASIR(per 100,000)
1990 2000 2010 2020 2030 2040
year

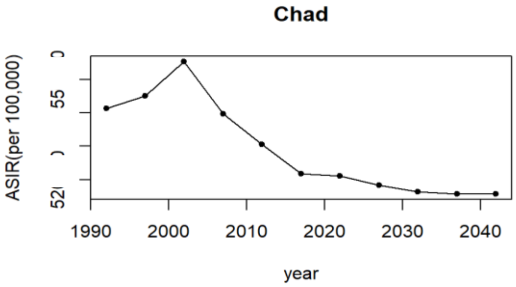
Chad
ASIR(per 100,000)
55
1990 2000 2010 2020 2030 2040
year

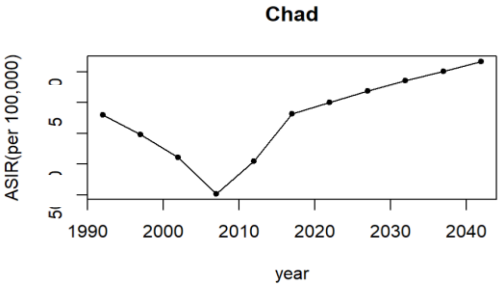
Chad
ASIR(per 100,000)
1990 2000 2010 2020 2030 2040
year

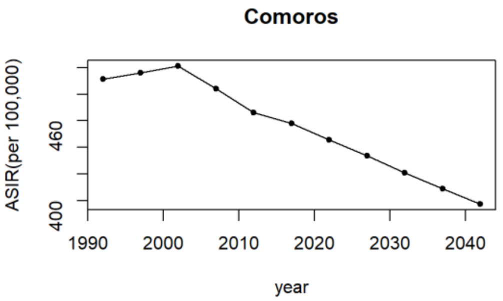
Comoros
ASIR(per 100,000)
460
400
1990 2000 2010 2020 2030 2040
year

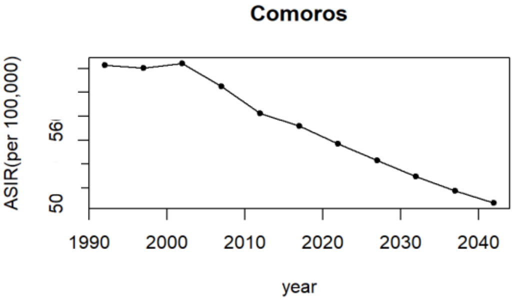
Comoros
ASIR(per 100,000)
50
1990 2000 2010 2020 2030 2040
year

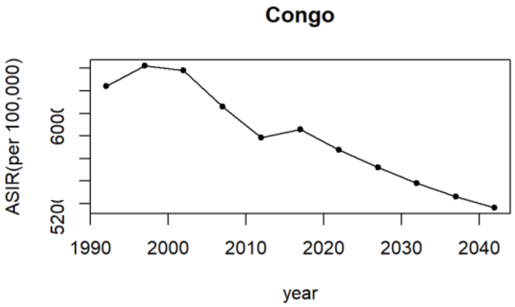
Congo
ASIR(per 100,000)
1990 2000 2010 2020 2030 2040
year

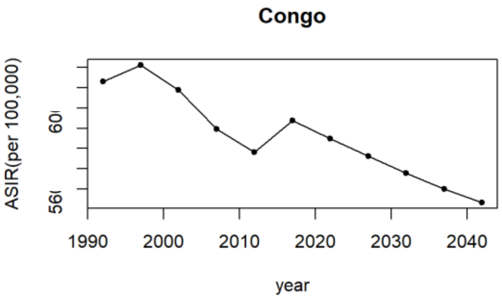
Congo
ASIR(per 100,000)
1990 2000 2010 2020 2030 2040
year

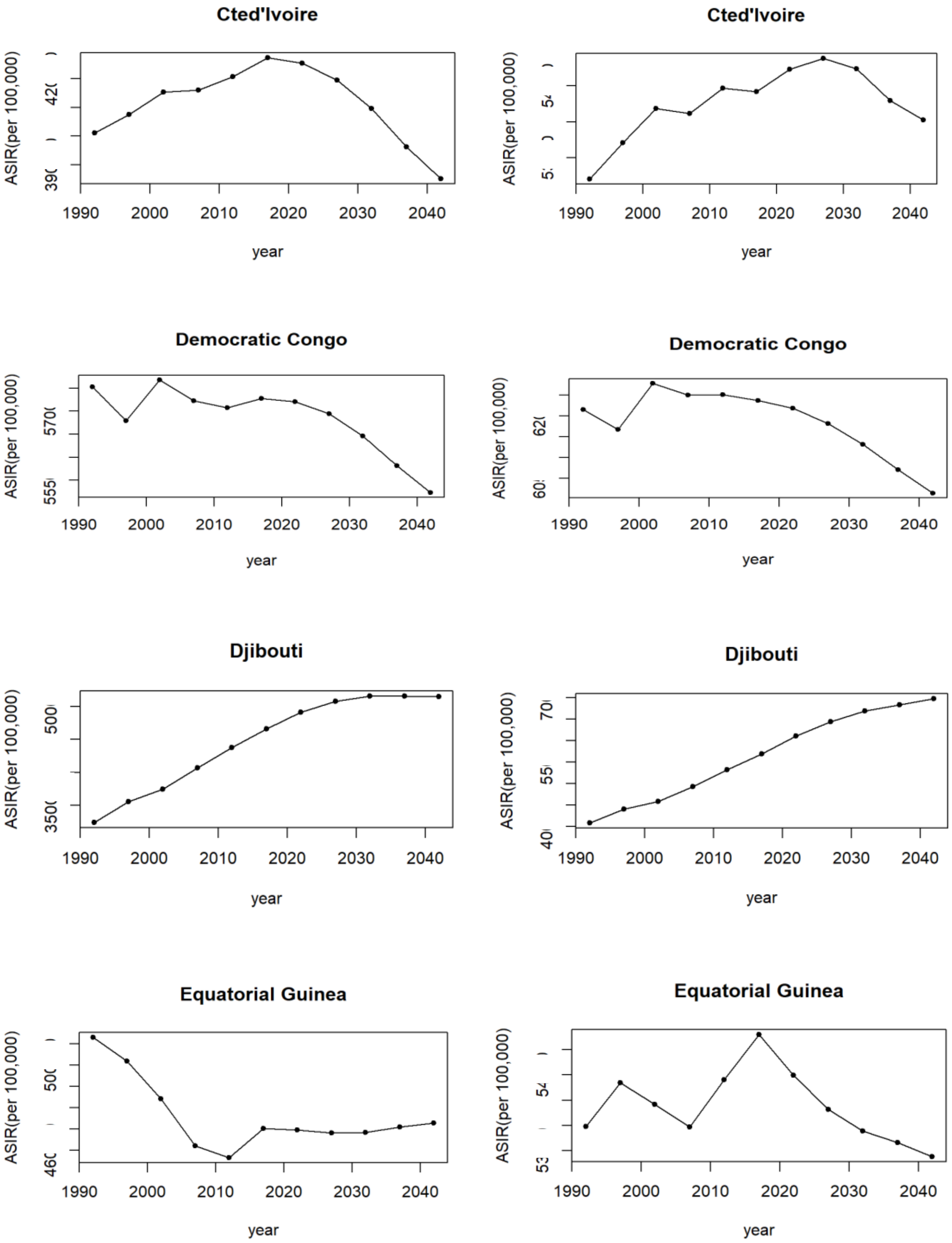
Cted'Ivoire
ASIR(per 100,000)
year
Cted'Ivoire
ASIR(per 100,000)
year
Democratic Congo
ASIR(per 100,000)
year
Democratic Congo
ASIR(per 100,000)
year
Djibouti
ASIR(per 100,000)
year
Djibouti
ASIR(per 100,000)
year
Equatorial Guinea
ASIR(per 100,000)
year
Equatorial Guinea
ASIR(per 100,000)
year

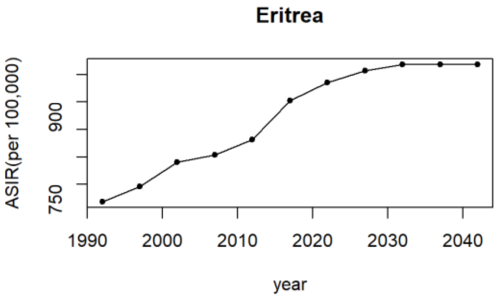
Eritrea
ASIR(per 100,000)
750
900
1990
2000
2010
2020
2030
2040
year

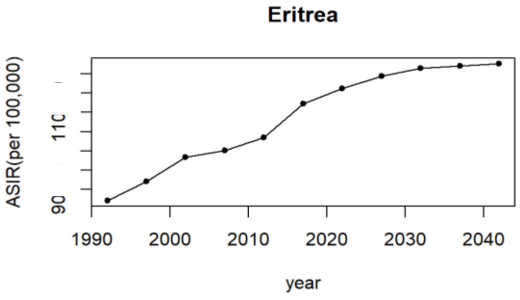
Eritrea
ASIR(per 100,000)
90
110
1990
2000
2010
2020
2030
2040
year

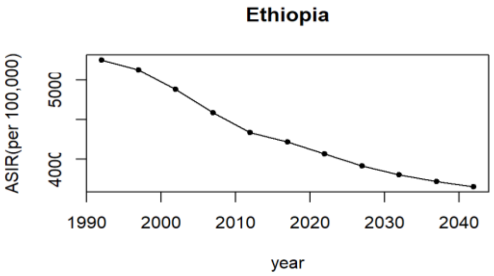
Ethiopia
ASIR(per 100,000)
4000
5000
1990
2000
2010
2020
2030
2040
year

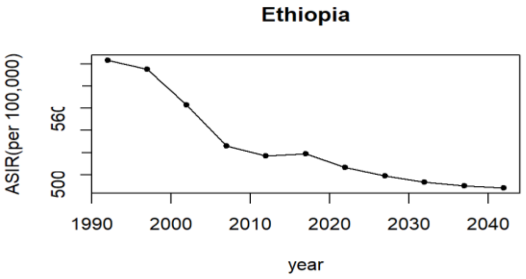
Ethiopia
ASIR(per 100,000)
500
560
1990
2000
2010
2020
2030
2040
year

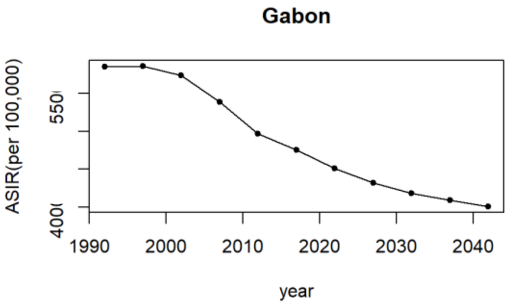
Gabon
ASIR(per 100,000)
400
550
1990
2000
2010
2020
2030
2040
year

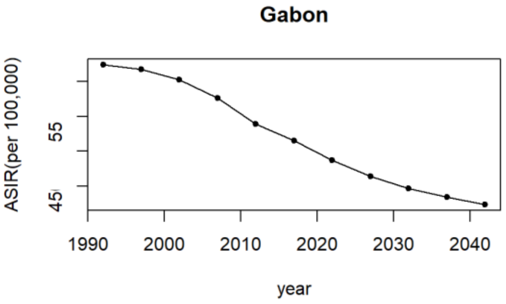
Gabon
ASIR(per 100,000)
45
55
1990
2000
2010
2020
2030
2040
year

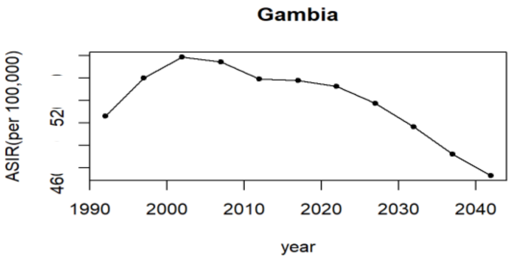
Gambia
ASIR(per 100,000)
46
52
1990
2000
2010
2020
2030
2040
year

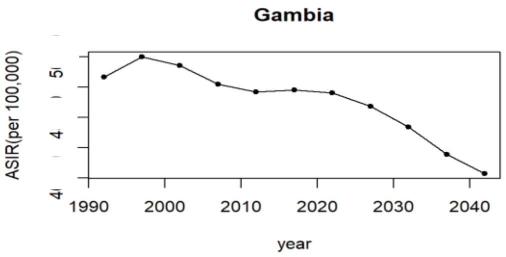
Gambia
ASIR(per 100,000)
4
5
1990
2000
2010
2020
2030
2040
year

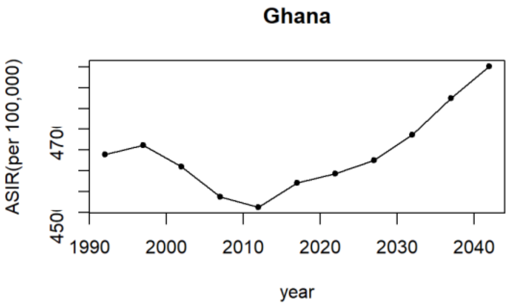
Ghana
ASIR(per 100,000)
450
470
1990
2000
2010
2020
2030
2040
year

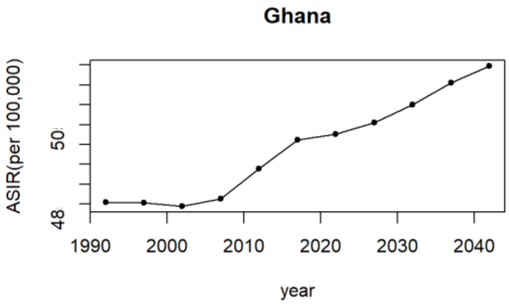
Ghana
ASIR(per 100,000)
48
50
1990
2000
2010
2020
2030
2040
year

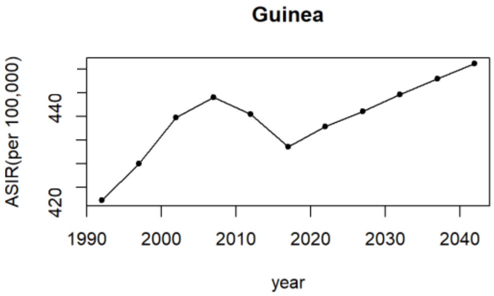
Guinea
ASIR(per 100,000)
420
440
1990
2000
2010
2020
2030
2040
year

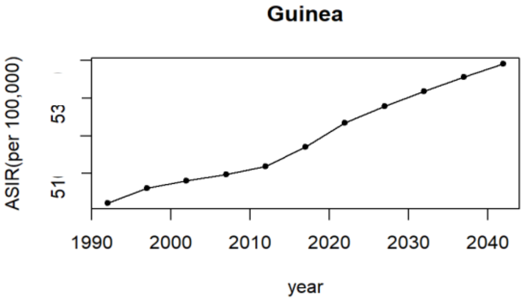
Guinea
ASIR(per 100,000)
51
53
1990
2000
2010
2020
2030
2040
year

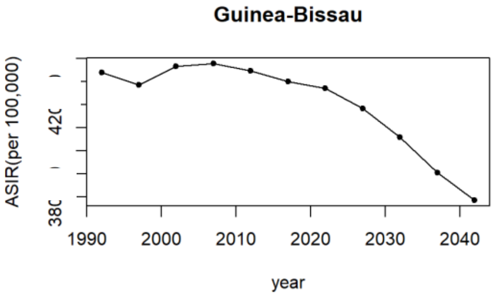
Guinea-Bissau
ASIR(per 100,000)
380
420
1990
2000
2010
2020
2030
2040
year

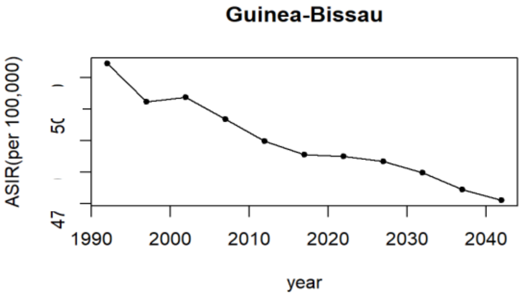
Guinea-Bissau
ASIR(per 100,000)
47
50
1990
2000
2010
2020
2030
2040
year

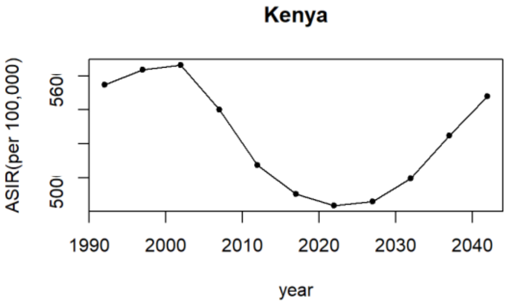
Kenya
ASIR(per 100,000)
500
560
1990
2000
2010
2020
2030
2040
year

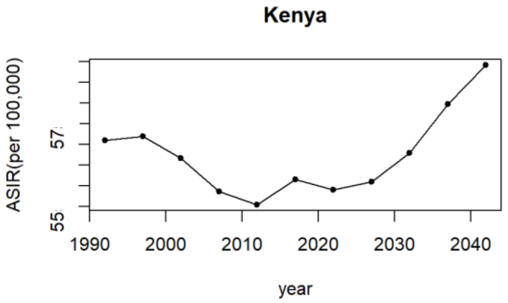
Kenya
ASIR(per 100,000)
55
57
1990
2000
2010
2020
2030
2040
year

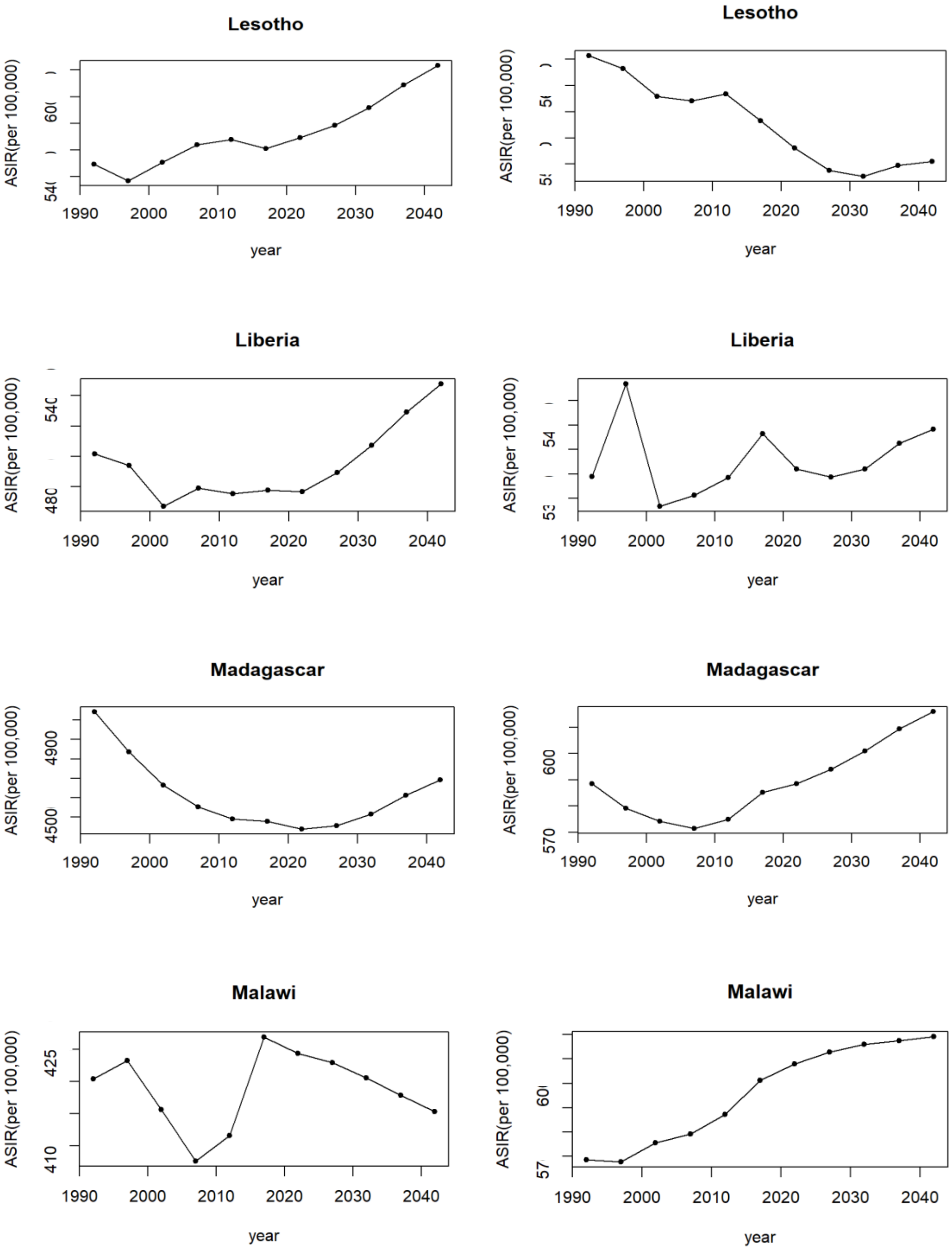
Lesotho
ASIR(per 100,000)
year
1990
2000
2010
2020
2030
2040
Lesotho
ASIR(per 100,000)
year
Liberia
ASIR(per 100,000)
540
480
year
Liberia
ASIR(per 100,000)
year
Madagascar
ASIR(per 100,000)
4900
4500
year
Madagascar
ASIR(per 100,000)
600
570
year
Malawi
ASIR(per 100,000)
425
410
year
Malawi
ASIR(per 100,000)
year

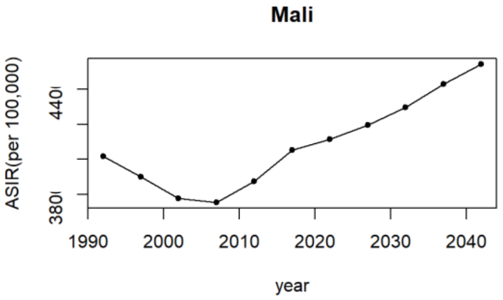
Mali
ASIR(per 100,000)
1990 2000 2010 2020 2030 2040
year

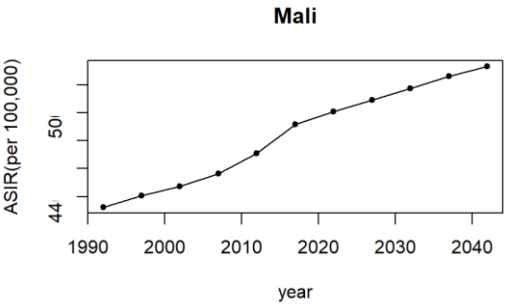
Mali
ASIR(per 100,000)
1990 2000 2010 2020 2030 2040
year

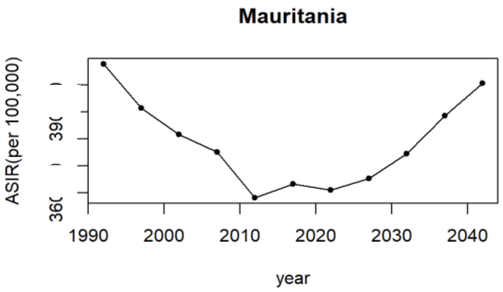
Mauritania
ASIR(per 100,000)
1990 2000 2010 2020 2030 2040
year

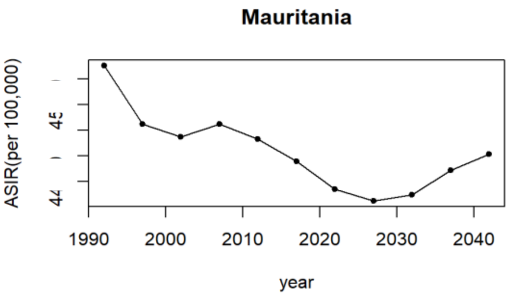
Mauritania
ASIR(per 100,000)
1990 2000 2010 2020 2030 2040
year

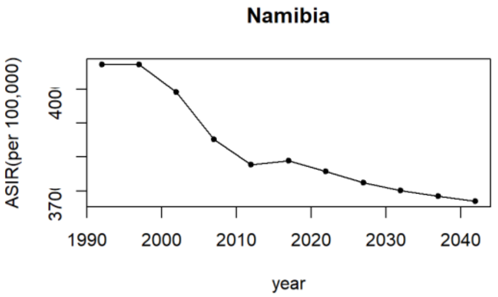
Namibia
ASIR(per 100,000)
1990 2000 2010 2020 2030 2040
year

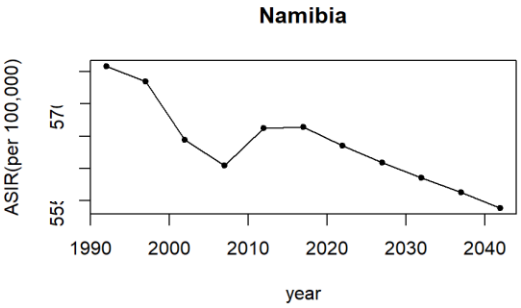
Namibia
ASIR(per 100,000)
1990 2000 2010 2020 2030 2040
year

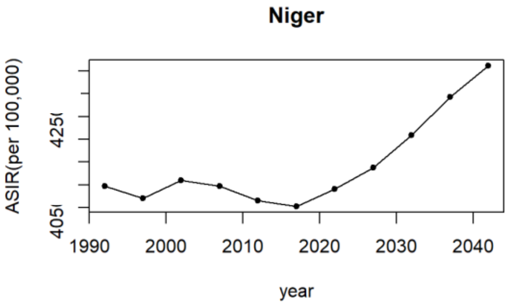
Niger
ASIR(per 100,000)
1990 2000 2010 2020 2030 2040
year

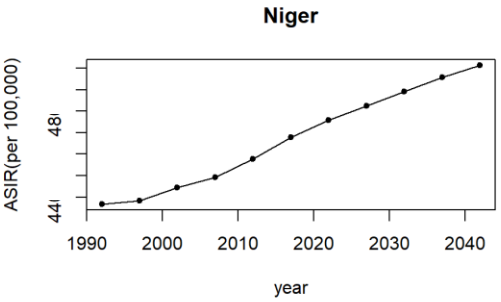
Niger
ASIR(per 100,000)
1990 2000 2010 2020 2030 2040
year

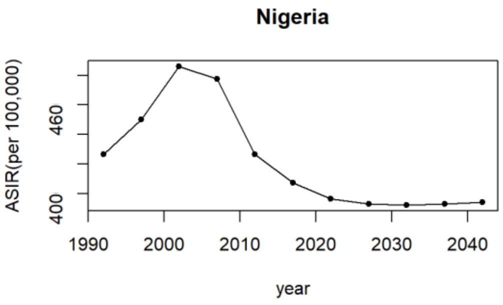
Nigeria
ASIR(per 100,000)
400
460
1990
2000
2010
2020
2030
2040
year

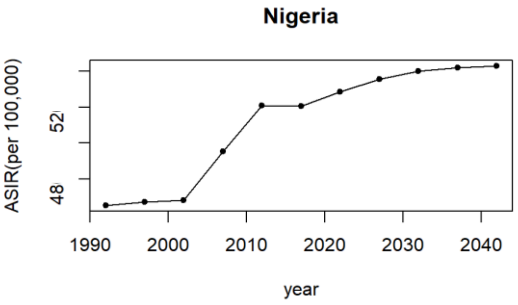
Nigeria
ASIR(per 100,000)
48
52
1990
2000
2010
2020
2030
2040
year

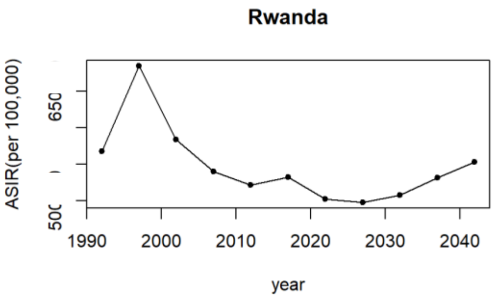
Rwanda
ASIR(per 100,000)
650
1990
2000
2010
2020
2030
2040
year

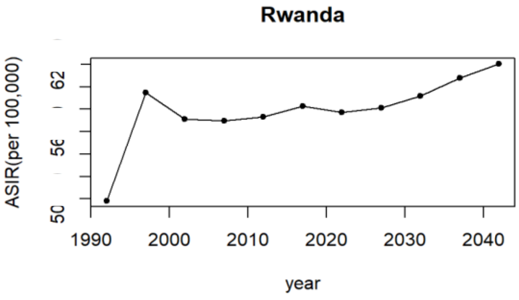
Rwanda
ASIR(per 100,000)
50
56
62
1990
2000
2010
2020
2030
2040
year

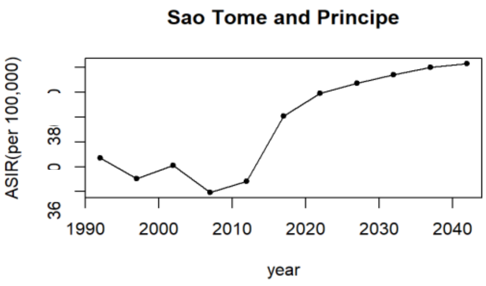
Sao Tome and Principe
ASIR(per 100,000)
36
38
1990
2000
2010
2020
2030
2040
year

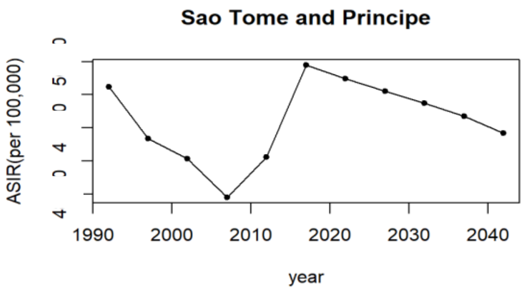
Sao Tome and Principe
ASIR(per 100,000)
4
4
5
1990
2000
2010
2020
2030
2040
year

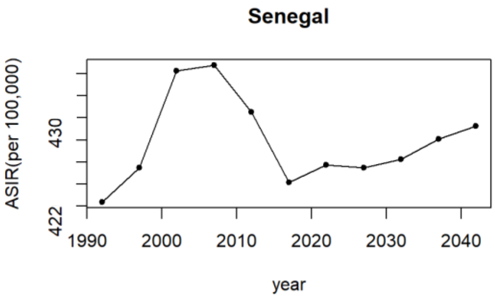
Senegal
ASIR(per 100,000)
422
430
1990
2000
2010
2020
2030
2040
year

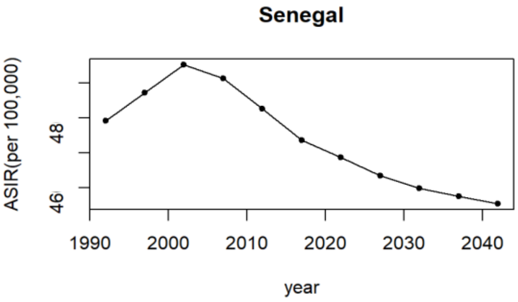
Senegal
ASIR(per 100,000)
46
48
1990
2000
2010
2020
2030
2040
year

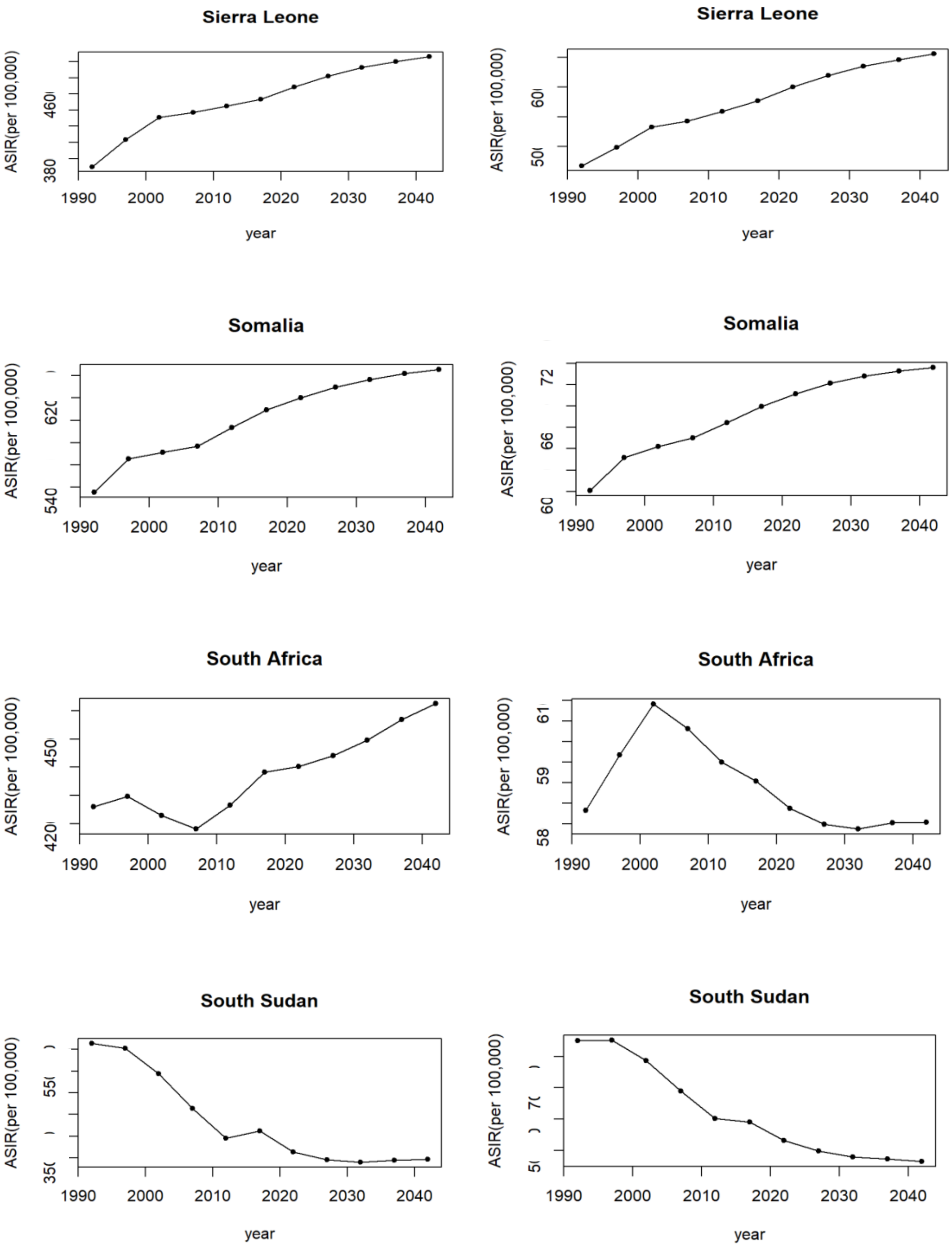
Sierra Leone
ASIR(per 100,000)
year
Sierra Leone
ASIR(per 100,000)
year
Somalia
ASIR(per 100,000)
year
Somalia
ASIR(per 100,000)
year
South Africa
ASIR(per 100,000)
year
South Africa
ASIR(per 100,000)
year
South Sudan
ASIR(per 100,000)
year
South Sudan
ASIR(per 100,000)
year

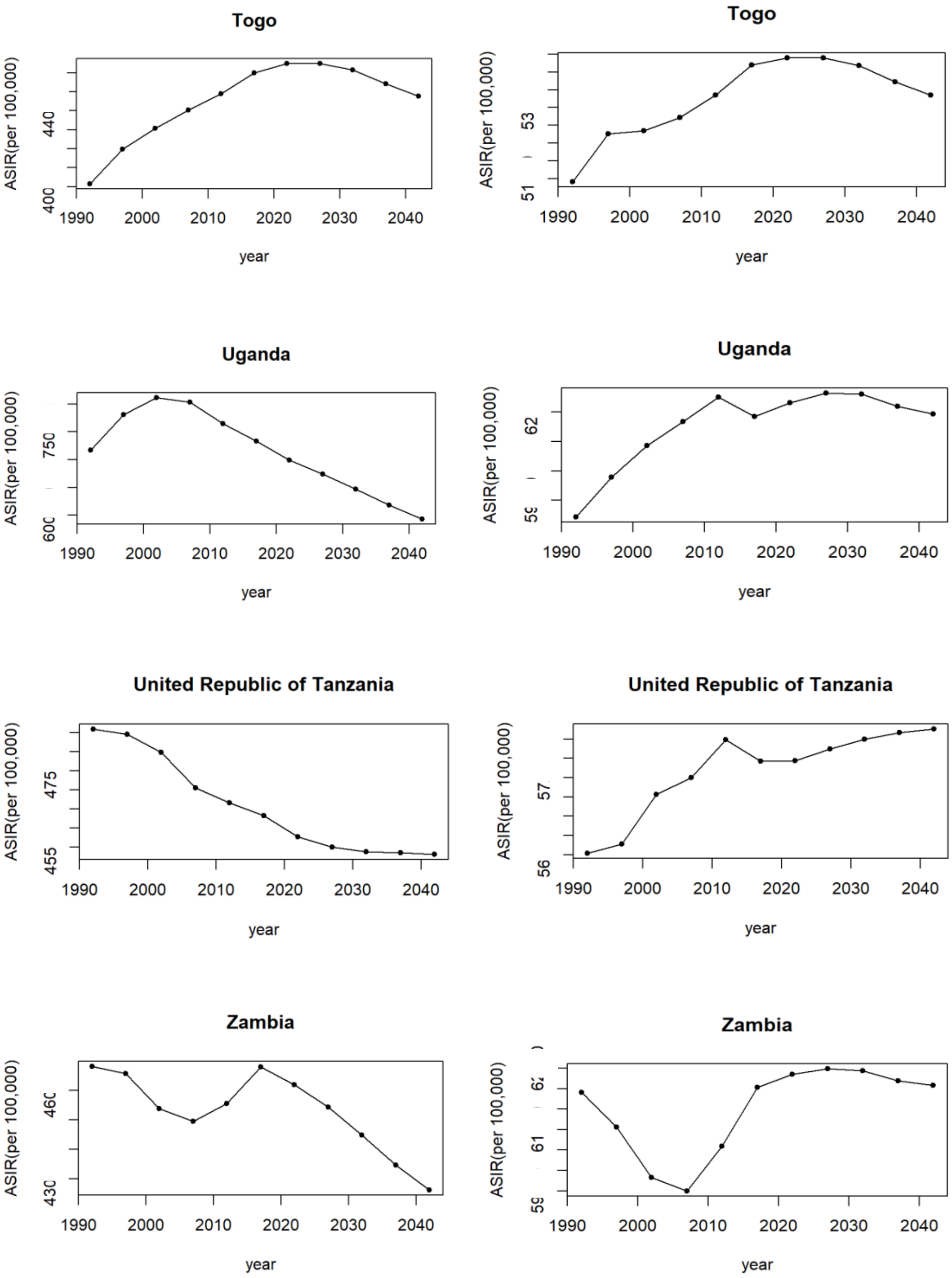
Togo
ASIR(per 100,000)
400
440
1990
2000
2010
2020
2030
2040
year
Togo
ASIR(per 100,000)
51
53
1990
2000
2010
2020
2030
2040
year
Uganda
ASIR(per 100,000)
600
750
1990
2000
2010
2020
2030
2040
year
Uganda
ASIR(per 100,000)
62
1990
2000
2010
2020
2030
2040
year
United Republic of Tanzania
ASIR(per 100,000)
455
475
1990
2000
2010
2020
2030
2040
year
United Republic of Tanzania
ASIR(per 100,000)
56
57
1990
2000
2010
2020
2030
2040
year
Zambia
ASIR(per 100,000)
430
1990
2000
2010
2020
2030
2040
year
Zambia
ASIR(per 100,000)
61
1990
2000
2010
2020
2030
2040
year

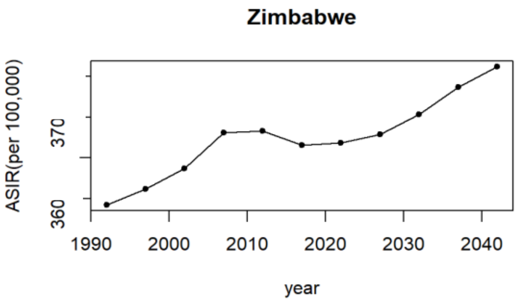
Zimbabwe
ASIR(per 100,000)
360
370
1990
2000
2010
2020
2030
2040
year

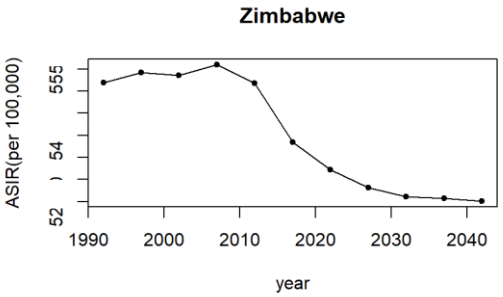
Zimbabwe
ASIR(per 100,000)
52
54
555
1990
2000
2010
2020
2030
2040
year
